# Supplementary material for: ­Comparative spigot ontogeny across the spider tree of life
Source: PeerJ. 2018 Jan 15;6:e4233. doi: 10.7717/peerj.4233 (PMC5772386; doi:10.7717/peerj.4233)
Supplement: Table S1 — This includes average numbers of each spigot for each instar, as well as categorical data of foraging strategies, silk used, maximum number of instars and a score of the diversity of silk spigots each species possesses. 1, Wąsowska, 1977; 2, Hajer, 1991; 3, RE Alfaro, 2017, unpublished data; 4, R Carlson & CE Griswold, 1996, unpublished data; 5, Yu & Coddington, 1990, 6, Townley & Tillinghast, 2009; and 7, Dolejš et al., 2014. [file peerj-06-4233-s001.docx]

**Table S1**: **Full ontogeny dataset of thirteen spider families comprising 22 species**. This includes average numbers of each spigot for each instar, as well as categorical data of foraging strategies, silk used, maximum number of instars and a score of the diversity of silk spigots each species possesses. 1 = Wąsowska 1977; 2 = Hajer 1991, 3 = RE Alfaro, unpublished data, 4 = R Carlson & CE Griswold, unpublished data, 5 = Yu & Coddington 1990, 6 = Townley & Tillinghast 2009, and 7 = Dolejš et al. 2014.

| **Species** | **Sex** | **Instar** | **Spinneret** | **Spigot** | **Number** | **Foraging Strategy** | **Specific Strategy** | **Type of Silk** | **Spigot Type** | **Max # Instars** |
| --- | --- | --- | --- | --- | --- | --- | --- | --- | --- | --- |
| *Tibellus oblongus^1^* | NA | 2 | Cribellum | Cribellar | NA | No web | Sit & Pursue | No | 1 | 6 |
| *Tibellus oblongus^1^* | NA | 2 | ALS | MAP | 0 | No web | Sit & Pursue | No | 1 | 6 |
| *Tibellus oblongus^1^* | NA | 2 | ALS | Piriform | 8 | No web | Sit & Pursue | No | 1 | 6 |
| *Tibellus oblongus^1^* | NA | 2 | PMS | mAP | 0 | No web | Sit & Pursue | No | 1 | 6 |
| *Tibellus oblongus^1^* | NA | 2 | PMS | Aciniform | 4 | No web | Sit & Pursue | No | 1 | 6 |
| *Tibellus oblongus^1^* | NA | 2 | PMS | Cylindrical | 0 | No web | Sit & Pursue | No | 1 | 6 |
| *Tibellus oblongus^1^* | NA | 2 | PLS | Aciniform | 7 | No web | Sit & Pursue | No | 1 | 6 |
| *Tibellus oblongus^1^* | NA | 2 | PLS | Cylindrical | 0 | No web | Sit & Pursue | No | 1 | 6 |
| *Tibellus oblongus^1^* | NA | 3 | Cribellum | Cribellar | NA | No web | Sit & Pursue | No | 1 | 6 |
| *Tibellus oblongus^1^* | NA | 3 | ALS | MAP | 2 | No web | Sit & Pursue | No | 1 | 6 |
| *Tibellus oblongus^1^* | NA | 3 | ALS | Piriform | 18 | No web | Sit & Pursue | No | 1 | 6 |
| *Tibellus oblongus^1^* | NA | 3 | PMS | mAP | 2 | No web | Sit & Pursue | No | 1 | 6 |
| *Tibellus oblongus^1^* | NA | 3 | PMS | Aciniform | 10 | No web | Sit & Pursue | No | 1 | 6 |
| *Tibellus oblongus^1^* | NA | 3 | PMS | Cylindrical | 0 | No web | Sit & Pursue | No | 1 | 6 |
| *Tibellus oblongus^1^* | NA | 3 | PLS | Aciniform | 15 | No web | Sit & Pursue | No | 1 | 6 |
| *Tibellus oblongus^1^* | NA | 3 | PLS | Cylindrical | 0 | No web | Sit & Pursue | No | 1 | 6 |
| *Tibellus oblongus^1^* | NA | 4 | Cribellum | Cribellar | NA | No web | Sit & Pursue | No | 1 | 6 |
| *Tibellus oblongus^1^* | NA | 4 | ALS | MAP | 2 | No web | Sit & Pursue | No | 1 | 6 |
| *Tibellus oblongus^1^* | NA | 4 | ALS | Piriform | 20 | No web | Sit & Pursue | No | 1 | 6 |
| *Tibellus oblongus^1^* | NA | 4 | PMS | mAP | 2 | No web | Sit & Pursue | No | 1 | 6 |
| *Tibellus oblongus^1^* | NA | 4 | PMS | Aciniform | 12 | No web | Sit & Pursue | No | 1 | 6 |
| *Tibellus oblongus^1^* | NA | 4 | PMS | Cylindrical | 0 | No web | Sit & Pursue | No | 1 | 6 |
| *Tibellus oblongus^1^* | NA | 4 | PLS | Aciniform | 16 | No web | Sit & Pursue | No | 1 | 6 |
| *Tibellus oblongus^1^* | NA | 4 | PLS | Cylindrical | 0 | No web | Sit & Pursue | No | 1 | 6 |
| *Tibellus oblongus^1^* | Pen Male | 5 | Cribellum | Cribellar | NA | No web | Sit & Pursue | No | 1 | 6 |
| *Tibellus oblongus^1^* | Pen Male | 5 | ALS | MAP | 2 | No web | Sit & Pursue | No | 1 | 6 |
| *Tibellus oblongus^1^* | Pen Male | 5 | ALS | Piriform | 21 | No web | Sit & Pursue | No | 1 | 6 |
| *Tibellus oblongus^1^* | Pen Male | 5 | PMS | mAP | 2 | No web | Sit & Pursue | No | 1 | 6 |
| *Tibellus oblongus^1^* | Pen Male | 5 | PMS | Aciniform | 13 | No web | Sit & Pursue | No | 1 | 6 |
| *Tibellus oblongus^1^* | Pen Male | 5 | PMS | Cylindrical | 0 | No web | Sit & Pursue | No | 1 | 6 |
| *Tibellus oblongus^1^* | Pen Male | 5 | PLS | Aciniform | 21 | No web | Sit & Pursue | No | 1 | 6 |
| *Tibellus oblongus^1^* | Pen Male | 5 | PLS | Cylindrical | 0 | No web | Sit & Pursue | No | 1 | 6 |
| *Tibellus oblongus^1^* | Pen Fem | 5 | Cribellum | Cribellar | NA | No web | Sit & Pursue | No | 1 | 6 |
| *Tibellus oblongus^1^* | Pen Fem | 5 | ALS | MAP | 2 | No web | Sit & Pursue | No | 1 | 6 |
| *Tibellus oblongus^1^* | Pen Fem | 5 | ALS | Piriform | 23 | No web | Sit & Pursue | No | 1 | 6 |
| *Tibellus oblongus^1^* | Pen Fem | 5 | PMS | mAP | 2 | No web | Sit & Pursue | No | 1 | 6 |
| *Tibellus oblongus^1^* | Pen Fem | 5 | PMS | Aciniform | 13 | No web | Sit & Pursue | No | 1 | 6 |
| *Tibellus oblongus^1^* | Pen Fem | 5 | PMS | Cylindrical | 0 | No web | Sit & Pursue | No | 1 | 6 |
| *Tibellus oblongus^1^* | Pen Fem | 5 | PLS | Aciniform | 26 | No web | Sit & Pursue | No | 1 | 6 |
| *Tibellus oblongus^1^* | Pen Fem | 5 | PLS | Cylindrical | 0 | No web | Sit & Pursue | No | 1 | 6 |
| *Tibellus oblongus^1^* | Male | 6 | Cribellum | Cribellar | NA | No web | Sit & Pursue | No | 1 | 6 |
| *Tibellus oblongus^1^* | Male | 6 | ALS | MAP | 1 | No web | Sit & Pursue | No | 1 | 6 |
| *Tibellus oblongus^1^* | Male | 6 | ALS | Piriform | 21 | No web | Sit & Pursue | No | 1 | 6 |
| *Tibellus oblongus^1^* | Male | 6 | PMS | mAP | 1 | No web | Sit & Pursue | No | 1 | 6 |
| *Tibellus oblongus^1^* | Male | 6 | PMS | Aciniform | 12 | No web | Sit & Pursue | No | 1 | 6 |
| *Tibellus oblongus^1^* | Male | 6 | PMS | Cylindrical | 0 | No web | Sit & Pursue | No | 1 | 6 |
| *Tibellus oblongus^1^* | Male | 6 | PLS | Aciniform | 21 | No web | Sit & Pursue | No | 1 | 6 |
| *Tibellus oblongus^1^* | Male | 6 | PLS | Cylindrical | 0 | No web | Sit & Pursue | No | 1 | 6 |
| *Tibellus oblongus^1^* | Female | 6 | Cribellum | Cribellar | NA | No web | Sit & Pursue | No | 1 | 6 |
| *Tibellus oblongus^1^* | Female | 6 | ALS | MAP | 2 | No web | Sit & Pursue | No | 1 | 6 |
| *Tibellus oblongus^1^* | Female | 6 | ALS | Piriform | 30 | No web | Sit & Pursue | No | 1 | 6 |
| *Tibellus oblongus^1^* | Female | 6 | PMS | mAP | 2 | No web | Sit & Pursue | No | 1 | 6 |
| *Tibellus oblongus^1^* | Female | 6 | PMS | Aciniform | 14 | No web | Sit & Pursue | No | 1 | 6 |
| *Tibellus oblongus^1^* | Female | 6 | PMS | Cylindrical | 2 | No web | Sit & Pursue | No | 1 | 6 |
| *Tibellus oblongus^1^* | Female | 6 | PLS | Aciniform | 25 | No web | Sit & Pursue | No | 1 | 6 |
| *Tibellus oblongus^1^* | Female | 6 | PLS | Cylindrical | 6 | No web | Sit & Pursue | No | 1 | 6 |
| *Xysticus cristatus^1^* | NA | 2 | Cribellum | Cribellar | NA | No web | Ambush | No | 1 | 6 |
| *Xysticus cristatus^1^* | NA | 2 | ALS | MAP | 0 | No web | Ambush | No | 1 | 6 |
| *Xysticus cristatus^1^* | NA | 2 | ALS | Piriform | 6 | No web | Ambush | No | 1 | 6 |
| *Xysticus cristatus^1^* | NA | 2 | PMS | mAP | 0 | No web | Ambush | No | 1 | 6 |
| *Xysticus cristatus^1^* | NA | 2 | PMS | Aciniform | 3 | No web | Ambush | No | 1 | 6 |
| *Xysticus cristatus^1^* | NA | 2 | PMS | Cylindrical | 0 | No web | Ambush | No | 1 | 6 |
| *Xysticus cristatus^1^* | NA | 2 | PLS | Aciniform | 4 | No web | Ambush | No | 1 | 6 |
| *Xysticus cristatus^1^* | NA | 2 | PLS | Cylindrical | 0 | No web | Ambush | No | 1 | 6 |
| *Xysticus cristatus^1^* | NA | 3 | Cribellum | Cribellar | NA | No web | Ambush | No | 1 | 6 |
| *Xysticus cristatus^1^* | NA | 3 | ALS | MAP | 2 | No web | Ambush | No | 1 | 6 |
| *Xysticus cristatus^1^* | NA | 3 | ALS | Piriform | 8 | No web | Ambush | No | 1 | 6 |
| *Xysticus cristatus^1^* | NA | 3 | PMS | mAP | 1 | No web | Ambush | No | 1 | 6 |
| *Xysticus cristatus^1^* | NA | 3 | PMS | Aciniform | 5 | No web | Ambush | No | 1 | 6 |
| *Xysticus cristatus^1^* | NA | 3 | PMS | Cylindrical | 0 | No web | Ambush | No | 1 | 6 |
| *Xysticus cristatus^1^* | NA | 3 | PLS | Aciniform | 9 | No web | Ambush | No | 1 | 6 |
| *Xysticus cristatus^1^* | NA | 3 | PLS | Cylindrical | 0 | No web | Ambush | No | 1 | 6 |
| *Xysticus cristatus^1^* | NA | 4 | Cribellum | Cribellar | NA | No web | Ambush | No | 1 | 6 |
| *Xysticus cristatus^1^* | NA | 4 | ALS | MAP | 2 | No web | Ambush | No | 1 | 6 |
| *Xysticus cristatus^1^* | NA | 4 | ALS | Piriform | 10 | No web | Ambush | No | 1 | 6 |
| *Xysticus cristatus^1^* | NA | 4 | PMS | mAP | 2 | No web | Ambush | No | 1 | 6 |
| *Xysticus cristatus^1^* | NA | 4 | PMS | Aciniform | 8 | No web | Ambush | No | 1 | 6 |
| *Xysticus cristatus^1^* | NA | 4 | PMS | Cylindrical | 0 | No web | Ambush | No | 1 | 6 |
| *Xysticus cristatus^1^* | NA | 4 | PLS | Aciniform | 10 | No web | Ambush | No | 1 | 6 |
| *Xysticus cristatus^1^* | NA | 4 | PLS | Cylindrical | 0 | No web | Ambush | No | 1 | 6 |
| *Xysticus cristatus^1^* | Pen Male | 5 | Cribellum | Cribellar | NA | No web | Ambush | No | 1 | 6 |
| *Xysticus cristatus^1^* | Pen Male | 5 | ALS | MAP | 2 | No web | Ambush | No | 1 | 6 |
| *Xysticus cristatus^1^* | Pen Male | 5 | ALS | Piriform | 16 | No web | Ambush | No | 1 | 6 |
| *Xysticus cristatus^1^* | Pen Male | 5 | PMS | mAP | 2 | No web | Ambush | No | 1 | 6 |
| *Xysticus cristatus^1^* | Pen Male | 5 | PMS | Aciniform | 8 | No web | Ambush | No | 1 | 6 |
| *Xysticus cristatus^1^* | Pen Male | 5 | PMS | Cylindrical | 0 | No web | Ambush | No | 1 | 6 |
| *Xysticus cristatus^1^* | Pen Male | 5 | PLS | Aciniform | 13 | No web | Ambush | No | 1 | 6 |
| *Xysticus cristatus^1^* | Pen Male | 5 | PLS | Cylindrical | 0 | No web | Ambush | No | 1 | 6 |
| *Xysticus cristatus^1^* | Pen Fem | 5 | Cribellum | Cribellar | NA | No web | Ambush | No | 1 | 6 |
| *Xysticus cristatus^1^* | Pen Fem | 5 | ALS | MAP | 2 | No web | Ambush | No | 1 | 6 |
| *Xysticus cristatus^1^* | Pen Fem | 5 | ALS | Piriform | 20 | No web | Ambush | No | 1 | 6 |
| *Xysticus cristatus^1^* | Pen Fem | 5 | PMS | mAP | 2 | No web | Ambush | No | 1 | 6 |
| *Xysticus cristatus^1^* | Pen Fem | 5 | PMS | Aciniform | 11 | No web | Ambush | No | 1 | 6 |
| *Xysticus cristatus^1^* | Pen Fem | 5 | PMS | Cylindrical | 0 | No web | Ambush | No | 1 | 6 |
| *Xysticus cristatus^1^* | Pen Fem | 5 | PLS | Aciniform | 18 | No web | Ambush | No | 1 | 6 |
| *Xysticus cristatus^1^* | Pen Fem | 5 | PLS | Cylindrical | 0 | No web | Ambush | No | 1 | 6 |
| *Xysticus cristatus^1^* | Male | 6 | Cribellum | Cribellar | NA | No web | Ambush | No | 1 | 6 |
| *Xysticus cristatus^1^* | Male | 6 | ALS | MAP | 1 | No web | Ambush | No | 1 | 6 |
| *Xysticus cristatus^1^* | Male | 6 | ALS | Piriform | 18 | No web | Ambush | No | 1 | 6 |
| *Xysticus cristatus^1^* | Male | 6 | PMS | mAP | 1 | No web | Ambush | No | 1 | 6 |
| *Xysticus cristatus^1^* | Male | 6 | PMS | Aciniform | 8 | No web | Ambush | No | 1 | 6 |
| *Xysticus cristatus^1^* | Male | 6 | PMS | Cylindrical | 0 | No web | Ambush | No | 1 | 6 |
| *Xysticus cristatus^1^* | Male | 6 | PLS | Aciniform | 13 | No web | Ambush | No | 1 | 6 |
| *Xysticus cristatus^1^* | Male | 6 | PLS | Cylindrical | 0 | No web | Ambush | No | 1 | 6 |
| *Xysticus cristatus^1^* | Female | 6 | Cribellum | Cribellar | NA | No web | Ambush | No | 1 | 6 |
| *Xysticus cristatus^1^* | Female | 6 | ALS | MAP | 2 | No web | Ambush | No | 1 | 6 |
| *Xysticus cristatus^1^* | Female | 6 | ALS | Piriform | 23 | No web | Ambush | No | 1 | 6 |
| *Xysticus cristatus^1^* | Female | 6 | PMS | mAP | 2 | No web | Ambush | No | 1 | 6 |
| *Xysticus cristatus^1^* | Female | 6 | PMS | Aciniform | 20 | No web | Ambush | No | 1 | 6 |
| *Xysticus cristatus^1^* | Female | 6 | PMS | Cylindrical | 3 | No web | Ambush | No | 1 | 6 |
| *Xysticus cristatus^1^* | Female | 6 | PLS | Aciniform | 24 | No web | Ambush | No | 1 | 6 |
| *Xysticus cristatus^1^* | Female | 6 | PLS | Cylindrical | 11 | No web | Ambush | No | 1 | 6 |
| *Xerolycosa nemoralis^7^* | NA | 2 | Cribellum | Cribellar | NA | No web | Active | No | 1 | 10 |
| *Xerolycosa nemoralis^7^* | NA | 2 | ALS | MAP | 2 | No web | Active | No | 1 | 10 |
| *Xerolycosa nemoralis^7^* | NA | 2 | ALS | Piriform | 3 | No web | Active | No | 1 | 10 |
| *Xerolycosa nemoralis^7^* | NA | 2 | PMS | mAP | 2 | No web | Active | No | 1 | 10 |
| *Xerolycosa nemoralis^7^* | NA | 2 | PMS | Aciniform | 4 | No web | Active | No | 1 | 10 |
| *Xerolycosa nemoralis^7^* | NA | 2 | PMS | Cylindrical | 0 | No web | Active | No | 1 | 10 |
| *Xerolycosa nemoralis^7^* | NA | 2 | PLS | Aciniform | 7 | No web | Active | No | 1 | 10 |
| *Xerolycosa nemoralis^7^* | NA | 2 | PLS | Modified | NA | No web | Active | No | 1 | 10 |
| *Xerolycosa nemoralis^7^* | NA | 2 | PLS | Flanking | NA | No web | Active | No | 1 | 10 |
| *Xerolycosa nemoralis^7^* | NA | 2 | PLS | Cylindrical | 0 | No web | Active | No | 1 | 10 |
| *Xerolycosa nemoralis^7^* | NA | 3 | Cribellum | Cribellar | NA | No web | Active | No | 1 | 10 |
| *Xerolycosa nemoralis^7^* | NA | 3 | ALS | MAP | 2 | No web | Active | No | 1 | 10 |
| *Xerolycosa nemoralis^7^* | NA | 3 | ALS | Piriform | 4 | No web | Active | No | 1 | 10 |
| *Xerolycosa nemoralis^7^* | NA | 3 | PMS | mAP | 2 | No web | Active | No | 1 | 10 |
| *Xerolycosa nemoralis^7^* | NA | 3 | PMS | Aciniform | 6 | No web | Active | No | 1 | 10 |
| *Xerolycosa nemoralis^7^* | NA | 3 | PMS | Cylindrical | 0 | No web | Active | No | 1 | 10 |
| *Xerolycosa nemoralis^7^* | NA | 3 | PLS | Aciniform | 9 | No web | Active | No | 1 | 10 |
| *Xerolycosa nemoralis^7^* | NA | 3 | PLS | Modified | NA | No web | Active | No | 1 | 10 |
| *Xerolycosa nemoralis^7^* | NA | 3 | PLS | Flanking | NA | No web | Active | No | 1 | 10 |
| *Xerolycosa nemoralis^7^* | NA | 3 | PLS | Cylindrical | 0 | No web | Active | No | 1 | 10 |
| *Xerolycosa nemoralis^7^* | NA | 4 | Cribellum | Cribellar | NA | No web | Active | No | 1 | 10 |
| *Xerolycosa nemoralis^7^* | NA | 4 | ALS | MAP | 2 | No web | Active | No | 1 | 10 |
| *Xerolycosa nemoralis^7^* | NA | 4 | ALS | Piriform | 5 | No web | Active | No | 1 | 10 |
| *Xerolycosa nemoralis^7^* | NA | 4 | PMS | mAP | 2 | No web | Active | No | 1 | 10 |
| *Xerolycosa nemoralis^7^* | NA | 4 | PMS | Aciniform | 7 | No web | Active | No | 1 | 10 |
| *Xerolycosa nemoralis^7^* | NA | 4 | PMS | Cylindrical | 0 | No web | Active | No | 1 | 10 |
| *Xerolycosa nemoralis^7^* | NA | 4 | PLS | Aciniform | 12 | No web | Active | No | 1 | 10 |
| *Xerolycosa nemoralis^7^* | NA | 4 | PLS | Modified | NA | No web | Active | No | 1 | 10 |
| *Xerolycosa nemoralis^7^* | NA | 4 | PLS | Flanking | NA | No web | Active | No | 1 | 10 |
| *Xerolycosa nemoralis^7^* | NA | 4 | PLS | Cylindrical | 0 | No web | Active | No | 1 | 10 |
| *Xerolycosa nemoralis^7^* | NA | 5 | Cribellum | Cribellar | NA | No web | Active | No | 1 | 10 |
| *Xerolycosa nemoralis^7^* | NA | 5 | ALS | MAP | 2 | No web | Active | No | 1 | 10 |
| *Xerolycosa nemoralis^7^* | NA | 5 | ALS | Piriform | 7 | No web | Active | No | 1 | 10 |
| *Xerolycosa nemoralis^7^* | NA | 5 | PMS | mAP | 2 | No web | Active | No | 1 | 10 |
| *Xerolycosa nemoralis^7^* | NA | 5 | PMS | Aciniform | 8 | No web | Active | No | 1 | 10 |
| *Xerolycosa nemoralis^7^* | NA | 5 | PMS | Cylindrical | 0 | No web | Active | No | 1 | 10 |
| *Xerolycosa nemoralis^7^* | NA | 5 | PLS | Aciniform | 15 | No web | Active | No | 1 | 10 |
| *Xerolycosa nemoralis^7^* | NA | 5 | PLS | Modified | NA | No web | Active | No | 1 | 10 |
| *Xerolycosa nemoralis^7^* | NA | 5 | PLS | Flanking | NA | No web | Active | No | 1 | 10 |
| *Xerolycosa nemoralis^7^* | NA | 5 | PLS | Cylindrical | 0 | No web | Active | No | 1 | 10 |
| *Xerolycosa nemoralis^7^* | NA | 6 | Cribellum | Cribellar | NA | No web | Active | No | 1 | 10 |
| *Xerolycosa nemoralis^7^* | NA | 6 | ALS | MAP | 2 | No web | Active | No | 1 | 10 |
| *Xerolycosa nemoralis^7^* | NA | 6 | ALS | Piriform | 9 | No web | Active | No | 1 | 10 |
| *Xerolycosa nemoralis^7^* | NA | 6 | PMS | mAP | 2 | No web | Active | No | 1 | 10 |
| *Xerolycosa nemoralis^7^* | NA | 6 | PMS | Aciniform | 12 | No web | Active | No | 1 | 10 |
| *Xerolycosa nemoralis^7^* | NA | 6 | PMS | Cylindrical | 0 | No web | Active | No | 1 | 10 |
| *Xerolycosa nemoralis^7^* | NA | 6 | PLS | Aciniform | 22 | No web | Active | No | 1 | 10 |
| *Xerolycosa nemoralis^7^* | NA | 6 | PLS | Modified | NA | No web | Active | No | 1 | 10 |
| *Xerolycosa nemoralis^7^* | NA | 6 | PLS | Flanking | NA | No web | Active | No | 1 | 10 |
| *Xerolycosa nemoralis^7^* | NA | 6 | PLS | Cylindrical | 0 | No web | Active | No | 1 | 10 |
| *Xerolycosa nemoralis^7^* | NA | 7 | Cribellum | Cribellar | NA | No web | Active | No | 1 | 10 |
| *Xerolycosa nemoralis^7^* | NA | 7 | ALS | MAP | 2 | No web | Active | No | 1 | 10 |
| *Xerolycosa nemoralis^7^* | NA | 7 | ALS | Piriform | 12 | No web | Active | No | 1 | 10 |
| *Xerolycosa nemoralis^7^* | NA | 7 | PMS | mAP | 2 | No web | Active | No | 1 | 10 |
| *Xerolycosa nemoralis^7^* | NA | 7 | PMS | Aciniform | 15 | No web | Active | No | 1 | 10 |
| *Xerolycosa nemoralis^7^* | NA | 7 | PMS | Cylindrical | 0 | No web | Active | No | 1 | 10 |
| *Xerolycosa nemoralis^7^* | NA | 7 | PLS | Aciniform | 26 | No web | Active | No | 1 | 10 |
| *Xerolycosa nemoralis^7^* | NA | 7 | PLS | Modified | NA | No web | Active | No | 1 | 10 |
| *Xerolycosa nemoralis^7^* | NA | 7 | PLS | Flanking | NA | No web | Active | No | 1 | 10 |
| *Xerolycosa nemoralis^7^* | NA | 7 | PLS | Cylindrical | 0 | No web | Active | No | 1 | 10 |
| *Xerolycosa nemoralis^7^* | NA | 8 | Cribellum | Cribellar | NA | No web | Active | No | 1 | 10 |
| *Xerolycosa nemoralis^7^* | NA | 8 | ALS | MAP | 2 | No web | Active | No | 1 | 10 |
| *Xerolycosa nemoralis^7^* | NA | 8 | ALS | Piriform | 15 | No web | Active | No | 1 | 10 |
| *Xerolycosa nemoralis^7^* | NA | 8 | PMS | mAP | 2 | No web | Active | No | 1 | 10 |
| *Xerolycosa nemoralis^7^* | NA | 8 | PMS | Aciniform | 21 | No web | Active | No | 1 | 10 |
| *Xerolycosa nemoralis^7^* | NA | 8 | PMS | Cylindrical | 0 | No web | Active | No | 1 | 10 |
| *Xerolycosa nemoralis^7^* | NA | 8 | PLS | Aciniform | 31 | No web | Active | No | 1 | 10 |
| *Xerolycosa nemoralis^7^* | NA | 8 | PLS | Modified | NA | No web | Active | No | 1 | 10 |
| *Xerolycosa nemoralis^7^* | NA | 8 | PLS | Flanking | NA | No web | Active | No | 1 | 10 |
| *Xerolycosa nemoralis^7^* | NA | 8 | PLS | Cylindrical | 0 | No web | Active | No | 1 | 10 |
| *Xerolycosa nemoralis^7^* | Pen Male | 9 | Cribellum | Cribellar | NA | No web | Active | No | 1 | 10 |
| *Xerolycosa nemoralis^7^* | Pen Male | 9 | ALS | MAP | 2 | No web | Active | No | 1 | 10 |
| *Xerolycosa nemoralis^7^* | Pen Male | 9 | ALS | Piriform | 14 | No web | Active | No | 1 | 10 |
| *Xerolycosa nemoralis^7^* | Pen Male | 9 | PMS | mAP | 2 | No web | Active | No | 1 | 10 |
| *Xerolycosa nemoralis^7^* | Pen Male | 9 | PMS | Aciniform | 18 | No web | Active | No | 1 | 10 |
| *Xerolycosa nemoralis^7^* | Pen Male | 9 | PMS | Cylindrical | 0 | No web | Active | No | 1 | 10 |
| *Xerolycosa nemoralis^7^* | Pen Male | 9 | PLS | Aciniform | 32 | No web | Active | No | 1 | 10 |
| *Xerolycosa nemoralis^7^* | Pen Male | 9 | PLS | Modified | NA | No web | Active | No | 1 | 10 |
| *Xerolycosa nemoralis^7^* | Pen Male | 9 | PLS | Flanking | NA | No web | Active | No | 1 | 10 |
| *Xerolycosa nemoralis^7^* | Pen Male | 9 | PLS | Cylindrical | 0 | No web | Active | No | 1 | 10 |
| *Xerolycosa nemoralis^7^* | Pen Fem | 9 | Cribellum | Cribellar | NA | No web | Active | No | 1 | 10 |
| *Xerolycosa nemoralis^7^* | Pen Fem | 9 | ALS | MAP | 2 | No web | Active | No | 1 | 10 |
| *Xerolycosa nemoralis^7^* | Pen Fem | 9 | ALS | Piriform | 15 | No web | Active | No | 1 | 10 |
| *Xerolycosa nemoralis^7^* | Pen Fem | 9 | PMS | mAP | 2 | No web | Active | No | 1 | 10 |
| *Xerolycosa nemoralis^7^* | Pen Fem | 9 | PMS | Aciniform | 22 | No web | Active | No | 1 | 10 |
| *Xerolycosa nemoralis^7^* | Pen Fem | 9 | PMS | Cylindrical | 0 | No web | Active | No | 1 | 10 |
| *Xerolycosa nemoralis^7^* | Pen Fem | 9 | PLS | Aciniform | 34 | No web | Active | No | 1 | 10 |
| *Xerolycosa nemoralis^7^* | Pen Fem | 9 | PLS | Modified | NA | No web | Active | No | 1 | 10 |
| *Xerolycosa nemoralis^7^* | Pen Fem | 9 | PLS | Flanking | NA | No web | Active | No | 1 | 10 |
| *Xerolycosa nemoralis^7^* | Pen Fem | 9 | PLS | Cylindrical | 0 | No web | Active | No | 1 | 10 |
| *Xerolycosa nemoralis^7^* | Male | 10 | Cribellum | Cribellar | NA | No web | Active | No | 1 | 10 |
| *Xerolycosa nemoralis^7^* | Male | 10 | ALS | MAP | 1 | No web | Active | No | 1 | 10 |
| *Xerolycosa nemoralis^7^* | Male | 10 | ALS | Piriform | 10 | No web | Active | No | 1 | 10 |
| *Xerolycosa nemoralis^7^* | Male | 10 | PMS | mAP | 1 | No web | Active | No | 1 | 10 |
| *Xerolycosa nemoralis^7^* | Male | 10 | PMS | Aciniform | 14 | No web | Active | No | 1 | 10 |
| *Xerolycosa nemoralis^7^* | Male | 10 | PMS | Cylindrical | 0 | No web | Active | No | 1 | 10 |
| *Xerolycosa nemoralis^7^* | Male | 10 | PLS | Aciniform | 29 | No web | Active | No | 1 | 10 |
| *Xerolycosa nemoralis^7^* | Male | 10 | PLS | Modified | NA | No web | Active | No | 1 | 10 |
| *Xerolycosa nemoralis^7^* | Male | 10 | PLS | Flanking | NA | No web | Active | No | 1 | 10 |
| *Xerolycosa nemoralis^7^* | Male | 10 | PLS | Cylindrical | 0 | No web | Active | No | 1 | 10 |
| *Xerolycosa nemoralis^7^* | Female | 10 | Cribellum | Cribellar | NA | No web | Active | No | 1 | 10 |
| *Xerolycosa nemoralis^7^* | Female | 10 | ALS | MAP | 2 | No web | Active | No | 1 | 10 |
| *Xerolycosa nemoralis^7^* | Female | 10 | ALS | Piriform | 23 | No web | Active | No | 1 | 10 |
| *Xerolycosa nemoralis^7^* | Female | 10 | PMS | mAP | 2 | No web | Active | No | 1 | 10 |
| *Xerolycosa nemoralis^7^* | Female | 10 | PMS | Aciniform | 30 | No web | Active | No | 1 | 10 |
| *Xerolycosa nemoralis^7^* | Female | 10 | PMS | Cylindrical | 7 | No web | Active | No | 1 | 10 |
| *Xerolycosa nemoralis^7^* | Female | 10 | PLS | Aciniform | 45 | No web | Active | No | 1 | 10 |
| *Xerolycosa nemoralis^7^* | Female | 10 | PLS | Modified | NA | No web | Active | No | 1 | 10 |
| *Xerolycosa nemoralis^7^* | Female | 10 | PLS | Flanking | NA | No web | Active | No | 1 | 10 |
| *Xerolycosa nemoralis^7^* | Female | 10 | PLS | Cylindrical | 1 | No web | Active | No | 1 | 10 |
| *Pardosa lugubris^1,7^* | NA | 2 | Cribellum | Cribellar | NA | No web | Active | No | 1 | 7 |
| *Pardosa lugubris^1,7^* | NA | 2 | ALS | MAP | 2 | No web | Active | No | 1 | 7 |
| *Pardosa lugubris^1,7^* | NA | 2 | ALS | Piriform | 3 | No web | Active | No | 1 | 7 |
| *Pardosa lugubris^1,7^* | NA | 2 | PMS | mAP | 2 | No web | Active | No | 1 | 7 |
| *Pardosa lugubris^1,7^* | NA | 2 | PMS | Aciniform | 4 | No web | Active | No | 1 | 7 |
| *Pardosa lugubris^1,7^* | NA | 2 | PMS | Cylindrical | 0 | No web | Active | No | 1 | 7 |
| *Pardosa lugubris^1,7^* | NA | 2 | PLS | Aciniform | 0 | No web | Active | No | 1 | 7 |
| *Pardosa lugubris^1,7^* | NA | 2 | PLS | Modified | NA | No web | Active | No | 1 | 7 |
| *Pardosa lugubris^1,7^* | NA | 2 | PLS | Flanking | NA | No web | Active | No | 1 | 7 |
| *Pardosa lugubris^1,7^* | NA | 2 | PLS | Cylindrical | 0 | No web | Active | No | 1 | 7 |
| *Pardosa lugubris^1,7^* | NA | 3 | Cribellum | Cribellar | NA | No web | Active | No | 1 | 7 |
| *Pardosa lugubris^1,7^* | NA | 3 | ALS | MAP | 2 | No web | Active | No | 1 | 7 |
| *Pardosa lugubris^1,7^* | NA | 3 | ALS | Piriform | 7 | No web | Active | No | 1 | 7 |
| *Pardosa lugubris^1,7^* | NA | 3 | PMS | mAP | 2 | No web | Active | No | 1 | 7 |
| *Pardosa lugubris^1,7^* | NA | 3 | PMS | Aciniform | 4 | No web | Active | No | 1 | 7 |
| *Pardosa lugubris^1,7^* | NA | 3 | PMS | Cylindrical | 0 | No web | Active | No | 1 | 7 |
| *Pardosa lugubris^1,7^* | NA | 3 | PLS | Aciniform | 10 | No web | Active | No | 1 | 7 |
| *Pardosa lugubris^1,7^* | NA | 3 | PLS | Modified | NA | No web | Active | No | 1 | 7 |
| *Pardosa lugubris^1,7^* | NA | 3 | PLS | Flanking | NA | No web | Active | No | 1 | 7 |
| *Pardosa lugubris^1,7^* | NA | 3 | PLS | Cylindrical | 0 | No web | Active | No | 1 | 7 |
| *Pardosa lugubris^1,7^* | NA | 4 | Cribellum | Cribellar | NA | No web | Active | No | 1 | 7 |
| *Pardosa lugubris^1,7^* | NA | 4 | ALS | MAP | 2 | No web | Active | No | 1 | 7 |
| *Pardosa lugubris^1,7^* | NA | 4 | ALS | Piriform | 12 | No web | Active | No | 1 | 7 |
| *Pardosa lugubris^1,7^* | NA | 4 | PMS | mAP | 2 | No web | Active | No | 1 | 7 |
| *Pardosa lugubris^1,7^* | NA | 4 | PMS | Aciniform | 9 | No web | Active | No | 1 | 7 |
| *Pardosa lugubris^1,7^* | NA | 4 | PMS | Cylindrical | 0 | No web | Active | No | 1 | 7 |
| *Pardosa lugubris^1,7^* | NA | 4 | PLS | Aciniform | 13 | No web | Active | No | 1 | 7 |
| *Pardosa lugubris^1,7^* | NA | 4 | PLS | Modified | NA | No web | Active | No | 1 | 7 |
| *Pardosa lugubris^1,7^* | NA | 4 | PLS | Flanking | NA | No web | Active | No | 1 | 7 |
| *Pardosa lugubris^1,7^* | NA | 4 | PLS | Cylindrical | 0 | No web | Active | No | 1 | 7 |
| *Pardosa lugubris^1,7^* | NA | 5 | Cribellum | Cribellar | NA | No web | Active | No | 1 | 7 |
| *Pardosa lugubris^1,7^* | NA | 5 | ALS | MAP | 2 | No web | Active | No | 1 | 7 |
| *Pardosa lugubris^1,7^* | NA | 5 | ALS | Piriform | 17 | No web | Active | No | 1 | 7 |
| *Pardosa lugubris^1,7^* | NA | 5 | PMS | mAP | 2 | No web | Active | No | 1 | 7 |
| *Pardosa lugubris^1,7^* | NA | 5 | PMS | Aciniform | 14 | No web | Active | No | 1 | 7 |
| *Pardosa lugubris^1,7^* | NA | 5 | PMS | Cylindrical | 0 | No web | Active | No | 1 | 7 |
| *Pardosa lugubris^1,7^* | NA | 5 | PLS | Aciniform | 15 | No web | Active | No | 1 | 7 |
| *Pardosa lugubris^1,7^* | NA | 5 | PLS | Modified | NA | No web | Active | No | 1 | 7 |
| *Pardosa lugubris^1,7^* | NA | 5 | PLS | Flanking | NA | No web | Active | No | 1 | 7 |
| *Pardosa lugubris^1,7^* | NA | 5 | PLS | Cylindrical | 0 | No web | Active | No | 1 | 7 |
| *Pardosa lugubris^1,7^* | Pen Male | 6 | Cribellum | Cribellar | NA | No web | Active | No | 1 | 7 |
| *Pardosa lugubris^1,7^* | Pen Male | 6 | ALS | MAP | 2 | No web | Active | No | 1 | 7 |
| *Pardosa lugubris^1,7^* | Pen Male | 6 | ALS | Piriform | 17 | No web | Active | No | 1 | 7 |
| *Pardosa lugubris^1,7^* | Pen Male | 6 | PMS | mAP | 2 | No web | Active | No | 1 | 7 |
| *Pardosa lugubris^1,7^* | Pen Male | 6 | PMS | Aciniform | 14 | No web | Active | No | 1 | 7 |
| *Pardosa lugubris^1,7^* | Pen Male | 6 | PMS | Cylindrical | 0 | No web | Active | No | 1 | 7 |
| *Pardosa lugubris^1,7^* | Pen Male | 6 | PLS | Aciniform | 15 | No web | Active | No | 1 | 7 |
| *Pardosa lugubris^1,7^* | Pen Male | 6 | PLS | Modified | NA | No web | Active | No | 1 | 7 |
| *Pardosa lugubris^1,7^* | Pen Male | 6 | PLS | Flanking | NA | No web | Active | No | 1 | 7 |
| *Pardosa lugubris^1,7^* | Pen Male | 6 | PLS | Cylindrical | 0 | No web | Active | No | 1 | 7 |
| *Pardosa lugubris^1,7^* | Pen Fem | 6 | Cribellum | Cribellar | NA | No web | Active | No | 1 | 7 |
| *Pardosa lugubris^1,7^* | Pen Fem | 6 | ALS | MAP | 2 | No web | Active | No | 1 | 7 |
| *Pardosa lugubris^1,7^* | Pen Fem | 6 | ALS | Piriform | 23 | No web | Active | No | 1 | 7 |
| *Pardosa lugubris^1,7^* | Pen Fem | 6 | PMS | mAP | 2 | No web | Active | No | 1 | 7 |
| *Pardosa lugubris^1,7^* | Pen Fem | 6 | PMS | Aciniform | 21 | No web | Active | No | 1 | 7 |
| *Pardosa lugubris^1,7^* | Pen Fem | 6 | PMS | Cylindrical | 0 | No web | Active | No | 1 | 7 |
| *Pardosa lugubris^1,7^* | Pen Fem | 6 | PLS | Aciniform | 26 | No web | Active | No | 1 | 7 |
| *Pardosa lugubris^1,7^* | Pen Fem | 6 | PLS | Modified | NA | No web | Active | No | 1 | 7 |
| *Pardosa lugubris^1,7^* | Pen Fem | 6 | PLS | Flanking | NA | No web | Active | No | 1 | 7 |
| *Pardosa lugubris^1,7^* | Pen Fem | 6 | PLS | Cylindrical | 0 | No web | Active | No | 1 | 7 |
| *Pardosa lugubris^1,7^* | Male | 7 | Cribellum | Cribellar | NA | No web | Active | No | 1 | 7 |
| *Pardosa lugubris^1,7^* | Male | 7 | ALS | MAP | 1 | No web | Active | No | 1 | 7 |
| *Pardosa lugubris^1,7^* | Male | 7 | ALS | Piriform | 16 | No web | Active | No | 1 | 7 |
| *Pardosa lugubris^1,7^* | Male | 7 | PMS | mAP | 1 | No web | Active | No | 1 | 7 |
| *Pardosa lugubris^1,7^* | Male | 7 | PMS | Aciniform | 15 | No web | Active | No | 1 | 7 |
| *Pardosa lugubris^1,7^* | Male | 7 | PMS | Cylindrical | 0 | No web | Active | No | 1 | 7 |
| *Pardosa lugubris^1,7^* | Male | 7 | PLS | Aciniform | 14 | No web | Active | No | 1 | 7 |
| *Pardosa lugubris^1,7^* | Male | 7 | PLS | Modified | NA | No web | Active | No | 1 | 7 |
| *Pardosa lugubris^1,7^* | Male | 7 | PLS | Flanking | NA | No web | Active | No | 1 | 7 |
| *Pardosa lugubris^1,7^* | Male | 7 | PLS | Cylindrical | 0 | No web | Active | No | 1 | 7 |
| *Pardosa lugubris^1,7^* | Female | 7 | Cribellum | Cribellar | NA | No web | Active | No | 1 | 7 |
| *Pardosa lugubris^1,7^* | Female | 7 | ALS | MAP | 2 | No web | Active | No | 1 | 7 |
| *Pardosa lugubris^1,7^* | Female | 7 | ALS | Piriform | 24 | No web | Active | No | 1 | 7 |
| *Pardosa lugubris^1,7^* | Female | 7 | PMS | mAP | 2 | No web | Active | No | 1 | 7 |
| *Pardosa lugubris^1,7^* | Female | 7 | PMS | Aciniform | 28 | No web | Active | No | 1 | 7 |
| *Pardosa lugubris^1,7^* | Female | 7 | PMS | Cylindrical | 9 | No web | Active | No | 1 | 7 |
| *Pardosa lugubris^1,7^* | Female | 7 | PLS | Aciniform | 34 | No web | Active | No | 1 | 7 |
| *Pardosa lugubris^1,7^* | Female | 7 | PLS | Modified | NA | No web | Active | No | 1 | 7 |
| *Pardosa lugubris^1,7^* | Female | 7 | PLS | Flanking | NA | No web | Active | No | 1 | 7 |
| *Pardosa lugubris^1,7^* | Female | 7 | PLS | Cylindrical | 13 | No web | Active | No | 1 | 7 |
| *Pardosa amentata^7^* | NA | 2 | Cribellum | Cribellar | NA | No web | Active | No | 1 | 9 |
| *Pardosa amentata^7^* | NA | 2 | ALS | MAP | 2 | No web | Active | No | 1 | 9 |
| *Pardosa amentata^7^* | NA | 2 | ALS | Piriform | 4 | No web | Active | No | 1 | 9 |
| *Pardosa amentata^7^* | NA | 2 | PMS | mAP | 2 | No web | Active | No | 1 | 9 |
| *Pardosa amentata^7^* | NA | 2 | PMS | Aciniform | 2 | No web | Active | No | 1 | 9 |
| *Pardosa amentata^7^* | NA | 2 | PMS | Cylindrical | 0 | No web | Active | No | 1 | 9 |
| *Pardosa amentata^7^* | NA | 2 | PLS | Aciniform | 5 | No web | Active | No | 1 | 9 |
| *Pardosa amentata^7^* | NA | 2 | PLS | Modified | NA | No web | Active | No | 1 | 9 |
| *Pardosa amentata^7^* | NA | 2 | PLS | Flanking | NA | No web | Active | No | 1 | 9 |
| *Pardosa amentata^7^* | NA | 2 | PLS | Cylindrical | 0 | No web | Active | No | 1 | 9 |
| *Pardosa amentata^7^* | NA | 3 | Cribellum | Cribellar | NA | No web | Active | No | 1 | 9 |
| *Pardosa amentata^7^* | NA | 3 | ALS | MAP | 2 | No web | Active | No | 1 | 9 |
| *Pardosa amentata^7^* | NA | 3 | ALS | Piriform | 5 | No web | Active | No | 1 | 9 |
| *Pardosa amentata^7^* | NA | 3 | PMS | mAP | 2 | No web | Active | No | 1 | 9 |
| *Pardosa amentata^7^* | NA | 3 | PMS | Aciniform | 3 | No web | Active | No | 1 | 9 |
| *Pardosa amentata^7^* | NA | 3 | PMS | Cylindrical | 0 | No web | Active | No | 1 | 9 |
| *Pardosa amentata^7^* | NA | 3 | PLS | Aciniform | 6 | No web | Active | No | 1 | 9 |
| *Pardosa amentata^7^* | NA | 3 | PLS | Modified | NA | No web | Active | No | 1 | 9 |
| *Pardosa amentata^7^* | NA | 3 | PLS | Flanking | NA | No web | Active | No | 1 | 9 |
| *Pardosa amentata^7^* | NA | 3 | PLS | Cylindrical | 0 | No web | Active | No | 1 | 9 |
| *Pardosa amentata^7^* | NA | 4 | Cribellum | Cribellar | NA | No web | Active | No | 1 | 9 |
| *Pardosa amentata^7^* | NA | 4 | ALS | MAP | 2 | No web | Active | No | 1 | 9 |
| *Pardosa amentata^7^* | NA | 4 | ALS | Piriform | 6 | No web | Active | No | 1 | 9 |
| *Pardosa amentata^7^* | NA | 4 | PMS | mAP | 2 | No web | Active | No | 1 | 9 |
| *Pardosa amentata^7^* | NA | 4 | PMS | Aciniform | 5 | No web | Active | No | 1 | 9 |
| *Pardosa amentata^7^* | NA | 4 | PMS | Cylindrical | 0 | No web | Active | No | 1 | 9 |
| *Pardosa amentata^7^* | NA | 4 | PLS | Aciniform | 7 | No web | Active | No | 1 | 9 |
| *Pardosa amentata^7^* | NA | 4 | PLS | Modified | NA | No web | Active | No | 1 | 9 |
| *Pardosa amentata^7^* | NA | 4 | PLS | Flanking | NA | No web | Active | No | 1 | 9 |
| *Pardosa amentata^7^* | NA | 4 | PLS | Cylindrical | 0 | No web | Active | No | 1 | 9 |
| *Pardosa amentata^7^* | NA | 5 | Cribellum | Cribellar | NA | No web | Active | No | 1 | 9 |
| *Pardosa amentata^7^* | NA | 5 | ALS | MAP | 2 | No web | Active | No | 1 | 9 |
| *Pardosa amentata^7^* | NA | 5 | ALS | Piriform | 6 | No web | Active | No | 1 | 9 |
| *Pardosa amentata^7^* | NA | 5 | PMS | mAP | 2 | No web | Active | No | 1 | 9 |
| *Pardosa amentata^7^* | NA | 5 | PMS | Aciniform | 6 | No web | Active | No | 1 | 9 |
| *Pardosa amentata^7^* | NA | 5 | PMS | Cylindrical | 0 | No web | Active | No | 1 | 9 |
| *Pardosa amentata^7^* | NA | 5 | PLS | Aciniform | 8 | No web | Active | No | 1 | 9 |
| *Pardosa amentata^7^* | NA | 5 | PLS | Modified | NA | No web | Active | No | 1 | 9 |
| *Pardosa amentata^7^* | NA | 5 | PLS | Flanking | NA | No web | Active | No | 1 | 9 |
| *Pardosa amentata^7^* | NA | 5 | PLS | Cylindrical | 0 | No web | Active | No | 1 | 9 |
| *Pardosa amentata^7^* | NA | 6 | Cribellum | Cribellar | NA | No web | Active | No | 1 | 9 |
| *Pardosa amentata^7^* | NA | 6 | ALS | MAP | 2 | No web | Active | No | 1 | 9 |
| *Pardosa amentata^7^* | NA | 6 | ALS | Piriform | 7 | No web | Active | No | 1 | 9 |
| *Pardosa amentata^7^* | NA | 6 | PMS | mAP | 2 | No web | Active | No | 1 | 9 |
| *Pardosa amentata^7^* | NA | 6 | PMS | Aciniform | 8 | No web | Active | No | 1 | 9 |
| *Pardosa amentata^7^* | NA | 6 | PMS | Cylindrical | 0 | No web | Active | No | 1 | 9 |
| *Pardosa amentata^7^* | NA | 6 | PLS | Aciniform | 10 | No web | Active | No | 1 | 9 |
| *Pardosa amentata^7^* | NA | 6 | PLS | Modified | NA | No web | Active | No | 1 | 9 |
| *Pardosa amentata^7^* | NA | 6 | PLS | Flanking | NA | No web | Active | No | 1 | 9 |
| *Pardosa amentata^7^* | NA | 6 | PLS | Cylindrical | 0 | No web | Active | No | 1 | 9 |
| *Pardosa amentata^7^* | NA | 7 | Cribellum | Cribellar | NA | No web | Active | No | 1 | 9 |
| *Pardosa amentata^7^* | NA | 7 | ALS | MAP | 2 | No web | Active | No | 1 | 9 |
| *Pardosa amentata^7^* | NA | 7 | ALS | Piriform | 10 | No web | Active | No | 1 | 9 |
| *Pardosa amentata^7^* | NA | 7 | PMS | mAP | 2 | No web | Active | No | 1 | 9 |
| *Pardosa amentata^7^* | Ante Pen Male | 7 | PMS | Aciniform | 10 | No web | Active | No | 1 | 9 |
| *Pardosa amentata^7^* | Ante Pen Fem | 7 | PMS | Aciniform | 15 | No web | Active | No | 1 | 9 |
| *Pardosa amentata^7^* | NA | 7 | PMS | Cylindrical | 0 | No web | Active | No | 1 | 9 |
| *Pardosa amentata^7^* | NA | 7 | PLS | Aciniform | 13 | No web | Active | No | 1 | 9 |
| *Pardosa amentata^7^* | NA | 7 | PLS | Modified | NA | No web | Active | No | 1 | 9 |
| *Pardosa amentata^7^* | NA | 7 | PLS | Flanking | NA | No web | Active | No | 1 | 9 |
| *Pardosa amentata^7^* | NA | 7 | PLS | Cylindrical | 0 | No web | Active | No | 1 | 9 |
| *Pardosa amentata^7^* | Pen Male | 8 | Cribellum | Cribellar | NA | No web | Active | No | 1 | 9 |
| *Pardosa amentata^7^* | Pen Male | 8 | ALS | MAP | 2 | No web | Active | No | 1 | 9 |
| *Pardosa amentata^7^* | Pen Male | 8 | ALS | Piriform | 13 | No web | Active | No | 1 | 9 |
| *Pardosa amentata^7^* | Pen Male | 8 | PMS | mAP | 2 | No web | Active | No | 1 | 9 |
| *Pardosa amentata^7^* | Pen Male | 8 | PMS | Aciniform | 12 | No web | Active | No | 1 | 9 |
| *Pardosa amentata^7^* | Pen Male | 8 | PMS | Cylindrical | 0 | No web | Active | No | 1 | 9 |
| *Pardosa amentata^7^* | Pen Male | 8 | PLS | Aciniform | 16 | No web | Active | No | 1 | 9 |
| *Pardosa amentata^7^* | Pen Male | 8 | PLS | Modified | NA | No web | Active | No | 1 | 9 |
| *Pardosa amentata^7^* | Pen Male | 8 | PLS | Flanking | NA | No web | Active | No | 1 | 9 |
| *Pardosa amentata^7^* | Pen Male | 8 | PLS | Cylindrical | 0 | No web | Active | No | 1 | 9 |
| *Pardosa amentata^7^* | Pen Fem | 8 | Cribellum | Cribellar | NA | No web | Active | No | 1 | 9 |
| *Pardosa amentata^7^* | Pen Fem | 8 | ALS | MAP | 2 | No web | Active | No | 1 | 9 |
| *Pardosa amentata^7^* | Pen Fem | 8 | ALS | Piriform | 17 | No web | Active | No | 1 | 9 |
| *Pardosa amentata^7^* | Pen Fem | 8 | PMS | mAP | 2 | No web | Active | No | 1 | 9 |
| *Pardosa amentata^7^* | Pen Fem | 8 | PMS | Aciniform | 23 | No web | Active | No | 1 | 9 |
| *Pardosa amentata^7^* | Pen Fem | 8 | PMS | Cylindrical | 0 | No web | Active | No | 1 | 9 |
| *Pardosa amentata^7^* | Pen Fem | 8 | PLS | Aciniform | 27 | No web | Active | No | 1 | 9 |
| *Pardosa amentata^7^* | Pen Fem | 8 | PLS | Modified | NA | No web | Active | No | 1 | 9 |
| *Pardosa amentata^7^* | Pen Fem | 8 | PLS | Flanking | NA | No web | Active | No | 1 | 9 |
| *Pardosa amentata^7^* | Pen Fem | 8 | PLS | Cylindrical | 0 | No web | Active | No | 1 | 9 |
| *Pardosa amentata^7^* | Male | 9 | Cribellum | Cribellar | NA | No web | Active | No | 1 | 9 |
| *Pardosa amentata^7^* | Male | 9 | ALS | MAP | 1 | No web | Active | No | 1 | 9 |
| *Pardosa amentata^7^* | Male | 9 | ALS | Piriform | 18 | No web | Active | No | 1 | 9 |
| *Pardosa amentata^7^* | Male | 9 | PMS | mAP | 1 | No web | Active | No | 1 | 9 |
| *Pardosa amentata^7^* | Male | 9 | PMS | Aciniform | 17 | No web | Active | No | 1 | 9 |
| *Pardosa amentata^7^* | Male | 9 | PMS | Cylindrical | 0 | No web | Active | No | 1 | 9 |
| *Pardosa amentata^7^* | Male | 9 | PLS | Aciniform | 20 | No web | Active | No | 1 | 9 |
| *Pardosa amentata^7^* | Male | 9 | PLS | Modified | NA | No web | Active | No | 1 | 9 |
| *Pardosa amentata^7^* | Male | 9 | PLS | Flanking | NA | No web | Active | No | 1 | 9 |
| *Pardosa amentata^7^* | Male | 9 | PLS | Cylindrical | 0 | No web | Active | No | 1 | 9 |
| *Pardosa amentata^7^* | Female | 9 | Cribellum | Cribellar | NA | No web | Active | No | 1 | 9 |
| *Pardosa amentata^7^* | Female | 9 | ALS | MAP | 2 | No web | Active | No | 1 | 9 |
| *Pardosa amentata^7^* | Female | 9 | ALS | Piriform | 29 | No web | Active | No | 1 | 9 |
| *Pardosa amentata^7^* | Female | 9 | PMS | mAP | 2 | No web | Active | No | 1 | 9 |
| *Pardosa amentata^7^* | Female | 9 | PMS | Aciniform | 18 | No web | Active | No | 1 | 9 |
| *Pardosa amentata^7^* | Female | 9 | PMS | Cylindrical | 21 | No web | Active | No | 1 | 9 |
| *Pardosa amentata^7^* | Female | 9 | PLS | Aciniform | 46 | No web | Active | No | 1 | 9 |
| *Pardosa amentata^7^* | Female | 9 | PLS | Modified | NA | No web | Active | No | 1 | 9 |
| *Pardosa amentata^7^* | Female | 9 | PLS | Flanking | NA | No web | Active | No | 1 | 9 |
| *Pardosa amentata^7^* | Female | 9 | PLS | Cylindrical | 1 | No web | Active | No | 1 | 9 |
| *Hogna carolinensis^3^* | NA | 2 | Cribellum | Cribellar | NA | No web | Sit & Pursue | No | 2 | 12 |
| *Hogna carolinensis^3^* | NA | 2 | ALS | MAP | 2 | No web | Sit & Pursue | No | 2 | 12 |
| *Hogna carolinensis^3^* | NA | 2 | ALS | Piriform | 4 | No web | Sit & Pursue | No | 2 | 12 |
| *Hogna carolinensis^3^* | NA | 2 | PMS | mAP | 2 | No web | Sit & Pursue | No | 2 | 12 |
| *Hogna carolinensis^3^* | NA | 2 | PMS | Aciniform | 4 | No web | Sit & Pursue | No | 2 | 12 |
| *Hogna carolinensis^3^* | NA | 2 | PMS | Cylindrical | 0 | No web | Sit & Pursue | No | 2 | 12 |
| *Hogna carolinensis^3^* | NA | 2 | PLS | Aciniform | 7 | No web | Sit & Pursue | No | 2 | 12 |
| *Hogna carolinensis^3^* | NA | 2 | PLS | Modified | 0 | No web | Sit & Pursue | No | 2 | 12 |
| *Hogna carolinensis^3^* | NA | 2 | PLS | Flanking | 0 | No web | Sit & Pursue | No | 2 | 12 |
| *Hogna carolinensis^3^* | NA | 2 | PLS | Cylindrical | 0 | No web | Sit & Pursue | No | 2 | 12 |
| *Hogna carolinensis^3^* | NA | 3 | Cribellum | Cribellar | NA | No web | Sit & Pursue | No | 2 | 12 |
| *Hogna carolinensis^3^* | NA | 3 | ALS | MAP | 2 | No web | Sit & Pursue | No | 2 | 12 |
| *Hogna carolinensis^3^* | NA | 3 | ALS | Piriform | 7 | No web | Sit & Pursue | No | 2 | 12 |
| *Hogna carolinensis^3^* | NA | 3 | PMS | mAP | 2 | No web | Sit & Pursue | No | 2 | 12 |
| *Hogna carolinensis^3^* | NA | 3 | PMS | Aciniform | 6 | No web | Sit & Pursue | No | 2 | 12 |
| *Hogna carolinensis^3^* | NA | 3 | PMS | Cylindrical | 0 | No web | Sit & Pursue | No | 2 | 12 |
| *Hogna carolinensis^3^* | NA | 3 | PLS | Aciniform | 9 | No web | Sit & Pursue | No | 2 | 12 |
| *Hogna carolinensis^3^* | NA | 3 | PLS | Modified | 0 | No web | Sit & Pursue | No | 2 | 12 |
| *Hogna carolinensis^3^* | NA | 3 | PLS | Flanking | 0 | No web | Sit & Pursue | No | 2 | 12 |
| *Hogna carolinensis^3^* | NA | 3 | PLS | Cylindrical | 0 | No web | Sit & Pursue | No | 2 | 12 |
| *Hogna carolinensis^3^* | NA | 4 | Cribellum | Cribellar | NA | No web | Sit & Pursue | No | 2 | 12 |
| *Hogna carolinensis^3^* | NA | 4 | ALS | MAP | 2 | No web | Sit & Pursue | No | 2 | 12 |
| *Hogna carolinensis^3^* | NA | 4 | ALS | Piriform | 11 | No web | Sit & Pursue | No | 2 | 12 |
| *Hogna carolinensis^3^* | NA | 4 | PMS | mAP | 2 | No web | Sit & Pursue | No | 2 | 12 |
| *Hogna carolinensis^3^* | NA | 4 | PMS | Aciniform | 3 | No web | Sit & Pursue | No | 2 | 12 |
| *Hogna carolinensis^3^* | NA | 4 | PMS | Cylindrical | 0 | No web | Sit & Pursue | No | 2 | 12 |
| *Hogna carolinensis^3^* | NA | 4 | PLS | Aciniform | 3 | No web | Sit & Pursue | No | 2 | 12 |
| *Hogna carolinensis^3^* | NA | 4 | PLS | Modified | 0 | No web | Sit & Pursue | No | 2 | 12 |
| *Hogna carolinensis^3^* | NA | 4 | PLS | Flanking | 0 | No web | Sit & Pursue | No | 2 | 12 |
| *Hogna carolinensis^3^* | NA | 4 | PLS | Cylindrical | 0 | No web | Sit & Pursue | No | 2 | 12 |
| *Hogna carolinensis^3^* | NA | 5 | Cribellum | Cribellar | NA | No web | Sit & Pursue | No | 2 | 12 |
| *Hogna carolinensis^3^* | NA | 5 | ALS | MAP | 2 | No web | Sit & Pursue | No | 2 | 12 |
| *Hogna carolinensis^3^* | NA | 5 | ALS | Piriform | 13 | No web | Sit & Pursue | No | 2 | 12 |
| *Hogna carolinensis^3^* | NA | 5 | PMS | mAP | 2 | No web | Sit & Pursue | No | 2 | 12 |
| *Hogna carolinensis^3^* | NA | 5 | PMS | Aciniform | 3 | No web | Sit & Pursue | No | 2 | 12 |
| *Hogna carolinensis^3^* | NA | 5 | PMS | Cylindrical | 0 | No web | Sit & Pursue | No | 2 | 12 |
| *Hogna carolinensis^3^* | NA | 5 | PLS | Aciniform | 3 | No web | Sit & Pursue | No | 2 | 12 |
| *Hogna carolinensis^3^* | NA | 5 | PLS | Modified | 0 | No web | Sit & Pursue | No | 2 | 12 |
| *Hogna carolinensis^3^* | NA | 5 | PLS | Flanking | 0 | No web | Sit & Pursue | No | 2 | 12 |
| *Hogna carolinensis^3^* | NA | 5 | PLS | Cylindrical | 0 | No web | Sit & Pursue | No | 2 | 12 |
| *Hogna carolinensis^3^* | NA | 6 | Cribellum | Cribellar | NA | No web | Sit & Pursue | No | 2 | 12 |
| *Hogna carolinensis^3^* | NA | 6 | ALS | MAP | 2 | No web | Sit & Pursue | No | 2 | 12 |
| *Hogna carolinensis^3^* | NA | 6 | ALS | Piriform | 17 | No web | Sit & Pursue | No | 2 | 12 |
| *Hogna carolinensis^3^* | NA | 6 | PMS | mAP | 2 | No web | Sit & Pursue | No | 2 | 12 |
| *Hogna carolinensis^3^* | NA | 6 | PMS | Aciniform | 6 | No web | Sit & Pursue | No | 2 | 12 |
| *Hogna carolinensis^3^* | NA | 6 | PMS | Cylindrical | 0 | No web | Sit & Pursue | No | 2 | 12 |
| *Hogna carolinensis^3^* | NA | 6 | PLS | Aciniform | 7 | No web | Sit & Pursue | No | 2 | 12 |
| *Hogna carolinensis^3^* | NA | 6 | PLS | Modified | 0 | No web | Sit & Pursue | No | 2 | 12 |
| *Hogna carolinensis^3^* | NA | 6 | PLS | Flanking | 0 | No web | Sit & Pursue | No | 2 | 12 |
| *Hogna carolinensis^3^* | NA | 6 | PLS | Cylindrical | 0 | No web | Sit & Pursue | No | 2 | 12 |
| *Hogna carolinensis^3^* | NA | 7 | Cribellum | Cribellar | NA | No web | Sit & Pursue | No | 2 | 12 |
| *Hogna carolinensis^3^* | NA | 7 | ALS | MAP | 2 | No web | Sit & Pursue | No | 2 | 12 |
| *Hogna carolinensis^3^* | NA | 7 | ALS | Piriform | 27 | No web | Sit & Pursue | No | 2 | 12 |
| *Hogna carolinensis^3^* | NA | 7 | PMS | mAP | 2 | No web | Sit & Pursue | No | 2 | 12 |
| *Hogna carolinensis^3^* | NA | 7 | PMS | Aciniform | 4 | No web | Sit & Pursue | No | 2 | 12 |
| *Hogna carolinensis^3^* | NA | 7 | PMS | Cylindrical | 0 | No web | Sit & Pursue | No | 2 | 12 |
| *Hogna carolinensis^3^* | NA | 7 | PLS | Aciniform | 7 | No web | Sit & Pursue | No | 2 | 12 |
| *Hogna carolinensis^3^* | NA | 7 | PLS | Modified | 0 | No web | Sit & Pursue | No | 2 | 12 |
| *Hogna carolinensis^3^* | NA | 7 | PLS | Flanking | 0 | No web | Sit & Pursue | No | 2 | 12 |
| *Hogna carolinensis^3^* | NA | 7 | PLS | Cylindrical | -1 | No web | Sit & Pursue | No | 2 | 12 |
| *Hogna carolinensis^3^* | Female | 12 | Cribellum | Cribellar | NA | No web | Sit & Pursue | No | 2 | 12 |
| *Hogna carolinensis^3^* | Female | 12 | ALS | MAP | 2 | No web | Sit & Pursue | No | 2 | 12 |
| *Hogna carolinensis^3^* | Female | 12 | ALS | Piriform | 122 | No web | Sit & Pursue | No | 2 | 12 |
| *Hogna carolinensis^3^* | Female | 12 | PMS | mAP | 2 | No web | Sit & Pursue | No | 2 | 12 |
| *Hogna carolinensis^3^* | Female | 12 | PMS | Aciniform | 82 | No web | Sit & Pursue | No | 2 | 12 |
| *Hogna carolinensis^3^* | Female | 12 | PMS | Cylindrical | 10 | No web | Sit & Pursue | No | 2 | 12 |
| *Hogna carolinensis^3^* | Female | 12 | PLS | Aciniform | 43 | No web | Sit & Pursue | No | 2 | 12 |
| *Hogna carolinensis^3^* | Female | 12 | PLS | Modified | 1 | No web | Sit & Pursue | No | 2 | 12 |
| *Hogna carolinensis^3^* | Female | 12 | PLS | Flanking | 0 | No web | Sit & Pursue | No | 2 | 12 |
| *Hogna carolinensis^3^* | Female | 12 | PLS | Cylindrical | 1 | No web | Sit & Pursue | No | 2 | 12 |
| *Arctosa lutetiana^7^* | NA | 2 | Cribellum | Cribellar | NA | No web | Ambush | Burrow | 1 | 9 |
| *Arctosa lutetiana^7^* | NA | 2 | ALS | MAP | 2 | No web | Ambush | Burrow | 1 | 9 |
| *Arctosa lutetiana^7^* | NA | 2 | ALS | Piriform | 5 | No web | Ambush | Burrow | 1 | 9 |
| *Arctosa lutetiana^7^* | NA | 2 | PMS | mAP | 2 | No web | Ambush | Burrow | 1 | 9 |
| *Arctosa lutetiana^7^* | NA | 2 | PMS | Aciniform | 3 | No web | Ambush | Burrow | 1 | 9 |
| *Arctosa lutetiana^7^* | NA | 2 | PMS | Cylindrical | 0 | No web | Ambush | Burrow | 1 | 9 |
| *Arctosa lutetiana^7^* | NA | 2 | PLS | Aciniform | 4 | No web | Ambush | Burrow | 1 | 9 |
| *Arctosa lutetiana^7^* | NA | 2 | PLS | Modified | NA | No web | Ambush | Burrow | 1 | 9 |
| *Arctosa lutetiana^7^* | NA | 2 | PLS | Flanking | NA | No web | Ambush | Burrow | 1 | 9 |
| *Arctosa lutetiana^7^* | NA | 2 | PLS | Cylindrical | 0 | No web | Ambush | Burrow | 1 | 9 |
| *Arctosa lutetiana^7^* | NA | 3 | Cribellum | Cribellar | NA | No web | Ambush | Burrow | 1 | 9 |
| *Arctosa lutetiana^7^* | NA | 3 | ALS | MAP | 2 | No web | Ambush | Burrow | 1 | 9 |
| *Arctosa lutetiana^7^* | NA | 3 | ALS | Piriform | 5 | No web | Ambush | Burrow | 1 | 9 |
| *Arctosa lutetiana^7^* | NA | 3 | PMS | mAP | 2 | No web | Ambush | Burrow | 1 | 9 |
| *Arctosa lutetiana^7^* | NA | 3 | PMS | Aciniform | 3 | No web | Ambush | Burrow | 1 | 9 |
| *Arctosa lutetiana^7^* | NA | 3 | PMS | Cylindrical | 0 | No web | Ambush | Burrow | 1 | 9 |
| *Arctosa lutetiana^7^* | NA | 3 | PLS | Aciniform | 5 | No web | Ambush | Burrow | 1 | 9 |
| *Arctosa lutetiana^7^* | NA | 3 | PLS | Modified | NA | No web | Ambush | Burrow | 1 | 9 |
| *Arctosa lutetiana^7^* | NA | 3 | PLS | Flanking | NA | No web | Ambush | Burrow | 1 | 9 |
| *Arctosa lutetiana^7^* | NA | 3 | PLS | Cylindrical | 0 | No web | Ambush | Burrow | 1 | 9 |
| *Arctosa lutetiana^7^* | NA | 4 | Cribellum | Cribellar | NA | No web | Ambush | Burrow | 1 | 9 |
| *Arctosa lutetiana^7^* | NA | 4 | ALS | MAP | 2 | No web | Ambush | Burrow | 1 | 9 |
| *Arctosa lutetiana^7^* | NA | 4 | ALS | Piriform | 6 | No web | Ambush | Burrow | 1 | 9 |
| *Arctosa lutetiana^7^* | NA | 4 | PMS | mAP | 2 | No web | Ambush | Burrow | 1 | 9 |
| *Arctosa lutetiana^7^* | NA | 4 | PMS | Aciniform | 4 | No web | Ambush | Burrow | 1 | 9 |
| *Arctosa lutetiana^7^* | NA | 4 | PMS | Cylindrical | 0 | No web | Ambush | Burrow | 1 | 9 |
| *Arctosa lutetiana^7^* | NA | 4 | PLS | Aciniform | 6 | No web | Ambush | Burrow | 1 | 9 |
| *Arctosa lutetiana^7^* | NA | 4 | PLS | Modified | NA | No web | Ambush | Burrow | 1 | 9 |
| *Arctosa lutetiana^7^* | NA | 4 | PLS | Flanking | NA | No web | Ambush | Burrow | 1 | 9 |
| *Arctosa lutetiana^7^* | NA | 4 | PLS | Cylindrical | 0 | No web | Ambush | Burrow | 1 | 9 |
| *Arctosa lutetiana^7^* | NA | 5 | Cribellum | Cribellar | NA | No web | Ambush | Burrow | 1 | 9 |
| *Arctosa lutetiana^7^* | NA | 5 | ALS | MAP | 2 | No web | Ambush | Burrow | 1 | 9 |
| *Arctosa lutetiana^7^* | NA | 5 | ALS | Piriform | 8 | No web | Ambush | Burrow | 1 | 9 |
| *Arctosa lutetiana^7^* | NA | 5 | PMS | mAP | 2 | No web | Ambush | Burrow | 1 | 9 |
| *Arctosa lutetiana^7^* | NA | 5 | PMS | Aciniform | 5 | No web | Ambush | Burrow | 1 | 9 |
| *Arctosa lutetiana^7^* | NA | 5 | PMS | Cylindrical | 0 | No web | Ambush | Burrow | 1 | 9 |
| *Arctosa lutetiana^7^* | NA | 5 | PLS | Aciniform | 8 | No web | Ambush | Burrow | 1 | 9 |
| *Arctosa lutetiana^7^* | NA | 5 | PLS | Modified | NA | No web | Ambush | Burrow | 1 | 9 |
| *Arctosa lutetiana^7^* | NA | 5 | PLS | Flanking | NA | No web | Ambush | Burrow | 1 | 9 |
| *Arctosa lutetiana^7^* | NA | 5 | PLS | Cylindrical | 0 | No web | Ambush | Burrow | 1 | 9 |
| *Arctosa lutetiana^7^* | NA | 6 | Cribellum | Cribellar | NA | No web | Ambush | Burrow | 1 | 9 |
| *Arctosa lutetiana^7^* | NA | 6 | ALS | MAP | 2 | No web | Ambush | Burrow | 1 | 9 |
| *Arctosa lutetiana^7^* | NA | 6 | ALS | Piriform | 9 | No web | Ambush | Burrow | 1 | 9 |
| *Arctosa lutetiana^7^* | NA | 6 | PMS | mAP | 2 | No web | Ambush | Burrow | 1 | 9 |
| *Arctosa lutetiana^7^* | NA | 6 | PMS | Aciniform | 5 | No web | Ambush | Burrow | 1 | 9 |
| *Arctosa lutetiana^7^* | NA | 6 | PMS | Cylindrical | 0 | No web | Ambush | Burrow | 1 | 9 |
| *Arctosa lutetiana^7^* | NA | 6 | PLS | Aciniform | 10 | No web | Ambush | Burrow | 1 | 9 |
| *Arctosa lutetiana^7^* | NA | 6 | PLS | Modified | NA | No web | Ambush | Burrow | 1 | 9 |
| *Arctosa lutetiana^7^* | NA | 6 | PLS | Flanking | NA | No web | Ambush | Burrow | 1 | 9 |
| *Arctosa lutetiana^7^* | NA | 6 | PLS | Cylindrical | 0 | No web | Ambush | Burrow | 1 | 9 |
| *Arctosa lutetiana^7^* | NA | 7 | Cribellum | Cribellar | NA | No web | Ambush | Burrow | 1 | 9 |
| *Arctosa lutetiana^7^* | NA | 7 | ALS | MAP | 2 | No web | Ambush | Burrow | 1 | 9 |
| *Arctosa lutetiana^7^* | NA | 7 | ALS | Piriform | 11 | No web | Ambush | Burrow | 1 | 9 |
| *Arctosa lutetiana^7^* | NA | 7 | PMS | mAP | 2 | No web | Ambush | Burrow | 1 | 9 |
| *Arctosa lutetiana^7^* | NA | 7 | PMS | Aciniform | 8 | No web | Ambush | Burrow | 1 | 9 |
| *Arctosa lutetiana^7^* | NA | 7 | PMS | Cylindrical | 0 | No web | Ambush | Burrow | 1 | 9 |
| *Arctosa lutetiana^7^* | NA | 7 | PLS | Aciniform | 15 | No web | Ambush | Burrow | 1 | 9 |
| *Arctosa lutetiana^7^* | NA | 7 | PLS | Modified | NA | No web | Ambush | Burrow | 1 | 9 |
| *Arctosa lutetiana^7^* | NA | 7 | PLS | Flanking | NA | No web | Ambush | Burrow | 1 | 9 |
| *Arctosa lutetiana^7^* | NA | 7 | PLS | Cylindrical | 0 | No web | Ambush | Burrow | 1 | 9 |
| *Arctosa lutetiana^7^* | Pen Male | 8 | Cribellum | Cribellar | NA | No web | Ambush | Burrow | 1 | 9 |
| *Arctosa lutetiana^7^* | Pen Male | 8 | ALS | MAP | 2 | No web | Ambush | Burrow | 1 | 9 |
| *Arctosa lutetiana^7^* | Pen Male | 8 | ALS | Piriform | 12 | No web | Ambush | Burrow | 1 | 9 |
| *Arctosa lutetiana^7^* | Pen Male | 8 | PMS | mAP | 2 | No web | Ambush | Burrow | 1 | 9 |
| *Arctosa lutetiana^7^* | Pen Male | 8 | PMS | Aciniform | 8 | No web | Ambush | Burrow | 1 | 9 |
| *Arctosa lutetiana^7^* | Pen Male | 8 | PMS | Cylindrical | 0 | No web | Ambush | Burrow | 1 | 9 |
| *Arctosa lutetiana^7^* | Pen Male | 8 | PLS | Aciniform | 13 | No web | Ambush | Burrow | 1 | 9 |
| *Arctosa lutetiana^7^* | Pen Male | 8 | PLS | Modified | NA | No web | Ambush | Burrow | 1 | 9 |
| *Arctosa lutetiana^7^* | Pen Male | 8 | PLS | Flanking | NA | No web | Ambush | Burrow | 1 | 9 |
| *Arctosa lutetiana^7^* | Pen Male | 8 | PLS | Cylindrical | 0 | No web | Ambush | Burrow | 1 | 9 |
| *Arctosa lutetiana^7^* | Pen Fem | 8 | Cribellum | Cribellar | NA | No web | Ambush | Burrow | 1 | 9 |
| *Arctosa lutetiana^7^* | Pen Fem | 8 | ALS | MAP | 2 | No web | Ambush | Burrow | 1 | 9 |
| *Arctosa lutetiana^7^* | Pen Fem | 8 | ALS | Piriform | 15 | No web | Ambush | Burrow | 1 | 9 |
| *Arctosa lutetiana^7^* | Pen Fem | 8 | PMS | mAP | 2 | No web | Ambush | Burrow | 1 | 9 |
| *Arctosa lutetiana^7^* | Pen Fem | 8 | PMS | Aciniform | 10 | No web | Ambush | Burrow | 1 | 9 |
| *Arctosa lutetiana^7^* | Pen Fem | 8 | PMS | Cylindrical | 0 | No web | Ambush | Burrow | 1 | 9 |
| *Arctosa lutetiana^7^* | Pen Fem | 8 | PLS | Aciniform | 19 | No web | Ambush | Burrow | 1 | 9 |
| *Arctosa lutetiana^7^* | Pen Fem | 8 | PLS | Modified | NA | No web | Ambush | Burrow | 1 | 9 |
| *Arctosa lutetiana^7^* | Pen Fem | 8 | PLS | Flanking | NA | No web | Ambush | Burrow | 1 | 9 |
| *Arctosa lutetiana^7^* | Pen Fem | 8 | PLS | Cylindrical | 0 | No web | Ambush | Burrow | 1 | 9 |
| *Arctosa lutetiana^7^* | Male | 9 | Cribellum | Cribellar | NA | No web | Ambush | Burrow | 1 | 9 |
| *Arctosa lutetiana^7^* | Male | 9 | ALS | MAP | 1 | No web | Ambush | Burrow | 1 | 9 |
| *Arctosa lutetiana^7^* | Male | 9 | ALS | Piriform | 12 | No web | Ambush | Burrow | 1 | 9 |
| *Arctosa lutetiana^7^* | Male | 9 | PMS | mAP | 1 | No web | Ambush | Burrow | 1 | 9 |
| *Arctosa lutetiana^7^* | Male | 9 | PMS | Aciniform | 6 | No web | Ambush | Burrow | 1 | 9 |
| *Arctosa lutetiana^7^* | Male | 9 | PMS | Cylindrical | 0 | No web | Ambush | Burrow | 1 | 9 |
| *Arctosa lutetiana^7^* | Male | 9 | PLS | Aciniform | 14 | No web | Ambush | Burrow | 1 | 9 |
| *Arctosa lutetiana^7^* | Male | 9 | PLS | Modified | NA | No web | Ambush | Burrow | 1 | 9 |
| *Arctosa lutetiana^7^* | Male | 9 | PLS | Flanking | NA | No web | Ambush | Burrow | 1 | 9 |
| *Arctosa lutetiana^7^* | Male | 9 | PLS | Cylindrical | 0 | No web | Ambush | Burrow | 1 | 9 |
| *Arctosa lutetiana^7^* | Female | 9 | Cribellum | Cribellar | NA | No web | Ambush | Burrow | 1 | 9 |
| *Arctosa lutetiana^7^* | Female | 9 | ALS | MAP | 2 | No web | Ambush | Burrow | 1 | 9 |
| *Arctosa lutetiana^7^* | Female | 9 | ALS | Piriform | 15 | No web | Ambush | Burrow | 1 | 9 |
| *Arctosa lutetiana^7^* | Female | 9 | PMS | mAP | 2 | No web | Ambush | Burrow | 1 | 9 |
| *Arctosa lutetiana^7^* | Female | 9 | PMS | Aciniform | 5 | No web | Ambush | Burrow | 1 | 9 |
| *Arctosa lutetiana^7^* | Female | 9 | PMS | Cylindrical | 6 | No web | Ambush | Burrow | 1 | 9 |
| *Arctosa lutetiana^7^* | Female | 9 | PLS | Aciniform | 20 | No web | Ambush | Burrow | 1 | 9 |
| *Arctosa lutetiana^7^* | Female | 9 | PLS | Modified | NA | No web | Ambush | Burrow | 1 | 9 |
| *Arctosa lutetiana^7^* | Female | 9 | PLS | Flanking | NA | No web | Ambush | Burrow | 1 | 9 |
| *Arctosa lutetiana^7^* | Female | 9 | PLS | Cylindrical | 1 | No web | Ambush | Burrow | 1 | 9 |
| *Arctosa alpigena lamperti^7^* | NA | 2 | Cribellum | Cribellar | NA | No web | Active | No | 1 | 10 |
| *Arctosa alpigena lamperti^7^* | NA | 2 | ALS | MAP | 2 | No web | Active | No | 1 | 10 |
| *Arctosa alpigena lamperti^7^* | NA | 2 | ALS | Piriform | 4 | No web | Active | No | 1 | 10 |
| *Arctosa alpigena lamperti^7^* | NA | 2 | PMS | mAP | 2 | No web | Active | No | 1 | 10 |
| *Arctosa alpigena lamperti^7^* | NA | 2 | PMS | Aciniform | 3 | No web | Active | No | 1 | 10 |
| *Arctosa alpigena lamperti^7^* | NA | 2 | PMS | Cylindrical | 0 | No web | Active | No | 1 | 10 |
| *Arctosa alpigena lamperti^7^* | NA | 2 | PLS | Aciniform | 10 | No web | Active | No | 1 | 10 |
| *Arctosa alpigena lamperti^7^* | NA | 2 | PLS | Modified | NA | No web | Active | No | 1 | 10 |
| *Arctosa alpigena lamperti^7^* | NA | 2 | PLS | Flanking | NA | No web | Active | No | 1 | 10 |
| *Arctosa alpigena lamperti^7^* | NA | 2 | PLS | Cylindrical | 0 | No web | Active | No | 1 | 10 |
| *Arctosa alpigena lamperti^7^* | NA | 3 | Cribellum | Cribellar | NA | No web | Active | No | 1 | 10 |
| *Arctosa alpigena lamperti^7^* | NA | 3 | ALS | MAP | 2 | No web | Active | No | 1 | 10 |
| *Arctosa alpigena lamperti^7^* | NA | 3 | ALS | Piriform | 5 | No web | Active | No | 1 | 10 |
| *Arctosa alpigena lamperti^7^* | NA | 3 | PMS | mAP | 2 | No web | Active | No | 1 | 10 |
| *Arctosa alpigena lamperti^7^* | NA | 3 | PMS | Aciniform | 4 | No web | Active | No | 1 | 10 |
| *Arctosa alpigena lamperti^7^* | NA | 3 | PMS | Cylindrical | 0 | No web | Active | No | 1 | 10 |
| *Arctosa alpigena lamperti^7^* | NA | 3 | PLS | Aciniform | 10 | No web | Active | No | 1 | 10 |
| *Arctosa alpigena lamperti^7^* | NA | 3 | PLS | Modified | NA | No web | Active | No | 1 | 10 |
| *Arctosa alpigena lamperti^7^* | NA | 3 | PLS | Flanking | NA | No web | Active | No | 1 | 10 |
| *Arctosa alpigena lamperti^7^* | NA | 3 | PLS | Cylindrical | 0 | No web | Active | No | 1 | 10 |
| *Arctosa alpigena lamperti^7^* | NA | 4 | Cribellum | Cribellar | NA | No web | Active | No | 1 | 10 |
| *Arctosa alpigena lamperti^7^* | NA | 4 | ALS | MAP | 2 | No web | Active | No | 1 | 10 |
| *Arctosa alpigena lamperti^7^* | NA | 4 | ALS | Piriform | 9 | No web | Active | No | 1 | 10 |
| *Arctosa alpigena lamperti^7^* | NA | 4 | PMS | mAP | 2 | No web | Active | No | 1 | 10 |
| *Arctosa alpigena lamperti^7^* | NA | 4 | PMS | Aciniform | 6 | No web | Active | No | 1 | 10 |
| *Arctosa alpigena lamperti^7^* | NA | 4 | PMS | Cylindrical | 0 | No web | Active | No | 1 | 10 |
| *Arctosa alpigena lamperti^7^* | NA | 4 | PLS | Aciniform | 13 | No web | Active | No | 1 | 10 |
| *Arctosa alpigena lamperti^7^* | NA | 4 | PLS | Modified | NA | No web | Active | No | 1 | 10 |
| *Arctosa alpigena lamperti^7^* | NA | 4 | PLS | Flanking | NA | No web | Active | No | 1 | 10 |
| *Arctosa alpigena lamperti^7^* | NA | 4 | PLS | Cylindrical | 0 | No web | Active | No | 1 | 10 |
| *Arctosa alpigena lamperti^7^* | NA | 5 | Cribellum | Cribellar | NA | No web | Active | No | 1 | 10 |
| *Arctosa alpigena lamperti^7^* | NA | 5 | ALS | MAP | 2 | No web | Active | No | 1 | 10 |
| *Arctosa alpigena lamperti^7^* | NA | 5 | ALS | Piriform | 11 | No web | Active | No | 1 | 10 |
| *Arctosa alpigena lamperti^7^* | NA | 5 | PMS | mAP | 2 | No web | Active | No | 1 | 10 |
| *Arctosa alpigena lamperti^7^* | NA | 5 | PMS | Aciniform | 8 | No web | Active | No | 1 | 10 |
| *Arctosa alpigena lamperti^7^* | NA | 5 | PMS | Cylindrical | 0 | No web | Active | No | 1 | 10 |
| *Arctosa alpigena lamperti^7^* | NA | 5 | PLS | Aciniform | 16 | No web | Active | No | 1 | 10 |
| *Arctosa alpigena lamperti^7^* | NA | 5 | PLS | Modified | NA | No web | Active | No | 1 | 10 |
| *Arctosa alpigena lamperti^7^* | NA | 5 | PLS | Flanking | NA | No web | Active | No | 1 | 10 |
| *Arctosa alpigena lamperti^7^* | NA | 5 | PLS | Cylindrical | 0 | No web | Active | No | 1 | 10 |
| *Arctosa alpigena lamperti^7^* | NA | 6 | Cribellum | Cribellar | NA | No web | Active | No | 1 | 10 |
| *Arctosa alpigena lamperti^7^* | NA | 6 | ALS | MAP | 2 | No web | Active | No | 1 | 10 |
| *Arctosa alpigena lamperti^7^* | NA | 6 | ALS | Piriform | 12 | No web | Active | No | 1 | 10 |
| *Arctosa alpigena lamperti^7^* | NA | 6 | PMS | mAP | 2 | No web | Active | No | 1 | 10 |
| *Arctosa alpigena lamperti^7^* | NA | 6 | PMS | Aciniform | 9 | No web | Active | No | 1 | 10 |
| *Arctosa alpigena lamperti^7^* | NA | 6 | PMS | Cylindrical | 0 | No web | Active | No | 1 | 10 |
| *Arctosa alpigena lamperti^7^* | NA | 6 | PLS | Aciniform | 18 | No web | Active | No | 1 | 10 |
| *Arctosa alpigena lamperti^7^* | NA | 6 | PLS | Modified | NA | No web | Active | No | 1 | 10 |
| *Arctosa alpigena lamperti^7^* | NA | 6 | PLS | Flanking | NA | No web | Active | No | 1 | 10 |
| *Arctosa alpigena lamperti^7^* | NA | 6 | PLS | Cylindrical | 0 | No web | Active | No | 1 | 10 |
| *Arctosa alpigena lamperti^7^* | NA | 7 | Cribellum | Cribellar | NA | No web | Active | No | 1 | 10 |
| *Arctosa alpigena lamperti^7^* | NA | 7 | ALS | MAP | 2 | No web | Active | No | 1 | 10 |
| *Arctosa alpigena lamperti^7^* | NA | 7 | ALS | Piriform | 14 | No web | Active | No | 1 | 10 |
| *Arctosa alpigena lamperti^7^* | NA | 7 | PMS | mAP | 2 | No web | Active | No | 1 | 10 |
| *Arctosa alpigena lamperti^7^* | NA | 7 | PMS | Aciniform | 13 | No web | Active | No | 1 | 10 |
| *Arctosa alpigena lamperti^7^* | NA | 7 | PMS | Cylindrical | 0 | No web | Active | No | 1 | 10 |
| *Arctosa alpigena lamperti^7^* | NA | 7 | PLS | Aciniform | 23 | No web | Active | No | 1 | 10 |
| *Arctosa alpigena lamperti^7^* | NA | 7 | PLS | Modified | NA | No web | Active | No | 1 | 10 |
| *Arctosa alpigena lamperti^7^* | NA | 7 | PLS | Flanking | NA | No web | Active | No | 1 | 10 |
| *Arctosa alpigena lamperti^7^* | NA | 7 | PLS | Cylindrical | 0 | No web | Active | No | 1 | 10 |
| *Arctosa alpigena lamperti^7^* | NA | 8 | Cribellum | Cribellar | NA | No web | Active | No | 1 | 10 |
| *Arctosa alpigena lamperti^7^* | NA | 8 | ALS | MAP | 2 | No web | Active | No | 1 | 10 |
| *Arctosa alpigena lamperti^7^* | NA | 8 | ALS | Piriform | 19 | No web | Active | No | 1 | 10 |
| *Arctosa alpigena lamperti^7^* | NA | 8 | PMS | mAP | 2 | No web | Active | No | 1 | 10 |
| *Arctosa alpigena lamperti^7^* | NA | 8 | PMS | Aciniform | 15 | No web | Active | No | 1 | 10 |
| *Arctosa alpigena lamperti^7^* | NA | 8 | PMS | Cylindrical | 0 | No web | Active | No | 1 | 10 |
| *Arctosa alpigena lamperti^7^* | NA | 8 | PLS | Aciniform | 31 | No web | Active | No | 1 | 10 |
| *Arctosa alpigena lamperti^7^* | NA | 8 | PLS | Modified | NA | No web | Active | No | 1 | 10 |
| *Arctosa alpigena lamperti^7^* | NA | 8 | PLS | Flanking | NA | No web | Active | No | 1 | 10 |
| *Arctosa alpigena lamperti^7^* | NA | 8 | PLS | Cylindrical | 0 | No web | Active | No | 1 | 10 |
| *Arctosa alpigena lamperti^7^* | Pen Male | 9 | Cribellum | Cribellar | NA | No web | Active | No | 1 | 10 |
| *Arctosa alpigena lamperti^7^* | Pen Male | 9 | ALS | MAP | 2 | No web | Active | No | 1 | 10 |
| *Arctosa alpigena lamperti^7^* | Pen Male | 9 | ALS | Piriform | 18 | No web | Active | No | 1 | 10 |
| *Arctosa alpigena lamperti^7^* | Pen Male | 9 | PMS | mAP | 2 | No web | Active | No | 1 | 10 |
| *Arctosa alpigena lamperti^7^* | Pen Male | 9 | PMS | Aciniform | 17 | No web | Active | No | 1 | 10 |
| *Arctosa alpigena lamperti^7^* | Pen Male | 9 | PMS | Cylindrical | 0 | No web | Active | No | 1 | 10 |
| *Arctosa alpigena lamperti^7^* | Pen Male | 9 | PLS | Aciniform | 27 | No web | Active | No | 1 | 10 |
| *Arctosa alpigena lamperti^7^* | Pen Male | 9 | PLS | Modified | NA | No web | Active | No | 1 | 10 |
| *Arctosa alpigena lamperti^7^* | Pen Male | 9 | PLS | Flanking | NA | No web | Active | No | 1 | 10 |
| *Arctosa alpigena lamperti^7^* | Pen Male | 9 | PLS | Cylindrical | 0 | No web | Active | No | 1 | 10 |
| *Arctosa alpigena lamperti^7^* | Pen Fem | 9 | Cribellum | Cribellar | NA | No web | Active | No | 1 | 10 |
| *Arctosa alpigena lamperti^7^* | Pen Fem | 9 | ALS | MAP | 2 | No web | Active | No | 1 | 10 |
| *Arctosa alpigena lamperti^7^* | Pen Fem | 9 | ALS | Piriform | 23 | No web | Active | No | 1 | 10 |
| *Arctosa alpigena lamperti^7^* | Pen Fem | 9 | PMS | mAP | 2 | No web | Active | No | 1 | 10 |
| *Arctosa alpigena lamperti^7^* | Pen Fem | 9 | PMS | Aciniform | 23 | No web | Active | No | 1 | 10 |
| *Arctosa alpigena lamperti^7^* | Pen Fem | 9 | PMS | Cylindrical | 0 | No web | Active | No | 1 | 10 |
| *Arctosa alpigena lamperti^7^* | Pen Fem | 9 | PLS | Aciniform | 42 | No web | Active | No | 1 | 10 |
| *Arctosa alpigena lamperti^7^* | Pen Fem | 9 | PLS | Modified | NA | No web | Active | No | 1 | 10 |
| *Arctosa alpigena lamperti^7^* | Pen Fem | 9 | PLS | Flanking | NA | No web | Active | No | 1 | 10 |
| *Arctosa alpigena lamperti^7^* | Pen Fem | 9 | PLS | Cylindrical | 0 | No web | Active | No | 1 | 10 |
| *Arctosa alpigena lamperti^7^* | Male | 10 | Cribellum | Cribellar | NA | No web | Active | No | 1 | 10 |
| *Arctosa alpigena lamperti^7^* | Male | 10 | ALS | MAP | 1 | No web | Active | No | 1 | 10 |
| *Arctosa alpigena lamperti^7^* | Male | 10 | ALS | Piriform | 19 | No web | Active | No | 1 | 10 |
| *Arctosa alpigena lamperti^7^* | Male | 10 | PMS | mAP | 1 | No web | Active | No | 1 | 10 |
| *Arctosa alpigena lamperti^7^* | Male | 10 | PMS | Aciniform | 21 | No web | Active | No | 1 | 10 |
| *Arctosa alpigena lamperti^7^* | Male | 10 | PMS | Cylindrical | 0 | No web | Active | No | 1 | 10 |
| *Arctosa alpigena lamperti^7^* | Male | 10 | PLS | Aciniform | 37 | No web | Active | No | 1 | 10 |
| *Arctosa alpigena lamperti^7^* | Male | 10 | PLS | Modified | NA | No web | Active | No | 1 | 10 |
| *Arctosa alpigena lamperti^7^* | Male | 10 | PLS | Flanking | NA | No web | Active | No | 1 | 10 |
| *Arctosa alpigena lamperti^7^* | Male | 10 | PLS | Cylindrical | 0 | No web | Active | No | 1 | 10 |
| *Arctosa alpigena lamperti^7^* | Female | 10 | Cribellum | Cribellar | NA | No web | Active | No | 1 | 10 |
| *Arctosa alpigena lamperti^7^* | Female | 10 | ALS | MAP | 2 | No web | Active | No | 1 | 10 |
| *Arctosa alpigena lamperti^7^* | Female | 10 | ALS | Piriform | 27 | No web | Active | No | 1 | 10 |
| *Arctosa alpigena lamperti^7^* | Female | 10 | PMS | mAP | 2 | No web | Active | No | 1 | 10 |
| *Arctosa alpigena lamperti^7^* | Female | 10 | PMS | Aciniform | 25 | No web | Active | No | 1 | 10 |
| *Arctosa alpigena lamperti^7^* | Female | 10 | PMS | Cylindrical | 1 | No web | Active | No | 1 | 10 |
| *Arctosa alpigena lamperti^7^* | Female | 10 | PLS | Aciniform | 58 | No web | Active | No | 1 | 10 |
| *Arctosa alpigena lamperti^7^* | Female | 10 | PLS | Modified | NA | No web | Active | No | 1 | 10 |
| *Arctosa alpigena lamperti^7^* | Female | 10 | PLS | Flanking | NA | No web | Active | No | 1 | 10 |
| *Arctosa alpigena lamperti^7^* | Female | 10 | PLS | Cylindrical | 1 | No web | Active | No | 1 | 10 |
| *Dolomedes tenebrosus^3^* | NA | 2 | Cribellum | Cribellar | NA | No web | Sit & Wait | No | 2 | 13 |
| *Dolomedes tenebrosus^3^* | NA | 2 | ALS | MAP | 2 | No web | Sit & Wait | No | 2 | 13 |
| *Dolomedes tenebrosus^3^* | NA | 2 | ALS | Piriform | 6 | No web | Sit & Wait | No | 2 | 13 |
| *Dolomedes tenebrosus^3^* | NA | 2 | PMS | mAP | 2 | No web | Sit & Wait | No | 2 | 13 |
| *Dolomedes tenebrosus^3^* | NA | 2 | PMS | Aciniform | 4 | No web | Sit & Wait | No | 2 | 13 |
| *Dolomedes tenebrosus^3^* | NA | 2 | PMS | Cylindrical | 0 | No web | Sit & Wait | No | 2 | 13 |
| *Dolomedes tenebrosus^3^* | NA | 2 | PLS | Aciniform | 4 | No web | Sit & Wait | No | 2 | 13 |
| *Dolomedes tenebrosus^3^* | NA | 2 | PLS | Modified | 0 | No web | Sit & Wait | No | 2 | 13 |
| *Dolomedes tenebrosus^3^* | NA | 2 | PLS | Flanking | 0 | No web | Sit & Wait | No | 2 | 13 |
| *Dolomedes tenebrosus^3^* | NA | 2 | PLS | Cylindrical | 0 | No web | Sit & Wait | No | 2 | 13 |
| *Dolomedes tenebrosus^3^* | NA | 3 | Cribellum | Cribellar | NA | No web | Sit & Wait | No | 2 | 13 |
| *Dolomedes tenebrosus^3^* | NA | 3 | ALS | MAP | 2 | No web | Sit & Wait | No | 2 | 13 |
| *Dolomedes tenebrosus^3^* | NA | 3 | ALS | Piriform | 9 | No web | Sit & Wait | No | 2 | 13 |
| *Dolomedes tenebrosus^3^* | NA | 3 | PMS | mAP | 2 | No web | Sit & Wait | No | 2 | 13 |
| *Dolomedes tenebrosus^3^* | NA | 3 | PMS | Aciniform | 4 | No web | Sit & Wait | No | 2 | 13 |
| *Dolomedes tenebrosus^3^* | NA | 3 | PMS | Cylindrical | 0 | No web | Sit & Wait | No | 2 | 13 |
| *Dolomedes tenebrosus^3^* | NA | 3 | PLS | Aciniform | 6 | No web | Sit & Wait | No | 2 | 13 |
| *Dolomedes tenebrosus^3^* | NA | 3 | PLS | Modified | 0 | No web | Sit & Wait | No | 2 | 13 |
| *Dolomedes tenebrosus^3^* | NA | 3 | PLS | Flanking | 0 | No web | Sit & Wait | No | 2 | 13 |
| *Dolomedes tenebrosus^3^* | NA | 3 | PLS | Cylindrical | 0 | No web | Sit & Wait | No | 2 | 13 |
| *Dolomedes tenebrosus^3^* | NA | 4 | Cribellum | Cribellar | NA | No web | Sit & Wait | No | 2 | 13 |
| *Dolomedes tenebrosus^3^* | NA | 4 | ALS | MAP | 2 | No web | Sit & Wait | No | 2 | 13 |
| *Dolomedes tenebrosus^3^* | NA | 4 | ALS | Piriform | 9 | No web | Sit & Wait | No | 2 | 13 |
| *Dolomedes tenebrosus^3^* | NA | 4 | PMS | mAP | 2 | No web | Sit & Wait | No | 2 | 13 |
| *Dolomedes tenebrosus^3^* | NA | 4 | PMS | Aciniform | 5 | No web | Sit & Wait | No | 2 | 13 |
| *Dolomedes tenebrosus^3^* | NA | 4 | PMS | Cylindrical | 0 | No web | Sit & Wait | No | 2 | 13 |
| *Dolomedes tenebrosus^3^* | NA | 4 | PLS | Aciniform | 6 | No web | Sit & Wait | No | 2 | 13 |
| *Dolomedes tenebrosus^3^* | NA | 4 | PLS | Modified | 0 | No web | Sit & Wait | No | 2 | 13 |
| *Dolomedes tenebrosus^3^* | NA | 4 | PLS | Flanking | 0 | No web | Sit & Wait | No | 2 | 13 |
| *Dolomedes tenebrosus^3^* | NA | 4 | PLS | Cylindrical | 0 | No web | Sit & Wait | No | 2 | 13 |
| *Dolomedes tenebrosus^3^* | NA | 5 | Cribellum | Cribellar | NA | No web | Sit & Wait | No | 2 | 13 |
| *Dolomedes tenebrosus^3^* | NA | 5 | ALS | MAP | 2 | No web | Sit & Wait | No | 2 | 13 |
| *Dolomedes tenebrosus^3^* | NA | 5 | ALS | Piriform | 14 | No web | Sit & Wait | No | 2 | 13 |
| *Dolomedes tenebrosus^3^* | NA | 5 | PMS | mAP | 2 | No web | Sit & Wait | No | 2 | 13 |
| *Dolomedes tenebrosus^3^* | NA | 5 | PMS | Aciniform | 5 | No web | Sit & Wait | No | 2 | 13 |
| *Dolomedes tenebrosus^3^* | NA | 5 | PMS | Cylindrical | 0 | No web | Sit & Wait | No | 2 | 13 |
| *Dolomedes tenebrosus^3^* | NA | 5 | PLS | Aciniform | 6 | No web | Sit & Wait | No | 2 | 13 |
| *Dolomedes tenebrosus^3^* | NA | 5 | PLS | Modified | 0 | No web | Sit & Wait | No | 2 | 13 |
| *Dolomedes tenebrosus^3^* | NA | 5 | PLS | Flanking | 0 | No web | Sit & Wait | No | 2 | 13 |
| *Dolomedes tenebrosus^3^* | NA | 5 | PLS | Cylindrical | 0 | No web | Sit & Wait | No | 2 | 13 |
| *Dolomedes tenebrosus^3^* | NA | 6 | Cribellum | Cribellar | NA | No web | Sit & Wait | No | 2 | 13 |
| *Dolomedes tenebrosus^3^* | NA | 6 | ALS | MAP | 2 | No web | Sit & Wait | No | 2 | 13 |
| *Dolomedes tenebrosus^3^* | NA | 6 | ALS | Piriform | 16 | No web | Sit & Wait | No | 2 | 13 |
| *Dolomedes tenebrosus^3^* | NA | 6 | PMS | mAP | 2 | No web | Sit & Wait | No | 2 | 13 |
| *Dolomedes tenebrosus^3^* | NA | 6 | PMS | Aciniform | 5 | No web | Sit & Wait | No | 2 | 13 |
| *Dolomedes tenebrosus^3^* | NA | 6 | PMS | Cylindrical | 0 | No web | Sit & Wait | No | 2 | 13 |
| *Dolomedes tenebrosus^3^* | NA | 6 | PLS | Aciniform | 9 | No web | Sit & Wait | No | 2 | 13 |
| *Dolomedes tenebrosus^3^* | NA | 6 | PLS | Modified | 0 | No web | Sit & Wait | No | 2 | 13 |
| *Dolomedes tenebrosus^3^* | NA | 6 | PLS | Flanking | 0 | No web | Sit & Wait | No | 2 | 13 |
| *Dolomedes tenebrosus^3^* | NA | 6 | PLS | Cylindrical | 0 | No web | Sit & Wait | No | 2 | 13 |
| *Dolomedes tenebrosus^3^* | NA | 7 | Cribellum | Cribellar | NA | No web | Sit & Wait | No | 2 | 13 |
| *Dolomedes tenebrosus^3^* | NA | 7 | ALS | MAP | 2 | No web | Sit & Wait | No | 2 | 13 |
| *Dolomedes tenebrosus^3^* | NA | 7 | ALS | Piriform | 18 | No web | Sit & Wait | No | 2 | 13 |
| *Dolomedes tenebrosus^3^* | NA | 7 | PMS | mAP | 2 | No web | Sit & Wait | No | 2 | 13 |
| *Dolomedes tenebrosus^3^* | NA | 7 | PMS | Aciniform | 6 | No web | Sit & Wait | No | 2 | 13 |
| *Dolomedes tenebrosus^3^* | NA | 7 | PMS | Cylindrical | 0 | No web | Sit & Wait | No | 2 | 13 |
| *Dolomedes tenebrosus^3^* | NA | 7 | PLS | Aciniform | 8 | No web | Sit & Wait | No | 2 | 13 |
| *Dolomedes tenebrosus^3^* | NA | 7 | PLS | Modified | 0 | No web | Sit & Wait | No | 2 | 13 |
| *Dolomedes tenebrosus^3^* | NA | 7 | PLS | Flanking | 0 | No web | Sit & Wait | No | 2 | 13 |
| *Dolomedes tenebrosus^3^* | NA | 7 | PLS | Cylindrical | 0 | No web | Sit & Wait | No | 2 | 13 |
| *Dolomedes tenebrosus^3^* | NA | 8 | Cribellum | Cribellar | NA | No web | Sit & Wait | No | 2 | 13 |
| *Dolomedes tenebrosus^3^* | NA | 8 | ALS | MAP | 2 | No web | Sit & Wait | No | 2 | 13 |
| *Dolomedes tenebrosus^3^* | NA | 8 | ALS | Piriform | 27 | No web | Sit & Wait | No | 2 | 13 |
| *Dolomedes tenebrosus^3^* | NA | 8 | PMS | mAP | 2 | No web | Sit & Wait | No | 2 | 13 |
| *Dolomedes tenebrosus^3^* | NA | 8 | PMS | Aciniform | 8 | No web | Sit & Wait | No | 2 | 13 |
| *Dolomedes tenebrosus^3^* | NA | 8 | PMS | Cylindrical | 0 | No web | Sit & Wait | No | 2 | 13 |
| *Dolomedes tenebrosus^3^* | NA | 8 | PLS | Aciniform | 9 | No web | Sit & Wait | No | 2 | 13 |
| *Dolomedes tenebrosus^3^* | NA | 8 | PLS | Modified | 0 | No web | Sit & Wait | No | 2 | 13 |
| *Dolomedes tenebrosus^3^* | NA | 8 | PLS | Flanking | 0 | No web | Sit & Wait | No | 2 | 13 |
| *Dolomedes tenebrosus^3^* | NA | 8 | PLS | Cylindrical | 0 | No web | Sit & Wait | No | 2 | 13 |
| *Dolomedes tenebrosus^3^* | NA | 9 | Cribellum | Cribellar | NA | No web | Sit & Wait | No | 2 | 13 |
| *Dolomedes tenebrosus^3^* | NA | 9 | ALS | MAP | 2 | No web | Sit & Wait | No | 2 | 13 |
| *Dolomedes tenebrosus^3^* | NA | 9 | ALS | Piriform | 57 | No web | Sit & Wait | No | 2 | 13 |
| *Dolomedes tenebrosus^3^* | NA | 9 | PMS | mAP | 2 | No web | Sit & Wait | No | 2 | 13 |
| *Dolomedes tenebrosus^3^* | NA | 9 | PMS | Aciniform | 8 | No web | Sit & Wait | No | 2 | 13 |
| *Dolomedes tenebrosus^3^* | NA | 9 | PMS | Cylindrical | 0 | No web | Sit & Wait | No | 2 | 13 |
| *Dolomedes tenebrosus^3^* | NA | 9 | PLS | Aciniform | 15 | No web | Sit & Wait | No | 2 | 13 |
| *Dolomedes tenebrosus^3^* | NA | 9 | PLS | Modified | 0 | No web | Sit & Wait | No | 2 | 13 |
| *Dolomedes tenebrosus^3^* | NA | 9 | PLS | Flanking | 0 | No web | Sit & Wait | No | 2 | 13 |
| *Dolomedes tenebrosus^3^* | NA | 9 | PLS | Cylindrical | 0 | No web | Sit & Wait | No | 2 | 13 |
| *Dolomedes tenebrosus^3^* | Ante Pen Fem | 10 | Cribellum | Cribellar | NA | No web | Sit & Wait | No | 2 | 13 |
| *Dolomedes tenebrosus^3^* | Ante Pen Fem | 10 | ALS | MAP | 2 | No web | Sit & Wait | No | 2 | 13 |
| *Dolomedes tenebrosus^3^* | Ante Pen Fem | 10 | ALS | Piriform | 52 | No web | Sit & Wait | No | 2 | 13 |
| *Dolomedes tenebrosus^3^* | Ante Pen Fem | 10 | PMS | mAP | 2 | No web | Sit & Wait | No | 2 | 13 |
| *Dolomedes tenebrosus^3^* | Ante Pen Fem | 10 | PMS | Aciniform | 8 | No web | Sit & Wait | No | 2 | 13 |
| *Dolomedes tenebrosus^3^* | Ante Pen Fem | 10 | PMS | Cylindrical | 0 | No web | Sit & Wait | No | 2 | 13 |
| *Dolomedes tenebrosus^3^* | Ante Pen Fem | 10 | PLS | Aciniform | 10 | No web | Sit & Wait | No | 2 | 13 |
| *Dolomedes tenebrosus^3^* | Ante Pen Fem | 10 | PLS | Modified | 0 | No web | Sit & Wait | No | 2 | 13 |
| *Dolomedes tenebrosus^3^* | Ante Pen Fem | 10 | PLS | Flanking | 0 | No web | Sit & Wait | No | 2 | 13 |
| *Dolomedes tenebrosus^3^* | Ante Pen Fem | 10 | PLS | Cylindrical | 0 | No web | Sit & Wait | No | 2 | 13 |
| *Dolomedes tenebrosus^3^* | Pen Male | 11 | Cribellum | Cribellar | NA | No web | Sit & Wait | No | 2 | 13 |
| *Dolomedes tenebrosus^3^* | Pen Male | 11 | ALS | MAP | 2 | No web | Sit & Wait | No | 2 | 13 |
| *Dolomedes tenebrosus^3^* | Pen Male | 11 | ALS | Piriform | 75 | No web | Sit & Wait | No | 2 | 13 |
| *Dolomedes tenebrosus^3^* | Pen Male | 11 | PMS | mAP | 2 | No web | Sit & Wait | No | 2 | 13 |
| *Dolomedes tenebrosus^3^* | Pen Male | 11 | PMS | Aciniform | 9 | No web | Sit & Wait | No | 2 | 13 |
| *Dolomedes tenebrosus^3^* | Pen Male | 11 | PMS | Cylindrical | 0 | No web | Sit & Wait | No | 2 | 13 |
| *Dolomedes tenebrosus^3^* | Pen Male | 11 | PLS | Aciniform | 14 | No web | Sit & Wait | No | 2 | 13 |
| *Dolomedes tenebrosus^3^* | Pen Male | 11 | PLS | Modified | 0 | No web | Sit & Wait | No | 2 | 13 |
| *Dolomedes tenebrosus^3^* | Pen Male | 11 | PLS | Flanking | 0 | No web | Sit & Wait | No | 2 | 13 |
| *Dolomedes tenebrosus^3^* | Pen Male | 11 | PLS | Cylindrical | 0 | No web | Sit & Wait | No | 2 | 13 |
| *Dolomedes tenebrosus^3^* | Pen Fem | 12 | Cribellum | Cribellar | NA | No web | Sit & Wait | No | 2 | 13 |
| *Dolomedes tenebrosus^3^* | Pen Fem | 12 | ALS | MAP | 2 | No web | Sit & Wait | No | 2 | 13 |
| *Dolomedes tenebrosus^3^* | Pen Fem | 12 | ALS | Piriform | 133 | No web | Sit & Wait | No | 2 | 13 |
| *Dolomedes tenebrosus^3^* | Pen Fem | 12 | PMS | mAP | 2 | No web | Sit & Wait | No | 2 | 13 |
| *Dolomedes tenebrosus^3^* | Pen Fem | 12 | PMS | Aciniform | 30 | No web | Sit & Wait | No | 2 | 13 |
| *Dolomedes tenebrosus^3^* | Pen Fem | 12 | PMS | Cylindrical | -1 | No web | Sit & Wait | No | 2 | 13 |
| *Dolomedes tenebrosus^3^* | Pen Fem | 12 | PLS | Aciniform | NA | No web | Sit & Wait | No | 2 | 13 |
| *Dolomedes tenebrosus^3^* | Pen Fem | 12 | PLS | Modified | NA | No web | Sit & Wait | No | 2 | 13 |
| *Dolomedes tenebrosus^3^* | Pen Fem | 12 | PLS | Flanking | NA | No web | Sit & Wait | No | 2 | 13 |
| *Dolomedes tenebrosus^3^* | Pen Fem | 12 | PLS | Cylindrical | NA | No web | Sit & Wait | No | 2 | 13 |
| *Dolomedes tenebrosus^3^* | Male | 12 | Cribellum | Cribellar | NA | No web | Sit & Wait | No | 2 | 13 |
| *Dolomedes tenebrosus^3^* | Male | 12 | ALS | MAP | 1 | No web | Sit & Wait | No | 2 | 13 |
| *Dolomedes tenebrosus^3^* | Male | 12 | ALS | Piriform | 67 | No web | Sit & Wait | No | 2 | 13 |
| *Dolomedes tenebrosus^3^* | Male | 12 | PMS | mAP | 1 | No web | Sit & Wait | No | 2 | 13 |
| *Dolomedes tenebrosus^3^* | Male | 12 | PMS | Aciniform | 7 | No web | Sit & Wait | No | 2 | 13 |
| *Dolomedes tenebrosus^3^* | Male | 12 | PMS | Cylindrical | 0 | No web | Sit & Wait | No | 2 | 13 |
| *Dolomedes tenebrosus^3^* | Male | 12 | PLS | Aciniform | 13 | No web | Sit & Wait | No | 2 | 13 |
| *Dolomedes tenebrosus^3^* | Male | 12 | PLS | Modified | 0 | No web | Sit & Wait | No | 2 | 13 |
| *Dolomedes tenebrosus^3^* | Male | 12 | PLS | Flanking | 0 | No web | Sit & Wait | No | 2 | 13 |
| *Dolomedes tenebrosus^3^* | Male | 12 | PLS | Cylindrical | 0 | No web | Sit & Wait | No | 2 | 13 |
| *Dolomedes tenebrosus^3^* | Female | 13 | Cribellum | Cribellar | NA | No web | Sit & Wait | No | 2 | 13 |
| *Dolomedes tenebrosus^3^* | Female | 13 | ALS | MAP | 2 | No web | Sit & Wait | No | 2 | 13 |
| *Dolomedes tenebrosus^3^* | Female | 13 | ALS | Piriform | 107 | No web | Sit & Wait | No | 2 | 13 |
| *Dolomedes tenebrosus^3^* | Female | 13 | PMS | mAP | 2 | No web | Sit & Wait | No | 2 | 13 |
| *Dolomedes tenebrosus^3^* | Female | 13 | PMS | Aciniform | 15 | No web | Sit & Wait | No | 2 | 13 |
| *Dolomedes tenebrosus^3^* | Female | 13 | PMS | Cylindrical | 32 | No web | Sit & Wait | No | 2 | 13 |
| *Dolomedes tenebrosus^3^* | Female | 13 | PLS | Aciniform | 24 | No web | Sit & Wait | No | 2 | 13 |
| *Dolomedes tenebrosus^3^* | Female | 13 | PLS | Modified | 1 | No web | Sit & Wait | No | 2 | 13 |
| *Dolomedes tenebrosus^3^* | Female | 13 | PLS | Flanking | 0 | No web | Sit & Wait | No | 2 | 13 |
| *Dolomedes tenebrosus^3^* | Female | 13 | PLS | Cylindrical | 28 | No web | Sit & Wait | No | 2 | 13 |
| *Tengella perfuga^3^* | NA | 2 | Cribellum | Cribellar | 0 | Web | Funnel web | Cribellate | 2.5 | 12 |
| *Tengella perfuga^3^* | NA | 2 | ALS | MAP | 2 | Web | Funnel web | Cribellate | 2.5 | 12 |
| *Tengella perfuga^3^* | NA | 2 | ALS | Piriform | 2 | Web | Funnel web | Cribellate | 2.5 | 12 |
| *Tengella perfuga^3^* | NA | 2 | PMS | mAP | 2 | Web | Funnel web | Cribellate | 2.5 | 12 |
| *Tengella perfuga^3^* | NA | 2 | PMS | Aciniform | 2 | Web | Funnel web | Cribellate | 2.5 | 12 |
| *Tengella perfuga^3^* | NA | 2 | PMS | Cylindrical | 0 | Web | Funnel web | Cribellate | 2.5 | 12 |
| *Tengella perfuga^3^* | NA | 2 | PLS | Aciniform | 3 | Web | Funnel web | Cribellate | 2.5 | 12 |
| *Tengella perfuga^3^* | NA | 2 | PLS | Modified | -1 | Web | Funnel web | Cribellate | 2.5 | 12 |
| *Tengella perfuga^3^* | NA | 2 | PLS | Flanking | -2 | Web | Funnel web | Cribellate | 2.5 | 12 |
| *Tengella perfuga^3^* | NA | 2 | PLS | Cylindrical | 0 | Web | Funnel web | Cribellate | 2.5 | 12 |
| *Tengella perfuga^3^* | NA | 3 | Cribellum | Cribellar | 172 | Web | Funnel web | Cribellate | 2.5 | 12 |
| *Tengella perfuga^3^* | NA | 3 | ALS | MAP | 2 | Web | Funnel web | Cribellate | 2.5 | 12 |
| *Tengella perfuga^3^* | NA | 3 | ALS | Piriform | 15 | Web | Funnel web | Cribellate | 2.5 | 12 |
| *Tengella perfuga^3^* | NA | 3 | PMS | mAP | 2 | Web | Funnel web | Cribellate | 2.5 | 12 |
| *Tengella perfuga^3^* | NA | 3 | PMS | Aciniform | 12 | Web | Funnel web | Cribellate | 2.5 | 12 |
| *Tengella perfuga^3^* | NA | 3 | PMS | Cylindrical | 0 | Web | Funnel web | Cribellate | 2.5 | 12 |
| *Tengella perfuga^3^* | NA | 3 | PLS | Aciniform | 17 | Web | Funnel web | Cribellate | 2.5 | 12 |
| *Tengella perfuga^3^* | NA | 3 | PLS | Modified | 1 | Web | Funnel web | Cribellate | 2.5 | 12 |
| *Tengella perfuga^3^* | NA | 3 | PLS | Flanking | 2 | Web | Funnel web | Cribellate | 2.5 | 12 |
| *Tengella perfuga^3^* | NA | 3 | PLS | Cylindrical | 0 | Web | Funnel web | Cribellate | 2.5 | 12 |
| *Tengella perfuga^3^* | NA | 4 | Cribellum | Cribellar | 240 | Web | Funnel web | Cribellate | 2.5 | 12 |
| *Tengella perfuga^3^* | NA | 4 | ALS | MAP | 2 | Web | Funnel web | Cribellate | 2.5 | 12 |
| *Tengella perfuga^3^* | NA | 4 | ALS | Piriform | 23 | Web | Funnel web | Cribellate | 2.5 | 12 |
| *Tengella perfuga^3^* | NA | 4 | PMS | mAP | 2 | Web | Funnel web | Cribellate | 2.5 | 12 |
| *Tengella perfuga^3^* | NA | 4 | PMS | Aciniform | 14 | Web | Funnel web | Cribellate | 2.5 | 12 |
| *Tengella perfuga^3^* | NA | 4 | PMS | Cylindrical | 0 | Web | Funnel web | Cribellate | 2.5 | 12 |
| *Tengella perfuga^3^* | NA | 4 | PLS | Aciniform | 18 | Web | Funnel web | Cribellate | 2.5 | 12 |
| *Tengella perfuga^3^* | NA | 4 | PLS | Modified | 1 | Web | Funnel web | Cribellate | 2.5 | 12 |
| *Tengella perfuga^3^* | NA | 4 | PLS | Flanking | 2 | Web | Funnel web | Cribellate | 2.5 | 12 |
| *Tengella perfuga^3^* | NA | 4 | PLS | Cylindrical | 0 | Web | Funnel web | Cribellate | 2.5 | 12 |
| *Tengella perfuga^3^* | NA | 5 | Cribellum | Cribellar | 404 | Web | Funnel web | Cribellate | 2.5 | 12 |
| *Tengella perfuga^3^* | NA | 5 | ALS | MAP | 2 | Web | Funnel web | Cribellate | 2.5 | 12 |
| *Tengella perfuga^3^* | NA | 5 | ALS | Piriform | 25 | Web | Funnel web | Cribellate | 2.5 | 12 |
| *Tengella perfuga^3^* | NA | 5 | PMS | mAP | 2 | Web | Funnel web | Cribellate | 2.5 | 12 |
| *Tengella perfuga^3^* | NA | 5 | PMS | Aciniform | 18 | Web | Funnel web | Cribellate | 2.5 | 12 |
| *Tengella perfuga^3^* | NA | 5 | PMS | Cylindrical | 0 | Web | Funnel web | Cribellate | 2.5 | 12 |
| *Tengella perfuga^3^* | NA | 5 | PLS | Aciniform | 22 | Web | Funnel web | Cribellate | 2.5 | 12 |
| *Tengella perfuga^3^* | NA | 5 | PLS | Modified | 1 | Web | Funnel web | Cribellate | 2.5 | 12 |
| *Tengella perfuga^3^* | NA | 5 | PLS | Flanking | 2 | Web | Funnel web | Cribellate | 2.5 | 12 |
| *Tengella perfuga^3^* | NA | 5 | PLS | Cylindrical | 0 | Web | Funnel web | Cribellate | 2.5 | 12 |
| *Tengella perfuga^3^* | NA | 6 | Cribellum | Cribellar | 656 | Web | Funnel web | Cribellate | 2.5 | 12 |
| *Tengella perfuga^3^* | NA | 6 | ALS | MAP | 2 | Web | Funnel web | Cribellate | 2.5 | 12 |
| *Tengella perfuga^3^* | NA | 6 | ALS | Piriform | 31 | Web | Funnel web | Cribellate | 2.5 | 12 |
| *Tengella perfuga^3^* | NA | 6 | PMS | mAP | 2 | Web | Funnel web | Cribellate | 2.5 | 12 |
| *Tengella perfuga^3^* | NA | 6 | PMS | Aciniform | 24 | Web | Funnel web | Cribellate | 2.5 | 12 |
| *Tengella perfuga^3^* | NA | 6 | PMS | Cylindrical | 0 | Web | Funnel web | Cribellate | 2.5 | 12 |
| *Tengella perfuga^3^* | NA | 6 | PLS | Aciniform | 26 | Web | Funnel web | Cribellate | 2.5 | 12 |
| *Tengella perfuga^3^* | NA | 6 | PLS | Modified | 1 | Web | Funnel web | Cribellate | 2.5 | 12 |
| *Tengella perfuga^3^* | NA | 6 | PLS | Flanking | 2 | Web | Funnel web | Cribellate | 2.5 | 12 |
| *Tengella perfuga^3^* | NA | 6 | PLS | Cylindrical | -1 | Web | Funnel web | Cribellate | 2.5 | 12 |
| *Tengella perfuga^3^* | NA | 7 | Cribellum | Cribellar | 864 | Web | Funnel web | Cribellate | 2.5 | 12 |
| *Tengella perfuga^3^* | NA | 7 | ALS | MAP | 2 | Web | Funnel web | Cribellate | 2.5 | 12 |
| *Tengella perfuga^3^* | NA | 7 | ALS | Piriform | 41 | Web | Funnel web | Cribellate | 2.5 | 12 |
| *Tengella perfuga^3^* | NA | 7 | ALS | MAP | NA | Web | Funnel web | Cribellate | 2.5 | 12 |
| *Tengella perfuga^3^* | NA | 7 | ALS | Piriform | NA | Web | Funnel web | Cribellate | 2.5 | 12 |
| *Tengella perfuga^3^* | NA | 7 | PMS | mAP | 2 | Web | Funnel web | Cribellate | 2.5 | 12 |
| *Tengella perfuga^3^* | NA | 7 | PMS | Aciniform | 26 | Web | Funnel web | Cribellate | 2.5 | 12 |
| *Tengella perfuga^3^* | NA | 7 | PMS | Cylindrical | 0 | Web | Funnel web | Cribellate | 2.5 | 12 |
| *Tengella perfuga^3^* | NA | 7 | PMS | mAP | 2 | Web | Funnel web | Cribellate | 2.5 | 12 |
| *Tengella perfuga^3^* | NA | 7 | PMS | Aciniform | 30 | Web | Funnel web | Cribellate | 2.5 | 12 |
| *Tengella perfuga^3^* | NA | 7 | PMS | Cylindrical | 0 | Web | Funnel web | Cribellate | 2.5 | 12 |
| *Tengella perfuga^3^* | NA | 7 | PLS | Aciniform | 30 | Web | Funnel web | Cribellate | 2.5 | 12 |
| *Tengella perfuga^3^* | NA | 7 | PLS | Modified | 1 | Web | Funnel web | Cribellate | 2.5 | 12 |
| *Tengella perfuga^3^* | NA | 7 | PLS | Flanking | 2 | Web | Funnel web | Cribellate | 2.5 | 12 |
| *Tengella perfuga^3^* | NA | 7 | PLS | Cylindrical | -3 | Web | Funnel web | Cribellate | 2.5 | 12 |
| *Tengella perfuga^3^* | NA | 7 | PLS | Aciniform | 32 | Web | Funnel web | Cribellate | 2.5 | 12 |
| *Tengella perfuga^3^* | NA | 7 | PLS | Modified | 1 | Web | Funnel web | Cribellate | 2.5 | 12 |
| *Tengella perfuga^3^* | NA | 7 | PLS | Flanking | 2 | Web | Funnel web | Cribellate | 2.5 | 12 |
| *Tengella perfuga^3^* | NA | 7 | PLS | Cylindrical | -3 | Web | Funnel web | Cribellate | 2.5 | 12 |
| *Tengella perfuga^3^* | NA | 7 | Cribellum | Cribellar | 1241 | Web | Funnel web | Cribellate | 2.5 | 12 |
| *Tengella perfuga^3^* | NA | 7 | ALS | MAP | 2 | Web | Funnel web | Cribellate | 2.5 | 12 |
| *Tengella perfuga^3^* | NA | 7 | ALS | Piriform | 48 | Web | Funnel web | Cribellate | 2.5 | 12 |
| *Tengella perfuga^3^* | NA | 7 | PMS | mAP | 2 | Web | Funnel web | Cribellate | 2.5 | 12 |
| *Tengella perfuga^3^* | NA | 7 | PMS | Aciniform | 34 | Web | Funnel web | Cribellate | 2.5 | 12 |
| *Tengella perfuga^3^* | NA | 7 | PMS | Cylindrical | 0 | Web | Funnel web | Cribellate | 2.5 | 12 |
| *Tengella perfuga^3^* | NA | 7 | PLS | Aciniform | 33 | Web | Funnel web | Cribellate | 2.5 | 12 |
| *Tengella perfuga^3^* | NA | 7 | PLS | Modified | 1 | Web | Funnel web | Cribellate | 2.5 | 12 |
| *Tengella perfuga^3^* | NA | 7 | PLS | Flanking | 2 | Web | Funnel web | Cribellate | 2.5 | 12 |
| *Tengella perfuga^3^* | NA | 7 | PLS | Cylindrical | -3 | Web | Funnel web | Cribellate | 2.5 | 12 |
| *Tengella perfuga^3^* | NA | 8 | Cribellum | Cribellar | 2613 | Web | Funnel web | Cribellate | 2.5 | 12 |
| *Tengella perfuga^3^* | NA | 8 | ALS | MAP | 2 | Web | Funnel web | Cribellate | 2.5 | 12 |
| *Tengella perfuga^3^* | NA | 8 | ALS | Piriform | 68 | Web | Funnel web | Cribellate | 2.5 | 12 |
| *Tengella perfuga^3^* | NA | 8 | PMS | mAP | 2 | Web | Funnel web | Cribellate | 2.5 | 12 |
| *Tengella perfuga^3^* | NA | 8 | PMS | Aciniform | 59 | Web | Funnel web | Cribellate | 2.5 | 12 |
| *Tengella perfuga^3^* | NA | 8 | PMS | Cylindrical | -1 | Web | Funnel web | Cribellate | 2.5 | 12 |
| *Tengella perfuga^3^* | NA | 8 | PLS | Aciniform | 43 | Web | Funnel web | Cribellate | 2.5 | 12 |
| *Tengella perfuga^3^* | NA | 8 | PLS | Modified | 1 | Web | Funnel web | Cribellate | 2.5 | 12 |
| *Tengella perfuga^3^* | NA | 8 | PLS | Flanking | 2 | Web | Funnel web | Cribellate | 2.5 | 12 |
| *Tengella perfuga^3^* | NA | 8 | PLS | Cylindrical | -4 | Web | Funnel web | Cribellate | 2.5 | 12 |
| *Tengella perfuga^3^* | Pen Fem | 8 | Cribellum | Cribellar | NA | Web | Funnel web | Cribellate | 2.5 | 12 |
| *Tengella perfuga^3^* | Pen Fem | 8 | ALS | MAP | 2 | Web | Funnel web | Cribellate | 2.5 | 12 |
| *Tengella perfuga^3^* | Pen Fem | 8 | ALS | Piriform | 78 | Web | Funnel web | Cribellate | 2.5 | 12 |
| *Tengella perfuga^3^* | Pen Fem | 8 | PMS | mAP | 2 | Web | Funnel web | Cribellate | 2.5 | 12 |
| *Tengella perfuga^3^* | Pen Fem | 8 | PMS | Aciniform | 65 | Web | Funnel web | Cribellate | 2.5 | 12 |
| *Tengella perfuga^3^* | Pen Fem | 8 | PMS | Cylindrical | -1 | Web | Funnel web | Cribellate | 2.5 | 12 |
| *Tengella perfuga^3^* | Pen Fem | 8 | PLS | Aciniform | 40 | Web | Funnel web | Cribellate | 2.5 | 12 |
| *Tengella perfuga^3^* | Pen Fem | 8 | PLS | Modified | 1 | Web | Funnel web | Cribellate | 2.5 | 12 |
| *Tengella perfuga^3^* | Pen Fem | 8 | PLS | Flanking | 2 | Web | Funnel web | Cribellate | 2.5 | 12 |
| *Tengella perfuga^3^* | Pen Fem | 8 | PLS | Cylindrical | -3 | Web | Funnel web | Cribellate | 2.5 | 12 |
| *Tengella perfuga^3^* | Pen Male | 8 | Cribellum | Cribellar | NA | Web | Funnel web | Cribellate | 2.5 | 12 |
| *Tengella perfuga^3^* | Pen Male | 8 | ALS | MAP | 2 | Web | Funnel web | Cribellate | 2.5 | 12 |
| *Tengella perfuga^3^* | Pen Male | 8 | ALS | Piriform | 83 | Web | Funnel web | Cribellate | 2.5 | 12 |
| *Tengella perfuga^3^* | Pen Male | 8 | PMS | mAP | 2 | Web | Funnel web | Cribellate | 2.5 | 12 |
| *Tengella perfuga^3^* | Pen Male | 8 | PMS | Aciniform | 69 | Web | Funnel web | Cribellate | 2.5 | 12 |
| *Tengella perfuga^3^* | Pen Male | 8 | PMS | Cylindrical | 0 | Web | Funnel web | Cribellate | 2.5 | 12 |
| *Tengella perfuga^3^* | Pen Male | 8 | PLS | Aciniform | 68 | Web | Funnel web | Cribellate | 2.5 | 12 |
| *Tengella perfuga^3^* | Pen Male | 8 | PLS | Modified | 1 | Web | Funnel web | Cribellate | 2.5 | 12 |
| *Tengella perfuga^3^* | Pen Male | 8 | PLS | Flanking | 2 | Web | Funnel web | Cribellate | 2.5 | 12 |
| *Tengella perfuga^3^* | Pen Male | 8 | PLS | Cylindrical | -3 | Web | Funnel web | Cribellate | 2.5 | 12 |
| *Tengella perfuga^3^* | NA | 9 | Cribellum | Cribellar | NA | Web | Funnel web | Cribellate | 2.5 | 12 |
| *Tengella perfuga^3^* | NA | 9 | ALS | MAP | 2 | Web | Funnel web | Cribellate | 2.5 | 12 |
| *Tengella perfuga^3^* | NA | 9 | ALS | Piriform | 73 | Web | Funnel web | Cribellate | 2.5 | 12 |
| *Tengella perfuga^3^* | NA | 9 | PMS | mAP | 2 | Web | Funnel web | Cribellate | 2.5 | 12 |
| *Tengella perfuga^3^* | NA | 9 | PMS | Aciniform | NA | Web | Funnel web | Cribellate | 2.5 | 12 |
| *Tengella perfuga^3^* | NA | 9 | PMS | Cylindrical | -1 | Web | Funnel web | Cribellate | 2.5 | 12 |
| *Tengella perfuga^3^* | NA | 9 | PLS | Aciniform | NA | Web | Funnel web | Cribellate | 2.5 | 12 |
| *Tengella perfuga^3^* | NA | 9 | PLS | Modified | 1 | Web | Funnel web | Cribellate | 2.5 | 12 |
| *Tengella perfuga^3^* | NA | 9 | PLS | Flanking | 2 | Web | Funnel web | Cribellate | 2.5 | 12 |
| *Tengella perfuga^3^* | NA | 9 | PLS | Cylindrical | -2 | Web | Funnel web | Cribellate | 2.5 | 12 |
| *Tengella perfuga^3^* | Male | 9 | Cribellum | Cribellar | 0 | Web | Funnel web | Cribellate | 2.5 | 12 |
| *Tengella perfuga^3^* | Male | 9 | ALS | MAP | 1 | Web | Funnel web | Cribellate | 2.5 | 12 |
| *Tengella perfuga^3^* | Male | 9 | ALS | Piriform | 84 | Web | Funnel web | Cribellate | 2.5 | 12 |
| *Tengella perfuga^3^* | Male | 9 | PMS | mAP | 2 | Web | Funnel web | Cribellate | 2.5 | 12 |
| *Tengella perfuga^3^* | Male | 9 | PMS | Aciniform | 83 | Web | Funnel web | Cribellate | 2.5 | 12 |
| *Tengella perfuga^3^* | Male | 9 | PMS | Cylindrical | 0 | Web | Funnel web | Cribellate | 2.5 | 12 |
| *Tengella perfuga^3^* | Male | 9 | PLS | Aciniform | 68 | Web | Funnel web | Cribellate | 2.5 | 12 |
| *Tengella perfuga^3^* | Male | 9 | PLS | Modified | 0 | Web | Funnel web | Cribellate | 2.5 | 12 |
| *Tengella perfuga^3^* | Male | 9 | PLS | Flanking | 0 | Web | Funnel web | Cribellate | 2.5 | 12 |
| *Tengella perfuga^3^* | Male | 9 | PLS | Cylindrical | 0 | Web | Funnel web | Cribellate | 2.5 | 12 |
| *Tengella perfuga^3^* | NA | 10 | Cribellum | Cribellar | 3600 | Web | Funnel web | Cribellate | 2.5 | 12 |
| *Tengella perfuga^3^* | NA | 10 | ALS | MAP | 2 | Web | Funnel web | Cribellate | 2.5 | 12 |
| *Tengella perfuga^3^* | NA | 10 | ALS | Piriform | 72 | Web | Funnel web | Cribellate | 2.5 | 12 |
| *Tengella perfuga^3^* | NA | 10 | PMS | mAP | 2 | Web | Funnel web | Cribellate | 2.5 | 12 |
| *Tengella perfuga^3^* | NA | 10 | PMS | Aciniform | 59 | Web | Funnel web | Cribellate | 2.5 | 12 |
| *Tengella perfuga^3^* | NA | 10 | PMS | Cylindrical | 0 | Web | Funnel web | Cribellate | 2.5 | 12 |
| *Tengella perfuga^3^* | NA | 10 | PLS | Aciniform | 42 | Web | Funnel web | Cribellate | 2.5 | 12 |
| *Tengella perfuga^3^* | NA | 10 | PLS | Modified | 1 | Web | Funnel web | Cribellate | 2.5 | 12 |
| *Tengella perfuga^3^* | NA | 10 | PLS | Flanking | 2 | Web | Funnel web | Cribellate | 2.5 | 12 |
| *Tengella perfuga^3^* | NA | 10 | PLS | Cylindrical | -5 | Web | Funnel web | Cribellate | 2.5 | 12 |
| *Tengella perfuga^3^* | Male | 10 | Cribellum | Cribellar | 0 | Web | Funnel web | Cribellate | 2.5 | 12 |
| *Tengella perfuga^3^* | Male | 10 | ALS | MAP | 1 | Web | Funnel web | Cribellate | 2.5 | 12 |
| *Tengella perfuga^3^* | Male | 10 | ALS | Piriform | 120 | Web | Funnel web | Cribellate | 2.5 | 12 |
| *Tengella perfuga^3^* | Male | 10 | PMS | mAP | 2 | Web | Funnel web | Cribellate | 2.5 | 12 |
| *Tengella perfuga^3^* | Male | 10 | PMS | Aciniform | 94 | Web | Funnel web | Cribellate | 2.5 | 12 |
| *Tengella perfuga^3^* | Male | 10 | PMS | Cylindrical | 0 | Web | Funnel web | Cribellate | 2.5 | 12 |
| *Tengella perfuga^3^* | Male | 10 | PLS | Aciniform | 62 | Web | Funnel web | Cribellate | 2.5 | 12 |
| *Tengella perfuga^3^* | Male | 10 | PLS | Modified | 0 | Web | Funnel web | Cribellate | 2.5 | 12 |
| *Tengella perfuga^3^* | Male | 10 | PLS | Flanking | 0 | Web | Funnel web | Cribellate | 2.5 | 12 |
| *Tengella perfuga^3^* | Male | 10 | PLS | Cylindrical | 0 | Web | Funnel web | Cribellate | 2.5 | 12 |
| *Tengella perfuga^3^* | Pen Fem | 11 | Cribellum | Cribellar | NA | Web | Funnel web | Cribellate | 2.5 | 12 |
| *Tengella perfuga^3^* | Pen Fem | 11 | ALS | MAP | NA | Web | Funnel web | Cribellate | 2.5 | 12 |
| *Tengella perfuga^3^* | Pen Fem | 11 | ALS | Piriform | NA | Web | Funnel web | Cribellate | 2.5 | 12 |
| *Tengella perfuga^3^* | Pen Fem | 11 | ALS | MAP | NA | Web | Funnel web | Cribellate | 2.5 | 12 |
| *Tengella perfuga^3^* | Pen Fem | 11 | ALS | Piriform | NA | Web | Funnel web | Cribellate | 2.5 | 12 |
| *Tengella perfuga^3^* | Pen Fem | 11 | PMS | mAP | NA | Web | Funnel web | Cribellate | 2.5 | 12 |
| *Tengella perfuga^3^* | Pen Fem | 11 | PMS | Aciniform | NA | Web | Funnel web | Cribellate | 2.5 | 12 |
| *Tengella perfuga^3^* | Pen Fem | 11 | PMS | Cylindrical | NA | Web | Funnel web | Cribellate | 2.5 | 12 |
| *Tengella perfuga^3^* | Pen Fem | 11 | PMS | mAP | NA | Web | Funnel web | Cribellate | 2.5 | 12 |
| *Tengella perfuga^3^* | Pen Fem | 11 | PMS | Aciniform | NA | Web | Funnel web | Cribellate | 2.5 | 12 |
| *Tengella perfuga^3^* | Pen Fem | 11 | PMS | Cylindrical | NA | Web | Funnel web | Cribellate | 2.5 | 12 |
| *Tengella perfuga^3^* | Pen Fem | 11 | PLS | Aciniform | NA | Web | Funnel web | Cribellate | 2.5 | 12 |
| *Tengella perfuga^3^* | Pen Fem | 11 | PLS | Modified | NA | Web | Funnel web | Cribellate | 2.5 | 12 |
| *Tengella perfuga^3^* | Pen Fem | 11 | PLS | Flanking | NA | Web | Funnel web | Cribellate | 2.5 | 12 |
| *Tengella perfuga^3^* | Pen Fem | 11 | PLS | Cylindrical | NA | Web | Funnel web | Cribellate | 2.5 | 12 |
| *Tengella perfuga^3^* | Pen Fem | 11 | PLS | Aciniform | NA | Web | Funnel web | Cribellate | 2.5 | 12 |
| *Tengella perfuga^3^* | Pen Fem | 11 | PLS | Modified | NA | Web | Funnel web | Cribellate | 2.5 | 12 |
| *Tengella perfuga^3^* | Pen Fem | 11 | PLS | Flanking | NA | Web | Funnel web | Cribellate | 2.5 | 12 |
| *Tengella perfuga^3^* | Pen Fem | 11 | PLS | Cylindrical | NA | Web | Funnel web | Cribellate | 2.5 | 12 |
| *Tengella perfuga^3^* | Pen Male | 11 | Cribellum | Cribellar | 7200 | Web | Funnel web | Cribellate | 2.5 | 12 |
| *Tengella perfuga^3^* | Pen Male | 11 | ALS | MAP | 2 | Web | Funnel web | Cribellate | 2.5 | 12 |
| *Tengella perfuga^3^* | Pen Male | 11 | ALS | Piriform | 89 | Web | Funnel web | Cribellate | 2.5 | 12 |
| *Tengella perfuga^3^* | Pen Male | 11 | PMS | mAP | 2 | Web | Funnel web | Cribellate | 2.5 | 12 |
| *Tengella perfuga^3^* | Pen Male | 11 | PMS | Aciniform | 65 | Web | Funnel web | Cribellate | 2.5 | 12 |
| *Tengella perfuga^3^* | Pen Male | 11 | PMS | Cylindrical | 0 | Web | Funnel web | Cribellate | 2.5 | 12 |
| *Tengella perfuga^3^* | Pen Male | 11 | PLS | Aciniform | 62 | Web | Funnel web | Cribellate | 2.5 | 12 |
| *Tengella perfuga^3^* | Pen Male | 11 | PLS | Modified | 1 | Web | Funnel web | Cribellate | 2.5 | 12 |
| *Tengella perfuga^3^* | Pen Male | 11 | PLS | Flanking | 2 | Web | Funnel web | Cribellate | 2.5 | 12 |
| *Tengella perfuga^3^* | Pen Male | 11 | PLS | Cylindrical | 0 | Web | Funnel web | Cribellate | 2.5 | 12 |
| *Tengella perfuga^3^* | Female | 11 | Cribellum | Cribellar | 9285 | Web | Funnel web | Cribellate | 2.5 | 12 |
| *Tengella perfuga^3^* | Female | 11 | ALS | MAP | 2 | Web | Funnel web | Cribellate | 2.5 | 12 |
| *Tengella perfuga^3^* | Female | 11 | ALS | Piriform | 110 | Web | Funnel web | Cribellate | 2.5 | 12 |
| *Tengella perfuga^3^* | Female | 11 | PMS | mAP | 2 | Web | Funnel web | Cribellate | 2.5 | 12 |
| *Tengella perfuga^3^* | Female | 11 | PMS | Aciniform | 91 | Web | Funnel web | Cribellate | 2.5 | 12 |
| *Tengella perfuga^3^* | Female | 11 | PMS | Cylindrical | 2 | Web | Funnel web | Cribellate | 2.5 | 12 |
| *Tengella perfuga^3^* | Female | 11 | PLS | Aciniform | 80 | Web | Funnel web | Cribellate | 2.5 | 12 |
| *Tengella perfuga^3^* | Female | 11 | PLS | Modified | 1 | Web | Funnel web | Cribellate | 2.5 | 12 |
| *Tengella perfuga^3^* | Female | 11 | PLS | Flanking | 2 | Web | Funnel web | Cribellate | 2.5 | 12 |
| *Tengella perfuga^3^* | Female | 11 | PLS | Cylindrical | 3 | Web | Funnel web | Cribellate | 2.5 | 12 |
| *Tengella perfuga^3^* | Female | 12 | Cribellum | Cribellar | 12010 | Web | Funnel web | Cribellate | 2.5 | 12 |
| *Tengella perfuga^3^* | Female | 12 | ALS | MAP | 2 | Web | Funnel web | Cribellate | 2.5 | 12 |
| *Tengella perfuga^3^* | Female | 12 | ALS | Piriform | 154 | Web | Funnel web | Cribellate | 2.5 | 12 |
| *Tengella perfuga^3^* | Female | 12 | PMS | mAP | 2 | Web | Funnel web | Cribellate | 2.5 | 12 |
| *Tengella perfuga^3^* | Female | 12 | PMS | Aciniform | 91 | Web | Funnel web | Cribellate | 2.5 | 12 |
| *Tengella perfuga^3^* | Female | 12 | PMS | Cylindrical | 2 | Web | Funnel web | Cribellate | 2.5 | 12 |
| *Tengella perfuga^3^* | Female | 12 | PLS | Aciniform | 87 | Web | Funnel web | Cribellate | 2.5 | 12 |
| *Tengella perfuga^3^* | Female | 12 | PLS | Modified | 1 | Web | Funnel web | Cribellate | 2.5 | 12 |
| *Tengella perfuga^3^* | Female | 12 | PLS | Flanking | 2 | Web | Funnel web | Cribellate | 2.5 | 12 |
| *Tengella perfuga^3^* | Female | 12 | PLS | Cylindrical | 3 | Web | Funnel web | Cribellate | 2.5 | 12 |
| *Tengella perfuga^3^* | Male | 12 | Cribellum | Cribellar | 0 | Web | Funnel web | Cribellate | 2.5 | 12 |
| *Tengella perfuga^3^* | Male | 12 | ALS | MAP | 1 | Web | Funnel web | Cribellate | 2.5 | 12 |
| *Tengella perfuga^3^* | Male | 12 | ALS | Piriform | 79 | Web | Funnel web | Cribellate | 2.5 | 12 |
| *Tengella perfuga^3^* | Male | 12 | PMS | mAP | 2 | Web | Funnel web | Cribellate | 2.5 | 12 |
| *Tengella perfuga^3^* | Male | 12 | PMS | Aciniform | 103 | Web | Funnel web | Cribellate | 2.5 | 12 |
| *Tengella perfuga^3^* | Male | 12 | PMS | Cylindrical | 0 | Web | Funnel web | Cribellate | 2.5 | 12 |
| *Tengella perfuga^3^* | Male | 12 | PLS | Aciniform | 63 | Web | Funnel web | Cribellate | 2.5 | 12 |
| *Tengella perfuga^3^* | Male | 12 | PLS | Modified | 0 | Web | Funnel web | Cribellate | 2.5 | 12 |
| *Tengella perfuga^3^* | Male | 12 | PLS | Flanking | 0 | Web | Funnel web | Cribellate | 2.5 | 12 |
| *Tengella perfuga^3^* | Male | 12 | PLS | Cylindrical | 0 | Web | Funnel web | Cribellate | 2.5 | 12 |
| *Argyroneta aquatica^1^* | NA | 2 | Cribellum | Cribellar | NA | Web | Sheet web | Acinform | 1 | 6 |
| *Argyroneta aquatica^1^* | NA | 2 | ALS | MAP | 2 | Web | Sheet web | Acinform | 1 | 6 |
| *Argyroneta aquatica^1^* | NA | 2 | ALS | Piriform | 6 | Web | Sheet web | Acinform | 1 | 6 |
| *Argyroneta aquatica^1^* | NA | 2 | PMS | mAP | 1 | Web | Sheet web | Acinform | 1 | 6 |
| *Argyroneta aquatica^1^* | NA | 2 | PMS | Aciniform | 6 | Web | Sheet web | Acinform | 1 | 6 |
| *Argyroneta aquatica^1^* | NA | 2 | PMS | Cylindrical | 0 | Web | Sheet web | Acinform | 1 | 6 |
| *Argyroneta aquatica^1^* | NA | 2 | PLS | Aciniform | 16 | Web | Sheet web | Acinform | 1 | 6 |
| *Argyroneta aquatica^1^* | NA | 2 | PLS | Cylindrical | 0 | Web | Sheet web | Acinform | 1 | 6 |
| *Argyroneta aquatica^1^* | NA | 3 | Cribellum | Cribellar | NA | Web | Sheet web | Acinform | 1 | 6 |
| *Argyroneta aquatica^1^* | NA | 3 | ALS | MAP | 2 | Web | Sheet web | Acinform | 1 | 6 |
| *Argyroneta aquatica^1^* | NA | 3 | ALS | Piriform | 24 | Web | Sheet web | Acinform | 1 | 6 |
| *Argyroneta aquatica^1^* | NA | 3 | PMS | mAP | 1 | Web | Sheet web | Acinform | 1 | 6 |
| *Argyroneta aquatica^1^* | NA | 3 | PMS | Aciniform | 10 | Web | Sheet web | Acinform | 1 | 6 |
| *Argyroneta aquatica^1^* | NA | 3 | PMS | Cylindrical | 0 | Web | Sheet web | Acinform | 1 | 6 |
| *Argyroneta aquatica^1^* | NA | 3 | PLS | Aciniform | 26 | Web | Sheet web | Acinform | 1 | 6 |
| *Argyroneta aquatica^1^* | NA | 3 | PLS | Cylindrical | 0 | Web | Sheet web | Acinform | 1 | 6 |
| *Argyroneta aquatica^1^* | NA | 4 | Cribellum | Cribellar | NA | Web | Sheet web | Acinform | 1 | 6 |
| *Argyroneta aquatica^1^* | NA | 4 | ALS | MAP | 2 | Web | Sheet web | Acinform | 1 | 6 |
| *Argyroneta aquatica^1^* | NA | 4 | ALS | Piriform | 49 | Web | Sheet web | Acinform | 1 | 6 |
| *Argyroneta aquatica^1^* | NA | 4 | PMS | mAP | 1 | Web | Sheet web | Acinform | 1 | 6 |
| *Argyroneta aquatica^1^* | NA | 4 | PMS | Aciniform | 22 | Web | Sheet web | Acinform | 1 | 6 |
| *Argyroneta aquatica^1^* | NA | 4 | PMS | Cylindrical | 0 | Web | Sheet web | Acinform | 1 | 6 |
| *Argyroneta aquatica^1^* | NA | 4 | PLS | Aciniform | 42 | Web | Sheet web | Acinform | 1 | 6 |
| *Argyroneta aquatica^1^* | NA | 4 | PLS | Cylindrical | 0 | Web | Sheet web | Acinform | 1 | 6 |
| *Argyroneta aquatica^1^* | Pen Male | 5 | Cribellum | Cribellar | NA | Web | Sheet web | Acinform | 1 | 6 |
| *Argyroneta aquatica^1^* | Pen Male | 5 | ALS | MAP | 2 | Web | Sheet web | Acinform | 1 | 6 |
| *Argyroneta aquatica^1^* | Pen Male | 5 | ALS | Piriform | 59 | Web | Sheet web | Acinform | 1 | 6 |
| *Argyroneta aquatica^1^* | Pen Male | 5 | PMS | mAP | 1 | Web | Sheet web | Acinform | 1 | 6 |
| *Argyroneta aquatica^1^* | Pen Male | 5 | PMS | Aciniform | 38 | Web | Sheet web | Acinform | 1 | 6 |
| *Argyroneta aquatica^1^* | Pen Male | 5 | PMS | Cylindrical | 0 | Web | Sheet web | Acinform | 1 | 6 |
| *Argyroneta aquatica^1^* | Pen Male | 5 | PLS | Aciniform | 65 | Web | Sheet web | Acinform | 1 | 6 |
| *Argyroneta aquatica^1^* | Pen Male | 5 | PLS | Cylindrical | 0 | Web | Sheet web | Acinform | 1 | 6 |
| *Argyroneta aquatica^1^* | Pen Fem | 5 | Cribellum | Cribellar | NA | Web | Sheet web | Acinform | 1 | 6 |
| *Argyroneta aquatica^1^* | Pen Fem | 5 | ALS | MAP | 2 | Web | Sheet web | Acinform | 1 | 6 |
| *Argyroneta aquatica^1^* | Pen Fem | 5 | ALS | Piriform | 69 | Web | Sheet web | Acinform | 1 | 6 |
| *Argyroneta aquatica^1^* | Pen Fem | 5 | PMS | mAP | 1 | Web | Sheet web | Acinform | 1 | 6 |
| *Argyroneta aquatica^1^* | Pen Fem | 5 | PMS | Aciniform | 36 | Web | Sheet web | Acinform | 1 | 6 |
| *Argyroneta aquatica^1^* | Pen Fem | 5 | PMS | Cylindrical | 0 | Web | Sheet web | Acinform | 1 | 6 |
| *Argyroneta aquatica^1^* | Pen Fem | 5 | PLS | Aciniform | 96 | Web | Sheet web | Acinform | 1 | 6 |
| *Argyroneta aquatica^1^* | Pen Fem | 5 | PLS | Cylindrical | 0 | Web | Sheet web | Acinform | 1 | 6 |
| *Argyroneta aquatica^1^* | Male | 6 | Cribellum | Cribellar | NA | Web | Sheet web | Acinform | 1 | 6 |
| *Argyroneta aquatica^1^* | Male | 6 | ALS | MAP | 2 | Web | Sheet web | Acinform | 1 | 6 |
| *Argyroneta aquatica^1^* | Male | 6 | ALS | Piriform | 97 | Web | Sheet web | Acinform | 1 | 6 |
| *Argyroneta aquatica^1^* | Male | 6 | PMS | mAP | 1 | Web | Sheet web | Acinform | 1 | 6 |
| *Argyroneta aquatica^1^* | Male | 6 | PMS | Aciniform | 55 | Web | Sheet web | Acinform | 1 | 6 |
| *Argyroneta aquatica^1^* | Male | 6 | PMS | Cylindrical | 0 | Web | Sheet web | Acinform | 1 | 6 |
| *Argyroneta aquatica^1^* | Male | 6 | PLS | Aciniform | 89 | Web | Sheet web | Acinform | 1 | 6 |
| *Argyroneta aquatica^1^* | Male | 6 | PLS | Cylindrical | 0 | Web | Sheet web | Acinform | 1 | 6 |
| *Argyroneta aquatica^1^* | Female | 6 | Cribellum | Cribellar | NA | Web | Sheet web | Acinform | 1 | 6 |
| *Argyroneta aquatica^1^* | Female | 6 | ALS | MAP | 2 | Web | Sheet web | Acinform | 1 | 6 |
| *Argyroneta aquatica^1^* | Female | 6 | ALS | Piriform | 84 | Web | Sheet web | Acinform | 1 | 6 |
| *Argyroneta aquatica^1^* | Female | 6 | PMS | mAP | 1 | Web | Sheet web | Acinform | 1 | 6 |
| *Argyroneta aquatica^1^* | Female | 6 | PMS | Aciniform | 34 | Web | Sheet web | Acinform | 1 | 6 |
| *Argyroneta aquatica^1^* | Female | 6 | PMS | Cylindrical | 7 | Web | Sheet web | Acinform | 1 | 6 |
| *Argyroneta aquatica^1^* | Female | 6 | PLS | Aciniform | 110 | Web | Sheet web | Acinform | 1 | 6 |
| *Argyroneta aquatica^1^* | Female | 6 | PLS | Cylindrical | 8 | Web | Sheet web | Acinform | 1 | 6 |
| *Eratigena atrica^1^* | NA | 2 | Cribellum | Cribellar | NA | Web | Funnel web | Aciniform | 1 | 9 |
| *Eratigena atrica^1^* | NA | 2 | ALS | MAP | 2 | Web | Funnel web | Aciniform | 1 | 9 |
| *Eratigena atrica^1^* | NA | 2 | ALS | Piriform | 10 | Web | Funnel web | Aciniform | 1 | 9 |
| *Eratigena atrica^1^* | NA | 2 | PMS | mAP | 1 | Web | Funnel web | Aciniform | 1 | 9 |
| *Eratigena atrica^1^* | NA | 2 | PMS | Aciniform | 4 | Web | Funnel web | Aciniform | 1 | 9 |
| *Eratigena atrica^1^* | NA | 2 | PMS | Cylindrical | 0 | Web | Funnel web | Aciniform | 1 | 9 |
| *Eratigena atrica^1^* | NA | 2 | PLS | Aciniform | 5 | Web | Funnel web | Aciniform | 1 | 9 |
| *Eratigena atrica^1^* | NA | 2 | PLS | Cylindrical | 0 | Web | Funnel web | Aciniform | 1 | 9 |
| *Eratigena atrica^1^* | NA | 3 | Cribellum | Cribellar | NA | Web | Funnel web | Aciniform | 1 | 9 |
| *Eratigena atrica^1^* | NA | 3 | ALS | MAP | 2 | Web | Funnel web | Aciniform | 1 | 9 |
| *Eratigena atrica^1^* | NA | 3 | ALS | Piriform | 16 | Web | Funnel web | Aciniform | 1 | 9 |
| *Eratigena atrica^1^* | NA | 3 | PMS | mAP | 1 | Web | Funnel web | Aciniform | 1 | 9 |
| *Eratigena atrica^1^* | NA | 3 | PMS | Aciniform | 5 | Web | Funnel web | Aciniform | 1 | 9 |
| *Eratigena atrica^1^* | NA | 3 | PMS | Cylindrical | 0 | Web | Funnel web | Aciniform | 1 | 9 |
| *Eratigena atrica^1^* | NA | 3 | PLS | Aciniform | 9 | Web | Funnel web | Aciniform | 1 | 9 |
| *Eratigena atrica^1^* | NA | 3 | PLS | Cylindrical | 0 | Web | Funnel web | Aciniform | 1 | 9 |
| *Eratigena atrica^1^* | NA | 4 | Cribellum | Cribellar | NA | Web | Funnel web | Aciniform | 1 | 9 |
| *Eratigena atrica^1^* | NA | 4 | ALS | MAP | 2 | Web | Funnel web | Aciniform | 1 | 9 |
| *Eratigena atrica^1^* | NA | 4 | ALS | Piriform | 27 | Web | Funnel web | Aciniform | 1 | 9 |
| *Eratigena atrica^1^* | NA | 4 | PMS | mAP | 1 | Web | Funnel web | Aciniform | 1 | 9 |
| *Eratigena atrica^1^* | NA | 4 | PMS | Aciniform | 10 | Web | Funnel web | Aciniform | 1 | 9 |
| *Eratigena atrica^1^* | NA | 4 | PMS | Cylindrical | 0 | Web | Funnel web | Aciniform | 1 | 9 |
| *Eratigena atrica^1^* | NA | 4 | PLS | Aciniform | 13 | Web | Funnel web | Aciniform | 1 | 9 |
| *Eratigena atrica^1^* | NA | 4 | PLS | Cylindrical | 0 | Web | Funnel web | Aciniform | 1 | 9 |
| *Eratigena atrica^1^* | NA | 5 | Cribellum | Cribellar | NA | Web | Funnel web | Aciniform | 1 | 9 |
| *Eratigena atrica^1^* | NA | 5 | ALS | MAP | 2 | Web | Funnel web | Aciniform | 1 | 9 |
| *Eratigena atrica^1^* | NA | 5 | ALS | Piriform | 32 | Web | Funnel web | Aciniform | 1 | 9 |
| *Eratigena atrica^1^* | NA | 5 | PMS | mAP | 1 | Web | Funnel web | Aciniform | 1 | 9 |
| *Eratigena atrica^1^* | NA | 5 | PMS | Aciniform | 14 | Web | Funnel web | Aciniform | 1 | 9 |
| *Eratigena atrica^1^* | NA | 5 | PMS | Cylindrical | 0 | Web | Funnel web | Aciniform | 1 | 9 |
| *Eratigena atrica^1^* | NA | 5 | PLS | Aciniform | 15 | Web | Funnel web | Aciniform | 1 | 9 |
| *Eratigena atrica^1^* | NA | 5 | PLS | Cylindrical | 0 | Web | Funnel web | Aciniform | 1 | 9 |
| *Eratigena atrica^1^* | NA | 6 | Cribellum | Cribellar | NA | Web | Funnel web | Aciniform | 1 | 9 |
| *Eratigena atrica^1^* | NA | 6 | ALS | MAP | 2 | Web | Funnel web | Aciniform | 1 | 9 |
| *Eratigena atrica^1^* | NA | 6 | ALS | Piriform | 45 | Web | Funnel web | Aciniform | 1 | 9 |
| *Eratigena atrica^1^* | NA | 6 | PMS | mAP | 1 | Web | Funnel web | Aciniform | 1 | 9 |
| *Eratigena atrica^1^* | NA | 6 | PMS | Aciniform | 16 | Web | Funnel web | Aciniform | 1 | 9 |
| *Eratigena atrica^1^* | NA | 6 | PMS | Cylindrical | 0 | Web | Funnel web | Aciniform | 1 | 9 |
| *Eratigena atrica^1^* | NA | 6 | PLS | Aciniform | 16 | Web | Funnel web | Aciniform | 1 | 9 |
| *Eratigena atrica^1^* | NA | 6 | PLS | Cylindrical | 0 | Web | Funnel web | Aciniform | 1 | 9 |
| *Eratigena atrica^1^* | NA | 7 | Cribellum | Cribellar | NA | Web | Funnel web | Aciniform | 1 | 9 |
| *Eratigena atrica^1^* | NA | 7 | ALS | MAP | 2 | Web | Funnel web | Aciniform | 1 | 9 |
| *Eratigena atrica^1^* | NA | 7 | ALS | Piriform | 54 | Web | Funnel web | Aciniform | 1 | 9 |
| *Eratigena atrica^1^* | NA | 7 | PMS | mAP | 1 | Web | Funnel web | Aciniform | 1 | 9 |
| *Eratigena atrica^1^* | NA | 7 | PMS | Aciniform | 16 | Web | Funnel web | Aciniform | 1 | 9 |
| *Eratigena atrica^1^* | NA | 7 | PMS | Cylindrical | 0 | Web | Funnel web | Aciniform | 1 | 9 |
| *Eratigena atrica^1^* | NA | 7 | PLS | Aciniform | 18 | Web | Funnel web | Aciniform | 1 | 9 |
| *Eratigena atrica^1^* | NA | 7 | PLS | Cylindrical | 0 | Web | Funnel web | Aciniform | 1 | 9 |
| *Eratigena atrica^1^* | Pen Male | 8 | Cribellum | Cribellar | NA | Web | Funnel web | Aciniform | 1 | 9 |
| *Eratigena atrica^1^* | Pen Male | 8 | ALS | MAP | 2 | Web | Funnel web | Aciniform | 1 | 9 |
| *Eratigena atrica^1^* | Pen Male | 8 | ALS | Piriform | 66 | Web | Funnel web | Aciniform | 1 | 9 |
| *Eratigena atrica^1^* | Pen Male | 8 | PMS | mAP | 1 | Web | Funnel web | Aciniform | 1 | 9 |
| *Eratigena atrica^1^* | Pen Male | 8 | PMS | Aciniform | 16 | Web | Funnel web | Aciniform | 1 | 9 |
| *Eratigena atrica^1^* | Pen Male | 8 | PMS | Cylindrical | 0 | Web | Funnel web | Aciniform | 1 | 9 |
| *Eratigena atrica^1^* | Pen Male | 8 | PLS | Aciniform | 23 | Web | Funnel web | Aciniform | 1 | 9 |
| *Eratigena atrica^1^* | Pen Male | 8 | PLS | Cylindrical | 0 | Web | Funnel web | Aciniform | 1 | 9 |
| *Eratigena atrica^1^* | Pen Fem | 8 | Cribellum | Cribellar | NA | Web | Funnel web | Aciniform | 1 | 9 |
| *Eratigena atrica^1^* | Pen Fem | 8 | ALS | MAP | 2 | Web | Funnel web | Aciniform | 1 | 9 |
| *Eratigena atrica^1^* | Pen Fem | 8 | ALS | Piriform | 73 | Web | Funnel web | Aciniform | 1 | 9 |
| *Eratigena atrica^1^* | Pen Fem | 8 | PMS | mAP | 1 | Web | Funnel web | Aciniform | 1 | 9 |
| *Eratigena atrica^1^* | Pen Fem | 8 | PMS | Aciniform | 18 | Web | Funnel web | Aciniform | 1 | 9 |
| *Eratigena atrica^1^* | Pen Fem | 8 | PMS | Cylindrical | 0 | Web | Funnel web | Aciniform | 1 | 9 |
| *Eratigena atrica^1^* | Pen Fem | 8 | PLS | Aciniform | 25 | Web | Funnel web | Aciniform | 1 | 9 |
| *Eratigena atrica^1^* | Pen Fem | 8 | PLS | Cylindrical | 0 | Web | Funnel web | Aciniform | 1 | 9 |
| *Eratigena atrica^1^* | Male | 9 | Cribellum | Cribellar | NA | Web | Funnel web | Aciniform | 1 | 9 |
| *Eratigena atrica^1^* | Male | 9 | ALS | MAP | 2 | Web | Funnel web | Aciniform | 1 | 9 |
| *Eratigena atrica^1^* | Male | 9 | ALS | Piriform | 80 | Web | Funnel web | Aciniform | 1 | 9 |
| *Eratigena atrica^1^* | Male | 9 | PMS | mAP | 1 | Web | Funnel web | Aciniform | 1 | 9 |
| *Eratigena atrica^1^* | Male | 9 | PMS | Aciniform | 16 | Web | Funnel web | Aciniform | 1 | 9 |
| *Eratigena atrica^1^* | Male | 9 | PMS | Cylindrical | 0 | Web | Funnel web | Aciniform | 1 | 9 |
| *Eratigena atrica^1^* | Male | 9 | PLS | Aciniform | 25 | Web | Funnel web | Aciniform | 1 | 9 |
| *Eratigena atrica^1^* | Male | 9 | PLS | Cylindrical | 0 | Web | Funnel web | Aciniform | 1 | 9 |
| *Eratigena atrica^1^* | Female | 9 | Cribellum | Cribellar | NA | Web | Funnel web | Aciniform | 1 | 9 |
| *Eratigena atrica^1^* | Female | 9 | ALS | MAP | 2 | Web | Funnel web | Aciniform | 1 | 9 |
| *Eratigena atrica^1^* | Female | 9 | ALS | Piriform | 83 | Web | Funnel web | Aciniform | 1 | 9 |
| *Eratigena atrica^1^* | Female | 9 | PMS | mAP | 1 | Web | Funnel web | Aciniform | 1 | 9 |
| *Eratigena atrica^1^* | Female | 9 | PMS | Aciniform | 21 | Web | Funnel web | Aciniform | 1 | 9 |
| *Eratigena atrica^1^* | Female | 9 | PMS | Cylindrical | 3 | Web | Funnel web | Aciniform | 1 | 9 |
| *Eratigena atrica^1^* | Female | 9 | PLS | Aciniform | 28 | Web | Funnel web | Aciniform | 1 | 9 |
| *Eratigena atrica^1^* | Female | 9 | PLS | Cylindrical | 4 | Web | Funnel web | Aciniform | 1 | 9 |
| *Phyxelida tanganensis^4^* | NA | 2 | Cribellum | Cribellar | 0 | Web | Sheet web | Cribellate | 4 | 8 |
| *Phyxelida tanganensis^4^* | NA | 2 | ALS | MAP | 2 | Web | Sheet web | Cribellate | 4 | 8 |
| *Phyxelida tanganensis^4^* | NA | 2 | ALS | Piriform | 4 | Web | Sheet web | Cribellate | 4 | 8 |
| *Phyxelida tanganensis^4^* | NA | 2 | PMS | mAP | 1 | Web | Sheet web | Cribellate | 4 | 8 |
| *Phyxelida tanganensis^4^* | NA | 2 | PMS | Aciniform | 0 | Web | Sheet web | Cribellate | 4 | 8 |
| *Phyxelida tanganensis^4^* | NA | 2 | PMS | Paracribellar | 0 | Web | Sheet web | Cribellate | 4 | 8 |
| *Phyxelida tanganensis^4^* | NA | 2 | PMS | Cylindrical | 0 | Web | Sheet web | Cribellate | 4 | 8 |
| *Phyxelida tanganensis^4^* | NA | 2 | PLS | Aciniform | 3 | Web | Sheet web | Cribellate | 4 | 8 |
| *Phyxelida tanganensis^4^* | NA | 2 | PLS | Pseudoflagelliform | 1 | Web | Sheet web | Cribellate | 4 | 8 |
| *Phyxelida tanganensis^4^* | NA | 2 | PLS | Cylindrical | 0 | Web | Sheet web | Cribellate | 4 | 8 |
| *Phyxelida tanganensis^4^* | NA | 3 | Cribellum | Cribellar | NA | Web | Sheet web | Cribellate | 4 | 8 |
| *Phyxelida tanganensis^4^* | NA | 3 | ALS | MAP | 2 | Web | Sheet web | Cribellate | 4 | 8 |
| *Phyxelida tanganensis^4^* | NA | 3 | ALS | Piriform | 5 | Web | Sheet web | Cribellate | 4 | 8 |
| *Phyxelida tanganensis^4^* | NA | 3 | PMS | mAP | 1 | Web | Sheet web | Cribellate | 4 | 8 |
| *Phyxelida tanganensis^4^* | NA | 3 | PMS | Aciniform | 1 | Web | Sheet web | Cribellate | 4 | 8 |
| *Phyxelida tanganensis^4^* | NA | 3 | PMS | Paracribellar | 3 | Web | Sheet web | Cribellate | 4 | 8 |
| *Phyxelida tanganensis^4^* | NA | 3 | PMS | Cylindrical | 0 | Web | Sheet web | Cribellate | 4 | 8 |
| *Phyxelida tanganensis^4^* | NA | 3 | PLS | Aciniform | 4 | Web | Sheet web | Cribellate | 4 | 8 |
| *Phyxelida tanganensis^4^* | NA | 3 | PLS | Pseudoflagelliform | 1 | Web | Sheet web | Cribellate | 4 | 8 |
| *Phyxelida tanganensis^4^* | NA | 3 | PLS | Cylindrical | 0 | Web | Sheet web | Cribellate | 4 | 8 |
| *Phyxelida tanganensis^4^* | NA | 4 | Cribellum | Cribellar | NA | Web | Sheet web | Cribellate | 4 | 8 |
| *Phyxelida tanganensis^4^* | NA | 4 | ALS | MAP | 2 | Web | Sheet web | Cribellate | 4 | 8 |
| *Phyxelida tanganensis^4^* | NA | 4 | ALS | Piriform | 9 | Web | Sheet web | Cribellate | 4 | 8 |
| *Phyxelida tanganensis^4^* | NA | 4 | PMS | mAP | 1 | Web | Sheet web | Cribellate | 4 | 8 |
| *Phyxelida tanganensis^4^* | NA | 4 | PMS | Aciniform | 3 | Web | Sheet web | Cribellate | 4 | 8 |
| *Phyxelida tanganensis^4^* | NA | 4 | PMS | Paracribellar | 6 | Web | Sheet web | Cribellate | 4 | 8 |
| *Phyxelida tanganensis^4^* | NA | 4 | PMS | Cylindrical | -1 | Web | Sheet web | Cribellate | 4 | 8 |
| *Phyxelida tanganensis^4^* | NA | 4 | PLS | Aciniform | 6 | Web | Sheet web | Cribellate | 4 | 8 |
| *Phyxelida tanganensis^4^* | NA | 4 | PLS | Pseudoflagelliform | 1 | Web | Sheet web | Cribellate | 4 | 8 |
| *Phyxelida tanganensis^4^* | NA | 4 | PLS | Cylindrical | 0 | Web | Sheet web | Cribellate | 4 | 8 |
| *Phyxelida tanganensis^4^* | NA | 5 | Cribellum | Cribellar | NA | Web | Sheet web | Cribellate | 4 | 8 |
| *Phyxelida tanganensis^4^* | NA | 5 | ALS | MAP | 2 | Web | Sheet web | Cribellate | 4 | 8 |
| *Phyxelida tanganensis^4^* | NA | 5 | ALS | Piriform | 16 | Web | Sheet web | Cribellate | 4 | 8 |
| *Phyxelida tanganensis^4^* | NA | 5 | PMS | mAP | 1 | Web | Sheet web | Cribellate | 4 | 8 |
| *Phyxelida tanganensis^4^* | NA | 5 | PMS | Aciniform | 6 | Web | Sheet web | Cribellate | 4 | 8 |
| *Phyxelida tanganensis^4^* | NA | 5 | PMS | Paracribellar | 8 | Web | Sheet web | Cribellate | 4 | 8 |
| *Phyxelida tanganensis^4^* | NA | 5 | PMS | Cylindrical | 0 | Web | Sheet web | Cribellate | 4 | 8 |
| *Phyxelida tanganensis^4^* | NA | 5 | PLS | Aciniform | 8 | Web | Sheet web | Cribellate | 4 | 8 |
| *Phyxelida tanganensis^4^* | NA | 5 | PLS | Pseudoflagelliform | 1 | Web | Sheet web | Cribellate | 4 | 8 |
| *Phyxelida tanganensis^4^* | NA | 5 | PLS | Cylindrical | 0 | Web | Sheet web | Cribellate | 4 | 8 |
| *Phyxelida tanganensis^4^* | Ante Pen Fem | 6 | Cribellum | Cribellar | NA | Web | Sheet web | Cribellate | 4 | 8 |
| *Phyxelida tanganensis^4^* | Ante Pen Fem | 6 | ALS | MAP | 2 | Web | Sheet web | Cribellate | 4 | 8 |
| *Phyxelida tanganensis^4^* | Ante Pen Fem | 6 | ALS | Piriform | 26 | Web | Sheet web | Cribellate | 4 | 8 |
| *Phyxelida tanganensis^4^* | Ante Pen Fem | 6 | PMS | mAP | 1 | Web | Sheet web | Cribellate | 4 | 8 |
| *Phyxelida tanganensis^4^* | Ante Pen Fem | 6 | PMS | Aciniform | 6 | Web | Sheet web | Cribellate | 4 | 8 |
| *Phyxelida tanganensis^4^* | Ante Pen Fem | 6 | PMS | Paracribellar | 10 | Web | Sheet web | Cribellate | 4 | 8 |
| *Phyxelida tanganensis^4^* | Ante Pen Fem | 6 | PMS | Cylindrical | -1 | Web | Sheet web | Cribellate | 4 | 8 |
| *Phyxelida tanganensis^4^* | Ante Pen Fem | 6 | PLS | Aciniform | 12 | Web | Sheet web | Cribellate | 4 | 8 |
| *Phyxelida tanganensis^4^* | Ante Pen Fem | 6 | PLS | Pseudoflagelliform | 1 | Web | Sheet web | Cribellate | 4 | 8 |
| *Phyxelida tanganensis^4^* | Ante Pen Fem | 6 | PLS | Cylindrical | 2 | Web | Sheet web | Cribellate | 4 | 8 |
| *Phyxelida tanganensis^4^* | Pen Fem | 7 | Cribellum | Cribellar | NA | Web | Sheet web | Cribellate | 4 | 8 |
| *Phyxelida tanganensis^4^* | Pen Fem | 7 | ALS | MAP | 2 | Web | Sheet web | Cribellate | 4 | 8 |
| *Phyxelida tanganensis^4^* | Pen Fem | 7 | ALS | Piriform | 72 | Web | Sheet web | Cribellate | 4 | 8 |
| *Phyxelida tanganensis^4^* | Pen Fem | 7 | PMS | mAP | 1 | Web | Sheet web | Cribellate | 4 | 8 |
| *Phyxelida tanganensis^4^* | Pen Fem | 7 | PMS | Aciniform | 11 | Web | Sheet web | Cribellate | 4 | 8 |
| *Phyxelida tanganensis^4^* | Pen Fem | 7 | PMS | Paracribellar | 13 | Web | Sheet web | Cribellate | 4 | 8 |
| *Phyxelida tanganensis^4^* | Pen Fem | 7 | PMS | Cylindrical | 2 | Web | Sheet web | Cribellate | 4 | 8 |
| *Phyxelida tanganensis^4^* | Pen Fem | 7 | PLS | Aciniform | 17 | Web | Sheet web | Cribellate | 4 | 8 |
| *Phyxelida tanganensis^4^* | Pen Fem | 7 | PLS | Pseudoflagelliform | 1 | Web | Sheet web | Cribellate | 4 | 8 |
| *Phyxelida tanganensis^4^* | Pen Fem | 7 | PLS | Cylindrical | 2 | Web | Sheet web | Cribellate | 4 | 8 |
| *Phyxelida tanganensis^4^* | Pen Male | 7 | Cribellum | Cribellar | NA | Web | Sheet web | Cribellate | 4 | 8 |
| *Phyxelida tanganensis^4^* | Pen Male | 7 | ALS | MAP | 2 | Web | Sheet web | Cribellate | 4 | 8 |
| *Phyxelida tanganensis^4^* | Pen Male | 7 | ALS | Piriform | 40 | Web | Sheet web | Cribellate | 4 | 8 |
| *Phyxelida tanganensis^4^* | Pen Male | 7 | PMS | mAP | 1 | Web | Sheet web | Cribellate | 4 | 8 |
| *Phyxelida tanganensis^4^* | Pen Male | 7 | PMS | Aciniform | 7 | Web | Sheet web | Cribellate | 4 | 8 |
| *Phyxelida tanganensis^4^* | Pen Male | 7 | PMS | Paracribellar | 11 | Web | Sheet web | Cribellate | 4 | 8 |
| *Phyxelida tanganensis^4^* | Pen Male | 7 | PMS | Cylindrical | -1 | Web | Sheet web | Cribellate | 4 | 8 |
| *Phyxelida tanganensis^4^* | Pen Male | 7 | PLS | Aciniform | 13 | Web | Sheet web | Cribellate | 4 | 8 |
| *Phyxelida tanganensis^4^* | Pen Male | 7 | PLS | Pseudoflagelliform | 1 | Web | Sheet web | Cribellate | 4 | 8 |
| *Phyxelida tanganensis^4^* | Pen Male | 7 | PLS | Cylindrical | 0 | Web | Sheet web | Cribellate | 4 | 8 |
| *Phyxelida tanganensis^4^* | Female | 8 | Cribellum | Cribellar | NA | Web | Sheet web | Cribellate | 4 | 8 |
| *Phyxelida tanganensis^4^* | Female | 8 | ALS | MAP | 2 | Web | Sheet web | Cribellate | 4 | 8 |
| *Phyxelida tanganensis^4^* | Female | 8 | ALS | Piriform | 76 | Web | Sheet web | Cribellate | 4 | 8 |
| *Phyxelida tanganensis^4^* | Female | 8 | PMS | mAP | 1 | Web | Sheet web | Cribellate | 4 | 8 |
| *Phyxelida tanganensis^4^* | Female | 8 | PMS | Aciniform | 11 | Web | Sheet web | Cribellate | 4 | 8 |
| *Phyxelida tanganensis^4^* | Female | 8 | PMS | Paracribellar | 14 | Web | Sheet web | Cribellate | 4 | 8 |
| *Phyxelida tanganensis^4^* | Female | 8 | PMS | Cylindrical | 2 | Web | Sheet web | Cribellate | 4 | 8 |
| *Phyxelida tanganensis^4^* | Female | 8 | PLS | Aciniform | 16 | Web | Sheet web | Cribellate | 4 | 8 |
| *Phyxelida tanganensis^4^* | Female | 8 | PLS | Pseudoflagelliform | 1 | Web | Sheet web | Cribellate | 4 | 8 |
| *Phyxelida tanganensis^4^* | Female | 8 | PLS | Cylindrical | 2 | Web | Sheet web | Cribellate | 4 | 8 |
| *Phyxelida tanganensis^4^* | Male | 8 | Cribellum | Cribellar | NA | Web | Sheet web | Cribellate | 4 | 8 |
| *Phyxelida tanganensis^4^* | Male | 8 | ALS | MAP | 1 | Web | Sheet web | Cribellate | 4 | 8 |
| *Phyxelida tanganensis^4^* | Male | 8 | ALS | Piriform | 15 | Web | Sheet web | Cribellate | 4 | 8 |
| *Phyxelida tanganensis^4^* | Male | 8 | PMS | mAP | 0 | Web | Sheet web | Cribellate | 4 | 8 |
| *Phyxelida tanganensis^4^* | Male | 8 | PMS | Aciniform | 7 | Web | Sheet web | Cribellate | 4 | 8 |
| *Phyxelida tanganensis^4^* | Male | 8 | PMS | Paracribellar | 0 | Web | Sheet web | Cribellate | 4 | 8 |
| *Phyxelida tanganensis^4^* | Male | 8 | PMS | Cylindrical | 0 | Web | Sheet web | Cribellate | 4 | 8 |
| *Phyxelida tanganensis^4^* | Male | 8 | PLS | Aciniform | 10 | Web | Sheet web | Cribellate | 4 | 8 |
| *Phyxelida tanganensis^4^* | Male | 8 | PLS | Pseudoflagelliform | 0 | Web | Sheet web | Cribellate | 4 | 8 |
| *Phyxelida tanganensis^4^* | Male | 8 | PLS | Cylindrical | 0 | Web | Sheet web | Cribellate | 4 | 8 |
| *Hyptiotes paradoxus^2^* | NA | 2 | Cribellum | Cribellar | NA | Web | Orb web | Cribellate | 4 | 6 |
| *Hyptiotes paradoxus^2^* | NA | 2 | ALS | MAP | 2 | Web | Orb web | Cribellate | 4 | 6 |
| *Hyptiotes paradoxus^2^* | NA | 2 | ALS | Piriform | 6 | Web | Orb web | Cribellate | 4 | 6 |
| *Hyptiotes paradoxus^2^* | NA | 2 | PMS | mAP | 0 | Web | Orb web | Cribellate | 4 | 6 |
| *Hyptiotes paradoxus^2^* | NA | 2 | PMS | Aciniform | 4 | Web | Orb web | Cribellate | 4 | 6 |
| *Hyptiotes paradoxus^2^* | NA | 2 | PMS | Paracribellar | 0 | Web | Orb web | Cribellate | 4 | 6 |
| *Hyptiotes paradoxus^2^* | NA | 2 | PMS | Cylindrical | 0 | Web | Orb web | Cribellate | 4 | 6 |
| *Hyptiotes paradoxus^2^* | NA | 2 | PLS | Aciniform | 8 | Web | Orb web | Cribellate | 4 | 6 |
| *Hyptiotes paradoxus^2^* | NA | 2 | PLS | Pseudoflagelliform | 0 | Web | Orb web | Cribellate | 4 | 6 |
| *Hyptiotes paradoxus^2^* | NA | 2 | PLS | Cylindrical | 0 | Web | Orb web | Cribellate | 4 | 6 |
| *Hyptiotes paradoxus^2^* | NA | 3 | Cribellum | Cribellar | NA | Web | Orb web | Cribellate | 4 | 6 |
| *Hyptiotes paradoxus^2^* | NA | 3 | ALS | MAP | 1 | Web | Orb web | Cribellate | 4 | 6 |
| *Hyptiotes paradoxus^2^* | NA | 3 | ALS | Piriform | 8 | Web | Orb web | Cribellate | 4 | 6 |
| *Hyptiotes paradoxus^2^* | NA | 3 | PMS | mAP | 1 | Web | Orb web | Cribellate | 4 | 6 |
| *Hyptiotes paradoxus^2^* | NA | 3 | PMS | Aciniform | 6 | Web | Orb web | Cribellate | 4 | 6 |
| *Hyptiotes paradoxus^2^* | NA | 3 | PMS | Paracribellar | 6 | Web | Orb web | Cribellate | 4 | 6 |
| *Hyptiotes paradoxus^2^* | NA | 3 | PMS | Cylindrical | 1 | Web | Orb web | Cribellate | 4 | 6 |
| *Hyptiotes paradoxus^2^* | NA | 3 | PLS | Aciniform | 10 | Web | Orb web | Cribellate | 4 | 6 |
| *Hyptiotes paradoxus^2^* | NA | 3 | PLS | Pseudoflagelliform | 1 | Web | Orb web | Cribellate | 4 | 6 |
| *Hyptiotes paradoxus^2^* | NA | 3 | PLS | Cylindrical | 0 | Web | Orb web | Cribellate | 4 | 6 |
| *Hyptiotes paradoxus^2^* | NA | 4 | Cribellum | Cribellar | NA | Web | Orb web | Cribellate | 4 | 6 |
| *Hyptiotes paradoxus^2^* | NA | 4 | ALS | MAP | 1 | Web | Orb web | Cribellate | 4 | 6 |
| *Hyptiotes paradoxus^2^* | NA | 4 | ALS | Piriform | 22 | Web | Orb web | Cribellate | 4 | 6 |
| *Hyptiotes paradoxus^2^* | NA | 4 | PMS | mAP | 1 | Web | Orb web | Cribellate | 4 | 6 |
| *Hyptiotes paradoxus^2^* | NA | 4 | PMS | Aciniform | 12 | Web | Orb web | Cribellate | 4 | 6 |
| *Hyptiotes paradoxus^2^* | NA | 4 | PMS | Paracribellar | 18 | Web | Orb web | Cribellate | 4 | 6 |
| *Hyptiotes paradoxus^2^* | NA | 4 | PMS | Cylindrical | 1 | Web | Orb web | Cribellate | 4 | 6 |
| *Hyptiotes paradoxus^2^* | NA | 4 | PLS | Aciniform | 24 | Web | Orb web | Cribellate | 4 | 6 |
| *Hyptiotes paradoxus^2^* | NA | 4 | PLS | Pseudoflagelliform | 1 | Web | Orb web | Cribellate | 4 | 6 |
| *Hyptiotes paradoxus^2^* | NA | 4 | PLS | Cylindrical | 0 | Web | Orb web | Cribellate | 4 | 6 |
| *Hyptiotes paradoxus^2^* | NA | 5 | Cribellum | Cribellar | NA | Web | Orb web | Cribellate | 4 | 6 |
| *Hyptiotes paradoxus^2^* | NA | 5 | ALS | MAP | 1 | Web | Orb web | Cribellate | 4 | 6 |
| *Hyptiotes paradoxus^2^* | NA | 5 | ALS | Piriform | 38 | Web | Orb web | Cribellate | 4 | 6 |
| *Hyptiotes paradoxus^2^* | NA | 5 | PMS | mAP | 1 | Web | Orb web | Cribellate | 4 | 6 |
| *Hyptiotes paradoxus^2^* | NA | 5 | PMS | Aciniform | 17 | Web | Orb web | Cribellate | 4 | 6 |
| *Hyptiotes paradoxus^2^* | NA | 5 | PMS | Paracribellar | 22 | Web | Orb web | Cribellate | 4 | 6 |
| *Hyptiotes paradoxus^2^* | NA | 5 | PMS | Cylindrical | 1 | Web | Orb web | Cribellate | 4 | 6 |
| *Hyptiotes paradoxus^2^* | NA | 5 | PLS | Aciniform | 46 | Web | Orb web | Cribellate | 4 | 6 |
| *Hyptiotes paradoxus^2^* | NA | 5 | PLS | Pseudoflagelliform | 1 | Web | Orb web | Cribellate | 4 | 6 |
| *Hyptiotes paradoxus^2^* | NA | 5 | PLS | Cylindrical | 0 | Web | Orb web | Cribellate | 4 | 6 |
| *Hyptiotes paradoxus^2^* | Female | 6 | Cribellum | Cribellar | NA | Web | Orb web | Cribellate | 4 | 6 |
| *Hyptiotes paradoxus^2^* | Female | 6 | ALS | MAP | 1 | Web | Orb web | Cribellate | 4 | 6 |
| *Hyptiotes paradoxus^2^* | Female | 6 | ALS | Piriform | 71 | Web | Orb web | Cribellate | 4 | 6 |
| *Hyptiotes paradoxus^2^* | Female | 6 | PMS | mAP | 1 | Web | Orb web | Cribellate | 4 | 6 |
| *Hyptiotes paradoxus^2^* | Female | 6 | PMS | Aciniform | 28 | Web | Orb web | Cribellate | 4 | 6 |
| *Hyptiotes paradoxus^2^* | Female | 6 | PMS | Paracribellar | 26 | Web | Orb web | Cribellate | 4 | 6 |
| *Hyptiotes paradoxus^2^* | Female | 6 | PMS | Cylindrical | 1 | Web | Orb web | Cribellate | 4 | 6 |
| *Hyptiotes paradoxus^2^* | Female | 6 | PLS | Aciniform | 90 | Web | Orb web | Cribellate | 4 | 6 |
| *Hyptiotes paradoxus^2^* | Female | 6 | PLS | Pseudoflagelliform | 1 | Web | Orb web | Cribellate | 4 | 6 |
| *Hyptiotes paradoxus^2^* | Female | 6 | PLS | Cylindrical | 4 | Web | Orb web | Cribellate | 4 | 6 |
| *Hyptiotes paradoxus^2^* | Male | 6 | Cribellum | Cribellar | 0 | Web | Orb web | Cribellate | 4 | 6 |
| *Hyptiotes paradoxus^2^* | Male | 6 | ALS | MAP | 1 | Web | Orb web | Cribellate | 4 | 6 |
| *Hyptiotes paradoxus^2^* | Male | 6 | ALS | Piriform | 36 | Web | Orb web | Cribellate | 4 | 6 |
| *Hyptiotes paradoxus^2^* | Male | 6 | PMS | mAP | 1 | Web | Orb web | Cribellate | 4 | 6 |
| *Hyptiotes paradoxus^2^* | Male | 6 | PMS | Aciniform | 19 | Web | Orb web | Cribellate | 4 | 6 |
| *Hyptiotes paradoxus^2^* | Male | 6 | PMS | Paracribellar | 0 | Web | Orb web | Cribellate | 4 | 6 |
| *Hyptiotes paradoxus^2^* | Male | 6 | PMS | Cylindrical | 0 | Web | Orb web | Cribellate | 4 | 6 |
| *Hyptiotes paradoxus^2^* | Male | 6 | PLS | Aciniform | 44 | Web | Orb web | Cribellate | 4 | 6 |
| *Hyptiotes paradoxus^2^* | Male | 6 | PLS | Pseudoflagelliform | 0 | Web | Orb web | Cribellate | 4 | 6 |
| *Hyptiotes paradoxus^2^* | Male | 6 | PLS | Cylindrical | 0 | Web | Orb web | Cribellate | 4 | 6 |
| *Metellina segmentata^1^* | NA | 2 | Cribellum | Cribellar | NA | Web | Orb web | Viscous | 3 | 5 |
| *Metellina segmentata^1^* | NA | 2 | ALS | MAP | 1 | Web | Orb web | Viscous | 3 | 5 |
| *Metellina segmentata^1^* | NA | 2 | ALS | Piriform | 25 | Web | Orb web | Viscous | 3 | 5 |
| *Metellina segmentata^1^* | NA | 2 | PMS | mAP | 1 | Web | Orb web | Viscous | 3 | 5 |
| *Metellina segmentata^1^* | NA | 2 | PMS | Aciniform | 3 | Web | Orb web | Viscous | 3 | 5 |
| *Metellina segmentata^1^* | NA | 2 | PMS | Cylindrical | 0 | Web | Orb web | Viscous | 3 | 5 |
| *Metellina segmentata^1^* | NA | 2 | PLS | Aciniform | 12 | Web | Orb web | Viscous | 3 | 5 |
| *Metellina segmentata^1^* | NA | 2 | PLS | Aggregate | 2 | Web | Orb web | Viscous | 3 | 5 |
| *Metellina segmentata^1^* | NA | 2 | PLS | Flagelliform | 1 | Web | Orb web | Viscous | 3 | 5 |
| *Metellina segmentata^1^* | NA | 2 | PLS | Cylindrical | 0 | Web | Orb web | Viscous | 3 | 5 |
| *Metellina segmentata^1^* | NA | 3 | Cribellum | Cribellar | NA | Web | Orb web | Viscous | 3 | 5 |
| *Metellina segmentata^1^* | NA | 3 | ALS | MAP | 1 | Web | Orb web | Viscous | 3 | 5 |
| *Metellina segmentata^1^* | NA | 3 | ALS | Piriform | 54 | Web | Orb web | Viscous | 3 | 5 |
| *Metellina segmentata^1^* | NA | 3 | PMS | mAP | 1 | Web | Orb web | Viscous | 3 | 5 |
| *Metellina segmentata^1^* | NA | 3 | PMS | Aciniform | 3 | Web | Orb web | Viscous | 3 | 5 |
| *Metellina segmentata^1^* | NA | 3 | PMS | Cylindrical | 0 | Web | Orb web | Viscous | 3 | 5 |
| *Metellina segmentata^1^* | NA | 3 | PLS | Aciniform | 19 | Web | Orb web | Viscous | 3 | 5 |
| *Metellina segmentata^1^* | NA | 3 | PLS | Aggregate | 2 | Web | Orb web | Viscous | 3 | 5 |
| *Metellina segmentata^1^* | NA | 3 | PLS | Flagelliform | 1 | Web | Orb web | Viscous | 3 | 5 |
| *Metellina segmentata^1^* | NA | 3 | PLS | Cylindrical | 0 | Web | Orb web | Viscous | 3 | 5 |
| *Metellina segmentata^1^* | NA | 4 | Cribellum | Cribellar | NA | Web | Orb web | Viscous | 3 | 5 |
| *Metellina segmentata^1^* | NA | 4 | ALS | MAP | 1 | Web | Orb web | Viscous | 3 | 5 |
| *Metellina segmentata^1^* | NA | 4 | ALS | Piriform | 69 | Web | Orb web | Viscous | 3 | 5 |
| *Metellina segmentata^1^* | NA | 4 | PMS | mAP | 1 | Web | Orb web | Viscous | 3 | 5 |
| *Metellina segmentata^1^* | NA | 4 | PMS | Aciniform | 3 | Web | Orb web | Viscous | 3 | 5 |
| *Metellina segmentata^1^* | NA | 4 | PMS | Cylindrical | 0 | Web | Orb web | Viscous | 3 | 5 |
| *Metellina segmentata^1^* | NA | 4 | PLS | Aciniform | 21 | Web | Orb web | Viscous | 3 | 5 |
| *Metellina segmentata^1^* | NA | 4 | PLS | Aggregate | 2 | Web | Orb web | Viscous | 3 | 5 |
| *Metellina segmentata^1^* | NA | 4 | PLS | Flagelliform | 1 | Web | Orb web | Viscous | 3 | 5 |
| *Metellina segmentata^1^* | NA | 4 | PLS | Cylindrical | 0 | Web | Orb web | Viscous | 3 | 5 |
| *Metellina segmentata^1^* | Male | 5 | Cribellum | Cribellar | NA | Web | Orb web | Viscous | 3 | 5 |
| *Metellina segmentata^1^* | Male | 5 | ALS | MAP | 1 | Web | Orb web | Viscous | 3 | 5 |
| *Metellina segmentata^1^* | Male | 5 | ALS | Piriform | 71 | Web | Orb web | Viscous | 3 | 5 |
| *Metellina segmentata^1^* | Male | 5 | PMS | mAP | 1 | Web | Orb web | Viscous | 3 | 5 |
| *Metellina segmentata^1^* | Male | 5 | PMS | Aciniform | 3 | Web | Orb web | Viscous | 3 | 5 |
| *Metellina segmentata^1^* | Male | 5 | PMS | Cylindrical | 0 | Web | Orb web | Viscous | 3 | 5 |
| *Metellina segmentata^1^* | Male | 5 | PLS | Aciniform | 20 | Web | Orb web | Viscous | 3 | 5 |
| *Metellina segmentata^1^* | Male | 5 | PLS | Aggregate | 0 | Web | Orb web | Viscous | 3 | 5 |
| *Metellina segmentata^1^* | Male | 5 | PLS | Flagelliform | 0 | Web | Orb web | Viscous | 3 | 5 |
| *Metellina segmentata^1^* | Male | 5 | PLS | Cylindrical | 0 | Web | Orb web | Viscous | 3 | 5 |
| *Metellina segmentata^1^* | Female | 5 | Cribellum | Cribellar | NA | Web | Orb web | Viscous | 3 | 5 |
| *Metellina segmentata^1^* | Female | 5 | ALS | MAP | 1 | Web | Orb web | Viscous | 3 | 5 |
| *Metellina segmentata^1^* | Female | 5 | ALS | Piriform | 78 | Web | Orb web | Viscous | 3 | 5 |
| *Metellina segmentata^1^* | Female | 5 | PMS | mAP | 1 | Web | Orb web | Viscous | 3 | 5 |
| *Metellina segmentata^1^* | Female | 5 | PMS | Aciniform | 4 | Web | Orb web | Viscous | 3 | 5 |
| *Metellina segmentata^1^* | Female | 5 | PMS | Cylindrical | 1 | Web | Orb web | Viscous | 3 | 5 |
| *Metellina segmentata^1^* | Female | 5 | PLS | Aciniform | 23 | Web | Orb web | Viscous | 3 | 5 |
| *Metellina segmentata^1^* | Female | 5 | PLS | Aggregate | 2 | Web | Orb web | Viscous | 3 | 5 |
| *Metellina segmentata^1^* | Female | 5 | PLS | Flagelliform | 1 | Web | Orb web | Viscous | 3 | 5 |
| *Metellina segmentata^1^* | Female | 5 | PLS | Cylindrical | 2 | Web | Orb web | Viscous | 3 | 5 |
| *Mimetus puritanus^6^* | NA | 2 | Cribellum | Cribellar | NA | No web | Stalking | No | 1.5 | 7 |
| *Mimetus puritanus^6^* | NA | 2 | ALS | MAP | 2 | No web | Stalking | No | 1.5 | 7 |
| *Mimetus puritanus^6^* | NA | 2 | ALS | Modified Pi | 0 | No web | Stalking | No | 1.5 | 7 |
| *Mimetus puritanus^6^* | NA | 2 | ALS | Piriform | 4 | No web | Stalking | No | 1.5 | 7 |
| *Mimetus puritanus^6^* | NA | 2 | PMS | mAP | 2 | No web | Stalking | No | 1.5 | 7 |
| *Mimetus puritanus^6^* | NA | 2 | PMS | Aciniform | 2 | No web | Stalking | No | 1.5 | 7 |
| *Mimetus puritanus^6^* | NA | 2 | PMS | Cylindrical | 0 | No web | Stalking | No | 1.5 | 7 |
| *Mimetus puritanus^6^* | NA | 2 | PLS | Aciniform | 3 | No web | Stalking | No | 1.5 | 7 |
| *Mimetus puritanus^6^* | NA | 2 | PLS | Flagelliform | 0 | No web | Stalking | No | 1.5 | 7 |
| *Mimetus puritanus^6^* | NA | 2 | PLS | Aggregate | 0 | No web | Stalking | No | 1.5 | 7 |
| *Mimetus puritanus^6^* | NA | 2 | PLS | Cylindrical | 0 | No web | Stalking | No | 1.5 | 7 |
| *Mimetus puritanus^6^* | NA | 3 | Cribellum | Cribellar | NA | No web | Stalking | No | 1.5 | 7 |
| *Mimetus puritanus^6^* | NA | 3 | ALS | MAP | 2 | No web | Stalking | No | 1.5 | 7 |
| *Mimetus puritanus^6^* | NA | 3 | ALS | Modified Pi | 0 | No web | Stalking | No | 1.5 | 7 |
| *Mimetus puritanus^6^* | NA | 3 | ALS | Piriform | 7 | No web | Stalking | No | 1.5 | 7 |
| *Mimetus puritanus^6^* | NA | 3 | PMS | mAP | 2 | No web | Stalking | No | 1.5 | 7 |
| *Mimetus puritanus^6^* | NA | 3 | PMS | Aciniform | 2 | No web | Stalking | No | 1.5 | 7 |
| *Mimetus puritanus^6^* | NA | 3 | PMS | Cylindrical | 0 | No web | Stalking | No | 1.5 | 7 |
| *Mimetus puritanus^6^* | NA | 3 | PLS | Aciniform | 5 | No web | Stalking | No | 1.5 | 7 |
| *Mimetus puritanus^6^* | NA | 3 | PLS | Flagelliform | 0 | No web | Stalking | No | 1.5 | 7 |
| *Mimetus puritanus^6^* | NA | 3 | PLS | Aggregate | 0 | No web | Stalking | No | 1.5 | 7 |
| *Mimetus puritanus^6^* | NA | 3 | PLS | Cylindrical | 0 | No web | Stalking | No | 1.5 | 7 |
| *Mimetus puritanus^6^* | Juve Male | 4 | Cribellum | Cribellar | NA | No web | Stalking | No | 1.5 | 7 |
| *Mimetus puritanus^6^* | Juve Male | 4 | ALS | MAP | 2 | No web | Stalking | No | 1.5 | 7 |
| *Mimetus puritanus^6^* | Juve Male | 4 | ALS | Modified Pi | 0 | No web | Stalking | No | 1.5 | 7 |
| *Mimetus puritanus^6^* | Juve Male | 4 | ALS | Piriform | 12 | No web | Stalking | No | 1.5 | 7 |
| *Mimetus puritanus^6^* | Juve Male | 4 | PMS | mAP | 2 | No web | Stalking | No | 1.5 | 7 |
| *Mimetus puritanus^6^* | Juve Male | 4 | PMS | Aciniform | 3 | No web | Stalking | No | 1.5 | 7 |
| *Mimetus puritanus^6^* | Juve Male | 4 | PMS | Cylindrical | 0 | No web | Stalking | No | 1.5 | 7 |
| *Mimetus puritanus^6^* | Juve Male | 4 | PLS | Aciniform | 7 | No web | Stalking | No | 1.5 | 7 |
| *Mimetus puritanus^6^* | Juve Male | 4 | PLS | Flagelliform | 0 | No web | Stalking | No | 1.5 | 7 |
| *Mimetus puritanus^6^* | Juve Male | 4 | PLS | Aggregate | 0 | No web | Stalking | No | 1.5 | 7 |
| *Mimetus puritanus^6^* | Juve Male | 4 | PLS | Cylindrical | 0 | No web | Stalking | No | 1.5 | 7 |
| *Mimetus puritanus^6^* | Juve Fem | 4 | Cribellum | Cribellar | NA | No web | Stalking | No | 1.5 | 7 |
| *Mimetus puritanus^6^* | Juve Fem | 4 | ALS | MAP | 2 | No web | Stalking | No | 1.5 | 7 |
| *Mimetus puritanus^6^* | Juve Fem | 4 | ALS | Modified Pi | 0 | No web | Stalking | No | 1.5 | 7 |
| *Mimetus puritanus^6^* | Juve Fem | 4 | ALS | Piriform | 12 | No web | Stalking | No | 1.5 | 7 |
| *Mimetus puritanus^6^* | Juve Fem | 4 | PMS | mAP | 2 | No web | Stalking | No | 1.5 | 7 |
| *Mimetus puritanus^6^* | Juve Fem | 4 | PMS | Aciniform | 3 | No web | Stalking | No | 1.5 | 7 |
| *Mimetus puritanus^6^* | Juve Fem | 4 | PMS | Cylindrical | 1 | No web | Stalking | No | 1.5 | 7 |
| *Mimetus puritanus^6^* | Juve Fem | 4 | PLS | Aciniform | 7 | No web | Stalking | No | 1.5 | 7 |
| *Mimetus puritanus^6^* | Juve Fem | 4 | PLS | Flagelliform | 0 | No web | Stalking | No | 1.5 | 7 |
| *Mimetus puritanus^6^* | Juve Fem | 4 | PLS | Aggregate | 0 | No web | Stalking | No | 1.5 | 7 |
| *Mimetus puritanus^6^* | Juve Fem | 4 | PLS | Cylindrical | 1 | No web | Stalking | No | 1.5 | 7 |
| *Mimetus puritanus^6^* | Pen Male | 5 | Cribellum | Cribellar | NA | No web | Stalking | No | 1.5 | 7 |
| *Mimetus puritanus^6^* | Pen Male | 5 | ALS | MAP | 2 | No web | Stalking | No | 1.5 | 7 |
| *Mimetus puritanus^6^* | Pen Male | 5 | ALS | Modified Pi | 0 | No web | Stalking | No | 1.5 | 7 |
| *Mimetus puritanus^6^* | Pen Male | 5 | ALS | Piriform | 20 | No web | Stalking | No | 1.5 | 7 |
| *Mimetus puritanus^6^* | Pen Male | 5 | PMS | mAP | 2 | No web | Stalking | No | 1.5 | 7 |
| *Mimetus puritanus^6^* | Pen Male | 5 | PMS | Aciniform | 4 | No web | Stalking | No | 1.5 | 7 |
| *Mimetus puritanus^6^* | Pen Male | 5 | PMS | Cylindrical | 0 | No web | Stalking | No | 1.5 | 7 |
| *Mimetus puritanus^6^* | Pen Male | 5 | PLS | Aciniform | 10 | No web | Stalking | No | 1.5 | 7 |
| *Mimetus puritanus^6^* | Pen Male | 5 | PLS | Flagelliform | 0 | No web | Stalking | No | 1.5 | 7 |
| *Mimetus puritanus^6^* | Pen Male | 5 | PLS | Aggregate | 0 | No web | Stalking | No | 1.5 | 7 |
| *Mimetus puritanus^6^* | Pen Male | 5 | PLS | Cylindrical | 0 | No web | Stalking | No | 1.5 | 7 |
| *Mimetus puritanus^6^* | Pen Fem | 5 | Cribellum | Cribellar | NA | No web | Stalking | No | 1.5 | 7 |
| *Mimetus puritanus^6^* | Pen Fem | 5 | ALS | MAP | 2 | No web | Stalking | No | 1.5 | 7 |
| *Mimetus puritanus^6^* | Pen Fem | 5 | ALS | Modified Pi | 0 | No web | Stalking | No | 1.5 | 7 |
| *Mimetus puritanus^6^* | Pen Fem | 5 | ALS | Piriform | 21 | No web | Stalking | No | 1.5 | 7 |
| *Mimetus puritanus^6^* | Pen Fem | 5 | PMS | mAP | 2 | No web | Stalking | No | 1.5 | 7 |
| *Mimetus puritanus^6^* | Pen Fem | 5 | PMS | Aciniform | 3 | No web | Stalking | No | 1.5 | 7 |
| *Mimetus puritanus^6^* | Pen Fem | 5 | PMS | Cylindrical | 1 | No web | Stalking | No | 1.5 | 7 |
| *Mimetus puritanus^6^* | Pen Fem | 5 | PLS | Aciniform | 10 | No web | Stalking | No | 1.5 | 7 |
| *Mimetus puritanus^6^* | Pen Fem | 5 | PLS | Flagelliform | 0 | No web | Stalking | No | 1.5 | 7 |
| *Mimetus puritanus^6^* | Pen Fem | 5 | PLS | Aggregate | 0 | No web | Stalking | No | 1.5 | 7 |
| *Mimetus puritanus^6^* | Pen Fem | 5 | PLS | Cylindrical | 1 | No web | Stalking | No | 1.5 | 7 |
| *Mimetus puritanus^6^* | Pen Male | 6 | Cribellum | Cribellar | NA | No web | Stalking | No | 1.5 | 7 |
| *Mimetus puritanus^6^* | Pen Male | 6 | ALS | MAP | 2 | No web | Stalking | No | 1.5 | 7 |
| *Mimetus puritanus^6^* | Pen Male | 6 | ALS | Modified Pi | 0 | No web | Stalking | No | 1.5 | 7 |
| *Mimetus puritanus^6^* | Pen Male | 6 | ALS | Piriform | 30 | No web | Stalking | No | 1.5 | 7 |
| *Mimetus puritanus^6^* | Pen Male | 6 | PMS | mAP | 2 | No web | Stalking | No | 1.5 | 7 |
| *Mimetus puritanus^6^* | Pen Male | 6 | PMS | Aciniform | 4 | No web | Stalking | No | 1.5 | 7 |
| *Mimetus puritanus^6^* | Pen Male | 6 | PMS | Cylindrical | 0 | No web | Stalking | No | 1.5 | 7 |
| *Mimetus puritanus^6^* | Pen Male | 6 | PLS | Aciniform | 12 | No web | Stalking | No | 1.5 | 7 |
| *Mimetus puritanus^6^* | Pen Male | 6 | PLS | Flagelliform | 0 | No web | Stalking | No | 1.5 | 7 |
| *Mimetus puritanus^6^* | Pen Male | 6 | PLS | Aggregate | 0 | No web | Stalking | No | 1.5 | 7 |
| *Mimetus puritanus^6^* | Pen Male | 6 | PLS | Cylindrical | 0 | No web | Stalking | No | 1.5 | 7 |
| *Mimetus puritanus^6^* | Pen Fem | 6 | Cribellum | Cribellar | NA | No web | Stalking | No | 1.5 | 7 |
| *Mimetus puritanus^6^* | Pen Fem | 6 | ALS | MAP | 2 | No web | Stalking | No | 1.5 | 7 |
| *Mimetus puritanus^6^* | Pen Fem | 6 | ALS | Modified Pi | 0 | No web | Stalking | No | 1.5 | 7 |
| *Mimetus puritanus^6^* | Pen Fem | 6 | ALS | Piriform | 31 | No web | Stalking | No | 1.5 | 7 |
| *Mimetus puritanus^6^* | Pen Fem | 6 | PMS | mAP | 2 | No web | Stalking | No | 1.5 | 7 |
| *Mimetus puritanus^6^* | Pen Fem | 6 | PMS | Aciniform | 3 | No web | Stalking | No | 1.5 | 7 |
| *Mimetus puritanus^6^* | Pen Fem | 6 | PMS | Cylindrical | 1 | No web | Stalking | No | 1.5 | 7 |
| *Mimetus puritanus^6^* | Pen Fem | 6 | PLS | Aciniform | 12 | No web | Stalking | No | 1.5 | 7 |
| *Mimetus puritanus^6^* | Pen Fem | 6 | PLS | Flagelliform | 0 | No web | Stalking | No | 1.5 | 7 |
| *Mimetus puritanus^6^* | Pen Fem | 6 | PLS | Aggregate | 0 | No web | Stalking | No | 1.5 | 7 |
| *Mimetus puritanus^6^* | Pen Fem | 6 | PLS | Cylindrical | 1 | No web | Stalking | No | 1.5 | 7 |
| *Mimetus puritanus^6^* | Male | 6 | Cribellum | Cribellar | NA | No web | Stalking | No | 1.5 | 7 |
| *Mimetus puritanus^6^* | Male | 6 | ALS | MAP | 1 | No web | Stalking | No | 1.5 | 7 |
| *Mimetus puritanus^6^* | Male | 6 | ALS | Modified Pi | 2 | No web | Stalking | No | 1.5 | 7 |
| *Mimetus puritanus^6^* | Male | 6 | ALS | Piriform | 38 | No web | Stalking | No | 1.5 | 7 |
| *Mimetus puritanus^6^* | Male | 6 | PMS | mAP | 1 | No web | Stalking | No | 1.5 | 7 |
| *Mimetus puritanus^6^* | Male | 6 | PMS | Aciniform | 4 | No web | Stalking | No | 1.5 | 7 |
| *Mimetus puritanus^6^* | Male | 6 | PMS | Cylindrical | 0 | No web | Stalking | No | 1.5 | 7 |
| *Mimetus puritanus^6^* | Male | 6 | PLS | Aciniform | 12 | No web | Stalking | No | 1.5 | 7 |
| *Mimetus puritanus^6^* | Male | 6 | PLS | Flagelliform | 0 | No web | Stalking | No | 1.5 | 7 |
| *Mimetus puritanus^6^* | Male | 6 | PLS | Aggregate | 0 | No web | Stalking | No | 1.5 | 7 |
| *Mimetus puritanus^6^* | Male | 6 | PLS | Cylindrical | 0 | No web | Stalking | No | 1.5 | 7 |
| *Mimetus puritanus^6^* | Female | 6 | Cribellum | Cribellar | NA | No web | Stalking | No | 1.5 | 7 |
| *Mimetus puritanus^6^* | Female | 6 | ALS | MAP | 1 | No web | Stalking | No | 1.5 | 7 |
| *Mimetus puritanus^6^* | Female | 6 | ALS | Modified Pi | 0 | No web | Stalking | No | 1.5 | 7 |
| *Mimetus puritanus^6^* | Female | 6 | ALS | Piriform | 40 | No web | Stalking | No | 1.5 | 7 |
| *Mimetus puritanus^6^* | Female | 6 | PMS | mAP | 1 | No web | Stalking | No | 1.5 | 7 |
| *Mimetus puritanus^6^* | Female | 6 | PMS | Aciniform | 4 | No web | Stalking | No | 1.5 | 7 |
| *Mimetus puritanus^6^* | Female | 6 | PMS | Cylindrical | 1 | No web | Stalking | No | 1.5 | 7 |
| *Mimetus puritanus^6^* | Female | 6 | PLS | Aciniform | 12 | No web | Stalking | No | 1.5 | 7 |
| *Mimetus puritanus^6^* | Female | 6 | PLS | Flagelliform | 0 | No web | Stalking | No | 1.5 | 7 |
| *Mimetus puritanus^6^* | Female | 6 | PLS | Aggregate | 0 | No web | Stalking | No | 1.5 | 7 |
| *Mimetus puritanus^6^* | Female | 6 | PLS | Cylindrical | 1 | No web | Stalking | No | 1.5 | 7 |
| *Mimetus puritanus^6^* | Male | 7 | Cribellum | Cribellar | NA | No web | Stalking | No | 1.5 | 7 |
| *Mimetus puritanus^6^* | Male | 7 | ALS | MAP | 1 | No web | Stalking | No | 1.5 | 7 |
| *Mimetus puritanus^6^* | Male | 7 | ALS | Modified Pi | 2 | No web | Stalking | No | 1.5 | 7 |
| *Mimetus puritanus^6^* | Male | 7 | ALS | Piriform | 48 | No web | Stalking | No | 1.5 | 7 |
| *Mimetus puritanus^6^* | Male | 7 | PMS | mAP | 1 | No web | Stalking | No | 1.5 | 7 |
| *Mimetus puritanus^6^* | Male | 7 | PMS | Aciniform | 4 | No web | Stalking | No | 1.5 | 7 |
| *Mimetus puritanus^6^* | Male | 7 | PMS | Cylindrical | 0 | No web | Stalking | No | 1.5 | 7 |
| *Mimetus puritanus^6^* | Male | 7 | PLS | Aciniform | 12 | No web | Stalking | No | 1.5 | 7 |
| *Mimetus puritanus^6^* | Male | 7 | PLS | Flagelliform | 0 | No web | Stalking | No | 1.5 | 7 |
| *Mimetus puritanus^6^* | Male | 7 | PLS | Aggregate | 0 | No web | Stalking | No | 1.5 | 7 |
| *Mimetus puritanus^6^* | Male | 7 | PLS | Cylindrical | 0 | No web | Stalking | No | 1.5 | 7 |
| *Mimetus puritanus^6^* | Female | 7 | Cribellum | Cribellar | NA | No web | Stalking | No | 1.5 | 7 |
| *Mimetus puritanus^6^* | Female | 7 | ALS | MAP | 1 | No web | Stalking | No | 1.5 | 7 |
| *Mimetus puritanus^6^* | Female | 7 | ALS | Modified Pi | 0 | No web | Stalking | No | 1.5 | 7 |
| *Mimetus puritanus^6^* | Female | 7 | ALS | Piriform | 55 | No web | Stalking | No | 1.5 | 7 |
| *Mimetus puritanus^6^* | Female | 7 | PMS | mAP | 1 | No web | Stalking | No | 1.5 | 7 |
| *Mimetus puritanus^6^* | Female | 7 | PMS | Aciniform | 4 | No web | Stalking | No | 1.5 | 7 |
| *Mimetus puritanus^6^* | Female | 7 | PMS | Cylindrical | 1 | No web | Stalking | No | 1.5 | 7 |
| *Mimetus puritanus^6^* | Female | 7 | PLS | Aciniform | 15 | No web | Stalking | No | 1.5 | 7 |
| *Mimetus puritanus^6^* | Female | 7 | PLS | Flagelliform | 0 | No web | Stalking | No | 1.5 | 7 |
| *Mimetus puritanus^6^* | Female | 7 | PLS | Aggregate | 0 | No web | Stalking | No | 1.5 | 7 |
| *Mimetus puritanus^6^* | Female | 7 | PLS | Cylindrical | 1 | No web | Stalking | No | 1.5 | 7 |
| *Mimetus notius^6^* | NA | 2 | Cribellum | Cribellar | NA | No web | Stalking | No | 1.5 | 7 |
| *Mimetus notius^6^* | NA | 2 | ALS | MAP | 2 | No web | Stalking | No | 1.5 | 7 |
| *Mimetus notius^6^* | NA | 2 | ALS | Modified Pi | 0 | No web | Stalking | No | 1.5 | 7 |
| *Mimetus notius^6^* | NA | 2 | ALS | Piriform | 4 | No web | Stalking | No | 1.5 | 7 |
| *Mimetus notius^6^* | NA | 2 | PMS | mAP | 2 | No web | Stalking | No | 1.5 | 7 |
| *Mimetus notius^6^* | NA | 2 | PMS | Aciniform | 2 | No web | Stalking | No | 1.5 | 7 |
| *Mimetus notius^6^* | NA | 2 | PMS | Cylindrical | 0 | No web | Stalking | No | 1.5 | 7 |
| *Mimetus notius^6^* | NA | 2 | PLS | Aciniform | 3 | No web | Stalking | No | 1.5 | 7 |
| *Mimetus notius^6^* | NA | 2 | PLS | Flagelliform | 0 | No web | Stalking | No | 1.5 | 7 |
| *Mimetus notius^6^* | NA | 2 | PLS | Aggregate | 0 | No web | Stalking | No | 1.5 | 7 |
| *Mimetus notius^6^* | NA | 2 | PLS | Cylindrical | 0 | No web | Stalking | No | 1.5 | 7 |
| *Mimetus notius^6^* | NA | 3 | Cribellum | Cribellar | NA | No web | Stalking | No | 1.5 | 7 |
| *Mimetus notius^6^* | NA | 3 | ALS | MAP | 2 | No web | Stalking | No | 1.5 | 7 |
| *Mimetus notius^6^* | NA | 3 | ALS | Modified Pi | 0 | No web | Stalking | No | 1.5 | 7 |
| *Mimetus notius^6^* | NA | 3 | ALS | Piriform | 8 | No web | Stalking | No | 1.5 | 7 |
| *Mimetus notius^6^* | NA | 3 | PMS | mAP | 2 | No web | Stalking | No | 1.5 | 7 |
| *Mimetus notius^6^* | NA | 3 | PMS | Aciniform | 2 | No web | Stalking | No | 1.5 | 7 |
| *Mimetus notius^6^* | NA | 3 | PMS | Cylindrical | 0 | No web | Stalking | No | 1.5 | 7 |
| *Mimetus notius^6^* | NA | 3 | PLS | Aciniform | 5 | No web | Stalking | No | 1.5 | 7 |
| *Mimetus notius^6^* | NA | 3 | PLS | Flagelliform | 0 | No web | Stalking | No | 1.5 | 7 |
| *Mimetus notius^6^* | NA | 3 | PLS | Aggregate | 0 | No web | Stalking | No | 1.5 | 7 |
| *Mimetus notius^6^* | NA | 3 | PLS | Cylindrical | 0 | No web | Stalking | No | 1.5 | 7 |
| *Mimetus notius^6^* | Juve Male | 4 | Cribellum | Cribellar | NA | No web | Stalking | No | 1.5 | 7 |
| *Mimetus notius^6^* | Juve Male | 4 | ALS | MAP | 2 | No web | Stalking | No | 1.5 | 7 |
| *Mimetus notius^6^* | Juve Male | 4 | ALS | Modified Pi | 0 | No web | Stalking | No | 1.5 | 7 |
| *Mimetus notius^6^* | Juve Male | 4 | ALS | Piriform | 14 | No web | Stalking | No | 1.5 | 7 |
| *Mimetus notius^6^* | Juve Male | 4 | PMS | mAP | 2 | No web | Stalking | No | 1.5 | 7 |
| *Mimetus notius^6^* | Juve Male | 4 | PMS | Aciniform | 3 | No web | Stalking | No | 1.5 | 7 |
| *Mimetus notius^6^* | Juve Male | 4 | PMS | Cylindrical | 0 | No web | Stalking | No | 1.5 | 7 |
| *Mimetus notius^6^* | Juve Male | 4 | PLS | Aciniform | 7 | No web | Stalking | No | 1.5 | 7 |
| *Mimetus notius^6^* | Juve Male | 4 | PLS | Flagelliform | 0 | No web | Stalking | No | 1.5 | 7 |
| *Mimetus notius^6^* | Juve Male | 4 | PLS | Aggregate | 0 | No web | Stalking | No | 1.5 | 7 |
| *Mimetus notius^6^* | Juve Male | 4 | PLS | Cylindrical | 0 | No web | Stalking | No | 1.5 | 7 |
| *Mimetus notius^6^* | Juve Fem | 4 | Cribellum | Cribellar | NA | No web | Stalking | No | 1.5 | 7 |
| *Mimetus notius^6^* | Juve Fem | 4 | ALS | MAP | 2 | No web | Stalking | No | 1.5 | 7 |
| *Mimetus notius^6^* | Juve Fem | 4 | ALS | Modified Pi | 0 | No web | Stalking | No | 1.5 | 7 |
| *Mimetus notius^6^* | Juve Fem | 4 | ALS | Piriform | 15 | No web | Stalking | No | 1.5 | 7 |
| *Mimetus notius^6^* | Juve Fem | 4 | PMS | mAP | 2 | No web | Stalking | No | 1.5 | 7 |
| *Mimetus notius^6^* | Juve Fem | 4 | PMS | Aciniform | 3 | No web | Stalking | No | 1.5 | 7 |
| *Mimetus notius^6^* | Juve Fem | 4 | PMS | Cylindrical | 1 | No web | Stalking | No | 1.5 | 7 |
| *Mimetus notius^6^* | Juve Fem | 4 | PLS | Aciniform | 8 | No web | Stalking | No | 1.5 | 7 |
| *Mimetus notius^6^* | Juve Fem | 4 | PLS | Flagelliform | 0 | No web | Stalking | No | 1.5 | 7 |
| *Mimetus notius^6^* | Juve Fem | 4 | PLS | Aggregate | 0 | No web | Stalking | No | 1.5 | 7 |
| *Mimetus notius^6^* | Juve Fem | 4 | PLS | Cylindrical | 1 | No web | Stalking | No | 1.5 | 7 |
| *Mimetus notius^6^* | Pen Male | 5 | Cribellum | Cribellar | NA | No web | Stalking | No | 1.5 | 7 |
| *Mimetus notius^6^* | Pen Male | 5 | ALS | MAP | 2 | No web | Stalking | No | 1.5 | 7 |
| *Mimetus notius^6^* | Pen Male | 5 | ALS | Modified Pi | 0 | No web | Stalking | No | 1.5 | 7 |
| *Mimetus notius^6^* | Pen Male | 5 | ALS | Piriform | 20 | No web | Stalking | No | 1.5 | 7 |
| *Mimetus notius^6^* | Pen Male | 5 | PMS | mAP | 2 | No web | Stalking | No | 1.5 | 7 |
| *Mimetus notius^6^* | Pen Male | 5 | PMS | Aciniform | 4 | No web | Stalking | No | 1.5 | 7 |
| *Mimetus notius^6^* | Pen Male | 5 | PMS | Cylindrical | 0 | No web | Stalking | No | 1.5 | 7 |
| *Mimetus notius^6^* | Pen Male | 5 | PLS | Aciniform | 10 | No web | Stalking | No | 1.5 | 7 |
| *Mimetus notius^6^* | Pen Male | 5 | PLS | Flagelliform | 0 | No web | Stalking | No | 1.5 | 7 |
| *Mimetus notius^6^* | Pen Male | 5 | PLS | Aggregate | 0 | No web | Stalking | No | 1.5 | 7 |
| *Mimetus notius^6^* | Pen Male | 5 | PLS | Cylindrical | 0 | No web | Stalking | No | 1.5 | 7 |
| *Mimetus notius^6^* | Pen Fem | 5 | Cribellum | Cribellar | NA | No web | Stalking | No | 1.5 | 7 |
| *Mimetus notius^6^* | Pen Fem | 5 | ALS | MAP | 2 | No web | Stalking | No | 1.5 | 7 |
| *Mimetus notius^6^* | Pen Fem | 5 | ALS | Modified Pi | 0 | No web | Stalking | No | 1.5 | 7 |
| *Mimetus notius^6^* | Pen Fem | 5 | ALS | Piriform | 22 | No web | Stalking | No | 1.5 | 7 |
| *Mimetus notius^6^* | Pen Fem | 5 | PMS | mAP | 2 | No web | Stalking | No | 1.5 | 7 |
| *Mimetus notius^6^* | Pen Fem | 5 | PMS | Aciniform | 4 | No web | Stalking | No | 1.5 | 7 |
| *Mimetus notius^6^* | Pen Fem | 5 | PMS | Cylindrical | 1 | No web | Stalking | No | 1.5 | 7 |
| *Mimetus notius^6^* | Pen Fem | 5 | PLS | Aciniform | 11 | No web | Stalking | No | 1.5 | 7 |
| *Mimetus notius^6^* | Pen Fem | 5 | PLS | Flagelliform | 0 | No web | Stalking | No | 1.5 | 7 |
| *Mimetus notius^6^* | Pen Fem | 5 | PLS | Aggregate | 0 | No web | Stalking | No | 1.5 | 7 |
| *Mimetus notius^6^* | Pen Fem | 5 | PLS | Cylindrical | 1 | No web | Stalking | No | 1.5 | 7 |
| *Mimetus notius^6^* | Pen Male | 6 | Cribellum | Cribellar | NA | No web | Stalking | No | 1.5 | 7 |
| *Mimetus notius^6^* | Pen Male | 6 | ALS | MAP | 2 | No web | Stalking | No | 1.5 | 7 |
| *Mimetus notius^6^* | Pen Male | 6 | ALS | Modified Pi | 0 | No web | Stalking | No | 1.5 | 7 |
| *Mimetus notius^6^* | Pen Male | 6 | ALS | Piriform | 27 | No web | Stalking | No | 1.5 | 7 |
| *Mimetus notius^6^* | Pen Male | 6 | PMS | mAP | 2 | No web | Stalking | No | 1.5 | 7 |
| *Mimetus notius^6^* | Pen Male | 6 | PMS | Aciniform | 3 | No web | Stalking | No | 1.5 | 7 |
| *Mimetus notius^6^* | Pen Male | 6 | PMS | Cylindrical | 0 | No web | Stalking | No | 1.5 | 7 |
| *Mimetus notius^6^* | Pen Male | 6 | PLS | Aciniform | 13 | No web | Stalking | No | 1.5 | 7 |
| *Mimetus notius^6^* | Pen Male | 6 | PLS | Flagelliform | 0 | No web | Stalking | No | 1.5 | 7 |
| *Mimetus notius^6^* | Pen Male | 6 | PLS | Aggregate | 0 | No web | Stalking | No | 1.5 | 7 |
| *Mimetus notius^6^* | Pen Male | 6 | PLS | Cylindrical | 0 | No web | Stalking | No | 1.5 | 7 |
| *Mimetus notius^6^* | Pen Fem | 6 | Cribellum | Cribellar | NA | No web | Stalking | No | 1.5 | 7 |
| *Mimetus notius^6^* | Pen Fem | 6 | ALS | MAP | 2 | No web | Stalking | No | 1.5 | 7 |
| *Mimetus notius^6^* | Pen Fem | 6 | ALS | Modified Pi | 0 | No web | Stalking | No | 1.5 | 7 |
| *Mimetus notius^6^* | Pen Fem | 6 | ALS | Piriform | 29 | No web | Stalking | No | 1.5 | 7 |
| *Mimetus notius^6^* | Pen Fem | 6 | PMS | mAP | 2 | No web | Stalking | No | 1.5 | 7 |
| *Mimetus notius^6^* | Pen Fem | 6 | PMS | Aciniform | 4 | No web | Stalking | No | 1.5 | 7 |
| *Mimetus notius^6^* | Pen Fem | 6 | PMS | Cylindrical | 1 | No web | Stalking | No | 1.5 | 7 |
| *Mimetus notius^6^* | Pen Fem | 6 | PLS | Aciniform | 12 | No web | Stalking | No | 1.5 | 7 |
| *Mimetus notius^6^* | Pen Fem | 6 | PLS | Flagelliform | 0 | No web | Stalking | No | 1.5 | 7 |
| *Mimetus notius^6^* | Pen Fem | 6 | PLS | Aggregate | 0 | No web | Stalking | No | 1.5 | 7 |
| *Mimetus notius^6^* | Pen Fem | 6 | PLS | Cylindrical | 1 | No web | Stalking | No | 1.5 | 7 |
| *Mimetus notius^6^* | Male | 6 | Cribellum | Cribellar | NA | No web | Stalking | No | 1.5 | 7 |
| *Mimetus notius^6^* | Male | 6 | ALS | MAP | 1 | No web | Stalking | No | 1.5 | 7 |
| *Mimetus notius^6^* | Male | 6 | ALS | Modified Pi | 2 | No web | Stalking | No | 1.5 | 7 |
| *Mimetus notius^6^* | Male | 6 | ALS | Piriform | 34 | No web | Stalking | No | 1.5 | 7 |
| *Mimetus notius^6^* | Male | 6 | PMS | mAP | 1 | No web | Stalking | No | 1.5 | 7 |
| *Mimetus notius^6^* | Male | 6 | PMS | Aciniform | 4 | No web | Stalking | No | 1.5 | 7 |
| *Mimetus notius^6^* | Male | 6 | PMS | Cylindrical | 0 | No web | Stalking | No | 1.5 | 7 |
| *Mimetus notius^6^* | Male | 6 | PLS | Aciniform | 13 | No web | Stalking | No | 1.5 | 7 |
| *Mimetus notius^6^* | Male | 6 | PLS | Flagelliform | 0 | No web | Stalking | No | 1.5 | 7 |
| *Mimetus notius^6^* | Male | 6 | PLS | Aggregate | 0 | No web | Stalking | No | 1.5 | 7 |
| *Mimetus notius^6^* | Male | 6 | PLS | Cylindrical | 0 | No web | Stalking | No | 1.5 | 7 |
| *Mimetus notius^6^* | Female | 6 | Cribellum | Cribellar | NA | No web | Stalking | No | 1.5 | 7 |
| *Mimetus notius^6^* | Female | 6 | ALS | MAP | 1 | No web | Stalking | No | 1.5 | 7 |
| *Mimetus notius^6^* | Female | 6 | ALS | Modified Pi | 0 | No web | Stalking | No | 1.5 | 7 |
| *Mimetus notius^6^* | Female | 6 | ALS | Piriform | 38 | No web | Stalking | No | 1.5 | 7 |
| *Mimetus notius^6^* | Female | 6 | PMS | mAP | 1 | No web | Stalking | No | 1.5 | 7 |
| *Mimetus notius^6^* | Female | 6 | PMS | Aciniform | 4 | No web | Stalking | No | 1.5 | 7 |
| *Mimetus notius^6^* | Female | 6 | PMS | Cylindrical | 1 | No web | Stalking | No | 1.5 | 7 |
| *Mimetus notius^6^* | Female | 6 | PLS | Aciniform | 14 | No web | Stalking | No | 1.5 | 7 |
| *Mimetus notius^6^* | Female | 6 | PLS | Flagelliform | 0 | No web | Stalking | No | 1.5 | 7 |
| *Mimetus notius^6^* | Female | 6 | PLS | Aggregate | 0 | No web | Stalking | No | 1.5 | 7 |
| *Mimetus notius^6^* | Female | 6 | PLS | Cylindrical | 1 | No web | Stalking | No | 1.5 | 7 |
| *Mimetus notius^6^* | Pen Fem | 7 | Cribellum | Cribellar | NA | No web | Stalking | No | 1.5 | 7 |
| *Mimetus notius^6^* | Pen Fem | 7 | ALS | MAP | 2 | No web | Stalking | No | 1.5 | 7 |
| *Mimetus notius^6^* | Pen Fem | 7 | ALS | Modified Pi | 0 | No web | Stalking | No | 1.5 | 7 |
| *Mimetus notius^6^* | Pen Fem | 7 | ALS | Piriform | 43 | No web | Stalking | No | 1.5 | 7 |
| *Mimetus notius^6^* | Pen Fem | 7 | PMS | mAP | 2 | No web | Stalking | No | 1.5 | 7 |
| *Mimetus notius^6^* | Pen Fem | 7 | PMS | Aciniform | 4 | No web | Stalking | No | 1.5 | 7 |
| *Mimetus notius^6^* | Pen Fem | 7 | PMS | Cylindrical | 1 | No web | Stalking | No | 1.5 | 7 |
| *Mimetus notius^6^* | Pen Fem | 7 | PLS | Aciniform | 14 | No web | Stalking | No | 1.5 | 7 |
| *Mimetus notius^6^* | Pen Fem | 7 | PLS | Flagelliform | 0 | No web | Stalking | No | 1.5 | 7 |
| *Mimetus notius^6^* | Pen Fem | 7 | PLS | Aggregate | 0 | No web | Stalking | No | 1.5 | 7 |
| *Mimetus notius^6^* | Pen Fem | 7 | PLS | Cylindrical | 1 | No web | Stalking | No | 1.5 | 7 |
| *Mimetus notius^6^* | Male | 7 | Cribellum | Cribellar | NA | No web | Stalking | No | 1.5 | 7 |
| *Mimetus notius^6^* | Male | 7 | ALS | MAP | 1 | No web | Stalking | No | 1.5 | 7 |
| *Mimetus notius^6^* | Male | 7 | ALS | Modified Pi | 2 | No web | Stalking | No | 1.5 | 7 |
| *Mimetus notius^6^* | Male | 7 | ALS | Piriform | 36 | No web | Stalking | No | 1.5 | 7 |
| *Mimetus notius^6^* | Male | 7 | PMS | mAP | 1 | No web | Stalking | No | 1.5 | 7 |
| *Mimetus notius^6^* | Male | 7 | PMS | Aciniform | 3 | No web | Stalking | No | 1.5 | 7 |
| *Mimetus notius^6^* | Male | 7 | PMS | Cylindrical | 0 | No web | Stalking | No | 1.5 | 7 |
| *Mimetus notius^6^* | Male | 7 | PLS | Aciniform | 15 | No web | Stalking | No | 1.5 | 7 |
| *Mimetus notius^6^* | Male | 7 | PLS | Flagelliform | 0 | No web | Stalking | No | 1.5 | 7 |
| *Mimetus notius^6^* | Male | 7 | PLS | Aggregate | 0 | No web | Stalking | No | 1.5 | 7 |
| *Mimetus notius^6^* | Male | 7 | PLS | Cylindrical | 0 | No web | Stalking | No | 1.5 | 7 |
| *Mimetus notius^6^* | Female | 7 | Cribellum | Cribellar | NA | No web | Stalking | No | 1.5 | 7 |
| *Mimetus notius^6^* | Female | 7 | ALS | MAP | 1 | No web | Stalking | No | 1.5 | 7 |
| *Mimetus notius^6^* | Female | 7 | ALS | Modified Pi | 0 | No web | Stalking | No | 1.5 | 7 |
| *Mimetus notius^6^* | Female | 7 | ALS | Piriform | 47 | No web | Stalking | No | 1.5 | 7 |
| *Mimetus notius^6^* | Female | 7 | PMS | mAP | 1 | No web | Stalking | No | 1.5 | 7 |
| *Mimetus notius^6^* | Female | 7 | PMS | Aciniform | 4 | No web | Stalking | No | 1.5 | 7 |
| *Mimetus notius^6^* | Female | 7 | PMS | Cylindrical | 1 | No web | Stalking | No | 1.5 | 7 |
| *Mimetus notius^6^* | Female | 7 | PLS | Aciniform | 15 | No web | Stalking | No | 1.5 | 7 |
| *Mimetus notius^6^* | Female | 7 | PLS | Flagelliform | 0 | No web | Stalking | No | 1.5 | 7 |
| *Mimetus notius^6^* | Female | 7 | PLS | Aggregate | 0 | No web | Stalking | No | 1.5 | 7 |
| *Mimetus notius^6^* | Female | 7 | PLS | Cylindrical | 1 | No web | Stalking | No | 1.5 | 7 |
| *Neoscona theisi^5^* | NA | 2 | Cribellum | Cribellar | NA | Web | Orb web | Viscous | 3 | 7 |
| *Neoscona theisi^5^* | NA | 2 | ALS | MAP | 2 | Web | Orb web | Viscous | 3 | 7 |
| *Neoscona theisi^5^* | NA | 2 | ALS | Piriform | 7 | Web | Orb web | Viscous | 3 | 7 |
| *Neoscona theisi^5^* | NA | 2 | PMS | mAP | 2 | Web | Orb web | Viscous | 3 | 7 |
| *Neoscona theisi^5^* | NA | 2 | PMS | Aciniform | 2 | Web | Orb web | Viscous | 3 | 7 |
| *Neoscona theisi^5^* | NA | 2 | PMS | Cylindrical | 0 | Web | Orb web | Viscous | 3 | 7 |
| *Neoscona theisi^5^* | NA | 2 | PLS | Aciniform | 3 | Web | Orb web | Viscous | 3 | 7 |
| *Neoscona theisi^5^* | NA | 2 | PLS | Flagelliform | 1 | Web | Orb web | Viscous | 3 | 7 |
| *Neoscona theisi^5^* | NA | 2 | PLS | Aggregate | 2 | Web | Orb web | Viscous | 3 | 7 |
| *Neoscona theisi^5^* | NA | 2 | PLS | Cylindrical | 0 | Web | Orb web | Viscous | 3 | 7 |
| *Neoscona theisi^5^* | NA | 3 | Cribellum | Cribellar | NA | Web | Orb web | Viscous | 3 | 7 |
| *Neoscona theisi^5^* | NA | 3 | ALS | MAP | 2 | Web | Orb web | Viscous | 3 | 7 |
| *Neoscona theisi^5^* | NA | 3 | ALS | Piriform | 11 | Web | Orb web | Viscous | 3 | 7 |
| *Neoscona theisi^5^* | NA | 3 | PMS | mAP | 2 | Web | Orb web | Viscous | 3 | 7 |
| *Neoscona theisi^5^* | NA | 3 | PMS | Aciniform | 6 | Web | Orb web | Viscous | 3 | 7 |
| *Neoscona theisi^5^* | NA | 3 | PMS | Cylindrical | 0 | Web | Orb web | Viscous | 3 | 7 |
| *Neoscona theisi^5^* | NA | 3 | PLS | Aciniform | 10 | Web | Orb web | Viscous | 3 | 7 |
| *Neoscona theisi^5^* | NA | 3 | PLS | Flagelliform | 1 | Web | Orb web | Viscous | 3 | 7 |
| *Neoscona theisi^5^* | NA | 3 | PLS | Aggregate | 2 | Web | Orb web | Viscous | 3 | 7 |
| *Neoscona theisi^5^* | NA | 3 | PLS | Cylindrical | 0 | Web | Orb web | Viscous | 3 | 7 |
| *Neoscona theisi^5^* | Ante Pen Fem | 4 | Cribellum | Cribellar | NA | Web | Orb web | Viscous | 3 | 7 |
| *Neoscona theisi^5^* | Ante Pen Fem | 4 | ALS | MAP | 2 | Web | Orb web | Viscous | 3 | 7 |
| *Neoscona theisi^5^* | Ante Pen Fem | 4 | ALS | Piriform | 24 | Web | Orb web | Viscous | 3 | 7 |
| *Neoscona theisi^5^* | Ante Pen Fem | 4 | PMS | mAP | 2 | Web | Orb web | Viscous | 3 | 7 |
| *Neoscona theisi^5^* | Ante Pen Fem | 4 | PMS | Aciniform | 18 | Web | Orb web | Viscous | 3 | 7 |
| *Neoscona theisi^5^* | Ante Pen Fem | 4 | PMS | Cylindrical | 0 | Web | Orb web | Viscous | 3 | 7 |
| *Neoscona theisi^5^* | Ante Pen Fem | 4 | PLS | Aciniform | 17 | Web | Orb web | Viscous | 3 | 7 |
| *Neoscona theisi^5^* | Ante Pen Fem | 4 | PLS | Flagelliform | 1 | Web | Orb web | Viscous | 3 | 7 |
| *Neoscona theisi^5^* | Ante Pen Fem | 4 | PLS | Aggregate | 2 | Web | Orb web | Viscous | 3 | 7 |
| *Neoscona theisi^5^* | Ante Pen Fem | 4 | PLS | Cylindrical | 0 | Web | Orb web | Viscous | 3 | 7 |
| *Neoscona theisi^5^* | Male | 5 | Cribellum | Cribellar | NA | Web | Orb web | Viscous | 3 | 7 |
| *Neoscona theisi^5^* | Male | 5 | ALS | MAP | NA | Web | Orb web | Viscous | 3 | 7 |
| *Neoscona theisi^5^* | Male | 5 | ALS | Piriform | 40 | Web | Orb web | Viscous | 3 | 7 |
| *Neoscona theisi^5^* | Male | 5 | PMS | mAP | NA | Web | Orb web | Viscous | 3 | 7 |
| *Neoscona theisi^5^* | Male | 5 | PMS | Aciniform | 42 | Web | Orb web | Viscous | 3 | 7 |
| *Neoscona theisi^5^* | Male | 5 | PMS | Cylindrical | 0 | Web | Orb web | Viscous | 3 | 7 |
| *Neoscona theisi^5^* | Male | 5 | PLS | Aciniform | 29 | Web | Orb web | Viscous | 3 | 7 |
| *Neoscona theisi^5^* | Male | 5 | PLS | Flagelliform | 0 | Web | Orb web | Viscous | 3 | 7 |
| *Neoscona theisi^5^* | Male | 5 | PLS | Aggregate | 0 | Web | Orb web | Viscous | 3 | 7 |
| *Neoscona theisi^5^* | Male | 5 | PLS | Cylindrical | 0 | Web | Orb web | Viscous | 3 | 7 |
| *Neoscona theisi^5^* | Pen Fem | 5 | Cribellum | Cribellar | NA | Web | Orb web | Viscous | 3 | 7 |
| *Neoscona theisi^5^* | Pen Fem | 5 | ALS | MAP | NA | Web | Orb web | Viscous | 3 | 7 |
| *Neoscona theisi^5^* | Pen Fem | 5 | ALS | Piriform | 45 | Web | Orb web | Viscous | 3 | 7 |
| *Neoscona theisi^5^* | Pen Fem | 5 | PMS | mAP | NA | Web | Orb web | Viscous | 3 | 7 |
| *Neoscona theisi^5^* | Pen Fem | 5 | PMS | Aciniform | 42 | Web | Orb web | Viscous | 3 | 7 |
| *Neoscona theisi^5^* | Pen Fem | 5 | PMS | Cylindrical | NA | Web | Orb web | Viscous | 3 | 7 |
| *Neoscona theisi^5^* | Pen Fem | 5 | PLS | Aciniform | 30 | Web | Orb web | Viscous | 3 | 7 |
| *Neoscona theisi^5^* | Pen Fem | 5 | PLS | Flagelliform | 1 | Web | Orb web | Viscous | 3 | 7 |
| *Neoscona theisi^5^* | Pen Fem | 5 | PLS | Aggregate | 2 | Web | Orb web | Viscous | 3 | 7 |
| *Neoscona theisi^5^* | Pen Fem | 5 | PLS | Cylindrical | NA | Web | Orb web | Viscous | 3 | 7 |
| *Neoscona theisi^5^* | Female | 6 | Cribellum | Cribellar | NA | Web | Orb web | Viscous | 3 | 7 |
| *Neoscona theisi^5^* | Female | 6 | ALS | MAP | 2 | Web | Orb web | Viscous | 3 | 7 |
| *Neoscona theisi^5^* | Female | 6 | ALS | Piriform | 65 | Web | Orb web | Viscous | 3 | 7 |
| *Neoscona theisi^5^* | Female | 6 | PMS | mAP | 1 | Web | Orb web | Viscous | 3 | 7 |
| *Neoscona theisi^5^* | Female | 6 | PMS | Aciniform | 66 | Web | Orb web | Viscous | 3 | 7 |
| *Neoscona theisi^5^* | Female | 6 | PMS | Cylindrical | 1 | Web | Orb web | Viscous | 3 | 7 |
| *Neoscona theisi^5^* | Female | 6 | PLS | Aciniform | 54 | Web | Orb web | Viscous | 3 | 7 |
| *Neoscona theisi^5^* | Female | 6 | PLS | Flagelliform | 1 | Web | Orb web | Viscous | 3 | 7 |
| *Neoscona theisi^5^* | Female | 6 | PLS | Aggregate | 2 | Web | Orb web | Viscous | 3 | 7 |
| *Neoscona theisi^5^* | Female | 6 | PLS | Cylindrical | 2 | Web | Orb web | Viscous | 3 | 7 |
| *Neoscona theisi^5^* | Female | 7 | Cribellum | Cribellar | NA | Web | Orb web | Viscous | 3 | 7 |
| *Neoscona theisi^5^* | Female | 7 | ALS | MAP | NA | Web | Orb web | Viscous | 3 | 7 |
| *Neoscona theisi^5^* | Female | 7 | ALS | Piriform | 74 | Web | Orb web | Viscous | 3 | 7 |
| *Neoscona theisi^5^* | Female | 7 | PMS | mAP | NA | Web | Orb web | Viscous | 3 | 7 |
| *Neoscona theisi^5^* | Female | 7 | PMS | Aciniform | 78 | Web | Orb web | Viscous | 3 | 7 |
| *Neoscona theisi^5^* | Female | 7 | PMS | Cylindrical | NA | Web | Orb web | Viscous | 3 | 7 |
| *Neoscona theisi^5^* | Female | 7 | PLS | Aciniform | 50 | Web | Orb web | Viscous | 3 | 7 |
| *Neoscona theisi^5^* | Female | 7 | PLS | Flagelliform | NA | Web | Orb web | Viscous | 3 | 7 |
| *Neoscona theisi^5^* | Female | 7 | PLS | Aggregate | NA | Web | Orb web | Viscous | 3 | 7 |
| *Neoscona theisi^5^* | Female | 7 | PLS | Cylindrical | NA | Web | Orb web | Viscous | 3 | 7 |
| *Araneus cavaticus^6^* | NA | 2 | Cribellum | Cribellar | NA | Web | Orb web | Viscous | 3 | 12 |
| *Araneus cavaticus^6^* | NA | 2 | ALS | MAP | 2 | Web | Orb web | Viscous | 3 | 12 |
| *Araneus cavaticus^6^* | NA | 2 | ALS | Modified Pi | 0 | Web | Orb web | Viscous | 3 | 12 |
| *Araneus cavaticus^6^* | NA | 2 | ALS | Piriform | 7 | Web | Orb web | Viscous | 3 | 12 |
| *Araneus cavaticus^6^* | NA | 2 | PMS | mAP | 2 | Web | Orb web | Viscous | 3 | 12 |
| *Araneus cavaticus^6^* | NA | 2 | PMS | Aciniform | 2 | Web | Orb web | Viscous | 3 | 12 |
| *Araneus cavaticus^6^* | NA | 2 | PMS | Cylindrical | 0 | Web | Orb web | Viscous | 3 | 12 |
| *Araneus cavaticus^6^* | NA | 2 | PLS | Aciniform | 3 | Web | Orb web | Viscous | 3 | 12 |
| *Araneus cavaticus^6^* | NA | 2 | PLS | Flagelliform | 1 | Web | Orb web | Viscous | 3 | 12 |
| *Araneus cavaticus^6^* | NA | 2 | PLS | Aggregate | 2 | Web | Orb web | Viscous | 3 | 12 |
| *Araneus cavaticus^6^* | NA | 2 | PLS | Cylindrical | 0 | Web | Orb web | Viscous | 3 | 12 |
| *Araneus cavaticus^6^* | NA | 3 | Cribellum | Cribellar | NA | Web | Orb web | Viscous | 3 | 12 |
| *Araneus cavaticus^6^* | NA | 3 | ALS | MAP | 2 | Web | Orb web | Viscous | 3 | 12 |
| *Araneus cavaticus^6^* | NA | 3 | ALS | Modified Pi | 0 | Web | Orb web | Viscous | 3 | 12 |
| *Araneus cavaticus^6^* | NA | 3 | ALS | Piriform | 13 | Web | Orb web | Viscous | 3 | 12 |
| *Araneus cavaticus^6^* | NA | 3 | PMS | mAP | 2 | Web | Orb web | Viscous | 3 | 12 |
| *Araneus cavaticus^6^* | NA | 3 | PMS | Aciniform | 6 | Web | Orb web | Viscous | 3 | 12 |
| *Araneus cavaticus^6^* | NA | 3 | PMS | Cylindrical | 0 | Web | Orb web | Viscous | 3 | 12 |
| *Araneus cavaticus^6^* | NA | 3 | PLS | Aciniform | 9 | Web | Orb web | Viscous | 3 | 12 |
| *Araneus cavaticus^6^* | NA | 3 | PLS | Flagelliform | 1 | Web | Orb web | Viscous | 3 | 12 |
| *Araneus cavaticus^6^* | NA | 3 | PLS | Aggregate | 2 | Web | Orb web | Viscous | 3 | 12 |
| *Araneus cavaticus^6^* | NA | 3 | PLS | Cylindrical | 0 | Web | Orb web | Viscous | 3 | 12 |
| *Araneus cavaticus^6^* | NA | 4 | Cribellum | Cribellar | NA | Web | Orb web | Viscous | 3 | 12 |
| *Araneus cavaticus^6^* | NA | 4 | ALS | MAP | 2 | Web | Orb web | Viscous | 3 | 12 |
| *Araneus cavaticus^6^* | NA | 4 | ALS | Modified Pi | 0 | Web | Orb web | Viscous | 3 | 12 |
| *Araneus cavaticus^6^* | NA | 4 | ALS | Piriform | 20 | Web | Orb web | Viscous | 3 | 12 |
| *Araneus cavaticus^6^* | NA | 4 | PMS | mAP | 2 | Web | Orb web | Viscous | 3 | 12 |
| *Araneus cavaticus^6^* | NA | 4 | PMS | Aciniform | 15 | Web | Orb web | Viscous | 3 | 12 |
| *Araneus cavaticus^6^* | NA | 4 | PMS | Cylindrical | 0 | Web | Orb web | Viscous | 3 | 12 |
| *Araneus cavaticus^6^* | NA | 4 | PLS | Aciniform | 13 | Web | Orb web | Viscous | 3 | 12 |
| *Araneus cavaticus^6^* | NA | 4 | PLS | Flagelliform | 1 | Web | Orb web | Viscous | 3 | 12 |
| *Araneus cavaticus^6^* | NA | 4 | PLS | Aggregate | 2 | Web | Orb web | Viscous | 3 | 12 |
| *Araneus cavaticus^6^* | NA | 4 | PLS | Cylindrical | 0 | Web | Orb web | Viscous | 3 | 12 |
| *Araneus cavaticus^6^* | Juve Male | 5 | Cribellum | Cribellar | NA | Web | Orb web | Viscous | 3 | 12 |
| *Araneus cavaticus^6^* | Juve Male | 5 | ALS | MAP | 2 | Web | Orb web | Viscous | 3 | 12 |
| *Araneus cavaticus^6^* | Juve Male | 5 | ALS | Modified Pi | 0 | Web | Orb web | Viscous | 3 | 12 |
| *Araneus cavaticus^6^* | Juve Male | 5 | ALS | Piriform | 27 | Web | Orb web | Viscous | 3 | 12 |
| *Araneus cavaticus^6^* | Juve Male | 5 | PMS | mAP | 2 | Web | Orb web | Viscous | 3 | 12 |
| *Araneus cavaticus^6^* | Juve Male | 5 | PMS | Aciniform | 31 | Web | Orb web | Viscous | 3 | 12 |
| *Araneus cavaticus^6^* | Juve Male | 5 | PMS | Cylindrical | 0 | Web | Orb web | Viscous | 3 | 12 |
| *Araneus cavaticus^6^* | Juve Male | 5 | PLS | Aciniform | 25 | Web | Orb web | Viscous | 3 | 12 |
| *Araneus cavaticus^6^* | Juve Male | 5 | PLS | Flagelliform | 1 | Web | Orb web | Viscous | 3 | 12 |
| *Araneus cavaticus^6^* | Juve Male | 5 | PLS | Aggregate | 2 | Web | Orb web | Viscous | 3 | 12 |
| *Araneus cavaticus^6^* | Juve Male | 5 | PLS | Cylindrical | 0 | Web | Orb web | Viscous | 3 | 12 |
| *Araneus cavaticus^6^* | Juve Fem | 5 | Cribellum | Cribellar | NA | Web | Orb web | Viscous | 3 | 12 |
| *Araneus cavaticus^6^* | Juve Fem | 5 | ALS | MAP | 2 | Web | Orb web | Viscous | 3 | 12 |
| *Araneus cavaticus^6^* | Juve Fem | 5 | ALS | Modified Pi | 0 | Web | Orb web | Viscous | 3 | 12 |
| *Araneus cavaticus^6^* | Juve Fem | 5 | ALS | Piriform | 28 | Web | Orb web | Viscous | 3 | 12 |
| *Araneus cavaticus^6^* | Juve Fem | 5 | PMS | mAP | 2 | Web | Orb web | Viscous | 3 | 12 |
| *Araneus cavaticus^6^* | Juve Fem | 5 | PMS | Aciniform | 28 | Web | Orb web | Viscous | 3 | 12 |
| *Araneus cavaticus^6^* | Juve Fem | 5 | PMS | Cylindrical | 1 | Web | Orb web | Viscous | 3 | 12 |
| *Araneus cavaticus^6^* | Juve Fem | 5 | PLS | Aciniform | 22 | Web | Orb web | Viscous | 3 | 12 |
| *Araneus cavaticus^6^* | Juve Fem | 5 | PLS | Flagelliform | 1 | Web | Orb web | Viscous | 3 | 12 |
| *Araneus cavaticus^6^* | Juve Fem | 5 | PLS | Aggregate | 2 | Web | Orb web | Viscous | 3 | 12 |
| *Araneus cavaticus^6^* | Juve Fem | 5 | PLS | Cylindrical | 2 | Web | Orb web | Viscous | 3 | 12 |
| *Araneus cavaticus^6^* | Juve Male | 6 | Cribellum | Cribellar | NA | Web | Orb web | Viscous | 3 | 12 |
| *Araneus cavaticus^6^* | Juve Male | 6 | ALS | MAP | 2 | Web | Orb web | Viscous | 3 | 12 |
| *Araneus cavaticus^6^* | Juve Male | 6 | ALS | Modified Pi | 0 | Web | Orb web | Viscous | 3 | 12 |
| *Araneus cavaticus^6^* | Juve Male | 6 | ALS | Piriform | 41 | Web | Orb web | Viscous | 3 | 12 |
| *Araneus cavaticus^6^* | Juve Male | 6 | PMS | mAP | 2 | Web | Orb web | Viscous | 3 | 12 |
| *Araneus cavaticus^6^* | Juve Male | 6 | PMS | Aciniform | 54 | Web | Orb web | Viscous | 3 | 12 |
| *Araneus cavaticus^6^* | Juve Male | 6 | PMS | Cylindrical | 0 | Web | Orb web | Viscous | 3 | 12 |
| *Araneus cavaticus^6^* | Juve Male | 6 | PLS | Aciniform | 42 | Web | Orb web | Viscous | 3 | 12 |
| *Araneus cavaticus^6^* | Juve Male | 6 | PLS | Flagelliform | 1 | Web | Orb web | Viscous | 3 | 12 |
| *Araneus cavaticus^6^* | Juve Male | 6 | PLS | Aggregate | 2 | Web | Orb web | Viscous | 3 | 12 |
| *Araneus cavaticus^6^* | Juve Male | 6 | PLS | Cylindrical | 0 | Web | Orb web | Viscous | 3 | 12 |
| *Araneus cavaticus^6^* | Juve Fem | 6 | Cribellum | Cribellar | NA | Web | Orb web | Viscous | 3 | 12 |
| *Araneus cavaticus^6^* | Juve Fem | 6 | ALS | MAP | 2 | Web | Orb web | Viscous | 3 | 12 |
| *Araneus cavaticus^6^* | Juve Fem | 6 | ALS | Modified Pi | 0 | Web | Orb web | Viscous | 3 | 12 |
| *Araneus cavaticus^6^* | Juve Fem | 6 | ALS | Piriform | 43 | Web | Orb web | Viscous | 3 | 12 |
| *Araneus cavaticus^6^* | Juve Fem | 6 | PMS | mAP | 2 | Web | Orb web | Viscous | 3 | 12 |
| *Araneus cavaticus^6^* | Juve Fem | 6 | PMS | Aciniform | 47 | Web | Orb web | Viscous | 3 | 12 |
| *Araneus cavaticus^6^* | Juve Fem | 6 | PMS | Cylindrical | 1 | Web | Orb web | Viscous | 3 | 12 |
| *Araneus cavaticus^6^* | Juve Fem | 6 | PLS | Aciniform | 39 | Web | Orb web | Viscous | 3 | 12 |
| *Araneus cavaticus^6^* | Juve Fem | 6 | PLS | Flagelliform | 1 | Web | Orb web | Viscous | 3 | 12 |
| *Araneus cavaticus^6^* | Juve Fem | 6 | PLS | Aggregate | 2 | Web | Orb web | Viscous | 3 | 12 |
| *Araneus cavaticus^6^* | Juve Fem | 6 | PLS | Cylindrical | 2 | Web | Orb web | Viscous | 3 | 12 |
| *Araneus cavaticus^6^* | Juve Male | 7 | Cribellum | Cribellar | NA | Web | Orb web | Viscous | 3 | 12 |
| *Araneus cavaticus^6^* | Juve Male | 7 | ALS | MAP | 2 | Web | Orb web | Viscous | 3 | 12 |
| *Araneus cavaticus^6^* | Juve Male | 7 | ALS | Modified Pi | 0 | Web | Orb web | Viscous | 3 | 12 |
| *Araneus cavaticus^6^* | Juve Male | 7 | ALS | Piriform | 57 | Web | Orb web | Viscous | 3 | 12 |
| *Araneus cavaticus^6^* | Juve Male | 7 | PMS | mAP | 2 | Web | Orb web | Viscous | 3 | 12 |
| *Araneus cavaticus^6^* | Juve Male | 7 | PMS | Aciniform | 91 | Web | Orb web | Viscous | 3 | 12 |
| *Araneus cavaticus^6^* | Juve Male | 7 | PMS | Cylindrical | 0 | Web | Orb web | Viscous | 3 | 12 |
| *Araneus cavaticus^6^* | Juve Male | 7 | PLS | Aciniform | 68 | Web | Orb web | Viscous | 3 | 12 |
| *Araneus cavaticus^6^* | Juve Male | 7 | PLS | Flagelliform | 1 | Web | Orb web | Viscous | 3 | 12 |
| *Araneus cavaticus^6^* | Juve Male | 7 | PLS | Aggregate | 2 | Web | Orb web | Viscous | 3 | 12 |
| *Araneus cavaticus^6^* | Juve Male | 7 | PLS | Cylindrical | 0 | Web | Orb web | Viscous | 3 | 12 |
| *Araneus cavaticus^6^* | Juve Fem | 7 | Cribellum | Cribellar | NA | Web | Orb web | Viscous | 3 | 12 |
| *Araneus cavaticus^6^* | Juve Fem | 7 | ALS | MAP | 2 | Web | Orb web | Viscous | 3 | 12 |
| *Araneus cavaticus^6^* | Juve Fem | 7 | ALS | Modified Pi | 0 | Web | Orb web | Viscous | 3 | 12 |
| *Araneus cavaticus^6^* | Juve Fem | 7 | ALS | Piriform | 65 | Web | Orb web | Viscous | 3 | 12 |
| *Araneus cavaticus^6^* | Juve Fem | 7 | PMS | mAP | 2 | Web | Orb web | Viscous | 3 | 12 |
| *Araneus cavaticus^6^* | Juve Fem | 7 | PMS | Aciniform | 96 | Web | Orb web | Viscous | 3 | 12 |
| *Araneus cavaticus^6^* | Juve Fem | 7 | PMS | Cylindrical | 1 | Web | Orb web | Viscous | 3 | 12 |
| *Araneus cavaticus^6^* | Juve Fem | 7 | PLS | Aciniform | 69 | Web | Orb web | Viscous | 3 | 12 |
| *Araneus cavaticus^6^* | Juve Fem | 7 | PLS | Flagelliform | 1 | Web | Orb web | Viscous | 3 | 12 |
| *Araneus cavaticus^6^* | Juve Fem | 7 | PLS | Aggregate | 2 | Web | Orb web | Viscous | 3 | 12 |
| *Araneus cavaticus^6^* | Juve Fem | 7 | PLS | Cylindrical | 2 | Web | Orb web | Viscous | 3 | 12 |
| *Araneus cavaticus^6^* | Pen Male | 8 | Cribellum | Cribellar | NA | Web | Orb web | Viscous | 3 | 12 |
| *Araneus cavaticus^6^* | Pen Male | 8 | ALS | MAP | 2 | Web | Orb web | Viscous | 3 | 12 |
| *Araneus cavaticus^6^* | Pen Male | 8 | ALS | Modified Pi | 0 | Web | Orb web | Viscous | 3 | 12 |
| *Araneus cavaticus^6^* | Pen Male | 8 | ALS | Piriform | 90 | Web | Orb web | Viscous | 3 | 12 |
| *Araneus cavaticus^6^* | Pen Male | 8 | PMS | mAP | 2 | Web | Orb web | Viscous | 3 | 12 |
| *Araneus cavaticus^6^* | Pen Male | 8 | PMS | Aciniform | 153 | Web | Orb web | Viscous | 3 | 12 |
| *Araneus cavaticus^6^* | Pen Male | 8 | PMS | Cylindrical | 0 | Web | Orb web | Viscous | 3 | 12 |
| *Araneus cavaticus^6^* | Pen Male | 8 | PLS | Aciniform | 119 | Web | Orb web | Viscous | 3 | 12 |
| *Araneus cavaticus^6^* | Pen Male | 8 | PLS | Flagelliform | 1 | Web | Orb web | Viscous | 3 | 12 |
| *Araneus cavaticus^6^* | Pen Male | 8 | PLS | Aggregate | 2 | Web | Orb web | Viscous | 3 | 12 |
| *Araneus cavaticus^6^* | Pen Male | 8 | PLS | Cylindrical | 0 | Web | Orb web | Viscous | 3 | 12 |
| *Araneus cavaticus^6^* | Pen Fem | 8 | Cribellum | Cribellar | NA | Web | Orb web | Viscous | 3 | 12 |
| *Araneus cavaticus^6^* | Pen Fem | 8 | ALS | MAP | 2 | Web | Orb web | Viscous | 3 | 12 |
| *Araneus cavaticus^6^* | Pen Fem | 8 | ALS | Modified Pi | 0 | Web | Orb web | Viscous | 3 | 12 |
| *Araneus cavaticus^6^* | Pen Fem | 8 | ALS | Piriform | 94 | Web | Orb web | Viscous | 3 | 12 |
| *Araneus cavaticus^6^* | Pen Fem | 8 | PMS | mAP | 2 | Web | Orb web | Viscous | 3 | 12 |
| *Araneus cavaticus^6^* | Pen Fem | 8 | PMS | Aciniform | 155 | Web | Orb web | Viscous | 3 | 12 |
| *Araneus cavaticus^6^* | Pen Fem | 8 | PMS | Cylindrical | 1 | Web | Orb web | Viscous | 3 | 12 |
| *Araneus cavaticus^6^* | Pen Fem | 8 | PLS | Aciniform | 114 | Web | Orb web | Viscous | 3 | 12 |
| *Araneus cavaticus^6^* | Pen Fem | 8 | PLS | Flagelliform | 1 | Web | Orb web | Viscous | 3 | 12 |
| *Araneus cavaticus^6^* | Pen Fem | 8 | PLS | Aggregate | 2 | Web | Orb web | Viscous | 3 | 12 |
| *Araneus cavaticus^6^* | Pen Fem | 8 | PLS | Cylindrical | 2 | Web | Orb web | Viscous | 3 | 12 |
| *Araneus cavaticus^6^* | Pen Male | 9 | Cribellum | Cribellar | NA | Web | Orb web | Viscous | 3 | 12 |
| *Araneus cavaticus^6^* | Pen Male | 9 | ALS | MAP | 2 | Web | Orb web | Viscous | 3 | 12 |
| *Araneus cavaticus^6^* | Pen Male | 9 | ALS | Modified Pi | 0 | Web | Orb web | Viscous | 3 | 12 |
| *Araneus cavaticus^6^* | Pen Male | 9 | ALS | Piriform | 122 | Web | Orb web | Viscous | 3 | 12 |
| *Araneus cavaticus^6^* | Pen Male | 9 | PMS | mAP | 2 | Web | Orb web | Viscous | 3 | 12 |
| *Araneus cavaticus^6^* | Pen Male | 9 | PMS | Aciniform | 207 | Web | Orb web | Viscous | 3 | 12 |
| *Araneus cavaticus^6^* | Pen Male | 9 | PMS | Cylindrical | 0 | Web | Orb web | Viscous | 3 | 12 |
| *Araneus cavaticus^6^* | Pen Male | 9 | PLS | Aciniform | 181 | Web | Orb web | Viscous | 3 | 12 |
| *Araneus cavaticus^6^* | Pen Male | 9 | PLS | Flagelliform | 1 | Web | Orb web | Viscous | 3 | 12 |
| *Araneus cavaticus^6^* | Pen Male | 9 | PLS | Aggregate | 2 | Web | Orb web | Viscous | 3 | 12 |
| *Araneus cavaticus^6^* | Pen Male | 9 | PLS | Cylindrical | 0 | Web | Orb web | Viscous | 3 | 12 |
| *Araneus cavaticus^6^* | Pen Fem | 9 | Cribellum | Cribellar | NA | Web | Orb web | Viscous | 3 | 12 |
| *Araneus cavaticus^6^* | Pen Fem | 9 | ALS | MAP | 2 | Web | Orb web | Viscous | 3 | 12 |
| *Araneus cavaticus^6^* | Pen Fem | 9 | ALS | Modified Pi | 0 | Web | Orb web | Viscous | 3 | 12 |
| *Araneus cavaticus^6^* | Pen Fem | 9 | ALS | Piriform | 134 | Web | Orb web | Viscous | 3 | 12 |
| *Araneus cavaticus^6^* | Pen Fem | 9 | PMS | mAP | 2 | Web | Orb web | Viscous | 3 | 12 |
| *Araneus cavaticus^6^* | Pen Fem | 9 | PMS | Aciniform | 215 | Web | Orb web | Viscous | 3 | 12 |
| *Araneus cavaticus^6^* | Pen Fem | 9 | PMS | Cylindrical | 1 | Web | Orb web | Viscous | 3 | 12 |
| *Araneus cavaticus^6^* | Pen Fem | 9 | PLS | Aciniform | 187 | Web | Orb web | Viscous | 3 | 12 |
| *Araneus cavaticus^6^* | Pen Fem | 9 | PLS | Flagelliform | 1 | Web | Orb web | Viscous | 3 | 12 |
| *Araneus cavaticus^6^* | Pen Fem | 9 | PLS | Aggregate | 2 | Web | Orb web | Viscous | 3 | 12 |
| *Araneus cavaticus^6^* | Pen Fem | 9 | PLS | Cylindrical | 2 | Web | Orb web | Viscous | 3 | 12 |
| *Araneus cavaticus^6^* | Male | 9 | Cribellum | Cribellar | NA | Web | Orb web | Viscous | 3 | 12 |
| *Araneus cavaticus^6^* | Male | 9 | ALS | MAP | 1 | Web | Orb web | Viscous | 3 | 12 |
| *Araneus cavaticus^6^* | Male | 9 | ALS | Modified Pi | 0 | Web | Orb web | Viscous | 3 | 12 |
| *Araneus cavaticus^6^* | Male | 9 | ALS | Piriform | 146 | Web | Orb web | Viscous | 3 | 12 |
| *Araneus cavaticus^6^* | Male | 9 | PMS | mAP | 1 | Web | Orb web | Viscous | 3 | 12 |
| *Araneus cavaticus^6^* | Male | 9 | PMS | Aciniform | NA | Web | Orb web | Viscous | 3 | 12 |
| *Araneus cavaticus^6^* | Male | 9 | PMS | Cylindrical | 0 | Web | Orb web | Viscous | 3 | 12 |
| *Araneus cavaticus^6^* | Male | 9 | PLS | Aciniform | NA | Web | Orb web | Viscous | 3 | 12 |
| *Araneus cavaticus^6^* | Male | 9 | PLS | Flagelliform | 0 | Web | Orb web | Viscous | 3 | 12 |
| *Araneus cavaticus^6^* | Male | 9 | PLS | Aggregate | 0 | Web | Orb web | Viscous | 3 | 12 |
| *Araneus cavaticus^6^* | Male | 9 | PLS | Cylindrical | 0 | Web | Orb web | Viscous | 3 | 12 |
| *Araneus cavaticus^6^* | Pen Male | 10 | Cribellum | Cribellar | NA | Web | Orb web | Viscous | 3 | 12 |
| *Araneus cavaticus^6^* | Pen Male | 10 | ALS | MAP | 2 | Web | Orb web | Viscous | 3 | 12 |
| *Araneus cavaticus^6^* | Pen Male | 10 | ALS | Modified Pi | 0 | Web | Orb web | Viscous | 3 | 12 |
| *Araneus cavaticus^6^* | Pen Male | 10 | ALS | Piriform | 153 | Web | Orb web | Viscous | 3 | 12 |
| *Araneus cavaticus^6^* | Pen Male | 10 | PMS | mAP | 2 | Web | Orb web | Viscous | 3 | 12 |
| *Araneus cavaticus^6^* | Pen Male | 10 | PMS | Aciniform | 239 | Web | Orb web | Viscous | 3 | 12 |
| *Araneus cavaticus^6^* | Pen Male | 10 | PMS | Cylindrical | 0 | Web | Orb web | Viscous | 3 | 12 |
| *Araneus cavaticus^6^* | Pen Male | 10 | PLS | Aciniform | 228 | Web | Orb web | Viscous | 3 | 12 |
| *Araneus cavaticus^6^* | Pen Male | 10 | PLS | Flagelliform | 1 | Web | Orb web | Viscous | 3 | 12 |
| *Araneus cavaticus^6^* | Pen Male | 10 | PLS | Aggregate | 2 | Web | Orb web | Viscous | 3 | 12 |
| *Araneus cavaticus^6^* | Pen Male | 10 | PLS | Cylindrical | 0 | Web | Orb web | Viscous | 3 | 12 |
| *Araneus cavaticus^6^* | Pen Fem | 10 | Cribellum | Cribellar | NA | Web | Orb web | Viscous | 3 | 12 |
| *Araneus cavaticus^6^* | Pen Fem | 10 | ALS | MAP | 2 | Web | Orb web | Viscous | 3 | 12 |
| *Araneus cavaticus^6^* | Pen Fem | 10 | ALS | Modified Pi | 0 | Web | Orb web | Viscous | 3 | 12 |
| *Araneus cavaticus^6^* | Pen Fem | 10 | ALS | Piriform | 162 | Web | Orb web | Viscous | 3 | 12 |
| *Araneus cavaticus^6^* | Pen Fem | 10 | PMS | mAP | 2 | Web | Orb web | Viscous | 3 | 12 |
| *Araneus cavaticus^6^* | Pen Fem | 10 | PMS | Aciniform | 266 | Web | Orb web | Viscous | 3 | 12 |
| *Araneus cavaticus^6^* | Pen Fem | 10 | PMS | Cylindrical | 1 | Web | Orb web | Viscous | 3 | 12 |
| *Araneus cavaticus^6^* | Pen Fem | 10 | PLS | Aciniform | 223 | Web | Orb web | Viscous | 3 | 12 |
| *Araneus cavaticus^6^* | Pen Fem | 10 | PLS | Flagelliform | 1 | Web | Orb web | Viscous | 3 | 12 |
| *Araneus cavaticus^6^* | Pen Fem | 10 | PLS | Aggregate | 2 | Web | Orb web | Viscous | 3 | 12 |
| *Araneus cavaticus^6^* | Pen Fem | 10 | PLS | Cylindrical | 2 | Web | Orb web | Viscous | 3 | 12 |
| *Araneus cavaticus^6^* | Male | 10 | Cribellum | Cribellar | NA | Web | Orb web | Viscous | 3 | 12 |
| *Araneus cavaticus^6^* | Male | 10 | ALS | MAP | 1 | Web | Orb web | Viscous | 3 | 12 |
| *Araneus cavaticus^6^* | Male | 10 | ALS | Modified Pi | 0 | Web | Orb web | Viscous | 3 | 12 |
| *Araneus cavaticus^6^* | Male | 10 | ALS | Piriform | 161 | Web | Orb web | Viscous | 3 | 12 |
| *Araneus cavaticus^6^* | Male | 10 | PMS | mAP | 1 | Web | Orb web | Viscous | 3 | 12 |
| *Araneus cavaticus^6^* | Male | 10 | PMS | Aciniform | 185 | Web | Orb web | Viscous | 3 | 12 |
| *Araneus cavaticus^6^* | Male | 10 | PMS | Cylindrical | 0 | Web | Orb web | Viscous | 3 | 12 |
| *Araneus cavaticus^6^* | Male | 10 | PLS | Aciniform | 150 | Web | Orb web | Viscous | 3 | 12 |
| *Araneus cavaticus^6^* | Male | 10 | PLS | Flagelliform | 0 | Web | Orb web | Viscous | 3 | 12 |
| *Araneus cavaticus^6^* | Male | 10 | PLS | Aggregate | 0 | Web | Orb web | Viscous | 3 | 12 |
| *Araneus cavaticus^6^* | Male | 10 | PLS | Cylindrical | 0 | Web | Orb web | Viscous | 3 | 12 |
| *Araneus cavaticus^6^* | Female | 10 | Cribellum | Cribellar | NA | Web | Orb web | Viscous | 3 | 12 |
| *Araneus cavaticus^6^* | Female | 10 | ALS | MAP | 1 | Web | Orb web | Viscous | 3 | 12 |
| *Araneus cavaticus^6^* | Female | 10 | ALS | Modified Pi | 0 | Web | Orb web | Viscous | 3 | 12 |
| *Araneus cavaticus^6^* | Female | 10 | ALS | Piriform | NA | Web | Orb web | Viscous | 3 | 12 |
| *Araneus cavaticus^6^* | Female | 10 | PMS | mAP | 1 | Web | Orb web | Viscous | 3 | 12 |
| *Araneus cavaticus^6^* | Female | 10 | PMS | Aciniform | 282 | Web | Orb web | Viscous | 3 | 12 |
| *Araneus cavaticus^6^* | Female | 10 | PMS | Cylindrical | 1 | Web | Orb web | Viscous | 3 | 12 |
| *Araneus cavaticus^6^* | Female | 10 | PLS | Aciniform | NA | Web | Orb web | Viscous | 3 | 12 |
| *Araneus cavaticus^6^* | Female | 10 | PLS | Flagelliform | 1 | Web | Orb web | Viscous | 3 | 12 |
| *Araneus cavaticus^6^* | Female | 10 | PLS | Aggregate | 2 | Web | Orb web | Viscous | 3 | 12 |
| *Araneus cavaticus^6^* | Female | 10 | PLS | Cylindrical | 2 | Web | Orb web | Viscous | 3 | 12 |
| *Araneus cavaticus^6^* | Pen Male | 11 | Cribellum | Cribellar | NA | Web | Orb web | Viscous | 3 | 12 |
| *Araneus cavaticus^6^* | Pen Male | 11 | ALS | MAP | 2 | Web | Orb web | Viscous | 3 | 12 |
| *Araneus cavaticus^6^* | Pen Male | 11 | ALS | Modified Pi | 0 | Web | Orb web | Viscous | 3 | 12 |
| *Araneus cavaticus^6^* | Pen Male | 11 | ALS | Piriform | NA | Web | Orb web | Viscous | 3 | 12 |
| *Araneus cavaticus^6^* | Pen Male | 11 | PMS | mAP | 2 | Web | Orb web | Viscous | 3 | 12 |
| *Araneus cavaticus^6^* | Pen Male | 11 | PMS | Aciniform | 274 | Web | Orb web | Viscous | 3 | 12 |
| *Araneus cavaticus^6^* | Pen Male | 11 | PMS | Cylindrical | 0 | Web | Orb web | Viscous | 3 | 12 |
| *Araneus cavaticus^6^* | Pen Male | 11 | PLS | Aciniform | NA | Web | Orb web | Viscous | 3 | 12 |
| *Araneus cavaticus^6^* | Pen Male | 11 | PLS | Flagelliform | 1 | Web | Orb web | Viscous | 3 | 12 |
| *Araneus cavaticus^6^* | Pen Male | 11 | PLS | Aggregate | 2 | Web | Orb web | Viscous | 3 | 12 |
| *Araneus cavaticus^6^* | Pen Male | 11 | PLS | Cylindrical | 0 | Web | Orb web | Viscous | 3 | 12 |
| *Araneus cavaticus^6^* | Pen Fem | 11 | Cribellum | Cribellar | NA | Web | Orb web | Viscous | 3 | 12 |
| *Araneus cavaticus^6^* | Pen Fem | 11 | ALS | MAP | 2 | Web | Orb web | Viscous | 3 | 12 |
| *Araneus cavaticus^6^* | Pen Fem | 11 | ALS | Modified Pi | 0 | Web | Orb web | Viscous | 3 | 12 |
| *Araneus cavaticus^6^* | Pen Fem | 11 | ALS | Piriform | 199 | Web | Orb web | Viscous | 3 | 12 |
| *Araneus cavaticus^6^* | Pen Fem | 11 | PMS | mAP | 2 | Web | Orb web | Viscous | 3 | 12 |
| *Araneus cavaticus^6^* | Pen Fem | 11 | PMS | Aciniform | 293 | Web | Orb web | Viscous | 3 | 12 |
| *Araneus cavaticus^6^* | Pen Fem | 11 | PMS | Cylindrical | 1 | Web | Orb web | Viscous | 3 | 12 |
| *Araneus cavaticus^6^* | Pen Fem | 11 | PLS | Aciniform | 262 | Web | Orb web | Viscous | 3 | 12 |
| *Araneus cavaticus^6^* | Pen Fem | 11 | PLS | Flagelliform | 1 | Web | Orb web | Viscous | 3 | 12 |
| *Araneus cavaticus^6^* | Pen Fem | 11 | PLS | Aggregate | 2 | Web | Orb web | Viscous | 3 | 12 |
| *Araneus cavaticus^6^* | Pen Fem | 11 | PLS | Cylindrical | 2 | Web | Orb web | Viscous | 3 | 12 |
| *Araneus cavaticus^6^* | Male | 11 | Cribellum | Cribellar | NA | Web | Orb web | Viscous | 3 | 12 |
| *Araneus cavaticus^6^* | Male | 11 | ALS | MAP | 1 | Web | Orb web | Viscous | 3 | 12 |
| *Araneus cavaticus^6^* | Male | 11 | ALS | Modified Pi | 0 | Web | Orb web | Viscous | 3 | 12 |
| *Araneus cavaticus^6^* | Male | 11 | ALS | Piriform | 200 | Web | Orb web | Viscous | 3 | 12 |
| *Araneus cavaticus^6^* | Male | 11 | PMS | mAP | 1 | Web | Orb web | Viscous | 3 | 12 |
| *Araneus cavaticus^6^* | Male | 11 | PMS | Aciniform | 241 | Web | Orb web | Viscous | 3 | 12 |
| *Araneus cavaticus^6^* | Male | 11 | PMS | Cylindrical | 0 | Web | Orb web | Viscous | 3 | 12 |
| *Araneus cavaticus^6^* | Male | 11 | PLS | Aciniform | 190 | Web | Orb web | Viscous | 3 | 12 |
| *Araneus cavaticus^6^* | Male | 11 | PLS | Flagelliform | 0 | Web | Orb web | Viscous | 3 | 12 |
| *Araneus cavaticus^6^* | Male | 11 | PLS | Aggregate | 0 | Web | Orb web | Viscous | 3 | 12 |
| *Araneus cavaticus^6^* | Male | 11 | PLS | Cylindrical | 0 | Web | Orb web | Viscous | 3 | 12 |
| *Araneus cavaticus^6^* | Female | 11 | Cribellum | Cribellar | NA | Web | Orb web | Viscous | 3 | 12 |
| *Araneus cavaticus^6^* | Female | 11 | ALS | MAP | 1 | Web | Orb web | Viscous | 3 | 12 |
| *Araneus cavaticus^6^* | Female | 11 | ALS | Modified Pi | 0 | Web | Orb web | Viscous | 3 | 12 |
| *Araneus cavaticus^6^* | Female | 11 | ALS | Piriform | 270 | Web | Orb web | Viscous | 3 | 12 |
| *Araneus cavaticus^6^* | Female | 11 | PMS | mAP | 1 | Web | Orb web | Viscous | 3 | 12 |
| *Araneus cavaticus^6^* | Female | 11 | PMS | Aciniform | 319 | Web | Orb web | Viscous | 3 | 12 |
| *Araneus cavaticus^6^* | Female | 11 | PMS | Cylindrical | 1 | Web | Orb web | Viscous | 3 | 12 |
| *Araneus cavaticus^6^* | Female | 11 | PLS | Aciniform | NA | Web | Orb web | Viscous | 3 | 12 |
| *Araneus cavaticus^6^* | Female | 11 | PLS | Flagelliform | 1 | Web | Orb web | Viscous | 3 | 12 |
| *Araneus cavaticus^6^* | Female | 11 | PLS | Aggregate | 2 | Web | Orb web | Viscous | 3 | 12 |
| *Araneus cavaticus^6^* | Female | 11 | PLS | Cylindrical | 2 | Web | Orb web | Viscous | 3 | 12 |
| *Araneus cavaticus^6^* | Female | 12 | Cribellum | Cribellar | NA | Web | Orb web | Viscous | 3 | 12 |
| *Araneus cavaticus^6^* | Female | 12 | ALS | MAP | 1 | Web | Orb web | Viscous | 3 | 12 |
| *Araneus cavaticus^6^* | Female | 12 | ALS | Modified Pi | 0 | Web | Orb web | Viscous | 3 | 12 |
| *Araneus cavaticus^6^* | Female | 12 | ALS | Piriform | 235 | Web | Orb web | Viscous | 3 | 12 |
| *Araneus cavaticus^6^* | Female | 12 | PMS | mAP | 1 | Web | Orb web | Viscous | 3 | 12 |
| *Araneus cavaticus^6^* | Female | 12 | PMS | Aciniform | 351 | Web | Orb web | Viscous | 3 | 12 |
| *Araneus cavaticus^6^* | Female | 12 | PMS | Cylindrical | 1 | Web | Orb web | Viscous | 3 | 12 |
| *Araneus cavaticus^6^* | Female | 12 | PLS | Aciniform | 281 | Web | Orb web | Viscous | 3 | 12 |
| *Araneus cavaticus^6^* | Female | 12 | PLS | Flagelliform | 1 | Web | Orb web | Viscous | 3 | 12 |
| *Araneus cavaticus^6^* | Female | 12 | PLS | Aggregate | 2 | Web | Orb web | Viscous | 3 | 12 |
| *Araneus cavaticus^6^* | Female | 12 | PLS | Cylindrical | 2 | Web | Orb web | Viscous | 3 | 12 |
| *Araneus diadematus^1^* | NA | 2 | Cribellum | Cribellar | NA | Web | Orb web | Viscous | 3 | 10 |
| *Araneus diadematus^1^* | NA | 2 | ALS | MAP | 2 | Web | Orb web | Viscous | 3 | 10 |
| *Araneus diadematus^1^* | NA | 2 | ALS | Piriform | 62 | Web | Orb web | Viscous | 3 | 10 |
| *Araneus diadematus^1^* | NA | 2 | PMS | mAP | 2 | Web | Orb web | Viscous | 3 | 10 |
| *Araneus diadematus^1^* | NA | 2 | PMS | Aciniform | 38 | Web | Orb web | Viscous | 3 | 10 |
| *Araneus diadematus^1^* | NA | 2 | PMS | Cylindrical | 0 | Web | Orb web | Viscous | 3 | 10 |
| *Araneus diadematus^1^* | NA | 2 | PLS | Aciniform | 52 | Web | Orb web | Viscous | 3 | 10 |
| *Araneus diadematus^1^* | NA | 2 | PLS | Aggregate | 2 | Web | Orb web | Viscous | 3 | 10 |
| *Araneus diadematus^1^* | NA | 2 | PLS | Flagelliform | 1 | Web | Orb web | Viscous | 3 | 10 |
| *Araneus diadematus^1^* | NA | 2 | PLS | Cylindrical | 0 | Web | Orb web | Viscous | 3 | 10 |
| *Araneus diadematus^1^* | NA | 3 | Cribellum | Cribellar | NA | Web | Orb web | Viscous | 3 | 10 |
| *Araneus diadematus^1^* | NA | 3 | ALS | MAP | 2 | Web | Orb web | Viscous | 3 | 10 |
| *Araneus diadematus^1^* | NA | 3 | ALS | Piriform | 65 | Web | Orb web | Viscous | 3 | 10 |
| *Araneus diadematus^1^* | NA | 3 | PMS | mAP | 2 | Web | Orb web | Viscous | 3 | 10 |
| *Araneus diadematus^1^* | NA | 3 | PMS | Aciniform | 45 | Web | Orb web | Viscous | 3 | 10 |
| *Araneus diadematus^1^* | NA | 3 | PMS | Cylindrical | 0 | Web | Orb web | Viscous | 3 | 10 |
| *Araneus diadematus^1^* | NA | 3 | PLS | Aciniform | 56 | Web | Orb web | Viscous | 3 | 10 |
| *Araneus diadematus^1^* | NA | 3 | PLS | Aggregate | 2 | Web | Orb web | Viscous | 3 | 10 |
| *Araneus diadematus^1^* | NA | 3 | PLS | Flagelliform | 1 | Web | Orb web | Viscous | 3 | 10 |
| *Araneus diadematus^1^* | NA | 3 | PLS | Cylindrical | 0 | Web | Orb web | Viscous | 3 | 10 |
| *Araneus diadematus^1^* | Juve Fem | 4 | Cribellum | Cribellar | NA | Web | Orb web | Viscous | 3 | 10 |
| *Araneus diadematus^1^* | Juve Fem | 4 | ALS | MAP | 2 | Web | Orb web | Viscous | 3 | 10 |
| *Araneus diadematus^1^* | Juve Fem | 4 | ALS | Piriform | 74 | Web | Orb web | Viscous | 3 | 10 |
| *Araneus diadematus^1^* | Juve Fem | 4 | PMS | mAP | 2 | Web | Orb web | Viscous | 3 | 10 |
| *Araneus diadematus^1^* | Juve Fem | 4 | PMS | Aciniform | 67 | Web | Orb web | Viscous | 3 | 10 |
| *Araneus diadematus^1^* | Juve Fem | 4 | PMS | Cylindrical | 0 | Web | Orb web | Viscous | 3 | 10 |
| *Araneus diadematus^1^* | Juve Fem | 4 | PLS | Aciniform | 80 | Web | Orb web | Viscous | 3 | 10 |
| *Araneus diadematus^1^* | Juve Fem | 4 | PLS | Aggregate | 2 | Web | Orb web | Viscous | 3 | 10 |
| *Araneus diadematus^1^* | Juve Fem | 4 | PLS | Flagelliform | 1 | Web | Orb web | Viscous | 3 | 10 |
| *Araneus diadematus^1^* | Juve Fem | 4 | PLS | Cylindrical | 0 | Web | Orb web | Viscous | 3 | 10 |
| *Araneus diadematus^1^* | Juve Male | 4 | Cribellum | Cribellar | NA | Web | Orb web | Viscous | 3 | 10 |
| *Araneus diadematus^1^* | Juve Male | 4 | ALS | MAP | 2 | Web | Orb web | Viscous | 3 | 10 |
| *Araneus diadematus^1^* | Juve Male | 4 | ALS | Piriform | 73 | Web | Orb web | Viscous | 3 | 10 |
| *Araneus diadematus^1^* | Juve Male | 4 | PMS | mAP | 2 | Web | Orb web | Viscous | 3 | 10 |
| *Araneus diadematus^1^* | Juve Male | 4 | PMS | Aciniform | 51 | Web | Orb web | Viscous | 3 | 10 |
| *Araneus diadematus^1^* | Juve Male | 4 | PMS | Cylindrical | 0 | Web | Orb web | Viscous | 3 | 10 |
| *Araneus diadematus^1^* | Juve Male | 4 | PLS | Aciniform | 63 | Web | Orb web | Viscous | 3 | 10 |
| *Araneus diadematus^1^* | Juve Male | 4 | PLS | Aggregate | 2 | Web | Orb web | Viscous | 3 | 10 |
| *Araneus diadematus^1^* | Juve Male | 4 | PLS | Flagelliform | 1 | Web | Orb web | Viscous | 3 | 10 |
| *Araneus diadematus^1^* | Juve Male | 4 | PLS | Cylindrical | 0 | Web | Orb web | Viscous | 3 | 10 |
| *Araneus diadematus^1^* | Juve Fem | 5 | Cribellum | Cribellar | NA | Web | Orb web | Viscous | 3 | 10 |
| *Araneus diadematus^1^* | Juve Fem | 5 | ALS | MAP | 2 | Web | Orb web | Viscous | 3 | 10 |
| *Araneus diadematus^1^* | Juve Fem | 5 | ALS | Piriform | 81 | Web | Orb web | Viscous | 3 | 10 |
| *Araneus diadematus^1^* | Juve Fem | 5 | PMS | mAP | 2 | Web | Orb web | Viscous | 3 | 10 |
| *Araneus diadematus^1^* | Juve Fem | 5 | PMS | Aciniform | 84 | Web | Orb web | Viscous | 3 | 10 |
| *Araneus diadematus^1^* | Juve Fem | 5 | PMS | Cylindrical | 0 | Web | Orb web | Viscous | 3 | 10 |
| *Araneus diadematus^1^* | Juve Fem | 5 | PLS | Aciniform | 91 | Web | Orb web | Viscous | 3 | 10 |
| *Araneus diadematus^1^* | Juve Fem | 5 | PLS | Aggregate | 2 | Web | Orb web | Viscous | 3 | 10 |
| *Araneus diadematus^1^* | Juve Fem | 5 | PLS | Flagelliform | 1 | Web | Orb web | Viscous | 3 | 10 |
| *Araneus diadematus^1^* | Juve Fem | 5 | PLS | Cylindrical | 0 | Web | Orb web | Viscous | 3 | 10 |
| *Araneus diadematus^1^* | Juve Male | 5 | Cribellum | Cribellar | NA | Web | Orb web | Viscous | 3 | 10 |
| *Araneus diadematus^1^* | Juve Male | 5 | ALS | MAP | 2 | Web | Orb web | Viscous | 3 | 10 |
| *Araneus diadematus^1^* | Juve Male | 5 | ALS | Piriform | 80 | Web | Orb web | Viscous | 3 | 10 |
| *Araneus diadematus^1^* | Juve Male | 5 | PMS | mAP | 2 | Web | Orb web | Viscous | 3 | 10 |
| *Araneus diadematus^1^* | Juve Male | 5 | PMS | Aciniform | 61 | Web | Orb web | Viscous | 3 | 10 |
| *Araneus diadematus^1^* | Juve Male | 5 | PMS | Cylindrical | 0 | Web | Orb web | Viscous | 3 | 10 |
| *Araneus diadematus^1^* | Juve Male | 5 | PLS | Aciniform | 79 | Web | Orb web | Viscous | 3 | 10 |
| *Araneus diadematus^1^* | Juve Male | 5 | PLS | Aggregate | 2 | Web | Orb web | Viscous | 3 | 10 |
| *Araneus diadematus^1^* | Juve Male | 5 | PLS | Flagelliform | 1 | Web | Orb web | Viscous | 3 | 10 |
| *Araneus diadematus^1^* | Juve Male | 5 | PLS | Cylindrical | 0 | Web | Orb web | Viscous | 3 | 10 |
| *Araneus diadematus^1^* | Juve Fem | 6 | Cribellum | Cribellar | NA | Web | Orb web | Viscous | 3 | 10 |
| *Araneus diadematus^1^* | Juve Fem | 6 | ALS | MAP | 2 | Web | Orb web | Viscous | 3 | 10 |
| *Araneus diadematus^1^* | Juve Fem | 6 | ALS | Piriform | 86 | Web | Orb web | Viscous | 3 | 10 |
| *Araneus diadematus^1^* | Juve Fem | 6 | PMS | mAP | 2 | Web | Orb web | Viscous | 3 | 10 |
| *Araneus diadematus^1^* | Juve Fem | 6 | PMS | Aciniform | 96 | Web | Orb web | Viscous | 3 | 10 |
| *Araneus diadematus^1^* | Juve Fem | 6 | PMS | Cylindrical | 0 | Web | Orb web | Viscous | 3 | 10 |
| *Araneus diadematus^1^* | Juve Fem | 6 | PLS | Aciniform | 114 | Web | Orb web | Viscous | 3 | 10 |
| *Araneus diadematus^1^* | Juve Fem | 6 | PLS | Aggregate | 2 | Web | Orb web | Viscous | 3 | 10 |
| *Araneus diadematus^1^* | Juve Fem | 6 | PLS | Flagelliform | 1 | Web | Orb web | Viscous | 3 | 10 |
| *Araneus diadematus^1^* | Juve Fem | 6 | PLS | Cylindrical | 0 | Web | Orb web | Viscous | 3 | 10 |
| *Araneus diadematus^1^* | Pen Male | 6 | Cribellum | Cribellar | NA | Web | Orb web | Viscous | 3 | 10 |
| *Araneus diadematus^1^* | Pen Male | 6 | ALS | MAP | 2 | Web | Orb web | Viscous | 3 | 10 |
| *Araneus diadematus^1^* | Pen Male | 6 | ALS | Piriform | 95 | Web | Orb web | Viscous | 3 | 10 |
| *Araneus diadematus^1^* | Pen Male | 6 | PMS | mAP | 2 | Web | Orb web | Viscous | 3 | 10 |
| *Araneus diadematus^1^* | Pen Male | 6 | PMS | Aciniform | 73 | Web | Orb web | Viscous | 3 | 10 |
| *Araneus diadematus^1^* | Pen Male | 6 | PMS | Cylindrical | 0 | Web | Orb web | Viscous | 3 | 10 |
| *Araneus diadematus^1^* | Pen Male | 6 | PLS | Aciniform | 91 | Web | Orb web | Viscous | 3 | 10 |
| *Araneus diadematus^1^* | Pen Male | 6 | PLS | Aggregate | 2 | Web | Orb web | Viscous | 3 | 10 |
| *Araneus diadematus^1^* | Pen Male | 6 | PLS | Flagelliform | 1 | Web | Orb web | Viscous | 3 | 10 |
| *Araneus diadematus^1^* | Pen Male | 6 | PLS | Cylindrical | 0 | Web | Orb web | Viscous | 3 | 10 |
| *Araneus diadematus^1^* | Juve Fem | 7 | Cribellum | Cribellar | NA | Web | Orb web | Viscous | 3 | 10 |
| *Araneus diadematus^1^* | Juve Fem | 7 | ALS | MAP | 2 | Web | Orb web | Viscous | 3 | 10 |
| *Araneus diadematus^1^* | Juve Fem | 7 | ALS | Piriform | 92 | Web | Orb web | Viscous | 3 | 10 |
| *Araneus diadematus^1^* | Juve Fem | 7 | PMS | mAP | 2 | Web | Orb web | Viscous | 3 | 10 |
| *Araneus diadematus^1^* | Juve Fem | 7 | PMS | Aciniform | 111 | Web | Orb web | Viscous | 3 | 10 |
| *Araneus diadematus^1^* | Juve Fem | 7 | PMS | Cylindrical | 0 | Web | Orb web | Viscous | 3 | 10 |
| *Araneus diadematus^1^* | Juve Fem | 7 | PLS | Aciniform | 128 | Web | Orb web | Viscous | 3 | 10 |
| *Araneus diadematus^1^* | Juve Fem | 7 | PLS | Aggregate | 2 | Web | Orb web | Viscous | 3 | 10 |
| *Araneus diadematus^1^* | Juve Fem | 7 | PLS | Flagelliform | 1 | Web | Orb web | Viscous | 3 | 10 |
| *Araneus diadematus^1^* | Juve Fem | 7 | PLS | Cylindrical | 0 | Web | Orb web | Viscous | 3 | 10 |
| *Araneus diadematus^1^* | Male | 7 | Cribellum | Cribellar | NA | Web | Orb web | Viscous | 3 | 10 |
| *Araneus diadematus^1^* | Male | 7 | ALS | MAP | 1 | Web | Orb web | Viscous | 3 | 10 |
| *Araneus diadematus^1^* | Male | 7 | ALS | Piriform | 78 | Web | Orb web | Viscous | 3 | 10 |
| *Araneus diadematus^1^* | Male | 7 | PMS | mAP | 1 | Web | Orb web | Viscous | 3 | 10 |
| *Araneus diadematus^1^* | Male | 7 | PMS | Aciniform | 70 | Web | Orb web | Viscous | 3 | 10 |
| *Araneus diadematus^1^* | Male | 7 | PMS | Cylindrical | 0 | Web | Orb web | Viscous | 3 | 10 |
| *Araneus diadematus^1^* | Male | 7 | PLS | Aciniform | 78 | Web | Orb web | Viscous | 3 | 10 |
| *Araneus diadematus^1^* | Male | 7 | PLS | Aggregate | 0 | Web | Orb web | Viscous | 3 | 10 |
| *Araneus diadematus^1^* | Male | 7 | PLS | Flagelliform | 0 | Web | Orb web | Viscous | 3 | 10 |
| *Araneus diadematus^1^* | Male | 7 | PLS | Cylindrical | 0 | Web | Orb web | Viscous | 3 | 10 |
| *Araneus diadematus^1^* | Ante Pen Fem | 8 | Cribellum | Cribellar | NA | Web | Orb web | Viscous | 3 | 10 |
| *Araneus diadematus^1^* | Ante Pen Fem | 8 | ALS | MAP | 2 | Web | Orb web | Viscous | 3 | 10 |
| *Araneus diadematus^1^* | Ante Pen Fem | 8 | ALS | Piriform | 106 | Web | Orb web | Viscous | 3 | 10 |
| *Araneus diadematus^1^* | Ante Pen Fem | 8 | PMS | mAP | 2 | Web | Orb web | Viscous | 3 | 10 |
| *Araneus diadematus^1^* | Ante Pen Fem | 8 | PMS | Aciniform | 142 | Web | Orb web | Viscous | 3 | 10 |
| *Araneus diadematus^1^* | Ante Pen Fem | 8 | PMS | Cylindrical | 0 | Web | Orb web | Viscous | 3 | 10 |
| *Araneus diadematus^1^* | Ante Pen Fem | 8 | PLS | Aciniform | 150 | Web | Orb web | Viscous | 3 | 10 |
| *Araneus diadematus^1^* | Ante Pen Fem | 8 | PLS | Aggregate | 2 | Web | Orb web | Viscous | 3 | 10 |
| *Araneus diadematus^1^* | Ante Pen Fem | 8 | PLS | Flagelliform | 1 | Web | Orb web | Viscous | 3 | 10 |
| *Araneus diadematus^1^* | Ante Pen Fem | 8 | PLS | Cylindrical | 0 | Web | Orb web | Viscous | 3 | 10 |
| *Araneus diadematus^1^* | Pen Fem | 9 | Cribellum | Cribellar | NA | Web | Orb web | Viscous | 3 | 10 |
| *Araneus diadematus^1^* | Pen Fem | 9 | ALS | MAP | 2 | Web | Orb web | Viscous | 3 | 10 |
| *Araneus diadematus^1^* | Pen Fem | 9 | ALS | Piriform | 118 | Web | Orb web | Viscous | 3 | 10 |
| *Araneus diadematus^1^* | Pen Fem | 9 | PMS | mAP | 2 | Web | Orb web | Viscous | 3 | 10 |
| *Araneus diadematus^1^* | Pen Fem | 9 | PMS | Aciniform | 162 | Web | Orb web | Viscous | 3 | 10 |
| *Araneus diadematus^1^* | Pen Fem | 9 | PMS | Cylindrical | 0 | Web | Orb web | Viscous | 3 | 10 |
| *Araneus diadematus^1^* | Pen Fem | 9 | PLS | Aciniform | 170 | Web | Orb web | Viscous | 3 | 10 |
| *Araneus diadematus^1^* | Pen Fem | 9 | PLS | Aggregate | 2 | Web | Orb web | Viscous | 3 | 10 |
| *Araneus diadematus^1^* | Pen Fem | 9 | PLS | Flagelliform | 1 | Web | Orb web | Viscous | 3 | 10 |
| *Araneus diadematus^1^* | Pen Fem | 9 | PLS | Cylindrical | 0 | Web | Orb web | Viscous | 3 | 10 |
| *Araneus diadematus^1^* | Female | 10 | Cribellum | Cribellar | NA | Web | Orb web | Viscous | 3 | 10 |
| *Araneus diadematus^1^* | Female | 10 | ALS | MAP | 1 | Web | Orb web | Viscous | 3 | 10 |
| *Araneus diadematus^1^* | Female | 10 | ALS | Piriform | 120 | Web | Orb web | Viscous | 3 | 10 |
| *Araneus diadematus^1^* | Female | 10 | PMS | mAP | 1 | Web | Orb web | Viscous | 3 | 10 |
| *Araneus diadematus^1^* | Female | 10 | PMS | Aciniform | 166 | Web | Orb web | Viscous | 3 | 10 |
| *Araneus diadematus^1^* | Female | 10 | PMS | Cylindrical | 1 | Web | Orb web | Viscous | 3 | 10 |
| *Araneus diadematus^1^* | Female | 10 | PLS | Aciniform | 174 | Web | Orb web | Viscous | 3 | 10 |
| *Araneus diadematus^1^* | Female | 10 | PLS | Aggregate | 2 | Web | Orb web | Viscous | 3 | 10 |
| *Araneus diadematus^1^* | Female | 10 | PLS | Flagelliform | 1 | Web | Orb web | Viscous | 3 | 10 |
| *Araneus diadematus^1^* | Female | 10 | PLS | Cylindrical | 2 | Web | Orb web | Viscous | 3 | 10 |
| *Larinioides cornutus^5^* | NA | 2 | Cribellum | Cribellar | NA | Web | Orb web | Viscous | 3 | 7 |
| *Larinioides cornutus^5^* | NA | 2 | ALS | MAP | 2 | Web | Orb web | Viscous | 3 | 7 |
| *Larinioides cornutus^5^* | NA | 2 | ALS | Piriform | 9 | Web | Orb web | Viscous | 3 | 7 |
| *Larinioides cornutus^5^* | NA | 2 | PMS | mAP | 2 | Web | Orb web | Viscous | 3 | 7 |
| *Larinioides cornutus^5^* | NA | 2 | PMS | Aciniform | 2 | Web | Orb web | Viscous | 3 | 7 |
| *Larinioides cornutus^5^* | NA | 2 | PMS | Cylindrical | 0 | Web | Orb web | Viscous | 3 | 7 |
| *Larinioides cornutus^5^* | NA | 2 | PLS | Aciniform | 3 | Web | Orb web | Viscous | 3 | 7 |
| *Larinioides cornutus^5^* | NA | 2 | PLS | Flagelliform | 1 | Web | Orb web | Viscous | 3 | 7 |
| *Larinioides cornutus^5^* | NA | 2 | PLS | Aggregate | 2 | Web | Orb web | Viscous | 3 | 7 |
| *Larinioides cornutus^5^* | NA | 2 | PLS | Cylindrical | 0 | Web | Orb web | Viscous | 3 | 7 |
| *Larinioides cornutus^5^* | NA | 3 | Cribellum | Cribellar | NA | Web | Orb web | Viscous | 3 | 7 |
| *Larinioides cornutus^5^* | NA | 3 | ALS | MAP | 2 | Web | Orb web | Viscous | 3 | 7 |
| *Larinioides cornutus^5^* | NA | 3 | ALS | Piriform | 16 | Web | Orb web | Viscous | 3 | 7 |
| *Larinioides cornutus^5^* | NA | 3 | PMS | mAP | 2 | Web | Orb web | Viscous | 3 | 7 |
| *Larinioides cornutus^5^* | NA | 3 | PMS | Aciniform | 6 | Web | Orb web | Viscous | 3 | 7 |
| *Larinioides cornutus^5^* | NA | 3 | PMS | Cylindrical | 0 | Web | Orb web | Viscous | 3 | 7 |
| *Larinioides cornutus^5^* | NA | 3 | PLS | Aciniform | 8 | Web | Orb web | Viscous | 3 | 7 |
| *Larinioides cornutus^5^* | NA | 3 | PLS | Flagelliform | 1 | Web | Orb web | Viscous | 3 | 7 |
| *Larinioides cornutus^5^* | NA | 3 | PLS | Aggregate | 2 | Web | Orb web | Viscous | 3 | 7 |
| *Larinioides cornutus^5^* | NA | 3 | PLS | Cylindrical | 0 | Web | Orb web | Viscous | 3 | 7 |
| *Larinioides cornutus^5^* | Ante Pen Fem | 4 | Cribellum | Cribellar | NA | Web | Orb web | Viscous | 3 | 7 |
| *Larinioides cornutus^5^* | Ante Pen Fem | 4 | ALS | MAP | 2 | Web | Orb web | Viscous | 3 | 7 |
| *Larinioides cornutus^5^* | Ante Pen Fem | 4 | ALS | Piriform | 41 | Web | Orb web | Viscous | 3 | 7 |
| *Larinioides cornutus^5^* | Ante Pen Fem | 4 | PMS | mAP | 2 | Web | Orb web | Viscous | 3 | 7 |
| *Larinioides cornutus^5^* | Ante Pen Fem | 4 | PMS | Aciniform | 7 | Web | Orb web | Viscous | 3 | 7 |
| *Larinioides cornutus^5^* | Ante Pen Fem | 4 | PMS | Cylindrical | 1 | Web | Orb web | Viscous | 3 | 7 |
| *Larinioides cornutus^5^* | Ante Pen Fem | 4 | PLS | Aciniform | 29 | Web | Orb web | Viscous | 3 | 7 |
| *Larinioides cornutus^5^* | Ante Pen Fem | 4 | PLS | Flagelliform | 1 | Web | Orb web | Viscous | 3 | 7 |
| *Larinioides cornutus^5^* | Ante Pen Fem | 4 | PLS | Aggregate | 2 | Web | Orb web | Viscous | 3 | 7 |
| *Larinioides cornutus^5^* | Ante Pen Fem | 4 | PLS | Cylindrical | 2 | Web | Orb web | Viscous | 3 | 7 |
| *Larinioides cornutus^5^* | Male | 4 | Cribellum | Cribellar | NA | Web | Orb web | Viscous | 3 | 7 |
| *Larinioides cornutus^5^* | Male | 4 | ALS | MAP | 2 | Web | Orb web | Viscous | 3 | 7 |
| *Larinioides cornutus^5^* | Male | 4 | ALS | Piriform | 47 | Web | Orb web | Viscous | 3 | 7 |
| *Larinioides cornutus^5^* | Male | 4 | PMS | mAP | 2 | Web | Orb web | Viscous | 3 | 7 |
| *Larinioides cornutus^5^* | Male | 4 | PMS | Aciniform | 10 | Web | Orb web | Viscous | 3 | 7 |
| *Larinioides cornutus^5^* | Male | 4 | PMS | Cylindrical | 0 | Web | Orb web | Viscous | 3 | 7 |
| *Larinioides cornutus^5^* | Male | 4 | PLS | Aciniform | 27 | Web | Orb web | Viscous | 3 | 7 |
| *Larinioides cornutus^5^* | Male | 4 | PLS | Flagelliform | 1 | Web | Orb web | Viscous | 3 | 7 |
| *Larinioides cornutus^5^* | Male | 4 | PLS | Aggregate | 2 | Web | Orb web | Viscous | 3 | 7 |
| *Larinioides cornutus^5^* | Male | 4 | PLS | Cylindrical | 0 | Web | Orb web | Viscous | 3 | 7 |
| *Larinioides cornutus^5^* | Pen Fem | 5 | Cribellum | Cribellar | NA | Web | Orb web | Viscous | 3 | 7 |
| *Larinioides cornutus^5^* | Pen Fem | 5 | ALS | MAP | 2 | Web | Orb web | Viscous | 3 | 7 |
| *Larinioides cornutus^5^* | Pen Fem | 5 | ALS | Piriform | 68 | Web | Orb web | Viscous | 3 | 7 |
| *Larinioides cornutus^5^* | Pen Fem | 5 | PMS | mAP | 2 | Web | Orb web | Viscous | 3 | 7 |
| *Larinioides cornutus^5^* | Pen Fem | 5 | PMS | Aciniform | 14 | Web | Orb web | Viscous | 3 | 7 |
| *Larinioides cornutus^5^* | Pen Fem | 5 | PMS | Cylindrical | 1 | Web | Orb web | Viscous | 3 | 7 |
| *Larinioides cornutus^5^* | Pen Fem | 5 | PLS | Aciniform | 43 | Web | Orb web | Viscous | 3 | 7 |
| *Larinioides cornutus^5^* | Pen Fem | 5 | PLS | Flagelliform | 1 | Web | Orb web | Viscous | 3 | 7 |
| *Larinioides cornutus^5^* | Pen Fem | 5 | PLS | Aggregate | 2 | Web | Orb web | Viscous | 3 | 7 |
| *Larinioides cornutus^5^* | Pen Fem | 5 | PLS | Cylindrical | 2 | Web | Orb web | Viscous | 3 | 7 |
| *Larinioides cornutus^5^* | Female | 6 | Cribellum | Cribellar | NA | Web | Orb web | Viscous | 3 | 7 |
| *Larinioides cornutus^5^* | Female | 6 | ALS | MAP | 1 | Web | Orb web | Viscous | 3 | 7 |
| *Larinioides cornutus^5^* | Female | 6 | ALS | Piriform | 110 | Web | Orb web | Viscous | 3 | 7 |
| *Larinioides cornutus^5^* | Female | 6 | PMS | mAP | 1 | Web | Orb web | Viscous | 3 | 7 |
| *Larinioides cornutus^5^* | Female | 6 | PMS | Aciniform | 21 | Web | Orb web | Viscous | 3 | 7 |
| *Larinioides cornutus^5^* | Female | 6 | PMS | Cylindrical | 1 | Web | Orb web | Viscous | 3 | 7 |
| *Larinioides cornutus^5^* | Female | 6 | PLS | Aciniform | 59 | Web | Orb web | Viscous | 3 | 7 |
| *Larinioides cornutus^5^* | Female | 6 | PLS | Flagelliform | 1 | Web | Orb web | Viscous | 3 | 7 |
| *Larinioides cornutus^5^* | Female | 6 | PLS | Aggregate | 2 | Web | Orb web | Viscous | 3 | 7 |
| *Larinioides cornutus^5^* | Female | 6 | PLS | Cylindrical | 2 | Web | Orb web | Viscous | 3 | 7 |
| *Larinioides cornutus^5^* | Female | 7 | Cribellum | Cribellar | NA | Web | Orb web | Viscous | 3 | 7 |
| *Larinioides cornutus^5^* | Female | 7 | ALS | MAP | 1 | Web | Orb web | Viscous | 3 | 7 |
| *Larinioides cornutus^5^* | Female | 7 | ALS | Piriform | 124 | Web | Orb web | Viscous | 3 | 7 |
| *Larinioides cornutus^5^* | Female | 7 | PMS | mAP | 1 | Web | Orb web | Viscous | 3 | 7 |
| *Larinioides cornutus^5^* | Female | 7 | PMS | Aciniform | 20 | Web | Orb web | Viscous | 3 | 7 |
| *Larinioides cornutus^5^* | Female | 7 | PMS | Cylindrical | 1 | Web | Orb web | Viscous | 3 | 7 |
| *Larinioides cornutus^5^* | Female | 7 | PLS | Aciniform | 71 | Web | Orb web | Viscous | 3 | 7 |
| *Larinioides cornutus^5^* | Female | 7 | PLS | Flagelliform | 1 | Web | Orb web | Viscous | 3 | 7 |
| *Larinioides cornutus^5^* | Female | 7 | PLS | Aggregate | 2 | Web | Orb web | Viscous | 3 | 7 |
| *Larinioides cornutus^5^* | Female | 7 | PLS | Cylindrical | 2 | Web | Orb web | Viscous | 3 | 7 |
| *Enoplognatha ovata^1^* | NA | 2 | Cribellum | Cribellar | NA | Web | Tangle web | Viscous | 3 | 4 |
| *Enoplognatha ovata^1^* | NA | 2 | ALS | MAP | 2 | Web | Tangle web | Viscous | 3 | 4 |
| *Enoplognatha ovata^1^* | NA | 2 | ALS | Piriform | 18 | Web | Tangle web | Viscous | 3 | 4 |
| *Enoplognatha ovata^1^* | NA | 2 | PMS | mAP | 1 | Web | Tangle web | Viscous | 3 | 4 |
| *Enoplognatha ovata^1^* | NA | 2 | PMS | Aciniform | 2 | Web | Tangle web | Viscous | 3 | 4 |
| *Enoplognatha ovata^1^* | NA | 2 | PMS | Cylindrical | 0 | Web | Tangle web | Viscous | 3 | 4 |
| *Enoplognatha ovata^1^* | NA | 2 | PLS | Aciniform | 6 | Web | Tangle web | Viscous | 3 | 4 |
| *Enoplognatha ovata^1^* | NA | 2 | PLS | Aggregate | 2 | Web | Tangle web | Viscous | 3 | 4 |
| *Enoplognatha ovata^1^* | NA | 2 | PLS | Flagelliform | 1 | Web | Tangle web | Viscous | 3 | 4 |
| *Enoplognatha ovata^1^* | NA | 2 | PLS | Cylindrical | 0 | Web | Tangle web | Viscous | 3 | 4 |
| *Enoplognatha ovata^1^* | NA | 3 | Cribellum | Cribellar | NA | Web | Tangle web | Viscous | 3 | 4 |
| *Enoplognatha ovata^1^* | NA | 3 | ALS | MAP | 2 | Web | Tangle web | Viscous | 3 | 4 |
| *Enoplognatha ovata^1^* | NA | 3 | ALS | Piriform | 24 | Web | Tangle web | Viscous | 3 | 4 |
| *Enoplognatha ovata^1^* | NA | 3 | PMS | mAP | 1 | Web | Tangle web | Viscous | 3 | 4 |
| *Enoplognatha ovata^1^* | NA | 3 | PMS | Aciniform | 2 | Web | Tangle web | Viscous | 3 | 4 |
| *Enoplognatha ovata^1^* | NA | 3 | PMS | Cylindrical | 0 | Web | Tangle web | Viscous | 3 | 4 |
| *Enoplognatha ovata^1^* | NA | 3 | PLS | Aciniform | 8 | Web | Tangle web | Viscous | 3 | 4 |
| *Enoplognatha ovata^1^* | NA | 3 | PLS | Aggregate | 2 | Web | Tangle web | Viscous | 3 | 4 |
| *Enoplognatha ovata^1^* | NA | 3 | PLS | Flagelliform | 1 | Web | Tangle web | Viscous | 3 | 4 |
| *Enoplognatha ovata^1^* | NA | 3 | PLS | Cylindrical | 0 | Web | Tangle web | Viscous | 3 | 4 |
| *Enoplognatha ovata^1^* | Male | 4 | Cribellum | Cribellar | NA | Web | Tangle web | Viscous | 3 | 4 |
| *Enoplognatha ovata^1^* | Male | 4 | ALS | MAP | 1 | Web | Tangle web | Viscous | 3 | 4 |
| *Enoplognatha ovata^1^* | Male | 4 | ALS | Piriform | 26 | Web | Tangle web | Viscous | 3 | 4 |
| *Enoplognatha ovata^1^* | Male | 4 | PMS | mAP | 1 | Web | Tangle web | Viscous | 3 | 4 |
| *Enoplognatha ovata^1^* | Male | 4 | PMS | Aciniform | 3 | Web | Tangle web | Viscous | 3 | 4 |
| *Enoplognatha ovata^1^* | Male | 4 | PMS | Cylindrical | 0 | Web | Tangle web | Viscous | 3 | 4 |
| *Enoplognatha ovata^1^* | Male | 4 | PLS | Aciniform | 5 | Web | Tangle web | Viscous | 3 | 4 |
| *Enoplognatha ovata^1^* | Male | 4 | PLS | Aggregate | 0 | Web | Tangle web | Viscous | 3 | 4 |
| *Enoplognatha ovata^1^* | Male | 4 | PLS | Flagelliform | 0 | Web | Tangle web | Viscous | 3 | 4 |
| *Enoplognatha ovata^1^* | Male | 4 | PLS | Cylindrical | 0 | Web | Tangle web | Viscous | 3 | 4 |
| *Enoplognatha ovata^1^* | Female | 4 | Cribellum | Cribellar | NA | Web | Tangle web | Viscous | 3 | 4 |
| *Enoplognatha ovata^1^* | Female | 4 | ALS | MAP | 2 | Web | Tangle web | Viscous | 3 | 4 |
| *Enoplognatha ovata^1^* | Female | 4 | ALS | Piriform | 39 | Web | Tangle web | Viscous | 3 | 4 |
| *Enoplognatha ovata^1^* | Female | 4 | PMS | mAP | 1 | Web | Tangle web | Viscous | 3 | 4 |
| *Enoplognatha ovata^1^* | Female | 4 | PMS | Aciniform | 4 | Web | Tangle web | Viscous | 3 | 4 |
| *Enoplognatha ovata^1^* | Female | 4 | PMS | Cylindrical | 1 | Web | Tangle web | Viscous | 3 | 4 |
| *Enoplognatha ovata^1^* | Female | 4 | PLS | Aciniform | 13 | Web | Tangle web | Viscous | 3 | 4 |
| *Enoplognatha ovata^1^* | Female | 4 | PLS | Aggregate | 2 | Web | Tangle web | Viscous | 3 | 4 |
| *Enoplognatha ovata^1^* | Female | 4 | PLS | Flagelliform | 1 | Web | Tangle web | Viscous | 3 | 4 |
| *Enoplognatha ovata^1^* | Female | 4 | PLS | Cylindrical | 5 | Web | Tangle web | Viscous | 3 | 4 |
