# Supplementary material for: ­Comparative spigot ontogeny across the spider tree of life
Source: PeerJ. 2018 Jan 15;6:e4233. doi: 10.7717/peerj.4233 (PMC5772386; doi:10.7717/peerj.4233)
Supplement: Table S2 — The substitution rate matrices for the four independent variables: Foraging (foraging strategy), Specific (specific foraging strategy), Silk (main type of silk used), and Type (variety of spigot types possessed) used as the models for the ancestral character estimation analyses. The rows are the from direction, while the columns are the to direction for state changes. [file peerj-06-4233-s002.docx]

**Table S2:** The substitution rate matrices for the four independent variables: Foraging (foraging strategy), Specific (specific foraging strategy), Silk (main type of silk used), and Type (variety of spigot types possessed) used as the models for the ancestral character estimation analyses. The rows are the from direction, while the columns are the to direction for state changes.

**Foraging:**

| **Foraging Strategy** | **Web** | **No web (Loss)** |
| --- | --- | --- |
| **Web** | - | 1 |
| **No Web** | 0 | - |

We took a conservative approach to the rate matrices utilizing the AToL as a suggested guide for the rate matrices (Wheeler et al. 2016). We also kept Dollo’s Law (Dollo 1893) in mind as we rated changes from one state to another: we assume that it is easier to lose a web than to re-evolve the use of one, thus we weighted the change from web to no web as 1 and the change from no web to web as 0.

**Specific:**

| **Specific Strategy** | **Sit & Wait** | **Ambush** | **Sit & Pursue** | **Stalking** | **Active** | **Sheet Web** | **Funnel Web** | **Orb Web** | **Tangle Web** |
| --- | --- | --- | --- | --- | --- | --- | --- | --- | --- |
| **Sit & Wait** | - | 1 | 1 | 2 | 2 | 0 | 0 | 0 | 0 |
| **Ambush** | 1 | - | 1 | 2 | 2 | 0 | 0 | 0 | 0 |
| **Sit & Pursue** | 1 | 1 | - | 1 | 1 | 0 | 0 | 0 | 0 |
| **Stalking** | 1 | 1 | 1 | - | 1 | 0 | 0 | 0 | 0 |
| **Active** | 0 | 0 | 1 | 1 | - | 0 | 0 | 0 | 0 |
| **Sheet Web** | 1 | 1 | 2 | 2 | 3 | - | 1 | 1 | 1 |
| **Funnel Web** | 1 | 1 | 0 | 0 | 0 | 1 | - | 0 | 0 |
| **Orb Web** | 1 | 1 | 2 | 2 | 3 | 1 | 1 | - | 1 |
| **Tangle Web** | 0 | 0 | 0 | 0 | 0 | 0 | 0 | 0 | - |

We rated these with the rationale that more energy investment in behavioral change, i.e., from sit & wait to active will take more evolutionary ‘steps.’ Also, we kept the same justification for non-web and web foraging strategies as unlikely to be regained, once lost. Therefore, we assume that web spinning states can take ‘steps’ towards active hunting and that types of webs considered to be more derived (e.g., the cob webs of Theridiidae) take more ‘steps’ to reach.

**Silk:**

| **Silk Type** | **None** | **Burrow** | **Aciniform** | **Cribellate** | **Viscous** |
| --- | --- | --- | --- | --- | --- |
| **None** | - | 0 | 0 | 0 | 0 |
| **Burrow** | 1 | - | 1 | 0 | 0 |
| **Aciniform** | 1 | 1 | - | 0 | 0 |
| **Cribellate** | 1 | 0 | 1 | - | 1 |
| **Viscous** | 1 | 0 | 1 | 0 | - |

Given what we know of the spider phylogeny (Wheeler et al. 2016), we came up with a conservative rate matrix for change from one silk type to another. For example, the loss of cribellate silk use, could move in the direction of the other silk types, while ‘Viscous’ is unlikely to change to cribellate. This variable was specifically representing the main type of silk used for foraging. ‘None’ would be the designation for active hunters, while ‘Burrow’ designates burrowing spiders, ‘Aciniform’ for ecribellate funnel weavers, ‘Cribellate’ for cribellate silk users, and ‘Viscous’ for orb weavers.

**Type:**

| **Variety of Spigot Type** | **Std 7** | **Std 7 + MsPi** | **Std 7 + MS** | **Std 7 + MS + Crib** | **Std 7 + Para + Crib** | **Std 7 + AG + FL** | **Std 7 + Pseudo + Para + Crib** |
| --- | --- | --- | --- | --- | --- | --- | --- |
| **Std 7** | - | 1 | 2 | 2 | 2 | 2 | 2 |
| **Std 7 + MsPi** | 1 | - | 0 | 0 | 0 | 0 | 0 |
| **Std 7 + MS** | 1 | 0 | - | 0 | 0 | 1 | 0 |
| **Std 7 + MS + Crib** | 2 | 0 | 1 | - | 0 | 2 | 0 |
| **Std 7 + Para + Crib** | 2 | 0 | 0 | 1 | - | 3 | 1 |
| **Std 7 + AG + FL** | 1 | 1 | 0 | 0 | 0 | - | 0 |
| **Std 7 + Pseudo + Para + Crib** | 2 | 0 | 2 | 1 | 1 | 2 | - |

This was rated with development of multiple spigot types in mind, thus we assume that the move from ‘Standard 7’ to more complex spigot arrangements takes more steps or that the loss of more structures to gain or transition to others takes more steps. We also allowed for the loss of complexity to return to ‘Standard 7’ to incorporate taxa like Mimetidae, which are derived Araneoidea that actively prey on spiders in the webs of prey; mimetids no longer produce an orb. Mimetids have lost the AG-FL triad in adult instars, but still present vestigial AG-FL spigots in very early instars (Townley & Tillinghast 2009).
