# Supplementary material for: ­Comparative spigot ontogeny across the spider tree of life
Source: PeerJ. 2018 Jan 15;6:e4233. doi: 10.7717/peerj.4233 (PMC5772386; doi:10.7717/peerj.4233)
Supplement: Results S1 — AICc and ΔAICc values for Model Selection for modes of evolution for PGLS Model, using Instar as the predictor variable, as Model Selection found Instar to be most important or significantly most important. [file peerj-06-4233-s003.docx]

**Results S1**: AIC_c_ and ΔAIC_c_ values for Model Selection for modes of evolution for PGLS Model, using Instar as the predictor variable, as Model Selection found Instar to be most important or significantly most important:

Term codes for Evolutionary Rate Model for phylogenetic correlation in PGLS: Brownian (Brownian motion model in Felsenstein 1985) = A, Blomberg (ACDC model; covariance matrix defined in Blomberg et al. 2003) = B, Pagel (covariance matrix defined in Freckleton et al. 2002) = C, Grafen (covariance matrix defined in Grafen 1989) = D, Martins (covariance matrix defined in Martins & Hansen 1997) = E. We did not test an Ornstein-Uhlenbeck model, as it is used for continuous characters, and our predictor variables are all categorical (Graber 2013).

**Significant Results are bolded, if not significant, but most important, they are *bold italicized***

| **Term** | **AIC_C_** | **Δ** | **Weight** |
| --- | --- | --- | --- |
| ***A*** | ***246.23*** | ***0.00*** | ***0.43*** |
| *B* | 246.74 | 0.51 | 0.34 |
| *C* | 247.98 | 1.76 | 0.18 |
| *D* | 251.95 | 5.73 | 0.02 |
| *E* | 251.95 | 5.73 | 0.02 |

References:

Blomberg, S.P., T. Garland, Jr & A.R. Ives. 2003. Testing for phylogenetic signal in

comparative data: behavioral traits are more labile. Evolution 57(4):717–745.

Felsenstein, J. 1985. Phylogenies and the comparative method. The American Naturalist

125:1–15.

Freckleton, R.P., P.H. Harvey & M. Pagel. 2002. Phylogenetic analysis and comparative

data: a test and review of evidence. The American Naturalist 160(6):712 –726.

Grafen, A. 1989. The phylogenetic regression. Philos. Trans. R. Soc. Lond. B. Biol. Sci.

326(1233):119–197.

Martins, E.P. & T.F. Hansen. 1997. Phylogenies and the comparatative method: A

general approach to incorporating phylogenetic information into the Analysis of the

interspecific data. The American Naturalist 149(4):646–667.
