# Supplementary material for: ­Comparative spigot ontogeny across the spider tree of life
Source: PeerJ. 2018 Jan 15;6:e4233. doi: 10.7717/peerj.4233 (PMC5772386; doi:10.7717/peerj.4233)
Supplement: Results S2 — AICc and ΔAICc values for all model iterations for each independent variable, by response variable. [file peerj-06-4233-s004.docx]

**Results S2**: AIC_c_ and ΔAIC_c_ values for all model iterations for each independent variable, by response variable:

**Term codes for Independent Variables: Instar = 1, Type = 2, Silk = 3, Specific = 4, Strategy = 5**

**Significant Results are bolded, if not significant, but most important, they are *bold italicized***

**2^nd^ Instar ALS MAP Model Selection:**

*Single Term PGLS Models:*

| **Term** | **AIC_C_** | **Δ** | **Weight** |
| --- | --- | --- | --- |
| ***Instar*** | **43.35** | **0.00** | **0.84** |
| *Strategy* | 48.75 | 5.40 | 0.06 |
| *Type* | 49.32 | 5.97 | 0.04 |
| *Specific* | 49.35 | 5.99 | 0.04 |
| *Silk* | 50.57 | 7.21 | 0.02 |

*Two Term Models:*

| **Term** | **AIC_C_** | **Δ** | **Weight** |
| --- | --- | --- | --- |
| *14* | 43.64 | 0.00 | 0.40 |
| *15* | 44.38 | 0.74 | 0.28 |
| *13* | 45.86 | 2.21 | 0.13 |
| *12* | 46.18 | 2.54 | 0.11 |
| *35* | 48.22 | 4.58 | 0.04 |
| *24* | 51.38 | 7.74 | 0.01 |
| *25* | 51.51 | 7.87 | 0.01 |
| *45* | 51.74 | 8.10 | 0.01 |
| *34* | 52.28 | 8.64 | 0.01 |
| *23* | 52.29 | 8.65 | 0.01 |

*Three Term Models:*

| **Term** | **AIC_C_** | **Δ** | **Weight** |
| --- | --- | --- | --- |
| *125* | 45.16 | 0.00 | 0.35 |
| *124* | 46.62 | 1.46 | 0.17 |
| *134* | 47.02 | 1.86 | 0.14 |
| *145* | 47.02 | 1.87 | 0.14 |
| *134* | 47.45 | 2.30 | 0.11 |
| *123* | 49.24 | 4.09 | 0.05 |
| *235* | 50.00 | 4.85 | 0.03 |
| *245* | 51.60 | 6.45 | 0.01 |
| *234* | 52.98 | 7.82 | 0.01 |
| *345* | 54.75 | 9.59 | 0.00 |

*Four and Five Term Models:*

| **Term** | **AIC_C_** | **Δ** | **Weight** |
| --- | --- | --- | --- |
| *12345* | 48.39 | 0.00 | 0.30 |
| *1235* | 48.68 | 0.28 | 0.26 |
| *1234* | 48.98 | 0.59 | 0.22 |
| *1245* | 50.28 | 1.88 | 0.12 |
| *1345* | 50.79 | 2.40 | 0.09 |
| *2345* | 53.71 | 5.32 | 0.02 |

**2^nd^ Instar ALS Piriform Model Selection:**

*Single Term PGLS Model:*

| **Term** | **AIC_C_** | **Δ** | **Weight** |
| --- | --- | --- | --- |
| ***1*** | ***205.31*** | ***0.00*** | ***0.37*** |
| *3* | 206.80 | 1.49 | 0.18 |
| *5* | 207.03 | 1.72 | 0.16 |
| *4* | 207.13 | 1.82 | 0.15 |
| *2* | 207.15 | 1.83 | 0.15 |

*Two Term Models:*

| **Term** | **AIC_C_** | **Δ** | **Weight** |
| --- | --- | --- | --- |
| *12* | 207.49 | 0.00 | 0.19 |
| *13* | 207.76 | 0.27 | 0.17 |
| *15* | 207.88 | 0.39 | 0.16 |
| *14* | 208.24 | 0.75 | 0.13 |
| *35* | 209.64 | 2.16 | 0.07 |
| *34* | 209.77 | 2.28 | 0.06 |
| *23* | 209.78 | 2.29 | 0.06 |
| *45* | 210.04 | 2.55 | 0.05 |
| *25* | 210.05 | 2.56 | 0.05 |
| *24* | 210.11 | 2.62 | 0.05 |

*Three Term Models:*

| **Term** | **AIC_C_** | **Δ** | **Weight** |
| --- | --- | --- | --- |
| *123* | 210.86 | 0.00 | 0.14 |
| *135* | 210.87 | 0.01 | 0.14 |
| *134* | 210.89 | 0.02 | 0.14 |
| *124* | 211.04 | 0.18 | 0.13 |
| *145* | 211.10 | 0.24 | 0.13 |
| *125* | 211.15 | 0.28 | 0.13 |
| *235* | 213.00 | 2.14 | 0.05 |
| *245* | 213.05 | 2.18 | 0.05 |
| *234* | 213.09 | 2.23 | 0.05 |
| *345* | 213.44 | 2.58 | 0.04 |

*Four & Five Term Models:*

| **Term** | **AIC_C_** | **Δ** | **Weight** |
| --- | --- | --- | --- |
| *12345* | 204.53 | 0.00 | 0.97 |
| *1345* | 214.67 | 10.14 | 0.01 |
| *1245* | 214.68 | 10.15 | 0.01 |
| *1234* | 214.71 | 10.18 | 0.01 |
| *1235* | 214.87 | 10.34 | 0.01 |
| *2345* | 216.85 | 12.32 | 0.00 |

**2^nd^ Instar ALS Piriform Model Selection:**

*Single Term PGLS Model:*

| **Term** | **AIC_C_** | **Δ** | **Weight** |
| --- | --- | --- | --- |
| ***1*** | ***205.31*** | ***0.00*** | ***0.37*** |
| *3* | 206.80 | 1.49 | 0.18 |
| *5* | 207.03 | 1.72 | 0.16 |
| *4* | 207.13 | 1.82 | 0.15 |
| *2* | 207.15 | 1.83 | 0.15 |

*Two Term Models:*

| **Term** | **AIC_C_** | **Δ** | **Weight** |
| --- | --- | --- | --- |
| *14* | 42.75 | 0.00 | 0.32 |
| *15* | 43.38 | 0.63 | 0.23 |
| *12* | 43.41 | 0.66 | 0.23 |
| *13* | 43.56 | 0.82 | 0.21 |
| *35* | 51.82 | 9.07 | 0.00 |
| *24* | 52.73 | 9.98 | 0.00 |
| *25* | 52.80 | 10.05 | 0.00 |
| *45* | 52.89 | 10.15 | 0.00 |
| *23* | 52.90 | 10.16 | 0.00 |
| *34* | 52.97 | 10.22 | 0.00 |

*Three Term Models:*

| **Term** | **AIC_C_** | **Δ** | **Weight** |
| --- | --- | --- | --- |
| *134* | 45.41 | 0.00 | 0.21 |
| *124* | 45.75 | 0.34 | 0.18 |
| *135* | 45.80 | 0.39 | 0.17 |
| *145* | 45.91 | 0.50 | 0.16 |
| *123* | 46.18 | 0.77 | 0.14 |
| *125* | 46.42 | 1.02 | 0.13 |
| *235* | 54.70 | 9.29 | 0.00 |
| *245* | 55.21 | 9.81 | 0.00 |
| *234* | 55.42 | 10.02 | 0.00 |
| *345* | 56.12 | 10.71 | 0.00 |

*Four & Five Term Models:*

| **Term** | **AIC_C_** | **Δ** | **Weight** |
| --- | --- | --- | --- |
| *12345* | 47.78 | 0.00 | 0.36 |
| *1345* | 49.25 | 1.47 | 0.17 |
| *1245* | 49.26 | 1.48 | 0.17 |
| *1235* | 49.59 | 1.81 | 0.15 |
| *1234* | 49.62 | 1.84 | 0.14 |
| *2345* | 58.49 | 10.71 | 0.00 |

**2^nd^ Instar PMS Aciniform Model Selection:**

*Single Term PGLS Model:*

| **Term** | **AIC_C_** | **Δ** | **Weight** |
| --- | --- | --- | --- |
| ***1*** | ***186.24*** | ***0.00*** | ***0.35*** |
| *3* | 187.75 | 1.51 | 0.16 |
| *5* | 187.75 | 1.51 | 0.16 |
| *2* | 187.75 | 1.51 | 0.16 |
| *4* | 187.75 | 1.51 | 0.16 |

*Two Term Models:*

| **Term** | **AIC_C_** | **Δ** | **Weight** |
| --- | --- | --- | --- |
| *12* | 189.02 | 0.00 | 0.16 |
| *15* | 189.22 | 0.20 | 0.15 |
| *13* | 189.24 | 0.23 | 0.14 |
| *14* | 189.26 | 0.24 | 0.14 |
| *35* | 190.76 | 1.74 | 0.07 |
| *23* | 190.76 | 1.74 | 0.07 |
| *34* | 190.76 | 1.75 | 0.07 |
| *25* | 190.77 | 1.75 | 0.07 |
| *45* | 190.77 | 1.75 | 0.07 |
| *24* | 190.77 | 1.75 | 0.07 |

*Three Term Models:*

| **Term** | **AIC_C_** | **Δ** | **Weight** |
| --- | --- | --- | --- |
| *123* | 192.31 | 0.00 | 0.14 |
| *134* | 192.37 | 0.06 | 0.14 |
| *135* | 192.39 | 0.08 | 0.13 |
| *145* | 192.51 | 0.20 | 0.13 |
| *125* | 192.59 | 0.28 | 0.12 |
| *124* | 192.61 | 0.30 | 0.12 |
| *234* | 194.15 | 1.84 | 0.06 |
| *235* | 194.15 | 1.84 | 0.06 |
| *245* | 194.16 | 1.85 | 0.06 |
| *345* | 194.16 | 1.85 | 0.06 |

*Four & Five Term Models:*

| **Term** | **AIC_C_** | **Δ** | **Weight** |
| --- | --- | --- | --- |
| *12345* | 185.05 | 0.00 | 0.98 |
| *1234* | 196.07 | 11.02 | 0.00 |
| *1245* | 196.16 | 11.11 | 0.00 |
| *1345* | 196.21 | 11.17 | 0.00 |
| *1235* | 196.30 | 11.26 | 0.00 |
| *2345* | 198.00 | 12.95 | 0.00 |

**2^nd^ Instar PLS Aciniform Model Selection:**

*Single Term PGLS Model:*

| **Term** | **AIC_C_** | **Δ** | **Weight** |
| --- | --- | --- | --- |
| ***1*** | ***200.62*** | ***0.00*** | ***0.33*** |
| *3* | 201.99 | 1.37 | 0.17 |
| *4* | 201.99 | 1.37 | 0.17 |
| *5* | 202.00 | 1.38 | 0.17 |
| *2* | 202.05 | 1.43 | 0.16 |

*Two Term Models:*

| **Term** | **AIC_C_** | **Δ** | **Weight** |
| --- | --- | --- | --- |
| *12* | 203.27 | 0.00 | 0.16 |
| *15* | 203.46 | 0.19 | 0.15 |
| *13* | 203.52 | 0.25 | 0.14 |
| *14* | 203.57 | 0.30 | 0.14 |
| *23* | 204.99 | 1.72 | 0.07 |
| *34* | 205.00 | 1.72 | 0.07 |
| *45* | 205.01 | 1.73 | 0.07 |
| *35* | 205.01 | 1.73 | 0.07 |
| *24* | 205.01 | 1.73 | 0.07 |
| *25* | 205.01 | 1.74 | 0.07 |

*Three Term Models:*

| **Term** | **AIC_C_** | **Δ** | **Weight** |
| --- | --- | --- | --- |
| *123* | 206.65 | 0.00 | 0.14 |
| *135* | 206.67 | 0.01 | 0.13 |
| *134* | 206.67 | 0.01 | 0.13 |
| *145* | 206.83 | 0.18 | 0.12 |
| *125* | 206.84 | 0.18 | 0.12 |
| *124* | 206.92 | 0.27 | 0.12 |
| *234* | 208.38 | 1.73 | 0.06 |
| *245* | 208.39 | 1.73 | 0.06 |
| *235* | 208.39 | 1.73 | 0.06 |
| *345* | 208.40 | 1.75 | 0.06 |

*Four & Five Term Models:*

| **Term** | **AIC_C_** | **Δ** | **Weight** |
| --- | --- | --- | --- |
| *12345* | 199.36 | 0.00 | 0.98 |
| *1234* | 210.38 | 11.02 | 0.00 |
| *1245* | 210.47 | 11.11 | 0.00 |
| *1345* | 210.52 | 11.16 | 0.00 |
| *1235* | 210.65 | 11.29 | 0.00 |
| *2345* | 212.23 | 12.87 | 0.00 |

**Female ALS MAP Model Selection:**

*Single Model Terms:*

| **Term** | **AIC_C_** | **Δ** | **Weight** |
| --- | --- | --- | --- |
| ***1*** | ***14.40*** | ***0.00*** | ***0.41*** |
| *2* | 16.37 | 1.96 | 0.16 |
| *4* | 16.52 | 2.12 | 0.14 |
| *5* | 16.52 | 2.12 | 0.14 |
| *3* | 16.52 | 2.12 | 0.14 |

*Two Term Models:*

| **Term** | **AIC_C_** | **Δ** | **Weight** |
| --- | --- | --- | --- |
| *15* | 17.37 | 0.00 | 0.16 |
| *12* | 17.40 | 0.03 | 0.16 |
| *14* | 17.42 | 0.04 | 0.16 |
| *13* | 17.42 | 0.05 | 0.16 |
| *23* | 19.25 | 1.88 | 0.06 |
| *25* | 19.26 | 1.89 | 0.06 |
| *24* | 19.38 | 2.01 | 0.06 |
| *45* | 19.52 | 2.15 | 0.06 |
| *34* | 19.54 | 2.16 | 0.06 |
| *35* | 19.54 | 2.17 | 0.06 |

*Three Term Models:*

| **Term** | **AIC_C_** | **Δ** | **Weight** |
| --- | --- | --- | --- |
| *145* | 20.54 | 0.00 | 0.14 |
| *125* | 20.64 | 0.09 | 0.14 |
| *135* | 20.77 | 0.23 | 0.13 |
| *124* | 20.77 | 0.23 | 0.13 |
| *134* | 20.78 | 0.24 | 0.13 |
| *123* | 20.80 | 0.26 | 0.13 |
| *345* | 22.51 | 1.97 | 0.05 |
| *234* | 22.57 | 2.03 | 0.05 |
| *235* | 22.64 | 2.10 | 0.05 |
| *245* | 22.91 | 2.37 | 0.04 |

*Four & Five Term Models:*

| **Term** | **AIC_C_** | **Δ** | **Weight** |
| --- | --- | --- | --- |
| *12345* | 13.82 | 0.00 | 0.98 |
| *1235* | 24.16 | 10.34 | 0.01 |
| *1345* | 24.36 | 10.54 | 0.01 |
| *1234* | 24.45 | 10.63 | 0.00 |
| *1245* | 24.62 | 10.80 | 0.00 |
| *2345* | 26.34 | 12.52 | 0.00 |

**Female ALS Piriform Model Selection:**

*Single Term PGLS Model:*

| **Term** | **AIC_C_** | **Δ** | **Weight** |
| --- | --- | --- | --- |
| ***1*** | **238.30** | **0.00** | **1.00** |
| *2* | 255.39 | 17.09 | 0.00 |
| *5* | 258.27 | 19.98 | 0.00 |
| *3* | 259.17 | 20.88 | 0.00 |
| *4* | 260.27 | 21.97 | 0.00 |

*Two Term Models:*

| **Term** | **AIC_C_** | **Δ** | **Weight** |
| --- | --- | --- | --- |
| *15* | 238.80 | 0.00 | 0.33 |
| *13* | 238.86 | 0.06 | 0.32 |
| *12* | 239.66 | 0.86 | 0.22 |
| *14* | 240.73 | 1.94 | 0.13 |
| *23* | 258.16 | 19.36 | 0.00 |
| *24* | 258.29 | 19.49 | 0.00 |
| *25* | 258.38 | 19.59 | 0.00 |
| *45* | 259.35 | 20.55 | 0.00 |
| *35* | 260.93 | 22.13 | 0.00 |
| *34* | 261.86 | 23.06 | 0.00 |

*Three Term Models:*

| **Term** | **AIC_C_** | **Δ** | **Weight** |
| --- | --- | --- | --- |
| *145* | 241.32 | 0.00 | 0.24 |
| *124* | 242.01 | 0.69 | 0.17 |
| *135* | 242.03 | 0.71 | 0.17 |
| *125* | 242.12 | 0.81 | 0.16 |
| *123* | 242.18 | 0.86 | 0.16 |
| *134* | 242.93 | 1.62 | 0.11 |
| *235* | 260.07 | 18.75 | 0.00 |
| *345* | 261.11 | 19.80 | 0.00 |
| *234* | 261.55 | 20.24 | 0.00 |
| *245* | 261.98 | 20.66 | 0.00 |

*Four & Five Term Models:*

| **Term** | **AIC_C_** | **Δ** | **Weight** |
| --- | --- | --- | --- |
| *1345* | 245.16 | 0.00 | 0.29 |
| *1235* | 245.16 | 0.00 | 0.29 |
| *1245* | 245.84 | 0.68 | 0.21 |
| *1234* | 245.87 | 0.71 | 0.21 |
| *12345* | 257.72 | 12.56 | 0.00 |
| *2345* | 263.12 | 17.96 | 0.00 |

**Female PMS mAP Model Selection:**

*Single Term PGLS Model:*

| **Term** | **AIC_C_** | **Δ** | **Weight** |
| --- | --- | --- | --- |
| ***5*** | ***-6.06*** | ***0.00*** | ***0.55*** |
| *4* | -3.67 | 2.39 | 0.17 |
| *3* | -3.03 | 3.03 | 0.12 |
| *2* | -2.63 | 3.42 | 0.10 |
| *1* | -1.52 | 4.54 | 0.06 |

*Two Term Models:*

| **Term** | **AIC_C_** | **Δ** | **Weight** |
| --- | --- | --- | --- |
| *35* | -6.83 | 0.00 | 0.57 |
| *15* | -3.49 | 3.34 | 0.11 |
| *45* | -3.17 | 3.66 | 0.09 |
| *25* | -3.11 | 3.72 | 0.09 |
| *24* | -1.13 | 5.70 | 0.03 |
| *34* | -0.75 | 6.08 | 0.03 |
| *14* | -0.74 | 6.09 | 0.03 |
| *12* | -0.29 | 6.54 | 0.02 |
| *13* | -0.17 | 6.66 | 0.02 |
| *23* | -0.13 | 6.70 | 0.02 |

*Three Term Models:*

| **Term** | **AIC_C_** | **Δ** | **Weight** |
| --- | --- | --- | --- |
| *125* | -4.84 | 0.00 | 0.39 |
| *245* | -3.96 | 0.87 | 0.25 |
| *235* | -3.58 | 1.25 | 0.21 |
| *145* | -0.37 | 4.47 | 0.04 |
| *135* | -0.10 | 4.74 | 0.04 |
| *345* | 0.02 | 4.86 | 0.03 |
| *134* | 1.83 | 6.67 | 0.01 |
| *234* | 2.22 | 7.05 | 0.01 |
| *124* | 2.54 | 7.37 | 0.01 |
| *123* | 2.86 | 7.69 | 0.01 |

*Four & Five Term Models:*

| **Term** | **AIC_C_** | **Δ** | **Weight** |
| --- | --- | --- | --- |
| *12345* | -4.12 | 0.00 | 0.51 |
| *1234* | -2.26 | 1.86 | 0.02 |
| *1235* | -2.23 | 1.89 | 0.20 |
| *2345* | -0.13 | 3.99 | 0.07 |
| *1345* | 3.43 | 7.55 | 0.01 |
| *1245* | 5.52 | 9.64 | 0.00 |

**Female PMS Aciniform Model Selection:**

*Single Term PGLS Model:*

| **Term** | **AIC_C_** | **Δ** | **Weight** |
| --- | --- | --- | --- |
| **1** | **258.04** | **0.00** | **0.98** |
| *2* | 268.36 | 10.32 | 0.01 |
| *5* | 268.51 | 10.47 | 0.01 |
| *3* | 268.70 | 10.66 | 0.00 |
| *4* | 268.74 | 10.70 | 0.00 |

*Two Term Models:*

| **Term** | **AIC_C_** | **Δ** | **Weight** |
| --- | --- | --- | --- |
| *14* | 260.65 | 0.00 | 0.27 |
| *12* | 260.82 | 0.17 | 0.25 |
| *13* | 260.86 | 0.22 | 0.24 |
| *15* | 260.95 | 0.30 | 0.23 |
| *25* | 271.32 | 10.67 | 0.00 |
| *24* | 271.33 | 10.68 | 0.00 |
| *23* | 271.38 | 10.73 | 0.00 |
| *35* | 271.44 | 10.79 | 0.00 |
| *45* | 271.51 | 10.86 | 0.00 |
| *34* | 271.70 | 11.05 | 0.00 |

*Three Term Models:*

| **Term** | **AIC_C_** | **Δ** | **Weight** |
| --- | --- | --- | --- |
| *123* | 262.91 | 0.00 | 0.24 |
| *134* | 263.45 | 0.53 | 0.18 |
| *135* | 263.54 | 0.63 | 0.17 |
| *145* | 263.93 | 1.01 | 0.14 |
| *124* | 264.05 | 1.13 | 0.14 |
| *125* | 264.21 | 1.30 | 0.12 |
| *235* | 274.49 | 11.58 | 0.00 |
| *234* | 274.66 | 11.74 | 0.00 |
| *345* | 274.72 | 11.80 | 0.00 |
| *245* | 274.80 | 11.89 | 0.00 |

*Four & Five Term Models:*

| **Term** | **AIC_C_** | **Δ** | **Weight** |
| --- | --- | --- | --- |
| *12345* | 266.23 | 0.00 | 0.27 |
| *1234* | 266.62 | 0.39 | 0.22 |
| *1245* | 266.75 | 0.52 | 0.21 |
| *1345* | 267.26 | 1.03 | 0.16 |
| *1235* | 267.50 | 1.27 | 0.14 |
| *2345* | 278.34 | 12.11 | 0.00 |

**Female PMS Cylindrical Model Selection:**

*Single Term PGLS Model:*

| **Term** | **AIC_C_** | **Δ** | **Weight** |
| --- | --- | --- | --- |
| ***1*** | ***164.46*** | ***0.00*** | ***0.40*** |
| *4* | 165.20 | 0.74 | 0.28 |
| *2* | 166.96 | 2.50 | 0.12 |
| *3* | 167.23 | 2.78 | 0.10 |
| *5* | 167.29 | 2.84 | 0.10 |

*Two Term Models:*

| **Term** | **AIC_C_** | **Δ** | **Weight** |
| --- | --- | --- | --- |
| *14* | 164.90 | 0.00 | 0.27 |
| *24* | 166.27 | 1.37 | 0.14 |
| *45* | 166.51 | 1.62 | 0.12 |
| *15* | 166.89 | 1.99 | 0.10 |
| *13* | 167.03 | 2.14 | 0.09 |
| *12* | 167.45 | 2.56 | 0.08 |
| *34* | 167.62 | 2.72 | 0.07 |
| *23* | 167.79 | 2.89 | 0.06 |
| *25* | 168.37 | 3.48 | 0.05 |
| *35* | 170.25 | 5.36 | 0.02 |

*Three Term Models:*

| **Term** | **AIC_C_** | **Δ** | **Weight** |
| --- | --- | --- | --- |
| *145* | 167.38 | 0.00 | 0.21 |
| *134* | 167.66 | 0.27 | 0.18 |
| *124* | 167.83 | 0.44 | 0.17 |
| *345* | 169.38 | 2.00 | 0.08 |
| *245* | 169.54 | 2.16 | 0.07 |
| *135* | 169.60 | 2.22 | 0.07 |
| *123* | 169.62 | 2.24 | 0.07 |
| *234* | 169.62 | 2.24 | 0.07 |
| *125* | 170.27 | 2.89 | 0.05 |
| *235* | 171.18 | 3.80 | 0.03 |

*Four & Five Term Models:*

| **Term** | **AIC_C_** | **Δ** | **Weight** |
| --- | --- | --- | --- |
| *12345* | 164.83 | 0.00 | 0.87 |
| *1345* | 171.17 | 6.34 | 0.04 |
| *1235* | 171.17 | 6.34 | 0.04 |
| *1245* | 171.48 | 6.64 | 0.03 |
| *2345* | 172.49 | 7.66 | 0.02 |
| *1234* | 173.38 | 8.55 | 0.01 |

**Female PLS Aciniform Model Selection:**

*Single Term PGLS Model:*

| **Term** | **AIC_C_** | **Δ** | **Weight** |
| --- | --- | --- | --- |
| **1** | **251.95** | **0.00** | **0.91** |
| *4* | 259.03 | 7.08 | 0.03 |
| *5* | 259.18 | 7.23 | 0.02 |
| *3* | 259.58 | 7.62 | 0.02 |
| *2* | 259.83 | 7.88 | 0.02 |

*Two Term Models:*

| **Term** | **AIC_C_** | **Δ** | **Weight** |
| --- | --- | --- | --- |
| *14* | 253.50 | 0.00 | 0.35 |
| *12* | 254.35 | 0.85 | 0.23 |
| *15* | 254.55 | 1.06 | 0.02 |
| *13* | 254.62 | 1.13 | 0.20 |
| *35* | 261.92 | 8.43 | 0.01 |
| *45* | 262.02 | 8.53 | 0.00 |
| *34* | 262.04 | 8.55 | 0.00 |
| *24* | 262.05 | 8.56 | 0.00 |
| *25* | 262.13 | 8.63 | 0.00 |
| *23* | 262.59 | 9.09 | 0.00 |

*Three Term Models:*

| **Term** | **AIC_C_** | **Δ** | **Weight** |
| --- | --- | --- | --- |
| *123* | 254.84 | 0.00 | 0.27 |
| *134* | 255.05 | 0.21 | 0.24 |
| *135* | 255.44 | 0.60 | 0.20 |
| *145* | 256.53 | 1.69 | 0.12 |
| *124* | 256.75 | 1.90 | 0.10 |
| *125* | 257.95 | 3.11 | 0.06 |
| *245* | 265.20 | 10.36 | 0.00 |
| *235* | 265.31 | 10.47 | 0.00 |
| *345* | 265.41 | 10.57 | 0.00 |
| *234* | 265.42 | 10.58 | 0.00 |

*Four & Five Term Models:*

| **Term** | **AIC_C_** | **Δ** | **Weight** |
| --- | --- | --- | --- |
| *12345* | 257.24 | 0.00 | 0.36 |
| *1245* | 258.26 | 1.02 | 0.22 |
| *1234* | 258.69 | 1.44 | 0.18 |
| *1345* | 258.80 | 1.55 | 0.17 |
| *1235* | 260.32 | 3.08 | 0.08 |
| *2345* | 269.05 | 11.81 | 0.00 |

**Female PLS Cylindrical Model Selection:**

*Single Term PGLS Model:*

| **Term** | **AIC_C_** | **Δ** | **Weight** |
| --- | --- | --- | --- |
| ***4*** | ***159.12*** | ***0.00*** | ***0.44*** |
| *5* | 161.21 | 2.10 | 0.15 |
| *3* | 161.23 | 2.11 | 0.15 |
| *2* | 161.51 | 2.39 | 0.13 |
| *1* | 161.64 | 2.25 | 0.12 |

*Two Term Models:*

| **Term** | **AIC_C_** | **Δ** | **Weight** |
| --- | --- | --- | --- |
| *45* | 160.95 | 0.00 | 0.22 |
| *24* | 161.21 | 0.26 | 0.19 |
| *34* | 161.66 | 0.71 | 0.15 |
| *14* | 162.13 | 1.19 | 0.12 |
| *23* | 163.03 | 2.09 | 0.08 |
| *25* | 163.23 | 2.28 | 0.07 |
| *35* | 164.22 | 3.28 | 0.04 |
| *15* | 164.23 | 3.29 | 0.04 |
| *13* | 164.24 | 3.30 | 0.04 |
| *12* | 164.49 | 3.55 | 0.04 |

*Three Term Models:*

| **Term** | **AIC_C_** | **Δ** | **Weight** |
| --- | --- | --- | --- |
| *245* | 164.16 | 0.00 | 0.15 |
| *145* | 164.22 | 0.06 | 0.15 |
| *345* | 164.23 | 0.07 | 0.15 |
| *134* | 164.37 | 0.21 | 0.14 |
| *234* | 164.60 | 0.44 | 0.12 |
| *124* | 165.04 | 0.88 | 0.10 |
| *123* | 166.10 | 1.94 | 0.06 |
| *235* | 166.41 | 2.24 | 0.05 |
| *135* | 166.51 | 2.23 | 0.05 |
| *125* | 167.62 | 3.46 | 0.03 |

*Four & Five Term Models:*

| **Term** | **AIC_C_** | **Δ** | **Weight** |
| --- | --- | --- | --- |
| *12345* | 158.94 | 0.00 | 0.95 |
| *2345* | 167.78 | 8.85 | 0.01 |
| *1235* | 167.79 | 8.86 | 0.01 |
| *1345* | 167.85 | 8.91 | 0.01 |
| *1245* | 168.21 | 9.28 | 0.01 |
| *1234* | 169.95 | 11.01 | 0.00 |
