# Supplementary material for: ­Comparative spigot ontogeny across the spider tree of life
Source: PeerJ. 2018 Jan 15;6:e4233. doi: 10.7717/peerj.4233 (PMC5772386; doi:10.7717/peerj.4233)
Supplement: Results S3 — Full results of the PGLS and ANOVA analyses of adult female spigot numbers. [file peerj-06-4233-s005.docx]

**Results S3**: Full results of the PGLS and ANOVA analyses of adult female spigot numbers.

| **Female ALS MAP** | | | |  | **Female ALS Piriform** | | | |  |  |  |  |  |
| --- | --- | --- | --- | --- | --- | --- | --- | --- | --- | --- | --- | --- | --- |
| *Model: Average ~ Strategy* | |  |  |  | *Model: Average ~ Strategy* | |  |  |  |  |  |  |  |
| **PGLS Coefficient:** | | **t-value** | **p-value** |  | **PGLS Coefficient:** | | **t-value** | **p-value** |  |  |  |  |  |
| Strategy | -0.030 | -0.186 | 0.854 |  | Strategy | -72.763 | -1.628 | 0.119 |  |  |  |  |  |
| **ANOVA** |  | **F-value** | **p-value** |  | **ANOVA** |  | **F-value** | **p-value** |  |  |  |  |  |
| Strategy |  | 0.035 | 0.854 |  | Strategy |  | 2.650 | 0.119 |  |  |  |  |  |
|  |  |  |  |  |  |  |  |  |  |  |  |  |  |
| **Female ALS MAP** | | | |  | **Female ALS Piriform** | | | |  |  |  |  |  |
| *Model: Average ~ Instar* | |  |  |  | *Model: Average ~ Instar* | |  |  |  |  |  |  |  |
| **PGLS Coefficient:** | | **t-value** | **p-value** |  | **PGLS Coefficient:** | | **t-value** | **p-value** |  |  |  |  |  |
| Instar | -0.017 | -1.009 | 0.325 |  | Instar | 17.204 | 5.355 | 0.000 |  |  |  |  |  |
| **ANOVA** |  | **F-value** | **p-value** |  | **ANOVA** |  | **F-value** | **p-value** |  |  |  |  |  |
| Instar |  | 1.018 | 0.325 |  | Instar |  | 28.672 | <0.0001 |  |  |  |  |  |
|  |  |  |  |  |  |  |  |  |  |  |  |  |  |
| **Female ALS MAP** | | | |  | **Female ALS Piriform** | | | |  |  |  |  |  |
| *Model: Average ~ Specific* | |  |  |  | *Model: Average ~ Specific* | |  |  |  |  |  |  |  |
| **PGLS Coefficient:** | | **t-value** | **p-value** |  | **PGLS Coefficient:** | | **t-value** | **p-value** |  |  |  |  |  |
| Specific | 0.005 | 0.114 | 0.910 |  | Specific | -4.400 | -0.329 | 0.746 |  |  |  |  |  |
| **ANOVA** |  | **F-value** | **p-value** |  | **ANOVA** |  | **F-value** | **p-value** |  |  |  |  |  |
| Specific |  | 0.013 | 0.910 |  | Specific |  | 0.108 | 0.746 |  |  |  |  |  |
|  |  |  |  |  |  |  |  |  |  |  |  |  |  |
|  |  |  |  |  |  |  |  |  |  |  |  |  |  |
|  |  |  |  |  |  |  |  |  |  |  |  |  |  |
|  |  |  |  |  |  |  |  |  |  |  |  |  |  |
| **Female ALS MAP** | | | |  | **Female ALS Piriform** | | | |  |  |  |  |  |
| *Model: Average ~ Silk* | |  |  |  | *Model: Average ~ Silk* | |  |  |  |  |  |  |  |
| **PGLS Coefficient:** | | **t-value** | **p-value** |  | **PGLS Coefficient:** | | **t-value** | **p-value** |  |  |  |  |  |
| Silk | 0.017 | 0.240 | 0.813 |  | Silk | 24.923 | 1.218 | 0.237 |  |  |  |  |  |
| **ANOVA** |  | **F-value** | **p-value** |  | **ANOVA** |  | **F-value** | **p-value** |  |  |  |  |  |
| Silk |  | 0.058 | 0.813 |  | Silk |  | 1.484 | 0.237 |  |  |  |  |  |
|  |  |  |  |  |  |  |  |  |  |  |  |  |  |
| **Female ALS MAP** | | | |  | **Female ALS Piriform** | | | |  |  |  |  |  |
| *Model: Average ~ Type* | |  |  |  | *Model: Average ~ Type* | |  |  |  |  |  |  |  |
| **PGLS Coefficient:** | | **t-value** | **p-value** |  | **PGLS Coefficient:** | | **t-value** | **p-value** |  |  |  |  |  |
| Type | -0.005 | -0.080 | 0.937 |  | Type | 61.692 | 4.413 | 0.0003 |  |  |  |  |  |
| **ANOVA** |  | **F-value** | **p-value** |  | **ANOVA** |  | **F-value** | **p-value** |  |  |  |  |  |
| Type |  | 0.006 | 0.937 |  | Type |  | 19.471 | 0.0003 |  |  |  |  |  |
|  |  |  |  |  |  |  |  |  |  |  |  |  |  |
|  |  |  |  |  |  |  |  |  |  |  |  |  |  |
| **Female PMS mAP** | | | |  | **Female PMS Aciniform** | | | |  | **Female PMS Cylindrical** | | | |
| *Model: Average ~ Strategy* | |  |  |  | *Model: Average ~ Strategy* | |  |  |  | *Model: Average ~ Strategy* | |  |  |
| **PGLS Coefficient:** | | **t-value** | **p-value** |  | **PGLS Coefficient:** | | **t-value** | **p-value** |  | **PGLS Coefficient:** | | **t-value** | **p-value** |
| Strategy | 0.291 | 2.448 | 0.024 |  | Strategy | -42.401 | -0.937 | 0.360 |  | Strategy | 4.214 | 0.532 | 0.600 |
| **ANOVA** |  | **F-value** | **p-value** |  | **ANOVA** |  | **F-value** | **p-value** |  | **ANOVA** |  | **F-value** | **p-value** |
| Strategy |  | 5.994 | 0.024 |  | Strategy |  | 0.879 | 0.360 |  | Strategy |  | 0.283 | 0.600 |
|  |  |  |  |  |  |  |  |  |  |  |  |  |  |
|  | | | |  |  | | | |  |  | | | |
|  | | | |  |  | | | |  |  | | | |
|  | | | |  |  | | | |  |  | | | |
| **Female PMS mAP** | | | |  | **Female PMS Aciniform** | | | |  | **Female PMS Cylindrical** | | | |
| *Model: Average ~ Instar* | |  |  |  | *Model: Average ~ Instar* | |  |  |  | *Model: Average ~ Instar* | |  |  |
| **PGLS Coefficient:** | | **t-value** | **p-value** |  | **PGLS Coefficient:** | | **t-value** | **p-value** |  | **PGLS Coefficient:** | | **t-value** | **p-value** |
| Instar | 0.005 | 0.342 | 0.736 |  | Instar | 11.725 | 2.857 | 0.010 |  | Instar | 1.388 | 1.805 | 0.086 |
| **ANOVA** |  | **F-value** | **p-value** |  | **ANOVA** |  | **F-value** | **p-value** |  | **ANOVA** |  | **F-value** | **p-value** |
| Instar |  | 0.117 | 0.736 |  | Instar |  | 8.161 | 0.010 |  | Instar |  | 3.259 | 0.086 |
|  |  |  |  |  |  |  |  |  |  |  |  |  |  |
| **Female PMS mAP** | | | |  | **Female PMS Aciniform** | | | |  | **Female PMS Cylindrical** | | | |
| *Model: Average ~ Specific* | |  |  |  | *Model: Average ~ Specific* | |  |  |  | *Model: Average ~ Specific* | |  |  |
| **PGLS Coefficient:** | | **t-value** | **p-value** |  | **PGLS Coefficient:** | | **t-value** | **p-value** |  | **PGLS Coefficient:** | | **t-value** | **p-value** |
| Specific | -0.052 | -1.434 | 0.167 |  | Specific | 3.661 | 0.281 | 0.782 |  | Specific | -2.429 | -1.112 | 0.279 |
| **ANOVA** |  | **F-value** | **p-value** |  | **ANOVA** |  | **F-value** | **p-value** |  | **ANOVA** |  | **F-value** | **p-value** |
| Specific |  | 2.056 | 0.167 |  | Specific |  | 0.079 | 0.782 |  | Specific |  | 1.237 | 0.279 |
|  |  |  |  |  |  |  |  |  |  |  |  |  |  |
| **Female PMS mAP** | | | |  | **Female PMS Aciniform** | | | |  | **Female PMS Cylindrical** | | | |
| *Model: Average ~ Silk* | |  |  |  | *Model: Average ~ Silk* | |  |  |  | *Model: Average ~ Silk* | |  |  |
| **PGLS Coefficient:** | | **t-value** | **p-value** |  | **PGLS Coefficient:** | | t-value | p-value |  | **PGLS Coefficient:** | | **t-value** | **p-value** |
| Silk | -0.066 | -1.130 | 0.272 |  | Silk | 15.095 | 0.743 | 0.466 |  | Silk | -2.215 | -0.629 | 0.536 |
| **ANOVA** |  | **F-value** | **p-value** |  | **ANOVA** |  | F-value | p-value |  | **ANOVA** |  | **F-value** | **p-value** |
| Silk |  | 1.276 | 0.272 |  | Silk |  | 0.552 | 0.466 |  | Silk |  | 0.396 | 0.536 |
|  |  |  |  |  |  |  |  |  |  |  |  |  |  |
|  | | | |  |  | | | |  |  | | | |
|  | | | |  |  | | | |  |  | | | |
|  | | | |  |  | | | |  |  | | | |
|  | | | |  |  | | | |  |  | | | |
|  | | | |  |  | | | |  |  | | | |
|  | | | |  |  | | | |  |  | | | |
| **Female PMS mAP** | | | |  | **Female PMS Aciniform** | | | |  | **Female PMS Cylindrical** | | | |
| *Model: Average ~ Type* | |  |  |  | *Model: Average ~ Type* | |  |  |  | *Model: Average ~ Type* | |  |  |
| **PGLS Coefficient:** | | **t-value** | **p-value** |  | **PGLS Coefficient:** | | **t-value** | **p-value** |  | **PGLS Coefficient:** | | **t-value** | **p-value** |
| Type | -0.032 | -0.571 | 0.575 |  | Type | 30.317 | 1.699 | 0.105 |  | Type | 1.764 | 0.540 | 0.595 |
| **ANOVA** |  | **F-value** | **p-value** |  | **ANOVA** |  | **F-value** | **p-value** |  | **ANOVA** |  | **F-value** | **p-value** |
| Type |  | 0.326 | 0.575 |  | Type |  | 2.888 | 0.105 |  | Type |  | 0.291 | 0.595 |
|  |  |  |  |  |  |  |  |  |  |  |  |  |  |
|  |  |  |  |  |  |  |  |  |  |  |  |  |  |
| **Female PLS Aciniform** | | | |  | **Female PLS Cylindrical** | | | |  |  |  |  |  |
| *Model: Average ~ Strategy* | |  |  |  | *Model: Average ~ Strategy* | |  |  |  |  |  |  |  |
| **PGLS Coefficient:** | | **t-value** | **p-value** |  | **PGLS Coefficient:** | | **t-value** | **p-value** |  |  |  |  |  |
| Strategy | -43.446 | -1.220 | 0.237 |  | Strategy | 3.689 | 0.497 | 0.624 |  |  |  |  |  |
| **ANOVA** |  | **F-value** | **p-value** |  | **ANOVA** |  | **F-value** | **p-value** |  |  |  |  |  |
| Strategy |  | 1.489 | 0.237 |  | Strategy |  | 0.247 | 0.624 |  |  |  |  |  |
|  |  |  |  |  |  |  |  |  |  |  |  |  |  |
| **Female PLS Aciniform** | | | |  | **Female PLS Cylindrical** | | | |  |  |  |  |  |
| *Model: Average ~ Instar* | |  |  |  | *Model: Average ~ Instar* | |  |  |  |  |  |  |  |
| **PGLS Coefficient:** | | **t-value** | **p-value** |  | **PGLS Coefficient:** | | **t-value** | **p-value** |  |  |  |  |  |
| Instar | 6.909 | 1.966 | 0.063 |  | Instar | -0.786 | -1.040 | 0.311 |  |  |  |  |  |
| **ANOVA** |  | **F-value** | **p-value** |  | **ANOVA** |  | **F-value** | **p-value** |  |  |  |  |  |
| Instar |  | 3.866 | 0.063 |  | Instar |  | 1.081 | 0.311 |  |  |  |  |  |
|  |  |  |  |  |  |  |  |  |  |  |  |  |  |
|  |  |  |  |  |  |  |  |  |  |  |  |  |  |
|  |  |  |  |  |  |  |  |  |  |  |  |  |  |
|  |  |  |  |  |  |  |  |  |  |  |  |  |  |
|  |  |  |  |  |  |  |  |  |  |  |  |  |  |
|  |  |  |  |  |  |  |  |  |  |  |  |  |  |
|  |  |  |  |  |  |  |  |  |  |  |  |  |  |
| **Female PLS Aciniform** | | | |  | **Female PLS Cylindrical** | | | |  |  |  |  |  |
| *Model: Average ~ Specific* | |  |  |  | *Model: Average ~ Specific* | |  |  |  |  |  |  |  |
| **PGLS Coefficient:** | | **t-value** | **p-value** |  | **PGLS Coefficient:** | | **t-value** | **p-value** |  |  |  |  |  |
| Specific | 12.992 | 1.299 | 0.209 |  | Specific | -2.331 | -1.142 | 0.267 |  |  |  |  |  |
| **ANOVA** |  | **F-value** | **p-value** |  | **ANOVA** |  | **F-value** | **p-value** |  |  |  |  |  |
| Specific |  | 1.687 | 0.209 |  | Specific |  | 1.304 | 0.267 |  |  |  |  |  |
|  | | | | | | | | | | | | | |
| **Female PLS Aciniform** | | | |  | **Female PLS Cylindrical** | | | |  |  |  |  |  |
| *Model: Average ~ Silk* | |  |  |  | *Model: Average ~ Silk* | |  |  |  |  |  |  |  |
| **PGLS Coefficient:** | | **t-value** | **p-value** |  | **PGLS Coefficient:** | | **t-value** | **p-value** |  |  |  |  |  |
| Silk | 14.229 | 0.881 | 0.389 |  | Silk | -2.014 | -0.611 | 0.548 |  |  |  |  |  |
| **ANOVA** |  | **F-value** | **p-value** |  | **ANOVA** |  | **F-value** | **p-value** |  |  |  |  |  |
| Silk |  | 0.777 | 0.389 |  | Silk |  | 0.373 | 0.548 |  |  |  |  |  |
|  |  |  |  |  |  |  |  |  |  |  |  |  |  |
| **Female PLS Aciniform** | | | |  | **Female PLS Cylindrical** | | | |  |  |  |  |  |
| *Model: Average ~ Type* | |  |  |  | *Model: Average ~ Type* | |  |  |  |  |  |  |  |
| **PGLS Coefficient:** | | **t-value** | **p-value** |  | **PGLS Coefficient:** | | **t-value** | **p-value** |  |  |  |  |  |
| Type | 5.894 | 0.388 | 0.702 |  | Type | 0.535 | 0.174 | 0.864 |  |  |  |  |  |
| **ANOVA** |  | **F-value** | **p-value** |  | **ANOVA** |  | **F-value** | **p-value** |  |  |  |  |  |
| Type |  | 0.151 | 0.702 |  | Type |  | 0.030 | 0.864 |  |  |  |  |  |
