# Supplementary material for: ­Comparative spigot ontogeny across the spider tree of life
Source: PeerJ. 2018 Jan 15;6:e4233. doi: 10.7717/peerj.4233 (PMC5772386; doi:10.7717/peerj.4233)
Supplement: Results S4 — Full results of the PGLS and ANOVA analyses of second instar spigot numbers. [file peerj-06-4233-s006.docx]

**Results S4**: Full results of the PGLS and ANOVA analyses of second instar spigot numbers.

| **Second Instar ALS MAP** | | | |  | **Second Instar ALS Piriform** | | | |
| --- | --- | --- | --- | --- | --- | --- | --- | --- |
| *Model: Average ~ Strategy* | |  |  |  | *Model: Average ~ Strategy* | |  |  |
| **PGLS Coefficient:** | | **t-value** | **p-value** |  | **PGLS Coefficient:** | | **t-value** | **p-value** |
| Strategy | -0.595 | -1.410 | 0.174 |  | Strategy | -3.029 | -0.287 | 0.777 |
| **ANOVA** |  | **F-value** | **p-value** |  | **ANOVA** |  | **F-value** | **p-value** |
| Strategy |  | 1.989 | 0.174 |  | Strategy |  | 0.083 | 0.777 |
|  |  |  |  |  |  |  |  |  |
| **Second Instar ALS MAP** | | | |  | **Second Instar ALS Piriform** | | | |
| *Model: Average ~ Instar* | |  |  |  | *Model: Average ~ Instar* | |  |  |
| **PGLS Coefficient:** | | **t-value** | **p-value** |  | **PGLS Coefficient:** | | **t-value** | **p-value** |
| Instar | 0.095 | 2.283 | 0.034 |  | Instar | -0.269 | -0.242 | 0.812 |
| **ANOVA** |  | **F-value** | **p-value** |  | **ANOVA** |  | **F-value** | **p-value** |
| Instar |  | 5.213 | 0.034 |  | Instar |  | 0.058 | 0.812 |
|  |  |  |  |  |  |  |  |  |
| **Second Instar ALS MAP** | | | |  | **Second Instar ALS Piriform** | | | |
| *Model: Average ~ Specific* | |  |  |  | *Model: Average ~ Specific* | |  |  |
| **PGLS Coefficient:** | | **t-value** | **p-value** |  | **PGLS Coefficient:** | | **t-value** | **p-value** |
| Specific | 0.166 | 1.394 | 0.179 |  | Specific | 0.196 | 0.066 | 0.948 |
| **ANOVA** |  | **F-value** | **p-value** |  | **ANOVA** |  | **F-value** | **p-value** |
| Specific |  | 1.943 | 0.179 |  | Specific |  | 0.004 | 0.948 |
|  |  |  |  |  |  |  |  |  |
| **Second Instar ALS MAP** | | | |  | **Second Instar ALS Piriform** | | | |
| *Model: Average ~ Silk* | |  |  |  | *Model: Average ~ Silk* | |  |  |
| **PGLS Coefficient:** | | **t-value** | **p-value** |  | **PGLS Coefficient:** | | **t-value** | **p-value** |
| Silk | 0.179 | 0.929 | 0.364 |  | Silk | 2.125 | 0.453 | 0.655 |
| **ANOVA** |  | **F-value** | **p-value** |  | **ANOVA** |  | **F-value** | **p-value** |
| Silk |  | 0.863 | 0.364 |  | Silk |  | 0.206 | 0.655 |
|  |  |  |  |  |  |  |  |  |
|  | | | |  |  | | | |
|  | | | |  |  | | | |
|  | | | |  |  | | | |
| **Second Instar ALS MAP** | | | |  | **Second Instar ALS Piriform** | | | |
| *Model: Average ~ Type* | |  |  |  | *Model: Average ~ Type* | |  |  |
| **PGLS Coefficient:** | | **t-value** | **p-value** |  | **PGLS Coefficient:** | | **t-value** | **p-value** |
| Type | 0.192 | 1.078 | 0.294 |  | Type | 0.758 | 0.174 | 0.864 |
| **ANOVA** |  | **F-value** | **p-value** |  | **ANOVA** |  | **F-value** | **p-value** |
| Type |  | 1.163 | 0.294 |  | Type |  | 0.030 | 0.864 |
|  |  |  |  |  |  |  |  |  |
|  |  |  |  |  |  |  |  |  |
| **Second Instar PMS mAP** | | | |  | **Second Instar PMS Aciniform** | | | |
| *Model: Average ~ Strategy* | |  |  |  | *Model: Average ~ Strategy* | |  |  |
| **PGLS Coefficient:** | | **t-value** | **p-value** |  | **PGLS Coefficient:** | | **t-value** | **p-value** |
| Strategy | -0.260 | -0.608 | 0.550 |  | Strategy | -0.260 | -0.004 | 0.997 |
| **ANOVA** |  | **F-value** | **p-value** |  | **ANOVA** |  | **F-value** | **p-value** |
| Strategy |  | 0.370 | 0.550 |  | Strategy |  | 0.000 | 0.997 |
|  |  |  |  |  |  |  |  |  |
|  |  |  |  |  |  |  |  |  |
| **Second Instar PMS mAP** | | | |  | **Second Instar PMS Aciniform** | | | |
| *Model: Average ~ Instar* | |  |  |  | *Model: Average ~ Instar* | |  |  |
| **PGLS Coefficient:** | | **t-value** | **p-value** |  | **PGLS Coefficient:** | | **t-value** | **p-value** |
| Instar | 0.104 | 2.641 | 0.016 |  | Instar | -0.194 | -0.269 | 0.790 |
| **ANOVA** |  | **F-value** | **p-value** |  | **ANOVA** |  | **F-value** | **p-value** |
| Instar |  | 6.977 | 0.016 |  | Instar |  | 0.073 | 0.790 |
|  |  |  |  |  |  |  |  |  |
| **Second Instar PMS mAP** | | | |  | **Second Instar PMS Aciniform** | | | |
| *Model: Average ~ Specific* | |  |  |  | *Model: Average ~ Specific* | |  |  |
| **PGLS Coefficient:** | | **t-value** | **p-value** |  | **PGLS Coefficient:** | | **t-value** | **p-value** |
| Specific | 0.101 | 0.843 | 0.409 |  | Specific | -0.146 | -0.075 | 0.941 |
| **ANOVA** |  | **F-value** | **p-value** |  | **ANOVA** |  | **F-value** | **p-value** |
| Specific |  | 0.710 | 0.409 |  | Specific |  | 0.006 | 0.941 |
|  |  |  |  |  |  |  |  |  |
|  |  |  |  |  |  |  |  |  |
|  |  |  |  |  |  |  |  |  |
|  |  |  |  |  |  |  |  |  |
| **Second Instar PMS mAP** | | | |  | **Second Instar PMS Aciniform** | | | |
| *Model: Average ~ Silk* | |  |  |  | *Model: Average ~ Silk* | |  |  |
| **PGLS Coefficient:** | | **t-value** | **p-value** |  | **PGLS Coefficient:** | | **t-value** | **p-value** |
| Silk | 0.098 | 0.512 | 0.615 |  | Silk | -0.056 | -0.018 | 0.986 |
| **ANOVA** |  | **F-value** | **p-value** |  | **ANOVA** |  | **F-value** | **p-value** |
| Silk |  | 0.262 | 0.615 |  | Silk |  | 0.0003 | 0.986 |
|  |  |  |  |  |  |  |  |  |
| **Second Instar PMS mAP** | | | |  | **Second Instar PMS Aciniform** | | | |
| *Model: Average ~ Type* | |  |  |  | *Model: Average ~ Type* | |  |  |
| **PGLS Coefficient:** | | **t-value** | **p-value** |  | **PGLS Coefficient:** | | **t-value** | **p-value** |
| Type | 0.133 | 0.759 | 0.457 |  | Type | 0.073 | 0.026 | 0.457 |
| **ANOVA** |  | **F-value** | **p-value** |  | **ANOVA** |  | **F-value** | **p-value** |
| Type |  | 0.576 | 0.457 |  | Type |  | 0.001 | 0.980 |
|  |  |  |  |  |  |  |  |  |
|  |  |  |  |  |  |  |  |  |
| **Second Instar PLS Aciniform** | | | |  |  |  |  |  |
| *Model: Average ~ Strategy* | |  |  |  |  |  |  |  |
| **PGLS Coefficient:** | | **t-value** | **p-value** |  |  |  |  |  |
| Strategy | -1.651 | -0.168 | 0.868 |  |  |  |  |  |
| **ANOVA** |  | **F-value** | **p-value** |  |  |  |  |  |
| Strategy |  | 0.028 | 0.868 |  |  |  |  |  |
|  |  |  |  |  |  |  |  |  |
| **Second Instar PLS Aciniform** | | | |  |  |  |  |  |
| *Model: Average ~ Instar* | |  |  |  |  |  |  |  |
| **PGLS Coefficient:** | | **t-value** | **p-value** |  |  |  |  |  |
| Instar | 0.358 | 0.347 | 0.732 |  |  |  |  |  |
| **ANOVA** |  | **F-value** | **p-value** |  |  |  |  |  |
| Instar |  | 0.120 | 0.732 |  |  |  |  |  |
|  |  |  |  |  |  |  |  |  |
|  |  |  |  |  |  |  |  |  |
|  |  |  |  |  |  |  |  |  |
|  |  |  |  |  |  |  |  |  |
|  |  |  |  |  |  |  |  |  |
| **Second Instar PLS Aciniform** | | | |  |  |  |  |  |
| *Model: Average ~ Specific* | |  |  |  |  |  |  |  |
| **PGLS Coefficient:** | | **t-value** | **p-value** |  |  |  |  |  |
| Specific | 0.301 | 0.109 | 0.915 |  |  |  |  |  |
| **ANOVA** |  | **F-value** | **p-value** |  |  |  |  |  |
| Specific |  | 0.012 | 0.915 |  |  |  |  |  |
|  |  |  |  |  |  |  |  |  |
| **Second Instar PLS Aciniform** | | | |  |  |  |  |  |
| *Model: Average ~ Silk* | |  |  |  |  |  |  |  |
| **PGLS Coefficient:** | | **t-value** | **p-value** |  |  |  |  |  |
| Silk | 0.482 | 0.110 | 0.913 |  |  |  |  |  |
| **ANOVA** |  | **F-value** | **p-value** |  |  |  |  |  |
| Silk |  | 0.012 | 0.913 |  |  |  |  |  |
|  |  |  |  |  |  |  |  |  |
| **Second Instar PLS Aciniform** | | | |  |  |  |  |  |
| *Model: Average ~ Type* | |  |  |  |  |  |  |  |
| **PGLS Coefficient:** | | **t-value** | **p-value** |  |  |  |  |  |
| Type | 0.684 | 0.169 | 0.868 |  |  |  |  |  |
| **ANOVA** |  | **F-value** | **p-value** |  |  |  |  |  |
| Type |  | 0.029 | 0.868 |  |  |  |  |  |
