# Supplementary material for: ­Comparative spigot ontogeny across the spider tree of life
Source: PeerJ. 2018 Jan 15;6:e4233. doi: 10.7717/peerj.4233 (PMC5772386; doi:10.7717/peerj.4233)
Supplement: Results S5 — The scaled likelihoods of each possible character state at each node, starting from the root towards to the tips. These correspond to the pie charts at each node in Fig. 3. Scaled likelihood at the nodes for each of the ancestral character state estimation analyses for the four independent variables: Foraging, Specific, Silk and Type. [file peerj-06-4233-s007.docx]

**Results S5**: The scaled likelihoods of each possible character state at each node, starting from the root towards to the tips. These correspond to the pie charts at each node in Figure 3.

| **Node** | **Flagelliform** | **Loss** | **Modified** | **None** | **Pseudoflagelliform** |
| --- | --- | --- | --- | --- | --- |
| **1** | 0.000 | 0.000 | 0.365 | 0.635 | 0.000 |
| **2** | 0.000 | 0.000 | 0.398 | 0.602 | 0.000 |
| **3** | 0.000 | 0.000 | 0.360 | 0.640 | 0.000 |
| **4** | 0.872 | 0.000 | 0.042 | 0.069 | 0.017 |
| **5** | 0.940 | 0.000 | 0.021 | 0.026 | 0.013 |
| **6** | 0.979 | 0.000 | 0.007 | 0.009 | 0.005 |
| **7** | 0.985 | 0.000 | 0.005 | 0.006 | 0.004 |
| **8** | 0.990 | 0.000 | 0.003 | 0.004 | 0.003 |
| **9** | 0.936 | 0.000 | 0.027 | 0.015 | 0.022 |
| **10** | 0.048 | 0.947 | 0.002 | 0.000 | 0.002 |
| **11** | 0.000 | 0.000 | 0.363 | 0.637 | 0.000 |
| **12** | 0.000 | 0.000 | 0.545 | 0.455 | 0.000 |
| **13** | 0.000 | 0.000 | 0.866 | 0.134 | 0.000 |
| **14** | 0.000 | 0.000 | 0.989 | 0.011 | 0.000 |
| **15** | 0.007 | 0.927 | 0.060 | 0.000 | 0.006 |
| **16** | 0.000 | 0.000 | 0.998 | 0.002 | 0.000 |
| **17** | 0.000 | 0.000 | 1.000 | 0.000 | 0.000 |
| **18** | 0.000 | 0.000 | 1.000 | 0.000 | 0.000 |
| **19** | 0.000 | 0.000 | 1.000 | 0.000 | 0.000 |
| **20** | 0.000 | 0.000 | 1.000 | 0.000 | 0.000 |
| **21** | 0.000 | 0.000 | 1.000 | 0.000 | 0.000 |
| **22** | 0.000 | 0.000 | 1.000 | 0.000 | 0.000 |
| **23** | 0.000 | 0.000 | 1.000 | 0.000 | 0.000 |
| **24** | 0.000 | 0.990 | 0.010 | 0.000 | 0.000 |
| **25** | 0.000 | 0.000 | 0.461 | 0.539 | 0.000 |
| **26** | 0.000 | 0.000 | 0.423 | 0.577 | 0.000 |

Scaled likelihood at the nodes for each of the ancestral character state estimation analyses for the four independent variables: Foraging, Specific, Silk and Type.

**Strategy**:

|  | **Web** | **No web (Loss)** |
| --- | --- | --- |
| **1** | 1.000 | 0.000 |
| **2** | 1.000 | 0.000 |
| **3** | 1.000 | 0.000 |
| **4** | 1.000 | 0.000 |
| **5** | 1.000 | 0.000 |
| **6** | 1.000 | 0.000 |
| **7** | 1.000 | 0.000 |
| **8** | 1.000 | 0.000 |
| **9** | 1.000 | 0.000 |
| **10** | 1.000 | 0.000 |
| **11** | 0.022 | 0.978 |
| **12** | 1.000 | 0.000 |
| **13** | 1.000 | 0.000 |
| **14** | 1.000 | 0.000 |
| **15** | 1.000 | 0.000 |
| **16** | 1.000 | 0.000 |
| **17** | 1.000 | 0.000 |
| **18** | 1.000 | 0.000 |
| **19** | 0.036 | 0.964 |
| **20** | 0.016 | 0.984 |
| **21** | 0.010 | 0.990 |
| **22** | 0.007 | 0.993 |
| **23** | 0.005 | 0.995 |
| **24** | 0.003 | 0.997 |
| **25** | 0.002 | 0.998 |
| **26** | 1.000 | 0.000 |

**Specific**:

|  | **Sit & Wait** | **Ambush** | **Sit & Pursue** | **Stalking** | **Active** | **Sheet Web** | **Funnel Web** | **Orb Web** | **Tangle Web** |
| --- | --- | --- | --- | --- | --- | --- | --- | --- | --- |
| **1** | 0.000 | 0.000 | 0.000 | 0.000 | 0.000 | 0.084 | 0.812 | 0.105 | 0.000 |
| **2** | 0.000 | 0.000 | 0.000 | 0.000 | 0.000 | 0.117 | 0.770 | 0.113 | 0.000 |
| **3** | 0.000 | 0.000 | 0.000 | 0.000 | 0.000 | 0.459 | 0.332 | 0.209 | 0.000 |
| **4** | 0.000 | 0.000 | 0.000 | 0.000 | 0.000 | 0.492 | 0.155 | 0.353 | 0.000 |
| **5** | 0.000 | 0.000 | 0.000 | 0.000 | 0.000 | 0.182 | 0.000 | 0.818 | 0.000 |
| **6** | 0.000 | 0.000 | 0.000 | 0.000 | 0.000 | 0.011 | 0.000 | 0.989 | 0.000 |
| **7** | 0.000 | 0.000 | 0.000 | 0.000 | 0.000 | 0.000 | 0.000 | 1.000 | 0.000 |
| **8** | 0.000 | 0.000 | 0.000 | 0.000 | 0.000 | 0.000 | 0.000 | 1.000 | 0.000 |
| **9** | N/A | N/A | N/A | N/A | N/A | N/A | N/A | N/A | N/A |
| **10** | 0.000 | 0.000 | 0.000 | 0.000 | 0.000 | 0.009 | 0.000 | 0.991 | 0.000 |
| **11** | 0.001 | 0.001 | 0.001 | 0.969 | 0.000 | 0.001 | 0.000 | 0.028 | 0.000 |
| **12** | 0.000 | 0.000 | 0.000 | 0.000 | 0.000 | 0.433 | 0.428 | 0.139 | 0.000 |
| **13** | 0.000 | 0.000 | 0.000 | 0.000 | 0.000 | 0.680 | 0.075 | 0.244 | 0.000 |
| **14** | 0.000 | 0.000 | 0.000 | 0.000 | 0.000 | 0.822 | 0.127 | 0.050 | 0.000 |
| **15** | 0.000 | 0.000 | 0.000 | 0.000 | 0.000 | 0.784 | 0.171 | 0.045 | 0.000 |
| **16** | N/A | N/A | N/A | N/A | N/A | N/A | N/A | N/A | N/A |
| **17** | 0.000 | 0.000 | 0.000 | 0.000 | 0.000 | 0.754 | 0.189 | 0.057 | 0.000 |
| **18** | 0.000 | 0.000 | 0.000 | 0.000 | 0.000 | 0.551 | 0.386 | 0.063 | 0.000 |
| **19** | 0.048 | 0.830 | 0.002 | 0.001 | 0.000 | 0.065 | 0.046 | 0.008 | 0.000 |
| **20** | 0.165 | 0.733 | 0.005 | 0.003 | 0.000 | 0.051 | 0.036 | 0.006 | 0.000 |
| **21** | 0.060 | 0.854 | 0.015 | 0.008 | 0.036 | 0.015 | 0.011 | 0.002 | 0.000 |
| **22** | 0.037 | 0.903 | 0.016 | 0.006 | 0.021 | 0.009 | 0.006 | 0.001 | 0.000 |
| **23** | 0.010 | 0.045 | 0.019 | 0.001 | 0.925 | 0.001 | 0.000 | 0.000 | 0.000 |
| **24** | 0.011 | 0.034 | 0.055 | 0.001 | 0.899 | 0.001 | 0.000 | 0.000 | 0.000 |
| **25** | 0.003 | 0.004 | 0.002 | 0.002 | 0.989 | 0.000 | 0.000 | 0.000 | 0.000 |
| **26** | N/A | N/A | N/A | N/A | N/A | N/A | N/A | N/A | N/A |

**Silk**:

|  | **None** | **Burrow** | **Aciniform** | **Cribellate** | **Viscous** |
| --- | --- | --- | --- | --- | --- |
| **1** | 0.000 | 0.000 | 0.000 | 1.000 | 0.000 |
| **2** | 0.000 | 0.000 | 0.000 | 1.000 | 0.000 |
| **3** | 0.000 | 0.000 | 0.000 | 1.000 | 0.000 |
| **4** | 0.000 | 0.000 | 0.000 | 1.000 | 0.000 |
| **5** | 0.000 | 0.000 | 0.000 | 0.052 | 0.948 |
| **6** | 0.000 | 0.000 | 0.000 | 0.031 | 0.969 |
| **7** | 0.000 | 0.000 | 0.000 | 0.010 | 0.990 |
| **8** | 0.000 | 0.000 | 0.000 | 0.008 | 0.992 |
| **9** | 0.000 | 0.000 | 0.000 | 0.005 | 0.995 |
| **10** | 0.000 | 0.000 | 0.000 | 0.031 | 0.969 |
| **11** | 0.971 | 0.000 | 0.001 | 0.001 | 0.027 |
| **12** | 0.000 | 0.000 | 0.000 | 1.000 | 0.000 |
| **13** | 0.000 | 0.000 | 0.000 | 1.000 | 0.000 |
| **14** | 0.000 | 0.000 | 0.000 | 1.000 | 0.000 |
| **15** | 0.000 | 0.000 | 0.000 | 1.000 | 0.000 |
| **16** | 0.000 | 0.000 | 0.984 | 0.015 | 0.001 |
| **17** | 0.000 | 0.000 | 0.000 | 1.000 | 0.000 |
| **18** | 0.000 | 0.000 | 0.000 | 1.000 | 0.000 |
| **19** | 0.000 | 0.018 | 0.232 | 0.744 | 0.006 |
| **20** | 0.000 | 0.059 | 0.359 | 0.573 | 0.009 |
| **21** | 0.000 | 0.113 | 0.423 | 0.454 | 0.010 |
| **22** | 0.000 | 0.179 | 0.452 | 0.359 | 0.010 |
| **23** | 0.979 | 0.004 | 0.009 | 0.007 | 0.000 |
| **24** | 0.992 | 0.002 | 0.004 | 0.003 | 0.000 |
| **25** | 0.996 | 0.001 | 0.002 | 0.001 | 0.000 |
| **26** | 0.000 | 0.000 | 0.000 | 1.000 | 0.000 |

**Type**:

|  | **Std 7** | **Std 7 + MsPi** | **Std 7 + MS** | **Std 7 + MS + Crib** | **Std 7 + Para + Crib** | **Std 7 + Ag + FL** | **Std 7 + Pseudo + Para + Crib** |
| --- | --- | --- | --- | --- | --- | --- | --- |
| **1** | 0.144 | 0.003 | 0.004 | 0.013 | 0.398 | 0.005 | 0.433 |
| **2** | 0.160 | 0.001 | 0.002 | 0.018 | 0.301 | 0.005 | 0.512 |
| **3** | 0.260 | 0.000 | 0.004 | 0.065 | 0.143 | 0.020 | 0.508 |
| **4** | 0.294 | 0.000 | 0.010 | 0.289 | 0.035 | 0.076 | 0.296 |
| **5** | 0.006 | 0.000 | 0.002 | 0.020 | 0.000 | 0.953 | 0.018 |
| **6** | 0.002 | 0.000 | 0.001 | 0.006 | 0.000 | 0.986 | 0.005 |
| **7** | 0.000 | 0.000 | 0.000 | 0.002 | 0.000 | 0.006 | 0.002 |
| **8** | 0.000 | 0.000 | 0.000 | 0.002 | 0.000 | 0.997 | 0.001 |
| **9** | 0.000 | 0.000 | 0.000 | 0.001 | 0.000 | 0.998 | 0.001 |
| **10** | 0.007 | 0.002 | 0.000 | 0.003 | 0.000 | 0.986 | 0.002 |
| **11** | 0.000 | 0.976 | 0.000 | 0.000 | 0.000 | 0.024 | 0.000 |
| **12** | 0.400 | 0.000 | 0.001 | 0.436 | 0.034 | 0.002 | 0.127 |
| **13** | 0.315 | 0.000 | 0.001 | 0.003 | 0.045 | 0.001 | 0.636 |
| **14** | 0.502 | 0.000 | 0.000 | 0.000 | 0.011 | 0.000 | 0.487 |
| **15** | 0.968 | 0.000 | 0.000 | 0.001 | 0.001 | 0.000 | 0.030 |
| **16** | 0.999 | 0.000 | 0.000 | 0.000 | 0.000 | 0.000 | 0.001 |
| **17** | 0.997 | 0.000 | 0.000 | 0.001 | 0.000 | 0.000 | 0.002 |
| **18** | 0.996 | 0.000 | 0.000 | 0.003 | 0.000 | 0.000 | 0.001 |
| **19** | 1.000 | 0.000 | 0.000 | 0.000 | 0.000 | 0.000 | 0.000 |
| **20** | 0.999 | 0.000 | 0.001 | 0.000 | 0.000 | 0.000 | 0.000 |
| **21** | 1.000 | 0.000 | 0.000 | 0.000 | 0.000 | 0.000 | 0.000 |
| **22** | 1.000 | 0.000 | 0.000 | 0.000 | 0.000 | 0.000 | 0.000 |
| **23** | 1.000 | 0.000 | 0.000 | 0.000 | 0.000 | 0.000 | 0.000 |
| **24** | 0.998 | 0.000 | 0.002 | 0.000 | 0.000 | 0.000 | 0.000 |
| **25** | 1.000 | 0.000 | 0.000 | 0.000 | 0.000 | 0.000 | 0.000 |
| **26** | 0.086 | 0.001 | 0.002 | 0.002 | 0.572 | 0.002 | 0.335 |
