# Supplementary material for: ­Comparative spigot ontogeny across the spider tree of life
Source: PeerJ. 2018 Jan 15;6:e4233. doi: 10.7717/peerj.4233 (PMC5772386; doi:10.7717/peerj.4233)
Supplement: Supplemental Information 1 — All R codes for tests for phylogenetic signal, model selection, PGLS analyses, ACE analyses and creating and labelling the resulting phylograms. [file peerj-06-4233-s008.docx]

**##R codes for all model testing, analyses, tree building and ancestral character estimations**

library(ape)

library(geiger)

library(nlme)

library (phytools)

library(picante)

library(phylobase)

library(adephylo)

setwd("C:/Users/Rachael/Desktop/Comparative Ontogeny")

##Load the spider phylogeny, plot it

SpiderTree <- read.newick ("SToL")

plot(SpiderTree)

##Load the dataset (specific to each spigot for each instar)

library(readxl)

Females <- read_excel("C:/Users/Rachael/Desktop/Comparative Ontogeny/Females.xlsx")

View(Females)

##Name check the tips and species data

rownames(Females) <- Females$Species

Females <- Females[match(SpiderTree$tip.label,rownames(Females)),]

name.check (SpiderTree, Females, data.names = NULL)

str(Females)

Species <- Females$Species

Average <- Females$Average

Strategy <- Females$Foraging_Strategy

Specific <- Females$Specific_Strategy

Type <- Females$Spigot_Type

Silk <- Females$Type_of_Silk

**## Test for phylogenetic signal:**

##Pagel's lambda test for each variable

##Average (continuous)

trait <- Females [,5]

names(trait) <- rownames(Females)

phylosig(SpiderTree, trait, method="lambda", test = TRUE, nsim=999)

##Foraging_Strategy

trait<- Females [,6]

phylosig(SpiderTree, trait, method="lambda", test = TRUE, nsim=999)

##Specific_Strategy

trait<- Females [,7]

phylosig(SpiderTree, trait, method="lambda", test = TRUE, nsim=999)

##Type_of_Silk

trait<- Females [,8]

phylosig(SpiderTree, trait, method="lambda", test = TRUE, nsim=999)

##Spigot_Type

trait <- Females [,9]

phylosig(SpiderTree, trait, method="lambda", test = TRUE, nsim=999)

##Blomberg's K test for each variable

##Average (continuous)

trait <- Females [,5]

phylosig(SpiderTree, trait, method="K", test = TRUE, nsim=999)

##Foraging_Strategy

trait<- Females [,6]

phylosig(SpiderTree, trait, method="K", test = TRUE, nsim=999)

##Specific_Strategy

trait<- Females [,7]

phylosig(SpiderTree, trait, method="K", test = TRUE, nsim=999)

##Type_of_Silk

trait<- Females [,8]

phylosig(SpiderTree, trait, method="K", test = TRUE, nsim=999)

##Spigot_Type

trait <- Females [,9]

phylosig(SpiderTree, trait, method="K", test = TRUE, nsim=999)

**##Code for Model Selection for variables and modes of evolution:**

# call up the following installed packages-----------------

library(nlme)

library(MuMIn)

##Modes of evolution test:

pglsOU <- gls(Average ~ Instar, correlation = corPagel(1, phy = SpiderTree, fixed = FALSE), data = Females, method = "ML")

summary(pglsOU)

anova(pglsOU)

pglsMartins <- gls(Average ~ Instar, correlation = corMartins(1, phy = SpiderTree, fixed = FALSE), data = Females, method = "ML")

summary(pglsMartins)

anova(pglsMartins)

pglsBlomberg <- gls(Average ~ Instar, correlation = corBlomberg(1, phy = SpiderTree, fixed = TRUE), data = Females, method = "ML")

summary(pglsBlomberg)

anova(pglsBlomberg)

pglsGrafen <- gls(Average ~ Instar, correlation = corGrafen(1, phy = SpiderTree, fixed = FALSE), data = Females, method = "ML")

summary(pglsGrafen)

anova(pglsGrafen)

pglsBrownian <- gls(Average ~ Instar, correlation = corBrownian(1, phy = SpiderTree, fixed = FALSE), data = Females, method = "ML")

summary(pglsBrownian)

anova(pglsBrownian)

##Brownian mode of evolution had the best fit, so I then moved on to test the model variables, subsequently finding that single term models had the highest power/best fit leading to the final PGLS codes below.

##These are the variable models tested for each type of spigot (see details below in the PGLS codes).

pgls1 <- gls(Average ~ Instar, correlation = corBrownian(phy = SpiderTree), data = Females, method = "ML")

pgls2 <- gls(Average ~ Type, correlation = corBrownian(phy = SpiderTree), data = Females, method = "ML")

pgls3 <- gls(Average ~ Silk, correlation = corBrownian(phy = SpiderTree), data = Females, method = "ML")

pgls4 <- gls(Average ~ Specific, correlation = corBrownian(phy = SpiderTree), data = Females, method = "ML")

pgls5 <- gls(Average ~ Strategy, correlation = corBrownian(phy = SpiderTree), data = Females, method = "ML")

pgls6 <- gls(Average ~ Instar + Type, correlation = corBrownian(phy = SpiderTree), data = Females, method = "ML")

pgls7 <- gls(Average ~ Instar + Silk, correlation = corBrownian(phy = SpiderTree), data = Females, method = "ML")

pgls8 <- gls(Average ~ Silk + Type, correlation = corBrownian(phy = SpiderTree), data = Females, method = "ML")

pgls9 <- gls(Average ~ Instar + Specific, correlation = corBrownian(phy = SpiderTree), data = Females, method = "ML")

pgls10 <- gls(Average ~ Specific + Type, correlation = corBrownian(phy = SpiderTree), data = Females, method = "ML")

pgls11 <- gls(Average ~ Specific + Silk, correlation = corBrownian(phy = SpiderTree), data = Females, method = "ML")

pgls12 <- gls(Average ~ Instar + Strategy, correlation = corBrownian(phy = SpiderTree), data = Females, method = "ML")

pgls13 <- gls(Average ~ Strategy + Type, correlation = corBrownian(phy = SpiderTree), data = Females, method = "ML")

pgls14 <- gls(Average ~ Strategy + Silk, correlation = corBrownian(phy = SpiderTree), data = Females, method = "ML")

pgls15 <- gls(Average ~ Strategy + Specific, correlation = corBrownian(phy = SpiderTree), data = Females, method = "ML")

pgls16 <- gls(Average ~ Instar + Type + Silk, correlation = corBrownian(phy = SpiderTree), data = Females, method = "ML")

pgls17 <- gls(Average ~ Specific + Type + Instar, correlation = corBrownian(phy = SpiderTree), data = Females, method = "ML")

pgls18 <- gls(Average ~ Instar + Specific + Silk, correlation = corBrownian(phy = SpiderTree), data = Females, method = "ML")

pgls19 <- gls(Average ~ Specific + Type + Silk, correlation = corBrownian(phy = SpiderTree), data = Females, method = "ML")

pgls20 <- gls(Average ~ Instar + Type + Strategy, correlation = corBrownian(phy = SpiderTree), data = Females, method = "ML")

pgls21 <- gls(Average ~ Instar + Strategy + Silk, correlation = corBrownian(phy = SpiderTree), data = Females, method = "ML")

pgls22 <- gls(Average ~ Strategy + Type + Silk, correlation = corBrownian(phy = SpiderTree), data = Females, method = "ML")

pgls23 <- gls(Average ~ Instar + Strategy + Specific, correlation = corBrownian(phy = SpiderTree), data = Females, method = "ML")

pgls24 <- gls(Average ~ Strategy + Specific + Type, correlation = corBrownian(phy = SpiderTree), data = Females, method = "ML")

pgls25 <- gls(Average ~ Strategy + Specific + Silk, correlation = corBrownian(phy = SpiderTree), data = Females, method = "ML")

pgls26 <- gls(Average ~ Instar + Type + Silk + Strategy, correlation = corBrownian(phy = SpiderTree), data = Females, method = "ML")

pgls27 <- gls(Average ~ Instar + Type + Specific + Strategy, correlation = corBrownian(phy = SpiderTree), data = Females, method = "ML")

pgls28 <- gls(Average ~ Instar + Specific + Silk + Strategy, correlation = corBrownian(phy = SpiderTree), data = Females, method = "ML")

pgls29 <- gls(Average ~ Specific + Type + Silk + Strategy, correlation = corBrownian(phy = SpiderTree), data = Females, method = "ML")

pgls30 <- gls(Average ~ Instar + Type + Silk + Specific, correlation = corBrownian(phy = SpiderTree), data = Females, method = "ML")

pgls31 <- gls(Average ~ 1, correlation = corBrownian(phy = SpiderTree), data = Females, method = "ML")

**##Final Code for PGLS analyses**

##Adult female PMS aciniform spigot example

##PGLS with discrete variable, run pgls, then run anova

pglsModel <- gls(Average ~ Strategy, correlation = corBrownian(phy = SpiderTree),

data = Females, method = "ML")

summary(pglsModel)

anova(pglsModel)

pglsModel1 <- gls(Average ~ Instar, correlation = corBrownian(phy = SpiderTree),

data = Females, method = "ML")

summary(pglsModel1)

anova(pglsModel1)

pglsModel2 <- gls(Average ~ Specific, correlation = corBrownian(phy = SpiderTree),

data = Females, method = "ML")

summary(pglsModel2)

anova(pglsModel2)

pglsModel3 <- gls(Average ~ Silk, correlation = corBrownian(phy = SpiderTree),

data = Females, method = "ML")

summary(pglsModel3)

anova(pglsModel3)

pglsModel4 <- gls(Average ~ Type, correlation = corBrownian(phy = SpiderTree),

data = Females, method = "ML")

summary(pglsModel4)

anova(pglsModel4)

##Adult female PMS minor ampullate spigot, female ALS major ampullate spigot, female ALS piriform spigot, female PMS cylindrical spigot, female ALS aciniform spigot, female PLS cylindrical spigot

##Run the data loading code again, but select the corresponding spreadsheet for this dataset

##Run the PGLS codes for each single term model

##Second instar data PGLS runs for 2^nd^ instar ALS major ampullate spigot, 2^nd^ instar ALS piriform spigot, 2^nd^ instar PMS minor ampullate spigot, 2^nd^ instar PMS aciniform spigot, 2^nd^ instar PLS aciniform spigot:

##Run same start and data loading code, except select the second instar excel file and follow through the subsequent spreadsheets for each specific spigot.

##The PGLS code is the same and the renaming strings work, just name the second instar dataset, “Females” in R, so you don’t have to change the code.

**##Converting the Spider Tree (SToL) into an ultrametric tree, rather than one with equal branch lengths**

##Ultrametric tree conversion codes used:

## Quick and dirty method:

> library(ape)

> SpiderTree <- read.newick ("Final SToL")

> plot(SpiderTree)

> is.ultrametric(SpiderTree)

> timetree <- chronos (SpiderTree)

> is.ultrametric(timetree)

> plot(timetree)

> write.tree (timetree, file = "timetreeNewick.tre")

> tr$edge.length

> timetree$edge.length

### Setting tree to 1 and scaling branch lengths in a relaxed model (other easy way to convert to ultrametric with no prior knowledge for calibration (fossil) or a clock model (prior branch lengths/molecular rates of evolution)

> rel_calib1 <- makeChronosCalib(SpiderTree, node = "root", age.min = 1, age.max = 1, interactive = FALSE, soft.bounds = FALSE)

> timetree2 <- chronos(SpiderTree, lambda = 0, model = "relaxed", calibration = rel_calib1)

> plot(timetree2)

> timetree2$edge.length

**##Ancestral character estimation for the specialized spigot on the PLS**

##Final ACE with deeper taxon sampling, change of none to loss to better delineate having none to begin with, versus loosing a spigot

> library(ape)

> library(geiger)

> library(nlme)

> library (phytools)

> setwd("C:/Users/Rachael/Desktop/Comparative Ontogeny")

> SpiderTree <- read.newick ("SpiderTree")

> plot(SpiderTree)

> is.ultrametric (SpiderTree)

> library(readxl)

> Females <- read_excel("C:/Users/Rachael/Desktop/Comparative Ontogeny/PLS.xlsx",

+ sheet = "PLS Other")

> View(Females)

> rownames(Females) <- Females$Species

> Females <- Females[match(SpiderTree$tip.label,rownames(Females)),]

> name.check (SpiderTree, Females, data.names = NULL)

> PLS <- Females$Spigot

> PLS

> fit <- ace(x = PLS, phy = SpiderTree, type = "discrete", method = "ML", CI = TRUE, model = matrix(c(0, 1, 0, 0, 0, 0, 0, 0, 0, 0, 1, 1, 0, 1, 1, 1, 0, 1, 0, 1, 1, 1, 0, 0, 0), nrow = 5, ncol = 5 , byrow = TRUE), use.expm = TRUE, use.eigen = FALSE, marginal = FALSE)

> fit

> round(fit$lik.anc, 3)

> plotTree(SpiderTree, type = "phylogram", fsize = 0.8, ftype = "i", label.offset = 1, jitter = 1)

> tiplabels(pie=to.matrix (PLS, sort(unique(PLS))), piecol = c("red", "black", "green", "blue", "yellow"), cex = 0.2)

> nodelabels(node=1:SpiderTree$Nnode+Ntip(SpiderTree), pie=fit$lik.anc, piecol= c("red", "black", "green", "blue", "yellow"), cex = 0.6)

> add.simmap.legend (leg = "Flagelliform", colors = c("red"), vertical = TRUE, prompt = TRUE, PLS=0.9*par()$usr[1], fsize=0.7)

> add.simmap.legend (leg = "Loss", colors = c("yellow"), vertical = TRUE, prompt = TRUE, PLS=0.9*par()$usr[1], fsize=0.7)

> add.simmap.legend (leg = "Modified", colors = c("black"), vertical = TRUE, prompt = TRUE, PLS=0.9*par()$usr[1], fsize=0.7)

> add.simmap.legend (leg = "None", colors = c("green"), vertical = TRUE, prompt = TRUE, PLS=0.9*par()$usr[1], fsize=0.7)

> add.simmap.legend (leg = "Pseudoflagelliform", colors = c("blue"), vertical = TRUE, prompt = TRUE, PLS=0.9*par()$usr[1], fsize=0.7)

**##Ancestral character estimation for four of the independent variables: foraging strategy, specific foraging strategy, type of silk utilized, variety of spigots possessed by spider species**

*##Ancestral character estimation for foraging strategy*

> library(ape)

> library(geiger)

> library(nlme)

> library (phytools)

> setwd("C:/Users/Rachael/Desktop/Comparative Ontogeny")

> SpiderTree <- read.newick ("ACE newick")

> plot(SpiderTree)

> is.ultrametric (SpiderTree)

> rel_calib1 <- makeChronosCalib(SpiderTree, node = "root", age.min = 1, age.max = 1, interactive = FALSE, soft.bounds = FALSE)

> timetree2 <- chronos(SpiderTree, lambda = 0, model = "relaxed", calibration = rel_calib1)

> plot(timetree2)

> is.ultrametric(timetree2)

> write.tree (timetree2, "ACE Charles")

> SpiderTree <- read.newick ("ACE Charles")

> plot(SpiderTree)

##Load Females dataset (updated version)

> rownames(Females) <- Females$Species

> Females <- Females[match(SpiderTree$tip.label,rownames(Females)),]

> name.check (SpiderTree, Females, data.names = NULL)

> PLS <- Females$Foraging

> PLS

> fit <- ace(x = PLS, phy = SpiderTree, type = "discrete", method = "ML", CI = TRUE, model = matrix(c(0, 1, 0, 0), nrow = 2, ncol = 2 , byrow = TRUE), use.expm = TRUE, use.eigen = FALSE, marginal = FALSE)

> fit

> round(fit$lik.anc, 3)

> plotTree(SpiderTree, type = "phylogram", fsize = 0.8, ftype = "i", label.offset = 1, jitter = 1)

> tiplabels(pie=to.matrix (PLS, sort(unique(PLS))), piecol = c("red", "blue"), cex = 0.2)

> nodelabels(node=1:SpiderTree$Nnode+Ntip(SpiderTree), pie=fit$lik.anc, piecol= c("red", "blue"), cex = 0.6)

> add.simmap.legend (leg = "0", colors = c("red"), vertical = TRUE, prompt = TRUE, PLS=0.9*par()$usr[1], fsize=0.7)

> add.simmap.legend (leg = "1", colors = c("blue"), vertical = TRUE, prompt = TRUE, PLS=0.9*par()$usr[1], fsize=0.7)

*##Ancestral character estimation for Specific foraging strategy*

> library(ape)

> library(geiger)

> library(nlme)

> library (phytools)

> setwd("C:/Users/Rachael/Desktop/Comparative Ontogeny")

> SpiderTree <- read.newick ("ACE newick")

> plot(SpiderTree)

> is.ultrametric (SpiderTree)

> rel_calib1 <- makeChronosCalib(SpiderTree, node = "root", age.min = 1, age.max = 1, interactive = FALSE, soft.bounds = FALSE)

> timetree2 <- chronos(SpiderTree, lambda = 0, model = "relaxed", calibration = rel_calib1)

> plot(timetree2)

> is.ultrametric(timetree2)

> write.tree (timetree2, "ACE Charles")

> SpiderTree <- read.newick ("ACE Charles")

> plot(SpiderTree)

##Load Females dataset (updated version)

> rownames(Females) <- Females$Species

> Females <- Females[match(SpiderTree$tip.label,rownames(Females)),]

> name.check (SpiderTree, Females, data.names = NULL)

> PLS <- Females$Specific

> PLS

> fit <- ace(x = PLS, phy = SpiderTree, type = "discrete", method = "ML", CI = TRUE, model = matrix(c(0, 1, 1, 2, 2, 0, 0, 0, 0, 1, 0, 1, 2, 2, 0, 0, 0, 0, 1, 1, 0, 1, 1, 0, 0, 0, 0, 1, 1, 1, 0, 1, 0, 0, 0, 0, 0, 0, 1, 1, 0, 0, 0, 0, 0, 1, 1, 2, 2, 3, 0, 1, 1, 1, 1, 1, 0, 0, 0, 1, 0, 0, 0, 1, 1, 2, 2, 3, 1, 1, 0, 1, 0, 0, 0, 0, 0, 0, 0, 0, 0), nrow = 9, ncol = 9 , byrow = TRUE), use.expm = TRUE, use.eigen = FALSE, marginal = FALSE)

> fit

> round(fit$lik.anc, 3)

> plotTree(SpiderTree, type = "phylogram", fsize = 0.8, ftype = "i", label.offset = 1, jitter = 1)

> tiplabels(pie=to.matrix (PLS, sort(unique(PLS))), piecol = c("red", "blue", "green", "yellow", "orange", "purple", "pink", "turquoise", "black"), cex = 0.2)

> nodelabels(node=1:SpiderTree$Nnode+Ntip(SpiderTree), pie=fit$lik.anc, piecol= c("red", "blue", "green", "yellow", "orange", "purple", "pink", "turquoise", "black"), cex = 0.6)

> add.simmap.legend (leg = "1", colors = c("red"), vertical = TRUE, prompt = TRUE, PLS=0.9*par()$usr[1], fsize=0.7)

> add.simmap.legend (leg = "1.5", colors = c("blue"), vertical = TRUE, prompt = TRUE, PLS=0.9*par()$usr[1], fsize=0.7)

> add.simmap.legend (leg = "2", colors = c("green"), vertical = TRUE, prompt = TRUE, PLS=0.9*par()$usr[1], fsize=0.7)

> add.simmap.legend (leg = "2.5", colors = c("yellow"), vertical = TRUE, prompt = TRUE, PLS=0.9*par()$usr[1], fsize=0.7)

> add.simmap.legend (leg = "3", colors = c("orange"), vertical = TRUE, prompt = TRUE, PLS=0.9*par()$usr[1], fsize=0.7)

> add.simmap.legend (leg = "4", colors = c("purple"), vertical = TRUE, prompt = TRUE, PLS=0.9*par()$usr[1], fsize=0.7)

> add.simmap.legend (leg = "4.5", colors = c("pink"), vertical = TRUE, prompt = TRUE, PLS=0.9*par()$usr[1], fsize=0.7)

> add.simmap.legend (leg = "5", colors = c("turquoise"), vertical = TRUE, prompt = TRUE, PLS=0.9*par()$usr[1], fsize=0.7)

> add.simmap.legend (leg = "5.5", colors = c("black"), vertical = TRUE, prompt = TRUE, PLS=0.9*par()$usr[1], fsize=0.7)

*##Ancestral character estimation for main Type of Silk utilized by the spider species*

> library(ape)

> library(geiger)

> library(nlme)

> library (phytools)

> setwd("C:/Users/Rachael/Desktop/Comparative Ontogeny")

> SpiderTree <- read.newick ("ACE newick")

> plot(SpiderTree)

> is.ultrametric (SpiderTree)

> rel_calib1 <- makeChronosCalib(SpiderTree, node = "root", age.min = 1, age.max = 1, interactive = FALSE, soft.bounds = FALSE)

> timetree2 <- chronos(SpiderTree, lambda = 0, model = "relaxed", calibration = rel_calib1)

> plot(timetree2)

> is.ultrametric(timetree2)

> write.tree (timetree2, "ACE Charles")

> SpiderTree <- read.newick ("ACE Charles")

> plot(SpiderTree)

##Load Females dataset (updated version)

> rownames(Females) <- Females$Species

> Females <- Females[match(SpiderTree$tip.label,rownames(Females)),]

> name.check (SpiderTree, Females, data.names = NULL)

> PLS <- Females$Type

> PLS

> fit <- ace(x = PLS, phy = SpiderTree, type = "discrete", method = "ML", CI = TRUE, model = matrix(c(0, 0, 0, 0, 0, 1, 0, 1, 0, 0, 1, 1, 0, 0, 0, 1, 0, 1, 0, 1, 1, 0, 1, 0, 0), nrow = 5, ncol = 5 , byrow = TRUE), use.expm = TRUE, use.eigen = FALSE, marginal = FALSE)

> fit

> round(fit$lik.anc, 3)

> plotTree(SpiderTree, type = "phylogram", fsize = 0.8, ftype = "i", label.offset = 1, jitter = 1)

> tiplabels(pie=to.matrix (PLS, sort(unique(PLS))), piecol = c("red", "blue", "green", "yellow", "black"), cex = 0.2)

> nodelabels(node=1:SpiderTree$Nnode+Ntip(SpiderTree), pie=fit$lik.anc, piecol= c("red", "blue", "green", "yellow", "black"), cex = 0.6)

> add.simmap.legend (leg = "1.0", colors = c("red"), vertical = TRUE, prompt = TRUE, PLS=0.9*par()$usr[1], fsize=0.7)

> add.simmap.legend (leg = "1.5", colors = c("blue"), vertical = TRUE, prompt = TRUE, PLS=0.9*par()$usr[1], fsize=0.7)

> add.simmap.legend (leg = "2.0", colors = c("green"), vertical = TRUE, prompt = TRUE, PLS=0.9*par()$usr[1], fsize=0.7)

> add.simmap.legend (leg = "3.0", colors = c("yellow"), vertical = TRUE, prompt = TRUE, PLS=0.9*par()$usr[1], fsize=0.7)

> add.simmap.legend (leg = "4.0", colors = c("black"), vertical = TRUE, prompt = TRUE, PLS=0.9*par()$usr[1], fsize=0.7)

*##Ancestral character estimation for variety of Spigot Types possessed by the spider species*

> library(ape)

> library(geiger)

> library(nlme)

> library (phytools)

> setwd("C:/Users/Rachael/Desktop/Comparative Ontogeny")

> SpiderTree <- read.newick ("ACE newick")

> plot(SpiderTree)

> is.ultrametric (SpiderTree)

> rel_calib1 <- makeChronosCalib(SpiderTree, node = "root", age.min = 1, age.max = 1, interactive = FALSE, soft.bounds = FALSE)

> timetree2 <- chronos(SpiderTree, lambda = 0, model = "relaxed", calibration = rel_calib1)

> plot(timetree2)

> is.ultrametric(timetree2)

> write.tree (timetree2, "ACE Charles")

> SpiderTree <- read.newick ("ACE Charles")

> plot(SpiderTree)

##Load Females dataset (updated version)

> rownames(Females) <- Females$Species

> Females <- Females[match(SpiderTree$tip.label,rownames(Females)),]

> name.check (SpiderTree, Females, data.names = NULL)

> PLS <- Females$Variety

> PLS

> fit <- ace(x = PLS, phy = SpiderTree, type = "discrete", method = "ML", CI = TRUE, model = matrix(c(0, 1, 2, 2, 2, 2, 2, 1, 0, 0, 0, 0, 0, 0, 1, 0, 0, 0, 0, 1, 0, 2, 0, 1, 0, 0, 2, 0, 2, 0, 0, 1, 0, 3, 1, 1, 1, 0, 0, 0, 0, 0, 2, 0, 2, 1, 1, 2, 0), nrow = 7, ncol = 7 , byrow = TRUE), use.expm = TRUE, use.eigen = FALSE, marginal = FALSE)

> fit

> round(fit$lik.anc, 3)

> plotTree(SpiderTree, type = "phylogram", fsize = 0.8, ftype = "i", label.offset = 1, jitter = 1)

> tiplabels(pie=to.matrix (PLS, sort(unique(PLS))), piecol = c("red", "blue", "green", "yellow", "orange", "purple", "black"), cex = 0.2)

> nodelabels(node=1:SpiderTree$Nnode+Ntip(SpiderTree), pie=fit$lik.anc, piecol= c("red", "blue", "green", "yellow", "orange", "purple", "black"), cex = 0.6)

> add.simmap.legend (leg = "1", colors = c("red"), vertical = TRUE, prompt = TRUE, PLS=0.9*par()$usr[1], fsize=0.7)

> add.simmap.legend (leg = "1.5", colors = c("blue"), vertical = TRUE, prompt = TRUE, PLS=0.9*par()$usr[1], fsize=0.7)

> add.simmap.legend (leg = "2", colors = c("green"), vertical = TRUE, prompt = TRUE, PLS=0.9*par()$usr[1], fsize=0.7)

> add.simmap.legend (leg = "2.5", colors = c("yellow"), vertical = TRUE, prompt = TRUE, PLS=0.9*par()$usr[1], fsize=0.7)

> add.simmap.legend (leg = "2.75", colors = c("orange"), vertical = TRUE, prompt = TRUE, PLS=0.9*par()$usr[1], fsize=0.7)

> add.simmap.legend (leg = "3", colors = c("purple"), vertical = TRUE, prompt = TRUE, PLS=0.9*par()$usr[1], fsize=0.7)

> add.simmap.legend (leg = "4", colors = c("black"), vertical = TRUE, prompt = TRUE, PLS=0.9*par()$usr[1], fsize=0.7)
